# An empirical evaluation of the impact scenario of pooling bodies of evidence from randomized controlled trials and cohort studies in medical research

**DOI:** 10.1186/s12916-022-02559-y

**Published:** 2022-10-24

**Authors:** Nils Bröckelmann, Julia Stadelmaier, Louisa Harms, Charlotte Kubiak, Jessica Beyerbach, Martin Wolkewitz, Jörg J. Meerpohl, Lukas Schwingshackl

**Affiliations:** 1 Institute for Evidence in Medicine, Medical Center - University of Freiburg, Faculty of Medicine, University of Freiburg, Freiburg, Germany; 2 Institute of Medical Biometry and Statistics, Faculty of Medicine and Medical Center - University of Freiburg, Freiburg, Germany; 3 Cochrane Germany, Cochrane Germany Foundation, Freiburg, Germany

**Keywords:** General medicine, Pooling, Meta-analysis, Cohort studies, Randomized controlled trials

## Abstract

**Background:**

Randomized controlled trials (RCTs) and cohort studies are the most common study design types used to assess treatment effects of medical interventions. We aimed to hypothetically pool bodies of evidence (BoE) from RCTs with matched BoE from cohort studies included in the same systematic review.

**Methods:**

BoE derived from systematic reviews of RCTs and cohort studies published in the 13 medical journals with the highest impact factor were considered. We re-analyzed effect estimates of the included systematic reviews by pooling BoE from RCTs with BoE from cohort studies using random and common effects models. We evaluated statistical heterogeneity, 95% prediction intervals, weight of BoE from RCTs to the pooled estimate, and whether integration of BoE from cohort studies modified the conclusion from BoE of RCTs.

**Results:**

Overall, 118 BoE-pairs based on 653 RCTs and 804 cohort studies were pooled. By pooling BoE from RCTs and cohort studies with a random effects model, for 61 (51.7%) out of 118 BoE-pairs, the 95% confidence interval (CI) excludes no effect. By pooling BoE from RCTs and cohort studies, the median *I*^2^ was 48%, and the median contributed percentage weight of RCTs to the pooled estimates was 40%. The direction of effect between BoE from RCTs and pooled effect estimates was mainly concordant (79.7%). The integration of BoE from cohort studies modified the conclusion (by examining the 95% CI) from BoE of RCTs in 32 (27%) of the 118 BoE-pairs, but the direction of effect was mainly concordant (88%).

**Conclusions:**

Our findings provide insights for the potential impact of pooling both BoE in systematic reviews. In medical research, it is often important to rely on both evidence of RCTs and cohort studies to get a whole picture of an investigated intervention-disease association. A decision for or against pooling different study designs should also always take into account, for example, PI/ECO similarity, risk of bias, coherence of effect estimates, and also the trustworthiness of the evidence. Overall, there is a need for more research on the influence of those issues on potential pooling.

**Supplementary Information:**

The online version contains supplementary material available at 10.1186/s12916-022-02559-y.

## Background

Randomized controlled trials (RCTs) and cohort studies are the most common study designs used to assess treatment effects of medical interventions [1, 2]. RCTs, if well-designed and well-conducted, are considered as the gold standard and are widely accepted as the ideal methodology for causal inference [1–3].

However, RCTs may not be available for certain medical treatments due to ethical reasons or may suffer from inherent methodological limitations such as low external validity [4]. On the other hand, cohort studies may often have higher external validity, but also a higher risk of confounding. It is generally considered that systematic reviews should be based on RCTs because these studies are more likely to provide unbiased information than other study designs [5].

According to recent GRADE guidance, cohort studies can be highly valuable and provide complementary, sequential, or replacement evidence for RCTs in a systematic review or other evidence syntheses [6]. However, the potential impact of integrating evidence from cohort studies in meta-analyses of RCTs in the medical field has not been investigated yet.

To close this important research gap, this empirical study aims to conduct a pooling scenario of bodies of evidence (BoE) from RCTs with matched BoE from cohort studies. We investigate the extent of how the integration of BoE from cohort studies modifies the conclusion of BoE of RCTs, the direction of effect estimates derived from BoE of RCTs, and its impact on statistical heterogeneity. Moreover, we will evaluate the contributed aggregated weights of RCTs to the pooled estimates, use random effects and common effects models for pooling, calculate 95% prediction intervals (PIs), and test for subgroup differences between BoE from RCTs and cohort studies.

## Methods

The sample of this empirical study was based on a large meta-epidemiological study [7], which was planned, written, and reported in adherence to current guidance for meta-epidemiological methodology research [8]. Eligibility criteria (PI/ECO: patient/population, intervention/exposure, comparator, and outcome) are reported in Table 1. Briefly, we included systematic reviews on medical interventions (or exposures) that included both RCTs and cohort studies for the same patient-relevant outcome and that performed meta-analyses for at least one BoE [7].

**Table 1:** Detailed description of inclusion and exclusion criteria

	Inclusion criteria	Exclusion criteria
Methods	Systematic review of interventions/exposure including RCTs and cohort studies; equivalent search for RCTs and cohort studies; performing quantitative meta-analysis for at least one BoE	Umbrella reviews, narrative reviews, systematic reviews of diagnostic test accuracy, individual patient data meta-analysis; no quantitative meta-analysis
BoE-pairs	BoE-pair with a BoE from RCTs and a BoE from cohort studies evaluating the same medical research question (e.g., association of exenatide with pancreatitis; effect of vitamin D on hypertension; comparing total with unicompartmental knee arthroplasty for range of movement of the knee)	Single small study (*n*<1000 participants) for one BoE (RCT or cohort studies); BoE-pair with one BoE using a continuous outcome and the other BoE using a binary outcome (e.g., risk of hypertension vs. mean difference of systolic blood pressure)
Population	All populations (e.g., primary prevention, secondary prevention, general population, adults, children)	-
Intervention/exposure	All types of medical interventions and exposures (e.g., drugs, invasive, procedures, nutrients, vaccines)	-
Comparator	All types of comparators (e.g., placebo, drugs, invasive, procedures, nutrients, vaccines)	-
Outcomes	Patient-relevant outcomes (e.g., mortality, cancer outcomes, cardiovascular outcomes, obstetrical outcomes) and of intermediate disease markers (e.g., LDL-cholesterol)	-
Study design	Randomized controlled trials (e.g., parallel, cluster, factorial, cross-over); cohort studies (e.g., prospective cohort, retrospective cohort, observational cohort analysis of RCTs)	Quasi-RCTs, non-randomized controlled trials, case-control studies, cross-sectional studies, ecological studies

*BoE* Bodies of evidence, *LDL* Low-density lipoprotein, *RCT* Randomized controlled trial

### Identification of systematic reviews of RCTs and cohort studies

The original search for the meta-epidemiological study was conducted in MEDLINE on 04.05.2020 for the period between 01.01.2010 and 31.12.2019 in the 13 medical journals with the highest impact factor (according to the Journal Citation Report [JCR] 2018 category general and internal medicine). Initially, we planned to include the ten highest impact factor journals, but three journals did not publish any systematic review with an eligible BoE-pair. We therefore included the subsequent three journals according to the JCR 2018. The search strategy including the list of considered journals is given in Additional file 1: Appendix S1. Title and abstract screening was conducted by one reviewer (NB), and potentially relevant full texts were screened for eligibility by two reviewers independently (NB, LS). Any discrepancies were resolved by discussion.

For each included BoE from a systematic review, we included a maximum of three patient-relevant outcomes (e.g., mortality) and a maximum of three intermediate disease markers (e.g., blood lipids). If more than three outcomes were available for a given systematic review, we included the primary outcomes and thereafter we used a top-down approach (mentioned first). We evaluated the similarity of the PI/ECO criteria between BoE-pair from RCTs and cohort studies within each systematic review. For each BoE-pair, the similarity of each PI/ECO domain was rated as “more or less identical,” “similar but not identical,” or “broadly similar” (Additional file 1: Table S1). A detailed description of identification and evaluating similarity BoE-pairs can be found elsewhere [7].

### Data extraction

Two reviewers (NB, LH) extracted the following data for each included BoE-pair into a piloted data extraction sheet: name of the first author, year of publication, type of intervention/exposure (e.g., antiretroviral therapy), description of the comparator, effect estimates (risk ratio [RR], hazard ratio [HR], odds ratio [OR], mean difference [MD], including 95% confidence interval [CI]), and number of studies. A detailed description of data extraction can be found elsewhere [7]. For the current analysis, we additionally extracted all effect estimates and corresponding 95% CI of the primary studies included for a relevant BoE (NB, LH).

### Statistical analysis

For our pooling scenario, we re-analyzed the effect estimates of all eligible systematic reviews in a two-step approach: For each identified BoE-pair, we first pooled the effect estimates obtained from RCTs and cohort studies separately using a random effects model. Primary studies based on inappropriate study designs (i.e., case-control, cross-sectional, and quasi-RCTs) were excluded.

Second, we pooled the BoE from RCTs with the BoE from cohort studies with a random effects model for each BoE-pair. Binary outcomes (pooled as RRs, HRs, or ORs) and continuous outcomes (pooled as MDs on the same scale) were considered for analysis. Random effects models were used to account for potential between-study heterogeneity. For the sensitivity analysis, we used a common effects model to evaluate whether this hypothetical scenario is more conservative for pooling BoE from RCTs and cohort studies.

To explore the impact of including cohort studies on pooled effect estimates by combining BoE from RCTs and cohort studies (with or without subgroups), we compared the results and conclusions (95% CI including vs. excluding the null effect) between the BoE of RCTs only and that including both RCTs and cohort studies. Then, we evaluated the contributed weight of RCTs to the pooled estimates and conducted a statistical test for subgroup differences between the two types of BoE. A *p*-value < 0.05 was considered as statistical significant.

In an additional analysis, we used effect estimates of cohort studies as a reference and compared the results and conclusion between the BoE of cohort studies only and that including both, RCTs and cohort studies.

Heterogeneity in meta-analyses was tested with a standard *χ*^2^ test. We quantified any inconsistency by using the *I*^2^ parameter: *I*^2^=((*Q*−df))/*Q* × 100%, where *Q* is the *χ*^2^ statistic and df is its degrees of freedom [9]. An *I*^2^-value of greater than 50% was considered to represent considerable heterogeneity [10]. For binary outcomes, we additionally calculated *τ*^*2*^, which is independent of study size and describes variability between studies in relation to the risk estimates [11]. For continuous outcomes, we did not calculate *τ*^2^ due to the use of different scales between meta-analyses (blood pressure [mmHg] or body weight [kg]. Meta-analyses were conducted using Review Manager (RevMan) version 5.3 [12].

Whereas in a random effects meta-analysis, the focus is usually on the average treatment effect and its 95% CI, the calculation of a prediction interval (95% PI) also considers the potential treatment effect within an individual study setting, as this may differ from the average effect [11]. 95% PIs were calculated for the summary random effects for each meta-analysis since they further account for the degree of between-study heterogeneity and give a range for which we are 95% confident that the effect in a new study examining the same association lies within [11]. Calculations of 95% PI were conducted with Stata 15.

## Results

Overall, 64 systematic reviews of RCTs and cohort studies were included [13–76]. Of the identified 129 outcome pairs, 118 from 59 systematic reviews were included in the present pooling scenario and re-analyzed (Additional file 1: Table S2-S3) [7, 13–76] (109 dichotomous and nine continuous outcomes) (Additional file 1: Figs. S1-118). Eleven outcome pairs from five systematic reviews [13, 26, 73–75] were excluded from the current analysis. Reasons for exclusion are provided in Additional file 1: Table S4.

Our sample of 118 BoE-pairs was based on 653 RCTs and 804 cohort studies. Detailed study characteristics including a description of the population, intervention/comparator, outcomes, range of study length, and risk of bias/study quality of primary studies included for each outcome pair have been described elsewhere [7].

Two of the outcome pairs were classified (PI/ECO similarity degree) as “more or less identical” and 82 as “similar but not identical,” whereas 34 were classified as “broadly similar.” Out of the 118 BoE from RCTs, for 39 (33.1%), the 95% CI excludes no effect, whereas for the BoE for cohort studies, 58 (49.2%) indicated a 95% CI excluding no effect. Twenty-four (20.3%) out of 118 BoE-pairs showed simultaneously for BoE from RCTs and BoE from cohort studies a 95% CI excluding no effect and a concordant direction of effect. The median *I*^2^ was 5% (*τ*^2^=0) across BoE from RCTs and 41% (*τ*^2^=0.03) across BoE from cohort studies, whereas the mean *I*^2^ was 23% (*τ*^2^=0.14) and 42% (*τ*^2^=0.18), respectively. Table 2 shows the summary effects of the BoE from RCTs, cohort studies, and the pooling scenario.

**Table 2:** Pooling results of bodies of evidence from RCTs with cohort studies based on random effects and common effects models, 95% prediction intervals, heterogeneity, test for subgroup difference, and population (P), intervention (I)/exposure (E), comparator (C), and outcome similarity degree

Author, year, and reference	Intervention/exposure	Outcome	BoE RCTs, *n*	Effect estimate (95% CI)	*I*^2^ (%)/tau^2^	BoE CSs, *n*	Effect estimate (95% CI)	*I*^2^ (%)/tau^2^	Pooled effect estimate (95%) RE (95% prediction interval)	*I*^2^ (%)/tau^2^	Weight RCTs (%)	RCT conclusion modified	Test for subgroup difference (*p*-value)	Pooled effect estimate (95%) CE	Degree of PI/ECO similarity^*^
Aburto 2013 [14]	Low sodium	Mortality	4	RR: 0.70 (0.44, 1.14)	0/0.00	7	RR: 0.94 (0.83, 1.06)	61/0.02	RR: 0.93 (0.83, 1.04) (0.68, 1.26)	47/0.02	5.0	N	0.25	RR: 0.94 (0.88, 1.00)	2
Aburto 2013 [14]	Low sodium	Cardiovascular disease	2	RR: 0.84 (0.57, 1.23)	0/0.00	9	RR: 0.90 (0.75, 1.08)	78/0.07	RR: 0.89 (0.75, 1.06) (0.49, 1.62)	74/0.07	8.7	N	0.78	RR: 0.86 (0.80, 0.93)	2
Ahmad 2015 [15]	Intra-aortic balloon pump	Mortality	12	OR: 0.96 (0.74, 1.24)	0/0.00	14	OR: 1.02 (0.57, 1.82)	97/1.03	OR: 1.02 (0.67, 1.56) (0.14, 7.32)	95/0.86	37.8	N	0.85	OR: 0.76 (0.72, 0.82)	1
Alipanah 2018 [16]	Self-administered therapy	Treatment success	4	RR: 0.95 (0.87, 1.03)	25/0.00	16	RR: 0.81 (0.74, 0.88)	91/0.02	RR: 0.84 (0.78, 0.90) (0.62, 1.14)	89/0.02	19.1	Y	0.01	RR: 0.92 (0.90, 0.94)	3
Alipanah 2018 [16]	Self-administered therapy	Treatment completion	5	RR: 0.79 (0.57, 1.09)	45/0.06	14	RR: 1.10 (0.91, 1.33)	86/0.07	RR: 1.02 (0.84, 1.23) (0.51, 2.02)	88/0.10	24.4	N	0.08	RR: 1.12 (1.07, 1.17)	3
Alipanah 2018 [16]	Self-administered therapy	Mortality	4	RR: 0.73 (0.45, 1.19)	0/0.00	23	RR: 1.35 (1.00, 1.83)	90/0.34	RR: 1.26 (0.95, 1.67) (0.37, 4.28)	88/0.33	9.8	N	0.04	RR: 1.26 (1.18, 1.34)	3
Anglemyer 2013 [17]	Antiretroviral therapy	HIV infection	1	RR: 0.11 (0.04, 0.30)	NA	9	RR: 0.59 (0.36, 0.97)	63/0.25	RR: 0.45 (0.26, 0.78) (0.09, 2.31)	75/0.42	11.8	N	0.003	RR: 0.72 (0.64, 0.82)	3
Azad 2017 [18]	Non-nutritive sweeteners	BMI	3	MD: −0.37 (−1.10, 0.36)	9/0.07	1	MD: 0.77 (0.47, 1.07)	NA	MD: 0.23 (−0.77, 1.23) (−3.88, 4.34)	79/0.65	61.4	N	0.005	MD: 0.53 (0.26, 0.80)	2
Barnard 2015 [19]	Surgical abortion by mid-level providers	Failure or incomplete abortion	2	RR: 2.84 (0.24, 32.97)	65/2.22	2	RR: 2.47 (1.44, 4.23)	0/0.00	RR: 2.23 (1.15, 4.32) (0.24, 20.54)	33/0.15	34.5	Y	0.91	RR: 2.14 (1.35, 3.39)	2
Barnard 2015 [19]	Surgical abortion by mid-level providers	Complications	2	RR: 0.94 (0.14, 6.44)	0/0.00	2	RR: 1.30 (0.57, 2.96)	70/0.26	RR: 1.31 (0.70, 2.42) (0.17, 10.11)	32/0.13	9.7	N	0.76	RR: 1.51 (1.05, 2.17)	2
Barnard 2015 [19]	Surgical abortion by mid-level providers	Abortion failure and complications	2	RR: 2.93 (0.19, 44.15)	72/2.90	3	RR: 1.33 (0.78, 2.27)	74/0.16	RR: 1.36 (0.83, 2.21) (0.29, 6.32)	65/0.17	19.5	N	0.58	RR: 1.43 (1.12, 1.82)	2
Bellemain-Appaix 2012 [20]	Clopidogrel pretreatment for percutaneous coronary intervention	Mortality	7	OR: 0.80 (0.58, 1.10)	0/0.00	8	OR: 0.79 (0.53, 1.18)	79/0.23	OR: 0.77 (0.57, 1.04) (0.30, 2.02)	66/0.17	30.7	N	0.96	OR: 0.65 (0.57, 0.75)	2
Bellemain-Appaix 2012 [20]	Clopidogrel pretreatment for percutaneous coronary intervention	Major bleeding	7	OR: 1.18 (0.93, 1.50)	0/0.00	8	OR: 1.03 (0.69, 1.53)	64/0.16	OR: 1.04 (0.81, 1.33) (0.54, 2.03)	46/0.08	40.4	N	0.56	OR: 1.07 (0.92, 1.24)	2
Bellemain-Appaix 2012 [20]	Clopidogrel pretreatment for percutaneous coronary intervention	Major coronary event	7	OR: 0.77 (0.66, 0.89)	4/0.00	8	OR: 0.76 (0.60, 0.95)	82/0.08	OR: 0.76 (0.65, 0.89) (0.45, 1.29)	69/0.05	36.0	N	0.92	OR: 0.78 (0.73, 0.85)	2
Bellemain-Appaix 2014 [21]	P2Y12 inhibitor pretreatment in non-ST elevation acute coronary syndrome	Mortality	3	OR: 0.90 (0.71, 1.14)	5/0.01	4	OR: 0.69 (0.35, 1.32)	35/0.17	OR: 0.90 (0.75, 1.07) (0.65, 1.24)	10/0.01	66.8	N	0.44	OR: 0.91 (0.80, 1.04)	2
Bellemain-Appaix 2014 [21]	P2Y12 inhibitor pretreatment in non-ST elevation acute coronary syndrome	Major bleeding	3	OR: 1.43 (1.16, 1.76)	0/0.00	4	OR: 1.13 (0.92, 1.39)	0/0.00	OR: 1.27 (1.10, 1.47) (1.05, 1.54)	0/0.00	49.7	N	0.11	OR: 1.27 (1.10, 1.47)	2
Bellemain-Appaix 2014 [21]	P2Y12 inhibitor pretreatment in non-ST elevation acute coronary syndrome	Main composite ischemic endpoint	3	OR: 0.87 (0.73, 1.04)	48/0.01	4	OR: 0.78 (0.56, 1.08)	65/0.07	OR: 0.84 (0.72, 0.98) (0.56, 1.26)	52/0.02	55.0	Y	0.55	OR: 0.85 (0.78, 0.93)	2
Bloomfield 2016 [22]	Mediterranean diet	Breast cancer	1	RR: 0.43 (0.21, 0.88)	NA	13	RR: 0.96 (0.90, 1.03)	52/0.01	RR: 0.95 (0.89, 1.02) (0.78, 1.17)	57/0.01	0.9	Y	0.03	RR: 0.98 (0.95, 1.02)	2
Bolland 2015 [23]	High calcium	All fractures	22	RR: 0.90 (0.83, 0.97)	23/0.00	5	RR: 1.02 (0.93, 1.12)	68/0.01	RR: 0.94 (0.88, 1.00) (0.78, 1.14)	50/0.01	58.0	Y	0.04	RR: 0.99 (0.96, 1.02)	2
Bolland 2015 [23]	High calcium	Vertebral fracture	12	RR: 0.86 (0.74, 1.00)	0/0.00	1	RR: 1.40 (1.10, 1.78)	NA	RR: 0.94 (0.79, 1.11) (0.65, 1.34)	22/0.02	76.7	N	0.0007	RR: 0.98 (0.87, 1.11)	2
Bolland 2015 [23]	High calcium	Hip fracture	13	RR: 0.95 (0.76, 1.18)	36/0.04	6	RR: 1.09 (0.91, 1.30)	50/0.03	RR: 1.02 (0.89, 1.18) (0.67, 1.56)	46/0.04	42.8	N	0.34	RR: 0.98 (0.91, 1.07)	2
Brenner 2014 [24]	Sigmoidoscopy, screening for CRC	Colorectal cancer mortality	4	RR: 0.72 (0.65, 0.80)	0/0.00	1	RR: 0.59 (0.45, 0.77)	NA	RR: 0.70 (0.64, 0.77) (0.60, 0.82)	0/0.00	87.3	N	0.18	RR: 0.70 (0.64, 0.77)	1
Brenner 2014 [24]	Sigmoidoscopy, screening for CRC	Colorectal cancer incidence	4	RR: 0.82 (0.75, 0.90)	51/0.00	2	RR: 0.50 (0.37, 0.69)	0/0.00	RR: 0.78 (0.69, 0.89) (0.55, 1.11)	65/0.01	89.0	N	0.003	RR: 0.79 (0.74, 0.84)	2
Chowdhury 2012 [25]	High omega-3-fatty acids	Cerebrovascular disease	2	RR: 0.99 (0.90, 1.08)	10/0.00	10	RR: 0.89 (0.80, 0.99)	17/0.01	RR: 0.93 (0.85, 1.01) (0.78, 1.10)	21/0.00	40.7	N	0.17	RR: 0.95 (0.89, 1.01)	2
Chowdhury 2014a [76]	High α-linolenic acid	Coronary event	4	RR: 0.97 (0.69, 1.36)	52/0.06	7	RR: 0.99 (0.88, 1.11)	61/0.02	RR: 0.99 (0.88, 1.11) (0.72, 1.37)	54/0.02	21.4	N	0.92	RR: 1.01 (0.95, 1.08)	3
Chowdhury 2014a [76]	High omega-3-fatty acids	Coronary event	17	RR: 0.94 (0.86, 1.03)	17/0.01	16	RR: 0.87 (0.78, 0.97)	76/0.03	RR: 0.90 (0.83, 0.97) (0.66, 1.22)	61/0.02	38.1	Y	0.26	RR: 0.93 (0.89, 0.97)	3
Chowdhury 2014a [76]	High omega-6-fatty acids	Coronary event	8	RR: 0.86 (0.69, 1.07)	59/0.05	8	RR: 0.98 (0.90, 1.06)	54/0.01	RR: 0.94 (0.87, 1.03) (0.73, 1.21)	56/0.01	30.0	N	0.27	RR: 0.96 (0.94, 1.01)	3
Chung 2016 [27]	High calcium	Cardiovascular disease mortality	2	RR: 1.05 (0.82, 1.33)	0/0.00	6	RR: 0.97 (0.86, 1.09)	37/0.01	RR: 0.99 (0.92, 1.07) (0.86, 1.15)	11/0.00	10.1	N	0.58	RR: 1.01 (0.95, 1.07)	2
Ding 2017 [28]	High dairy	Systolic blood pressure	8	MD: −0.21 (−0.98, 0.57)	0/0.00	27	MD: −0.11 (−0.20, −0.02)	30/0.01	MD: −0.11 (−0.20, −0.03) (−0.34, 0.11)	24/0.01	1.2	Y	0.80	MD: −0.16 (−0.21, −0.11)	2
Fenton 2018 [29]	Radiation therapy	Erectile dysfunction	1	RR: 0.91 (0.77, 1.08)	NA	7	RR: 1.30 (1.19, 1.43)	31/0.00	RR: 1.24 (1.09, 1.41) (0.83, 1.86)	70/0.02	14.3	Y	0.0003	RR: 1.23 (1.15, 1.32)	2
Fenton 2018 [29]	Radical prostatectomy	Urinary incontinence	3	RR: 2.25 (1.80, 2.82)	0/0.00	5	RR: 2.91 (1.80, 4.71)	67/0.18	RR: 2.54 (1.97, 3.27) (1.28, 5.03)	51/0.06	47.9	N	0.34	RR: 2.46 (2.08, 2.90)	2
Fenton 2018 [29]	Radical prostatectomy	Erectile dysfunction	3	RR: 1.60 (1.24, 2.07)	87/0.05	6	RR: 1.49 (1.33, 1.66)	63/0.01	RR: 1.53 (1.37, 1.70) (1.07, 2.18)	75/0.02	34.9	N	0.62	RR: 1.50 (1.42, 1.58)	2
Filippini 2017 [30]	Disease-modifying drugs	Conversion to clinically definite multiple sclerosis	7	HR: 0.52 (0.46, 0.60)	0/0.00	2	HR: 0.48 (0.30, 0.78)	62/0.08	HR: 0.53 (0.47, 0.59) (0.46, 0.61)	0/0.00	70.0	N	0.74	HR: 0.53 (0.47, 0.59)	2
Fluri 2010 [31]	Extracranial-intracranial arterial bypass	Mortality	2	OR: 0.81 (0.62, 1.05)	0/0.00	11	OR: 0.97 (0.58, 1.62)	0/0.00	OR: 0.84 (0.66, 1.06) (0.64, 1.09)	0/0.00	79.7	N	0.54	OR: 0.84 (0.66, 1.06)	2
Fluri 2010 [31]	Extracranial-intracranial arterial bypass	Any stroke	2	OR: 0.44 (0.06, 3.21)	85/1.80	15	OR: 0.76 (0.49, 1.17)	2/0.02	OR: 0.77 (0.50, 1.17) (0.29, 2.05)	29/0.17	32.5	N	0.60	OR: 0.95 (0.78, 1.16)	2
Fluri 2010 [31]	Extracranial-intracranial arterial bypass	Death or dependency	1	OR: 0.94 (0.74, 1.21)	NA	8	OR: 0.81 (0.50, 1.31)	0/0.00	OR: 0.91 (0.73, 1.14) (0.70, 1.19)	0/0.00	79.4	N	0.59	OR: 0.91 (0.73, 1.14)	2
Gargiulo 2016 [32]	Transcatheter aortic valve implantation	Early mortality	5	OR: 0.80 (0.58, 1.11)	0/0.00	29	OR: 1.08 (0.84, 1.39)	41/0.16	OR: 1.01 (0.81, 1.26) (0.47, 2.20)	39/0.13	18.4	N	0.16	OR: 1.02 (0.88, 1.19)	2
Gargiulo 2016 [32]	Transcatheter aortic valve implantation	Mid-term mortality	5	OR: 0.90 (0.71, 1.13)	22/0.01	18	OR: 1.00 (0.81, 1.24)	46/0.08	OR: 0.96 (0.82, 1.13) (0.59, 1.58)	40/0.05	29.0	N	0.49	OR: 0.93 (0.83, 1.04)	2
Gargiulo 2016 [32]	Transcatheter aortic valve implantation	Long-term mortality	4	OR: 1.03 (0.77, 1.37)	65/0.05	6	OR: 1.70 (1.31, 2.20)	0/0.00	OR: 1.28 (1.00, 1.65) (0.62, 2.66)	62/0.08	53.3	N	0.01	OR: 1.18 (1.03, 1.35)	2
Hartling 2013 [33]	Treating gestational diabetes mellitus	Birth weight > 4000g	5	RR: 0.50 (0.36, 0.71)	49/0.07	6	RR: 0.69 (0.31, 1.54)	88/0.64	RR: 0.58 (0.40, 0.86) (0.17, 2.01)	79/0.25	51.7	N	0.49	RR: 0.54 (0.46, 0.63)	2
Hartling 2013 [33]	Treating gestational diabetes mellitus	Large-for-gestational age neonate	3	RR: 0.56 (0.45, 0.69)	0/0.00	4	RR: 0.43 (0.27, 0.70)	58/0.13	RR: 0.47 (0.36, 0.62) (0.22, 1.02)	60/0.07	50.2	N	0.35	RR: 0.45 (0.39, 0.52)	2
Hartling 2013 [33]	Treating gestational diabetes mellitus	Shoulder dystocia	3	RR: 0.42 (0.23, 0.77)	0/0.00	4	RR: 0.38 (0.19, 0.75)	16/0.09	RR: 0.39 (0.26, 0.60) (0.23, 0.68)	0/0.00	49.9	N	0.81	RR: 0.39 (0.26, 0.60)	2
Henderson 2019 [34]	Treating asymptomatic bacteriuria	Pyelonephritis	12	RR: 0.24 (0.14, 0.40)	56/0.40	2	RR: 0.29 (0.15, 0.57)	0/0.00	RR: 0.25 (0.16, 0.39) (0.07, 0.87)	48/0.28	84.9	N	0.64	RR: 0.30 (0.23, 0.40)	3
Higgins 2016 [35]	BCG	Mortality	3	RR: 0.67 (0.40, 1.14)	58/0.11	8	RR: 0.46 (0.30, 0.69)	63/0.19	RR: 0.51 (0.36, 0.72) (0.18, 1.46)	67/0.19	29.9	Y	0.25	RR: 0.57 (0.48, 0.68)	3
Higgins 2016 [35]	Measles containing vaccines	Mortality	4	RR: 0.74 (0.51, 1.07)	0/0.00	13	RR: 0.53 (0.40, 0.70)	67/0.14	RR: 0.57 (0.45, 0.72) (0.27, 1.21)	58/0.11	19.6	Y	0.15	RR: 0.65 (0.57, 0.74)	3
Hopley 2010 [36]	Total hip arthroplasty	Reoperation	4	RR: 1.09 (0.40, 2.99)	30/0.31	6	RR: 0.45 (0.19, 1.08)	23/0.28	RR: 0.66 (0.33, 1.32) (0.13, 3.42)	34/0.39	43.8	N	0.19	RR: 0.72 (0.43, 1.20)	2
Hopley 2010 [36]	Total hip arthroplasty	Dislocation	4	RR: 2.47 (0.69, 8.76)	0/0.00	5	RR: 0.79 (0.27, 2.35)	18/0.28	RR: 1.20 (0.52, 2.76) (0.28, 5.08)	12/0.17	36.3	N	0.18	RR: 1.16 (0.54 2.52)	2
Hopley 2010 [36]	Total hip arthroplasty	Deep infection	4	RR: 1.71 (0.66, 4.45)	0/0.00	4	RR: 0.91 (0.25, 3.28)	0/0.00	RR: 1.37 (0.64, 2.94) (0.50, 3.73)	0/0.00	64.1	N	0.44	RR: 1.37 (0.64, 2.94)	2
Hüpfl 2010 [37]	Chest-compression-only cardiopulmonary resuscitation	Survival	3	RR: 1.22 (1.01, 1.46)	0/0.00	7	RR: 0.96 (0.83, 1.11)	0/0.00	RR: 1.04 (0.92, 1.19) (0.83, 1.31)	13/0.01	38.1	Y	0.05	RR: 1.05 (0.93, 1.18)	3
Jamal 2013 [38]	Non-calcium-based phosphate binders	Mortality	8	RR: 0.78 (0.62, 0.98)	43/0.03	3	RR: 0.89 (0.78, 1.00)	0/0.00	RR: 0.87 (0.77, 0.97) (0.67, 1.12)	28/0.01	50.9	N	0.34	RR: 0.89 (0.82, 0.96)	2
Jefferson 2010 [39]	Parenteral influenza vaccine	Influenza-like illness	4	RR: 0.59 (0.47, 0.73)	0/0.00	30	RR: 0.76 (0.66, 0.87)	57/0.07	RR: 0.73 (0.64, 0.82) (0.43, 1.22)	54/0.06	14.4	N	0.05	RR: 0.70 (0.65, 0.75)	3
Jefferson 2010 [39]	Parenteral influenza vaccine	Influenza	3	RR: 0.42 (0.27, 0.66)	0/0.00	10	RR: 0.51 (0.27, 0.97)	64/0.52	RR: 0.51 (0.32, 0.80) (0.13, 2.02)	59/0.34	31.3	N	0.62	RR: 0.60 (0.47, 0.78)	2
Jefferson 2012 [40]	Inactivated influenza vaccines	Influenza	5	RR: 0.41 (0.29, 0.59)	36/0.08	1	RR: 0.20 (0.10, 0.39)	NA	RR: 0.37 (0.26, 0.53) (0.15, 0.92)	44/0.11	84.8	N	0.07	RR: 0.34 (0.27, 0.43)	2
Jefferson 2012 [40]	Inactivated influenza vaccines	Influenza-like illness	5	RR: 0.64 (0.54, 0.76)	67/0.02	2	RR: 0.29 (0.07, 1.15)	95/1.43	RR: 0.56 (0.46, 0.68) (0.33, 0.94)	87/0.04	65.2	N	0.26	RR: 0.74 (0.71, 0.77)	2
Jin 2012 [41]	High total flavonoids	Colorectal neoplasms	1	RR: 1.09 (0.93, 1.28)	NA	3	RR: 1.00 (0.80, 1.25)	66/0.02	RR: 1.03 (0.88, 1.20) (0.56, 1.88)	56/0.01	30.4	N	0.55	RR: 1.02 (0.93, 1.13)	3
Kansagara 2013 [42]	Transfusion	Mortality	6	RR: 0.94 (0.62, 1.43)	17/0.05	11	RR: 2.49 (1.40, 4.43)	97/0.94	RR: 1.84 (1.10, 3.07) (0.20, 16.54)	96/1.00	25.6	Y	0.007	RR: 3.32 (3.03, 3.65)	3
Keag 2018 [43]	Caesarean section	Urinary incontinence	1	OR: 0.78 (0.56, 1.08)	NA	8	OR: 0.56 (0.48, 0.66)	70/0.04	OR: 0.58 (0.50, 0.68) (0.36, 0.94)	68/0.04	10.0	Y	0.08	OR: 0.62 (0.57, 0.67)	3
Keag 2018 [43]	Caesarean section	Fecal incontinence	1	OR: 3.07 (0.90, 10.47)	NA	5	OR: 1.04 (0.73, 1.48)	72/0.10	OR: 1.11 (0.78, 1.58) (0.38, 3.26)	71/0.12	6.4	N	0.10	OR: 1.11 (0.94, 1.31)	3
Kredo 2014 [44]	Antiretroviral therapy by nurses	Mortality	1	RR: 0.96 (0.82, 1.12)	NA	2	RR: 1.23 (1.14, 1.33)	0/0.00	RR: 1.13 (0.94, 1.36) (0.13, 9.93)	76/0.02	35.3	N	0.004	RR: 1.17 (1.10, 1.26)	3
Kredo 2014 [44]	Antiretroviral therapy by nurses	Attrition	1	RR: 0.73 (0.55, 0.97)	NA	2	RR: 0.30 (0.05, 1.94)	98/1.77	RR: 0.43 (0.21, 0.86) (0.00, 2691.24)	95/0.35	34.4	N	0.35	RR: 0.75 (0.71, 0.79)	3
Kredo 2014 [44]	Nurses for maintenance of antiretroviral therapy	Mortality	2	RR: 0.89 (0.59, 1.32)	0/0.00	1	RR: 0.19 (0.05, 0.78)	NA	RR: 0.61 (0.28, 1.35) (0.00, 2756.46)	56/0.28	79.8	N	0.04	RR: 0.79 (0.54, 1.16)	3
Li 2014 [45]	Exenatide	Acute pancreatitis/admission for acute pancreatitis	5	RR: 0.86 (0.22, 3.39)	0/0.00	2	RR: 0.92 (0.69, 1.22)	0/0.00	RR: 0.92 (0.69, 1.22) (0.64, 1.32)	0/0.00	4.0	N	0.92	RR: 0.92 (0.69, 1.21)	2
Li 2016 [46]	DDP-4 inhibitors	Heart failure	34	RR: 0.95 (0.60, 1.50)	0/0.00	4	RR: 1.10 (1.04, 1.17)	0/0.00	RR: 1.10 (1.04, 1.17) (1.03, 1.16)	0/0.00	1.6	Y	0.53	RR: 1.10 (1.04, 1.17)	2
Li 2016 [46]	DDP-4 Inhibitors	Hospital admission for heart failure	5	OR: 1.13 (1.00, 1.27)	0/0.00	6	OR: 0.85 (0.74, 0.97)	33/0.01	OR: 0.94 (0.83, 1.08) (0.66, 1.36)	55/0.02	41.9	N	0.002	OR: 0.97 (0.90, 1.05)	2
Matthews 2018 [47]	Tamoxifen	Heart failure	1	RR: 0.52 (0.33, 0.79)	NA	2	RR: 0.85 (0.66, 1.09)	10/0.00	RR: 0.74 (0.53, 1.04) (0.02, 29.27)	59/0.05	29.5	Y	0.05	RR: 0.75 (0.61, 0.92)	3
Menne 2019 [48]	SGLT-2 inhibitors	Acute kidney injury	41	OR: 0.75 (0.66, 0.84)	0/0.00	5	OR: 0.40 (0.31, 0.52)	39/0.03	OR: 0.58 (0.49, 0.69) (0.41, 0.99)	27/0.05	63.1	N	<0.0001	OR: 0.62 (0.56, 0.68)	2
Mesgarpour 2017 [49]	Erythropoiesis stimulating agents	Venous thromboembolism	12	RR: 1.12 (0.90, 1.40)	9/0.01	5	RR: 1.92 (0.64, 5.76)	75/1.03	RR: 1.26 (0.76, 2.10) (0.20, 8.14)	84/0.70	71.6	N	0.35	RR: 1.71 (1.45, 2.01)	2
Mesgarpour 2017 [49]	Erythropoiesis stimulating agents	Mortality	17	RR: 0.82 (0.71, 0.93)	0/0.00	7	RR: 1.08 (0.66, 1.78)	91/0.35	RR: 0.88 (0.64, 1.21) (0.21, 3.71)	92/0.46	66.5	Y	0.28	RR: 2.20 (2.15, 2.25)	2
Moberley 2013 [50]	Pneumococcal polysaccharide vaccines	Invasive pneumococcal disease	10	OR: 0.26 (0.14, 0.45)	0/0.00	2	OR: 0.57 (0.36, 0.89)	0/0.00	OR: 0.40 (0.26, 0.61) (0.18, 0.85)	12/0.07	48.1	N	0.03	OR: 0.42 (0.29, 0.59)	2
Molnar 2015 [51]	Neoral (cyclosporine)	Acute rejection of kidney transplant	2	OR: 1.25 (0.61, 2.56)	9/0.03	2	OR: 0.46 (0.25, 0.86)	5/0.02	OR: 0.74 (0.36, 1.54) (0.04, 12.62)	56/0.29	49.6	N	0.04	OR: 0.71 (0.46, 1.10)	2
Navarese 2013 [52]	Early intervention for NSTE-ACS	Mortality	7	OR: 0.83 (0.64, 1.09)	0/0.00	4	OR: 0.80 (0.63, 1.02)	78/0.04	OR: 0.82 (0.69, 0.97) (0.54, 1.24)	45/0.03	25.2	Y	0.86	OR: 0.86 (0.80, 0.94)	2
Navarese 2013 [52]	Early intervention for NSTE-ACS	Myocardial infarction	7	OR: 1.16 (0.67, 2.00)	81/0.41	3	OR: 0.86 (0.69, 1.08)	86/0.03	OR: 0.97 (0.77, 1.22) (0.48, 1.94)	81/0.08	50.6	N	0.32	OR: 0.90 (0.83, 0.97)	2
Navarese 2013 [52]	Early intervention for NSTE-ACS	Major bleeding	7	OR: 0.76 (0.56, 1.04)	0/0.00	3	OR: 1.12 (0.69, 1.82)	92/0.17	OR: 0.92 (0.68, 1.24) (0.39, 2.15)	70/0.11	43.7	N	0.19	OR: 1.00 (0.88, 1.13)	2
Nelson 2010 [53]	Caesarean section	Anal incontinence, feces	1	OR: 1.00 (0.49, 2.05)	NA	12	OR: 0.91 (0.72, 1.16)	0/0.00	OR: 0.92 (0.74, 1.16) (0.72, 1.19)	0/0.00	10.0	N	0.81	OR: 0.92 (0.74, 1.16)	3
Nelson 2010 [53]	Caesarean section	Anal incontinence, flatus	1	OR: 0.83 (0.51, 1.36)	NA	4	OR: 1.02 (0.87, 1.20)	0/0.00	OR: 1.00 (0.86, 1.16) (0.78, 1.28)	0/0.00	9.7	N	0.44	OR: 1.00 (0.86, 1.16)	3
Nieuwenhuijse 2014 [54]	Ceramic-on-ceramic bearings for total hip arthroplasty	Harris Hip Score	7	MD: −0.23 (−1.09, 0.63)	24/0.31	3	MD: −0.50 (−2.09, 1.09)	62/1.08	MD: −0.29 (−0.96, 0.38) (−1.81, 1.22)	32/0.31	59.3	N	0.77	MD: −0.20 (−0.66, 0.26)	2
Nieuwenhuijse 2014 [54]	High-flexion total knee arthroplasty	Flexion (degrees)	20	MD: 1.68 (0.28, 3.08)	45/3.83	26	MD: 3.78 (1.64, 5.92)	78/19.12	MD: 2.91 (1.56, 4.27) (-4.42, 10.25)	73/12.7	46.8	N	0.11	MD: 2.49 (1.84, 3.14)	2
Nieuwenhuijse 2014 [54]	Gender-specific total knee arthroplasty	Flexion-extension range (degrees)	6	MD: 1.40 (−0.18, 2.99)	5/0.23	2	MD: 3.15 (−0.03, 6.34)	29/1.58	MD: 1.80 (0.40, 3.21) (−0.53, 4.14)	9/0.40	74.4	Y	0.33	MD: 1.85 (0.54, 3.16)	2
Nikooie 2019 [55]	Second-generation antipsychotics	Sedation	6	RR: 1.26 (0.92, 1.72)	0/0.00	3	RR: 1.84 (0.40, 8.54)	34/0.84	RR: 1.29 (0.95, 1.74) (0.91, 1.83)	0/0.00	94.0	N	0.63	RR: 1.29 (0.95, 1.74)	2
Nikooie 2019 [55]	Second-generation antipsychotics	Neurologic outcomes	6	RR: 0.45 (0.20, 1.01)	0/0.00	5	RR: 0.76 (0.59, 0.99)	0/0.00	RR: 0.73 (0.57, 0.93) (0.56, 0.95)	0/0.00	9.0	Y	0.22	RR: 0.73 (0.57, 0.93)	2
Ochen 2019 [56]	Surgery for Achilles tendon rupture	Re-rupture	10	RR: 0.40 (0.24, 0.69)	0/0.00	18	RR: 0.42 (0.28, 0.65)	30/0.19	RR: 0.43 (0.31, 0.60) (0.20, 0.96)	21/0.12	30.4	N	0.90	RR: 0.65 (0.54, 0.79)	2
Ochen 2019 [56]	Surgery for Achilles tendon rupture	Complications	9	RR: 3.13 (1.33, 7.38)	68/1.04	15	RR: 2.93 (2.28, 3.75)	0/0.00	RR: 2.72 (1.84, 4.02) (0.84, 8.82)	41/0.28	42.2	N	0.88	RR: 2.63 (2.13, 3.27)	2
Pittas 2010 [57]	High vitamin D	Hypertension	1	RR: 1.01 (0.96, 1.06)	NA	3	RR: 0.57 (0.41, 0.79)	0/0.00	RR: 0.68 (0.43, 1.07) (0.10, 4.51)	77/0.14	38.2	N	0.0006	RR: 1.00 (0.95, 1.05)	3
Raman 2013 [58]	Carotid endarterectomy	Ipsilateral stroke	3	RR: 0.72 (0.58, 0.90)	0/0.00	2	RR: 0.47 (0.05, 4.46)	83/2.19	RR: 0.70 (0.51, 0.97) (0.29, 1.69)	38/0.05	88.1	N	0.71	RR: 0.72 (0.58, 0.89)	2
Raman 2013 [58]	Carotid endarterectomy	Any stroke	3	RR: 0.68 (0.56, 0.82)	18/0.01	3	RR: 0.73 (0.43, 1.22)	0/0.00	RR: 0.67 (0.57, 0.79) (0.53, 0.84)	0/0.00	90.3	N	0.79	RR: 0.67 (0.57, 0.79)	2
Raman 2013 [58]	Carotid artery stenting	Periprocedural stroke	2	RR: 1.75 (0.87, 3.52)	0/0.00	5	RR: 1.91 (1.72, 2.11)	7/0.00	RR: 1.91 (1.74, 2.10) (1.69, 2.16)	0/0.00	1.8	Y	0.81	RR: 1.91 (1.74, 2.10)	2
Schweizer 2013 [59]	Nasal deconolization	Surgical site infection	5	RR: 0.63 (0.36, 1.12)	49/0.20	6	RR: 0.40 (0.28, 0.57)	0/0.00	RR: 0.48 (0.33, 0.69) (0.18, 1.26)	44/0.15	47.6	Y	0.19	RR: 0.54 (0.42, 0.69)	2
Schweizer 2013 [59]	Glycopeptide prophylaxis	Surgical site infection	8	RR: 1.13 (0.90, 1.42)	0/0.00	7	RR: 0.35 (0.12, 1.03)	80/1.44	RR: 0.71 (0.48, 1.05) (0.22, 2.27)	62/0.25	61.5	N	0.04	RR: 1.04 (0.66, 1.24)	2
Silvain 2012 [60]	Enoxaparin	Mortality	6	RR: 0.88 (0.70, 1.10)	0/0.00	7	RR: 0.50 (0.40, 0.62)	0/0.00	RR: 0.64 (0.49, 0.82) (0.32, 1.26)	46/0.08	51.1	Y	0.0004	RR: 0.66 (0.56, 0.77)	2
Silvain 2012 [60]	Enoxaparin	Major bleeding	9	RR: 0.88 (0.62, 1.24)	53/0.12	7	RR: 0.72 (0.56, 0.93)	0/0.00	RR: 0.81 (0.66, 1.00) (0.49, 1.37)	30/0.05	57.5	N	0.37	RR: 0.84 (0.72, 0.98)	2
Silvain 2012 [60]	Enoxaparin	Death or myocardial infarction	13	RR: 0.86 (0.74, 0.99)	21/0.01	7	RR: 0.44 (0.35, 0.55)	0/0.00	RR: 0.67 (0.55, 0.81) (0.37, 1.21)	58/0.07	65.7	N	<0.00001	RR: 0.77 (0.71, 0.85)	2
Suthar 2012 [61]	Antiretroviral therapy	Tuberculosis infection	2	HR: 0.50 (0.34, 0.75)	0/0.00	9	HR: 0.32 (0.25, 0.41)	27/0.03	HR: 0.35 (0.29, 0.44) (0.22, 0.57)	26/0.03	21.1	N	0.07	HR: 0.37 (0.31, 0.44)	3
Te Morenga 2013 [62]	High sugar intake	Weight gain (kg)	10	MD: 0.74 (0.30, 1.19)	82/0.34	4	MD: 0.31 (−0.07, 0.68)	99/0.14	MD: 0.51 (0.26, 0.75) (−0.36, 1.37)	99/0.14	58.0	N	0.14	MD: 0.59 (0.58, 0.60)	2
Te Morenga 2013 [62]	High sugar intake	BMI (kg/m^2^)	3	MD: −0.06 (−0.15, 0.04)	0/0.00	4	MD: −0.02 (−0.05, 0.00)	74/0.00	MD: −0.02 (−0.05, −0.00) (−0.05, 0.09)	58/0.00	5.0	N	0.42	MD: −0.01 (−0.03, −0.00)	2
Thomas 2010 [63]	Influenza vaccines	Influenza-like illness	3	RR: 0.71 (0.55, 0.90)	45/0.02	1	RR: 0.31 (0.26, 0.36)	NA	RR: 0.53 (0.31, 0.89) (0.08, 3.48)	94/0.28	75.5	N	<0.00001	RR: 0.48 (0.43, 0.53)	3
Tickell-Painter 2017 [64]	Mefloquine	Discontinuation due to adverse effects	3	RR: 2.86 (1.53, 5.31)	0/0.00	9	RR: 2.73 (1.84, 4.06)	31/0.11	RR: 2.78 (2.05, 3.77) (1.57, 4.91)	15/0.04	20.8	N	0.91	RR: 2.85 (2.19, 3.71)	2
Tickell-Painter 2017 [64]	Mefloquine	Serious adverse events or effects	3	RR: 0.68 (0.11, 4.27)	0/0.00	2	RR: 3.09 (0.38, 24.95)	0/0.00	RR: 1.31 (0.33, 5.23) (0.14, 12.39)	0/0.00	56.3	N	0.29	RR: 1.31 (0.33, 5.23)	3
Tickell-Painter 2017 [64]	Mefloquine	Nausea	2	RR: 1.34 (1.04, 1.71)	0/0.00	3	RR: 1.86 (1.42, 2.42)	0/0.00	RR: 1.56 (1.30, 1.87) (1.16, 2.09)	0/0.00	53.7	N	0.08	RR: 1.56 (1.30, 1.87)	3
Tricco 2018 [65]	Live-attenuated zoster vaccines	Suspected Herpes Zoster	5	RR: 0.60 (0.54, 0.66)	0/0.00	3	RR: 0.48 (0.27, 0.83)	99/0.24	RR: 0.55 (0.40, 0.77) (0.20, 1.52)	97/0.14	43.4	N	0.44	RR: 0.72 (0.70, 0.74)	2
Vinceti 2018 [66]	Selenium	Any cancer	5	RR: 0.99 (0.86, 1.14)	46/0.01	7	RR: 0.72 (0.55, 0.93)	46/0.06	RR: 0.86 (0.73, 1.01) (0.52, 1.42)	64/0.04	54.3	N	0.03	RR: 0.94 (0.88, 1.01)	3
Vinceti 2018 [66]	Selenium	Cancer mortality	2	RR: 0.81 (0.49, 1.32)	79/0.10	7	RR: 0.76 (0.59, 0.97)	66/0.07	RR: 0.78 (0.64, 0.95) (0.44, 1.39)	65/0.05	26.8	Y	0.83	RR: 0.88 (0.80, 0.96)	3
Vinceti 2018 [66]	Selenium	Colorectal cancer	3	RR: 0.74 (0.41, 1.33)	48/0.13	6	RR: 0.82 (0.72, 0.94)	0/0.00	RR: 0.83 (0.74, 0.94) (0.73, 0.95)	0/0.00	14.4	Y	0.72	RR: 0.83 (0.74, 0.94)	3
Wilson 2011 [67]	Training for traditional birth attendants/assistance by traditional birth attendants	Perinatal mortality	5	RR: 0.77 (0.66, 0.89)	62/0.02	1	RR: 0.82 (0.38, 1.78)	NA	RR: 0.77 (0.67, 0.89) (0.53, 1.13)	52/0.01	97.0	N	0.87	RR: 0.79 (0.73, 0.86)	3
Wilson 2011 [67]	Training for traditional birth attendants/assistance by traditional birth attendants	Neonatal mortality	6	RR: 0.80 (0.71, 0.90)	37/0.01	2	RR: 0.80 (0.47, 1.37)	0/0.00	RR: 0.80 (0.73, 0.88) (0.67, 0.95)	14.0/0.00	97.0	N	0.99	RR: 0.80 (0.74, 0.87)	3
Wilson 2019 [68]	Unilateral knee arthroplasty	Venous thromboembolism	2	RR: 0.24 (0.04, 1.37)	0/0.00	8	RR: 0.42 (0.30, 0.57)	24/0.04	RR: 0.43 (0.33, 0.55) (0.29, 0.64)	8/0.01	1.9	Y	0.53	RR: 0.45 (0.37, 0.54)	2
Wilson 2019 [68]	Unilateral knee arthroplasty	Range of movement (degrees)	3	MD: −4.58 (−10.75, 1.59)	95/27.67	11	MD: −8.43 (−10.15, −6.71)	86/6.20	MD: −7.60 (−9.27, −5.93) (−13.98, −1.22)	91/7.85	22.2	Y	0.24	MD: −8.29 (−8.63, −7.95)	2
Wilson 2019 [68]	Unilateral knee arthroplasty	Operation duration (minutes)	3	MD: −1.72 (−11.89, 8.45)	90/71.69	8	MD: −23.80 (−40.43, −7.17)	99/491.19	MD: −17.07 (−29.11, −5.04) (−63.37, 29.23)	98/365.45	30.2	Y	<0.00001	MD: −11.25 (−12.71, −9.97)	2
Yank 2011 [69]	Recombinant factor VII	Mortality	2	RR: 1.40 (0.49, 4.02)	0/0.00	2	RR: 0.91 (0.39, 2.12)	0/0.00	RR: 1.08 (0.56, 2.09) (0.25, 4.59)	0/0.00	39.4	N	0.53	RR: 1.08 (0.56, 2.09)	2
Yank 2011 [69]	Recombinant factor VII	Thromboembolic events	2	RR: 2.04 (0.51, 8.20)	8/0.08	2	RR: 1.81 (0.67, 4.87)	0/0.00	RR: 1.88 (0.85, 4.16) (0.33, 10.76)	0/0.00	35.5	N	0.89	RR: 1.88 (0.85, 4.16)	2
Zhang 2016 [70]	Everolimus-eluting bioresorbable vascular scaffold	Stent thrombosis	5	OR: 1.97 (0.90, 4.29)	0/0.00	3	OR: 2.22 (1.00, 4.93)	0/0.00	OR: 2.09 (1.20, 3.64) (1.04, 4.18)	0/0.00	51.1	Y	0.83	OR: 2.09 (1.20, 3.64)	2
Zhang 2016 [70]	Everolimus-eluting bioresorbable vascular scaffold	Mortality	5	OR: 0.71 (0.17, 3.01)	45/1.16	4	OR: 0.63 (0.24, 1.63)	0/0.00	OR: 0.73 (0.34, 1.57) (0.18, 2.97)	15/0.20	51.7	N	0.89	OR: 0.82 (0.42, 1.60)	2
Zhang 2016 [70]	Everolimus-eluting bioresorbable vascular scaffold	Cardiac death	3	OR: 1.39 (0.17, 11.14)	44/1.48	4	OR: 0.94 (0.43, 2.06)	0/0.00	OR: 1.05 (0.53, 2.12) (0.42, 2.63)	0/0.00	21.4	N	0.73	OR: 1.05 (0.53, 2.12)	2
Zhang 2017 [71]	Percutaneous coronary intervention	Mortality	5	HR: 1.00 (0.79, 1.26)	22/0.02	17	HR: 1.07 (0.92, 1.26)	37/0.03	HR: 1.05 (0.93, 1.20) (0.73, 1.52)	32/0.03	25.6	N	0.59	HR: 1.08 (0.98, 1.19)	2
Zhang 2017 [71]	Percutaneous coronary intervention	Cardiovascular mortality	4	HR: 0.99 (0.71, 1.39)	21/0.02	5	HR: 1.08 (0.51, 2.28)	78/0.49	HR: 1.05 (0.69, 1.59) (0.29, 3.81)	72/0.25	51.2	N	0.85	HR: 1.33 (1.09, 1.62)	2
Zhang 2017 [71]	Percutaneous coronary intervention	Myocardial infarction	5	HR: 1.39 (0.86, 2.26)	57/0.16	5	HR: 2.00 (1.65, 2.44)	0/0.00	HR: 1.69 (1.22, 2.33) (0.71, 4.03)	57/0.12	53.7	Y	0.17	HR: 1.66 (1.42, 1.94)	2
Ziff 2015 [72]	Digoxin	Mortality	7	RR: 0.99 (0.93, 1.05)	0/0.00	8	RR: 1.60 (1.31, 1.96)	63/0.05	RR: 1.38 (1.15, 1.66) (0.77, 2.49)	75/0.06	30.2	Y	<0.00001	RR: 1.08 (1.03, 1.14)	3
Ziff 2015 [72]	Digoxin	Cardiovascular mortality	5	RR: 1.01 (0.94, 1.09)	0/0.00	3	RR: 2.53 (1.12, 5.70)	96/0.48	RR: 1.71 (1.04, 2.80) (0.26, 11.38)	96/0.29	41.9	Y	0.03	RR: 1.15 (1.08, 1.22)	3
Ziff 2015 [72]	Digoxin	Hospital admission	2	RR: 0.96 (0.87, 1.05)	65/0.00	4	RR: 0.92 (0.85, 0.99)	64/0.00	RR: 0.93 (0.88, 0.98) (0.80, 1.09)	61/0.00	37.8	Y	0.49	RR: 0.92 (0.89, 0.95)	2

*PI/ECO similarity degree: 1=more or less identical; 2=similar but not identical; 3=broadly similar

*BCG* Bacillus Calmette-Guérin, *BMI* Body mass index, *BoE* Bodies of evidence, *CE* Common effects, *CI* Confidence interval, *CRC* Colorectal cancer, *CS* Cohort studies, *DDP 4* Dipeptidylpeptidase-4, *HIV* Human immunodeficiency virus, *HR* Hazard ratio, *MD* Mean difference, *N* No, *NA* Not applicable, *NSTE-ACS* Non-ST-segment elevation acute coronary syndromes, *OR* Odds ratio, *PI/ECO* Population–intervention/exposure–comparator–outcome, *RCT* Randomized controlled trial, *RE* Random effects, *RR* Risk ratio, *SGLT-2* Sodium-dependent glucose transporter 2, *Y* Yes

### Pooling scenarios

By pooling BoE from RCTs and cohort studies with a random effects model, for 61 (51.7%) out of 118 BoE-pairs, the 95% CI excludes no effect. For the common effects model, for 77 (62.3%) out of 118 BoE-pairs, the 95% CI excludes no effect. Approximately half of the binary effect sizes were in the range of 0.75 to 1.25, and 64.2% reported an effect estimate <1. The test for subgroup difference comparing BoE from RCTs and BoE of cohort studies was statistically significant (*p*<0.05) for 25 BoE-pairs (21.2%). By pooling BoE from RCTs and cohort studies, the median *I*^2^ was 51% (*τ*^2^=0.05), whereas the mean *I*^2^ was 45% (*τ*^2^=0.11). The contributed weight of RCTs to the pooled estimates was 40% (median) and 42% (mean). As for the 95% PIs, 21.2% (*n*=25) of the pooled BoE from RCTs and cohort studies excluded no effect.

The direction of effect between BoE from RCTs and pooled effect estimates was mainly concordant in 94 of 118 BoE-pairs (79.7%). The difference between effect estimates was >0.25 for 4.2% (*n*=5) and >0.50 for 3.4% (*n*=4) for the dichotomous effect measures. The integration of BoE from cohort studies modified the conclusion from BoE of RCTs in 32 (27.1%) of the 118 BoE-pairs (i.e., 95% CI excludes no effect changed to 95% CI overlaps no effect or vice versa); in 28 (87.5%) of these 32 BoE, the direction of effect was concordant. In nine (28.1%) of these 32 BoE-pairs, the test of subgroup difference was statistically significant (*p*<0.05) comparing BoE from RCTs and BoE from cohort studies (in three of these nine associations, the direction of effect was opposite). In 12 (37.5 %) of these 32 BoE-pairs, the overall degree of PI/ECO similarity was judged as “broadly similar.” Populations (*n*=7, 21.9%), interventions (*n*=5, 15.6%), and comparators (*n*=4, 12.5%) rated as “broadly similar” accounted for PI/ECO dissimilarities overall. In 20 (62.5%) of the 32 BoE-pairs, the degree of PI/ECO similarity was judged as “similar but not identical.” Populations (*n*=18; 56.3%), interventions (*n*=11; 34.4%), comparators (*n*=7; 21.9%), and outcomes (*n*=1; 3.1%) rated as “similar but not identical” accounted for PI/ECO dissimilarities.

In the additional analysis with cohort studies as reference (Additional file 1: Table S5), the direction of effect between BoE from cohort studies and pooled estimates was concordant in 106 (89.8%) of the 118 BoE-pairs. The integration of BoE from RCTs modified the conclusion from BoE of cohort studies in 24 (20.3%) of the 118 BoE-pairs.

## Discussion

### Summary of findings

This meta-epidemiological study is the first empirical study in medical research that evaluates the impact scenario of pooling bodies of evidence from RCTs and cohort studies. Overall, 118 BoE-pairs based on 653 RCTs and 804 cohort studies were included. By pooling BoE from RCTs and cohort studies in about 50% of the BoE-pairs, the 95% CI excludes no effect, whereas in about one-third of the included BoE from RCTs, the 95% CI excludes no effect. For 21% of pooled estimates, the test for subgroup difference comparing BoE from RCTs and BoE of cohort studies was statistically significant. The median weights of BoE from RCTs to the pooled estimates were 40%, suggesting that on average the contribution weight was not dissimilar between both BoE. Overall, the degree of statistical heterogeneity was moderate (*I*^2^=51%, *τ*^2^=0.05) and higher across meta-analyses of cohort studies (*I*^2^=41%, *τ*^2^=0.03) compared to meta-analyses of RCTs (*I*^2^=5%, *τ*^2^=0.00). The integration of BoE from cohort studies modified the conclusion derived from BoE of RCTs in nearly 30% of the BoE-pairs. The direction of effect between BoE of RCTs and pooled estimates, however, was mainly concordant. This suggests that by adding evidence from cohort studies statistical precision increased substantially.

### Comparison with other studies

We did not identify any similar empirical study using a pooling scenario of different study designs in the field of medical research. However, a recent methodological study investigated a similar pooling scenario in nutrition research [77]. This large pooling scenario study showed that the integration of BoE from cohort studies modified the conclusion from BoE of RCTs in nearly 50% of included diet-disease associations, although the direction of effect was mainly concordant between BoE of RCTs and pooled estimates. The median weight of RCTs to the pooled estimates was 34%, and the statistical heterogeneity was substantially higher across meta-analyses of cohort studies (*I*^2^=55%, *τ*^2^=0.01) compared to RCTs (*I*^2^=0%, *τ*^2^=0). This finding is in line with our study. However, in our study, the integration of BoE from cohort studies modified the conclusion from BoE of RCTs less often (27% vs. 44%) [77]. Two main reasons may explain this difference. First, it has been suggested that effect estimates between RCTs and cohort studies differ quite often in nutrition research [78]. A recent meta-epidemiological study, however, has shown that on average the effect-difference between both study designs was even smaller than expected [79]. Second, the median weight of RCTs to the pooled estimated was larger in our study (40% vs. 34%) [79].

A recent meta-research study investigated how RCTs and observational studies were combined in meta-analyses [80]. In nearly 40% of meta-analyses, both observational studies and RCTs were combined in a single meta-analysis, without considering the two designs as subgroups. When comparing the results of those meta-analyses with meta-analyses restricted to RCTs only, the conclusion was modified by the integration of observational studies for nearly 71%, whereas in our study this was the case for 27%. In line with our findings, the authors found that including observational studies frequently increased statistical heterogeneity.

### Implications for the broader research field

In a survey investigating the rationale, perceptions, and preferences for the integration of RCTs and observational studies in evidence syntheses by Cuello-Garcia and colleagues [81], it was shown that conducting separate meta-analyses for both study designs was the most frequent approach used. However, nearly half of the experts interviewed reported that they have already, on at least one occasion, pooled RCTs and observational studies in a meta-analysis [81].

According to the recent GRADE guidance on optimizing the integration of RCT and observational studies in evidence syntheses, observational studies can provide valuable information as complementary, sequential, or replacement evidence for RCTs [6]. In our empirical scenario, evidence from cohort studies was always considered as complementary evidence for RCTs. The GRADE guidance suggests, when RCTs provide already high certainty of evidence, looking for observational evidence will be unnecessary because the high certainty will not be improved [6]. However, in our sample of 118 BoE-pairs, only six BoE of RCTs were rated as high certainty, 18 as moderate, 11 as low, and two as very low. Thus, evidence from cohort studies seems valuable in the field of medical research [7].

In line with our findings, the *Cochrane Handbook* indicated that authors should expect greater statistical heterogeneity in a systematic review of observational studies compared to a systematic review of RCTs. Reasons include diverse ways in which observational studies may be designed to investigate the effects of interventions/exposures, and partly due to the increased potential for methodological variation between primary studies and the resulting variation in their risk of bias. Therefore, the *Cochrane Handbook* recommends that RCTs and observational studies should not be combined in a meta-analysis (although the power to detect an effect may increase [82]). In contrast to the recommendations of Cochrane, a recent framework for the synthesis of observational studies and RCTs does not reject the pooling of both study designs in principle. It presents recommendations on when and how to combine evidence from different study designs, but also highlights challenges in this process [83]. Moreover, a recent scoping review summarized the methods to systematically review and meta-analyze observational studies and highlighted that existing guidance is highly conflicting for pooling if results are similar over different study designs [84]. Finally, in several high-impact factor journal meta-analyses, both study designs were pooled [21, 32, 36, 56].

Overall, it looks like further methodological research is needed to shed light into this gray area. On the one hand, further research should address the application of existing guidance in terms of utility, acceptability, and reproducibility and elaborate ways to deal with occurring challenges [83]. On the other hand, factors such as risk of bias/study quality that may contribute to the differences in effect estimates between BoE of RCTs and cohort studies and conflicting results in pooling scenarios should be further explored. Our previously conducted study analyzed disagreement of effect estimates with regard to differences by each PI/ECO domain [7]. In the meta-regression, we showed that differences of interventions were the main drivers towards disagreement. The average effect on the other pooled effect estimators, however, was not statistically significant [7].

We assume that methodological trial characteristics are other possible drivers towards disagreement, since observational studies are prone to risk of bias by confounding [5], and appropriate adjustment for confounding is thus crucial to integrate both RCTs and cohort studies (or other non-randomized studies) in a pooling scenario. In the sample provided in this study, the tools used to assess the quality/risk of bias of primary studies included across the BoE were heterogeneous, which makes the comparison of results challenging. Future studies should focus on the impact of quality characteristics on pooling scenarios by using similar appraisal tools to increase comparability between RCTs and cohort studies (e.g., ROBINS-I [85] and the Cochrane Risk of Bias Tool [86]). Moreover, attention in future studies should also be paid to the integration of other non-randomized study designs a part from cohort studies. However, overall, we assume generalizability of our findings since concordance may not be linked to study design per se, but rather on the quality/risk of bias of the studies included [1].

This paper did not aim to provide insights on how pooling results from different study designs impacts the certainty rating of results and whether it reduces or increases the amount of low or very low certainty of evidence ratings. In a recently published hypothetical scenario analysis, we could show that pooling BoE from RCTs and cohort studies for nutrition-related research questions would reduce the amount of very low and low certainty of evidence ratings [87]. We recommend that future research should examine also the impact scenario of pooling BoE of RCTs and cohort studies for medical research questions on the overall GRADE rating and on individual GRADE domains in order to inform future guidance development.

### Strengths and limitations

This study has several strengths. First, we analyzed a large sample of BoE-pairs (*n*=118), which was based on 653 RCTs and 804 cohort studies. Second, we selected BoE-pairs from systematic reviews published in high-impact medical journals, which have shown to be of higher methodological quality [88]. Third, our study was based on a broad methodological repertoire, i.e., by including meta-analyses of binary outcomes, and also continuous outcomes, investigating different statistical measures of heterogeneity, conducting random and common effects models, and calculating 95% PI.

Limitations of this study are as follows. First, although we pooled a large sample of BoE-pairs, our sample may not be representative of all meta-analyses, and the totality of evidence of available associations might provide different results. Second, we did not consider and weighted risk of bias of primary studies in our pooling scenario. Third, only two BoE-pairs were judged as “more or less identical,” indicating that BoE of RCTs and cohort studies differ at least slightly in terms of PI/ECO criteria and caution is therefore required when pooling both BoE. Fourth, the potential for confounding in the individual cohort studies and subgroup analyses in the meta-analysis cannot be ruled out. Moreover, several subgroups also included only a small number of studies. Fifth, the methodological quality of the systematic reviews included in this study was not assessed. Although we assume that systematic reviews published in high-impact factor journals adhere to high methodological standards, this is nevertheless an important limitation. Due to these limitations, our findings need to be interpreted with caution.

## Conclusions

This large pooling scenario study showed that the integration of BoE from cohort studies modified the conclusion from BoE of RCTs in 27% of included BoE, although the direction of effect was mainly concordant between BoE of RCTs and pooled estimates. The median weight of RCTs to the pooled estimates was 40%, and the statistical heterogeneity was substantially driven by integrating BoE of cohort studies. Our findings provide a first insight regarding the potential impact of pooling of both BoE in evidence syntheses. A decision for or against pooling different study designs should also always take into account, for example, PI/ECO similarity, risk of bias, coherence of effect estimates, and also the trustworthiness of the evidence. Overall, there is a need for more research on the influence of those issues on potential pooling.

## Supplementary Information

**Additional file 1: Appendix S1.** Search strategy. **Tables S1-S5. Table S1.** Explanation and definition for PI/ECO similarity degree. **Table S2.** PI/ECO similarity degree. **Table S3.** Differences between published (reported) effect estimates and re-calculated effect estimates**. Table S4.** Reason for exclusion from the pooling scenario. **Table S5.** Pooling results. **Figures S1-S118. Fig S1.** Forest plot: Low sodium (Intervention/Exposure); All-cause mortality (Outcome). **Fig. S2.** Forest plot: Low sodium; Cardiovascular disease. **Fig. S3.** Forest plot: Intra-aortic balloon pump; All-cause mortality. **Fig. S4.** Forest plot: Self-administered therapy; Treatment success. **Fig S5.** Forest plot: Self-administered therapy; Treatment completion. **Fig. S6.** Forest plot: Self-administered therapy; All-cause mortality. **Fig S7.** Forest plot: Antiretroviral therapy; HIV infection. **FigS8**-Forest plot: Nonnutritive sweeteners; Body Mass Index random sequence. **Fig. S9.** Forest plot: Surgical abortion by mid-level providers; Failure or incomplete abortion. **Fig. S10.** Forest plot: Surgical abortion by mid-level providers; Complications. **Fig. S11.** Forest plot: Surgical abortion by mid-level providers; Abortion failure and complications. **Fig. S12.** Forest plot: Clopidogrel pretreatment for percutaneous coronary intervention; All-cause mortality. **Fig. S13.** Forest plot: Clopidogrel pretreatment for percutaneous coronary intervention; Major bleeding. **Fig. S14.** Forest plot: Clopidogrel pretreatment for percutaneous coronary intervention; Coronary heart disease. **Fig. S15.** Forest plot: P2Y12 inhibitor pretreatment in non-ST elevation acute coronary syndrome; All-cause mortality. **Fig. S16.** Forest plot: P2Y12 inhibitor pretreatment in non-ST elevation acute coronary syndrome; Major bleeding. **Fig. S17.** Forest plot: P2Y12 inhibitor pretreatment in non-ST elevation acute coronary syndrome; Main composite ischemic endpoint. **Fig. S18.** Forest plot: Mediterranean diet; Breast cancer. **Fig. S19.** Forest plot: High calcium; All fractures. **Fig. S20.** Forest plot: High calcium; Verterbral fractures. **Fig. S21.** Forest plot: High calcium; Hip fracture. **Fig. S22.** Forest plot: Sigmoidoscopy; Colorectal cancer mortality. **Fig. S23.** Forest plot: Sigmoidoscopy; Colorectal cancer incidence. **Fig. S24.** Forest plot: High omega-3; Cerebrovascular disease. **Fig. S25.** Forest plot: High α-linolenic acid; Coronary heart disease. **Fig. S26.** Forest plot: High omega-3; Coronary heart disease. **Fig. S27.** Forest plot: Omega-6; Coronary heart disease. **Fig. S28.** Forest plot: High calcium; Cardiovascular mortality. **Fig. S29.** Forest plot: High dairy; Systolic blood pressure. **Fig. S30.** Forest plot: Radiation therapy; Erectile dysfunction. **Fig. S31.** Forest plot: Radical prostatectomy; Urinary incontinence. **Fig. S32.** Forest plot: Radical Prostatectomy; Erectile dysfunction. **Fig. S33.** Forest plot: Disease-modifying drugs; Conversion to clinically definite multiple sclerosis. **Fig. S34.** Forest plot: Extracranial-intracranial arterial bypass; All-cause mortality. **Fig. S35.** Forest plot: Extracranial-intracranial arterial bypass; Stroke. **Fig. S36.** Forest plot: Extracranial-intracranial arterial bypass; Stroke mortality or dependency. **Fig. S37.** Forest plot: Transcatheter aortic valve implantation; Early all-cause mortality. **Fig. S38.** Forest plot: Transcatheter aortic valve implantation; Mid-term all-cause mortality. **Fig. S39.** Forest plot: Transcatheter aortic valve implantation; Long-term all-cause mortality. **Fig. S40.** Forest plot: Treating gestational diabetes mellitus; High birth weight. **Fig. S41.** Forest plot: Treating gestational diabetes mellitus; Large-for-gestational age neonate. **Fig. S42.** Forest plot: Treating gestational diabetes mellitus; Shoulder dystocia. **Fig. S43.** Forest plot: Treating asymptomatic bacteriuria; Pyelonephritis. **Fig. S44.** Forest plot: Bacillus Calmette-Guérin vaccination; All-cause mortality. **Fig. S45.** Forest plot: Measles containing vaccines; All-cause mortality. **Fig. S46.** Forest plot: Total hip arthroplasty; Reoperation. **Fig. S47.** Forest plot: Total hip arthroplasty; Dislocation. **Fig. S48.** Forest plot: Total hip arthroplasty; Deep infection. **Fig. S49.** Forest plot: Chest-compression-only cardiopulmonary resuscitation; Survival. **Fig. S50**. Forest plot: Non-calcium-based phosphat binders; All-cause mortality. **Fig. S51.** Forest plot: Parenteral influenza vaccine; Influenza-like illness. **Fig. S52.** Forest plot: Parenteral influenza vaccine Influenza. **FigS53**-Forest plot: Inactivated influenza vaccines; Influenza. **Fig. S54.** Forest plot: Inactivated influenza vaccines; Influenza-like illness. **Fig. S55.** Forest plot: High total flavonoids; Colorectal neoplasms. **Fig. S56.** Forest plot: Transfusion; All-cause mortality. **Fig. S57.** Forest plot: Caesarean section; Urinary incontinence. **Fig. S58.** Forest plot: Caesarean section; Fecal incontinence. **Fig. S59.** Forest plot: Antiretroviral therapy by nurses; All-cause mortality. **Fig. S60.** Forest plot: Antiretroviral therapy by nurses; Attrition. **Fig. S61.** Forest plot: Nurses for maintenance of antiretroviral therapy; All-cause mortality. **Fig. S62.** Forest plot: Exenatide; Acute pancreatitis. **Fig. S63.** Forest plot: DDP-4 inhibitors; Heart failure. **Fig. S64.** Forest plot: DDP-4 inhibitors; Hospital admission for heart failure. **Fig. S65.** Forest plot: Tamoxifen; Heart failure. **Fig. S66.** Forest plot: SGLT-2 inhibitors; Acute kidney injury. **Fig. S67.** Forest plot: Erythropoiesis stimulating agents; Venous thromboembolism. **Fig. S68.** Forest plot: Erythropoiesis stimulating agents; All-cause mortality. **Fig. S69.** Forest plot: Pneumococcal polysaccharide vaccines; Invasive pneumococcal disease. **Fig. S70.** Forest plot: Neoral (Cyclosporin); Acute rejection of kidney transplant. **Fig. S71.** Forest plot: Early intervention for NSTE-ACS; All-cause mortality. **Fig. S72.** Forest plot: Early intervention for NSTE-ACS; Myocardial infarction. **Fig. S73.** Forest plot: Early intervention for NSTE-ACS; Major bleeding. **Fig. S74.** Forest plot: Caesarean section; Anal incontinence; feces. **Fig. S75.** Forest plot: Caesarean section; Anal incontinence; flatus. **Fig. S76.** Forest plot: Ceramic-on-ceramic bearings for total hip arthroplasty; Harris Hip Score. **Fig. S77.** Forest plot: High-flexion total knee arthroplasty; Flexion in degrees. **Fig. S78.** Forest plot: Gender-specific total knee arthroplasty; Flexion-extension range. **Fig. S79.** Forest plot: Second generation antipsychotics; Sedation. **Fig. S80.** Forest plot: Second generation antipsychotics; Neurologic outcomes. **Fig. S81**. Forest plot: Surgery for achilles tendon rupture; Re-rupture. **Fig. S82.** Forest plot: Surgery for achilles tendon rupture; Complications. **Fig. S83.** Forest plot: High vitamin D; Hypertension. **Fig. S84.** Forest plot: Carotid endarterectomy; Ipsilateral stroke. **FigS85**-Forest plot: Carotid endarterectomy; Stroke. **Fig. S86.** Forest plot: Carotid artery stenting; Periprocedural stroke. **Fig. S87.** Forest plot: Nasal deconolization; Surgical site infection. **Fig. S88.** Forest plot: Glycopeptide prophylaxis; Surgical site infection. **Fig. S89.** Forest plot: Enoxaparin; All-cause mortality. **Fig. S90.** Forest plot: Enoxaparin; Major bleeding. **Fig. S91.** Forest plot: Enoxaparin; All-cause mortality or myocardial infarction. **Fig. S92.** Forest plot: Antiretroviral therapy; Tuberculosis infection. **Fig. S93.** Forest plot: High sugar intake; Weight gain. **Fig. S94.** Forest plot: High sugar intake; Body Mass Index. **Fig. S95.** Forest plot: Influenza vaccines; Influenza-like illness. **Fig. S96.** Forest plot: Mefloquine; Discontinuation due to adverse effects. **Fig. S97.** Forest plot: Mefloquine; Serious adverse events or effects. **Fig. S98.** Forest plot: Mefloquine; Nausea. **Fig. S99.** Forest plot: Live-attenuated zoster vaccines; Suspected Herpes Zoster. **Fig. S100.** Forest plot: High selenium; Cancer. **Fig. S101.** Forest plot: High selenium; Cancer mortality. **FigS102**-Forest plot: High selenium; Colorectal cancer. **FigS103**-Forest plot: Training for traditional birth attendants/ assistance by traditional birth attendants; Perinatal mortality. **Fig. S104.** Forest plot: Training for traditional birth attendants/ assistance by traditional birth attendants; Neonatal mortality. **Fig. S105.** Forest plot: Unicompartimental knee arthroplasty; Venous thromboembolism. **Fig. S106.** Forest plot: Unicompartimental knee arthroplasty; Flexion-extension range. **Fig. S107.** Forest plot: Unicompartimental knee arthroplasty; Operation duration. **Fig. S108.** Forest plot: Recombinant factor VII; All-cause mortality. **Fig. S109.** Forest plot: Recombinant factor VII; Thromboembolism. **Fig. S110.** Forest plot: Everolimus-eluting bioresorbable vascular scaffold; Stent thrombosis. **Fig. S111.** Forest plot: Everolimus-eluting bioresorbable vascular scaffold; All-cause mortality. **Fig. S112.** Forest plot: Everolimus-eluting bioresorbable vascular scaffold; Coronary heart disease mortality. **Fig. S113.** Forest plot: Percutaneous coronary intervention; All-cause mortality. **Fig. S114.** Forest plot: Percutaneous coronary intervention; Cardiovascular mortality. **Fig. S115.** Forest plot: Percutaneous coronary intervention; Myocardial infarction. **Fig. S116.** Forest plot: Digoxin; All-cause mortality. **Fig. S117.** Forest plot: Digoxin; Cardiovascular mortality. **Fig. S118.** Forest plot: Digoxin; Hospital admission.

## Data Availability

Data are based on published meta-analyses.

## Abbreviations

BoE: Bodies of evidence
CI: Confidence interval
GRADE: Grading of Recommendations, Assessment, Development and Evaluation
PI: Prediction interval
PI/ECO: Patient/population, intervention/exposure, comparator, outcome
RCT: Randomized controlled trial
*τ*^2^: Heterogeneity value with the restricted maximum-likelihood estimation method
