# Supplementary material for: An empirical evaluation of the impact scenario of pooling bodies of evidence from randomized controlled trials and cohort studies in medical research
Source: BMC Med. 2022 Oct 24;20:355. doi: 10.1186/s12916-022-02559-y (PMC9590141; doi:10.1186/s12916-022-02559-y)
Supplement: Supplementary file 1 — Additional file 1: Appendix S1. Search strategy. Tables S1-S5. Table S1. Explanation and definition for PI/ECO similarity degree. Table S2. PI/ECO similarity degree. Table S3. Differences between published (reported) effect estimates and re-calculated effect estimates. Table S4. Reason for exclusion from the pooling scenario. Table S5. Pooling results. Figures S1-S118. Fig S1. Forest plot: Low sodium (Intervention/Exposure); All-cause mortality (Outcome). Fig. S2. Forest plot: Low sodium; Cardiovascular disease. Fig. S3. Forest plot: Intra-aortic balloon pump; All-cause mortality. Fig. S4. Forest plot: Self-administered therapy; Treatment success. Fig S5. Forest plot: Self-administered therapy; Treatment completion. Fig. S6. Forest plot: Self-administered therapy; All-cause mortality. Fig S7. Forest plot: Antiretroviral therapy; HIV infection. FigS8-Forest plot: Nonnutritive sweeteners; Body Mass Index random sequence. Fig. S9. Forest plot: Surgical abortion by mid-level providers; Failure or incomplete abortion. Fig. S10. Forest plot: Surgical abortion by mid-level providers; Complications. Fig. S11. Forest plot: Surgical abortion by mid-level providers; Abortion failure and complications. Fig. S12. Forest plot: Clopidogrel pretreatment for percutaneous coronary intervention; All-cause mortality. Fig. S13. Forest plot: Clopidogrel pretreatment for percutaneous coronary intervention; Major bleeding. Fig. S14. Forest plot: Clopidogrel pretreatment for percutaneous coronary intervention; Coronary heart disease. Fig. S15. Forest plot: P2Y12 inhibitor pretreatment in non-ST elevation acute coronary syndrome; All-cause mortality. Fig. S16. Forest plot: P2Y12 inhibitor pretreatment in non-ST elevation acute coronary syndrome; Major bleeding. Fig. S17. Forest plot: P2Y12 inhibitor pretreatment in non-ST elevation acute coronary syndrome; Main composite ischemic endpoint. Fig. S18. Forest plot: Mediterranean diet; Breast cancer. Fig. S19. Forest plot: High calcium; All frac [file 12916_2022_2559_MOESM1_ESM.pdf]

ADDITIONAL FILE 1

An empirical evaluation of the impact scenario of pooling bodies of evidence from randomized controlled trials and cohort studies in medical research

Nils Bröckelmann[1*], Julia Stadelmaier[1*], Louisa Harms[1], Charlotte Kubiak[1], Jessica Beyerbach[1], Martin Wolkewitz[2], Jörg J Meerpohl[1,3], Lukas Schwingshackl[1]

[1] Institute for Evidence in Medicine, Medical Center - University of Freiburg, Faculty of Medicine, University of Freiburg, Freiburg, Germany.

[2] Institute of Medical Biometry and Statistics, Faculty of Medicine and Medical Center - University of Freiburg, Freiburg, Germany.

[3] Cochrane Germany, Cochrane Germany Foundation, Freiburg, Germany.

*contributed equally

Corresponding author: Lukas Schwingshackl, PhD

Breisacher Straße 86, 79110 Freiburg, Germany

M: schwingshackl@ifem.uni-freiburg.de

P: +49 (0)761 203-96867

ORCID number: 0000-0003-3407-7594

Twitter: https://twitter.com/LSchwingshackl

Content Supplement

Appendix S1: Search strategy for systematic reviews in MEDLINE via PubMed 3

Table S1: Explanation and definition for Population (P), Intervention/Exposure (I/E), Comparator (C), Outcome (O) similarity degree 4

Table S2: Ratings of Population (P), Intervention/Exposure (I/E), Comparator (C), Outcome (O) similarity degree for all identified body of evidence-pairs 6

Table S3: Differences between published (reported) effect estimates and re-calculated effect estimates 15

Table S4: Reason for exclusion from the pooling scenario 22

Table S5: Pooling results of bodies of evidence from cohort studies with RCTs based on random effects and common effect model, 95% prediction intervals, heterogeneity, test for subgroup difference, and Population (P), Intervention (I) /Exposure (E), Comparator (C) Outcome similarity degree. 23

Figure S1 to 118: Forest plots 33

Supplementary References 151

Appendix S1: Search strategy for systematic reviews in MEDLINE via PubMed

ID	Search
#1	"lancet london england"[Journal] OR "JAMA"[Journal] OR "bmj clinical research ed"[Journal] OR "jama internal medicine"[Journal] OR "Annals of internal medicine"[Journal] OR "PLoS medicine"[Journal] OR "BMC medicine"[Journal] OR "The Cochrane database of systematic reviews"[Journal] OR "Mayo Clinic proceedings"[Journal] OR "Canadian Medical Association journal"[Journal] OR "Nat Rev Dis Primers"[Journal] OR "J Cachexia Sarcopenia Muscle"[Journal] OR "N Engl J Med"[Journal]
#2	"systematic review"[Title/Abstract] OR "systematic literature review"[Title/Abstract] OR "systematic scoping review"[Title/Abstract] OR "systematic meta-review"[Title/Abstract] OR "systematic search"[Title/Abstract] OR "systematic review"[Publication Type] OR "meta analys*"[Title/Abstract] OR "meta analys*"[Publication Type] OR "cochrane database syst rev"[Journal]
#3	"random*"[Title/Abstract] OR "placebo"[Title/Abstract] OR "clinical trials as topic"[MeSH Terms:noexp] OR "trial"[Title]
#4	"epidemiolog*"[Title/Abstract] OR "cohort stud*"[Title/Abstract] OR "observation*"[Title/Abstract] OR "non rct*"[Title/Abstract] OR "non random*"[Title/Abstract]
#5	#1 AND #2 AND #3 AND #4
#6	#1 AND #2 AND #3 AND #4 Filters: from 2010/1/1 - 2019/12/31

Table S1: Explanation and definition for Population (P), Intervention/Exposure (I/E), Comparator (C), Outcome (O) similarity degree

Rating	Population	Intervention/Exposure	Comparator	Outcome
**1 = "more or less identical"**	Same health status and type of population	Same drug, invasive procedure, nutrition-intervention or vaccine	-Same drug or invasive procedure<br>-Nutrition: Placebo vs. Nil or low intake; Low intake vs. Low intake	Same outcome
	e.g.<br>-Both BoE with either healthy population, general population or diseased population<br>-Same age category (both adults, both postmenopausal women)	e.g.<br>- Both Enoxaparin<br>- Both PCI<br>- Both high dairy-intake	e.g.<br>-Both no Enoxaparin<br>-Both UKA<br>-Placebo vs. No intervention or low intake	e.g.<br>Mortality in both BoE
**2 = "similar but not identical"**	Populations with mixed health status in RCTs and/ or cohort studies	-Different drugs of the same class/ Any drug of the same class vs. Specific drug of the same class<br>-Similar invasive procedure/ same invasive procedure with different co-interventions<br>-Similar vaccines or identical vaccine with different route of administration<br>-Supplementary or free food vs. Intake<br>-Similar but not identical time frame of intervention	-Different drugs of the same class<br>-Similar invasive procedures, drug, vaccine or diet<br>-General dietary advice vs. High intake	-Similar outcome<br>-Both with mixed similar outcomes

	e.g.<br>-Merged healthy and diseased population in one BoE vs. healthy population in the other BoE<br>-Both BoE with merged healthy and diseased population<br>-Population with cardiovascular risk factors (without manifest disease) vs. Healthy population	e.g.<br>-Both different SGAs<br>-Both similar regional anaesthetic nerve blocks<br>-Various pneumococcal vaccines (2,3,12,14,17 and 23-valent) versus 23-valent only<br>-One or two doses of measles containing vaccines versus unclear number of doses<br>-Free non-caloric beverages vs. Low intake of SSBs<br>-Early intervention with different time frame (first 14 vs. first 24 hours)	e.g.<br>-Both different DDP-4 inhibitors<br>-"Best medical treatment" with Aspirin and additionally with various other drugs<br>-No vaccination or delayed vaccination versus no vaccination<br>-Transfemoral vs. transapical TAVI<br>-General dietary advice vs. High red meat intake	e.g.<br>-Late stage only or all CRC vs. All CRC<br>-Both with mixed sedation outcomes (e.g. Sleepiness, sedation)
**3 = "broadly similar"**	-Different health status of populations in RCTs and cohort studies<br>-Other substantial differences (e.g. Age-category, type of population)	-Same drug for different indication<br>-Enhanced treatment vs. Any treatment<br>-Supplement vs. Status<br>-Different time frame/ early treatment vs. Any treatment	-Active intervention (drug, invasive procedure, nutrient) vs. No intervention or placebo<br>-Different time frame	-Broadly similar
	e.g.<br>-Healthy population in one BoE vs. population with cardiovascular disease in the other BoE<br>-Children/adolescents vs. Adults<br>-Travellers vs. Pregnant women	e.g.<br>-Digoxin for HF vs. digoxin post-myocardial infarction without HF<br>-Dispatcher-assisted bystander CPR vs. unassisted bystander CPR<br>-Enhanced training of birth attendants vs. Any support by birth attendant<br>-Selenium supplements vs. High selenium status<br>-Early ART vs. Any ART	e.g.<br>-Restrictive transfusion vs. No transfusion<br>-Placebo vs. Low selenium status<br>-No vaccination of health care workers vs. Low share of vaccinated health care workers per facility<br>-Pregnant women with untreated bacteriuria vs. Pregnant without screening for bacteriuria<br>-Delayed ART vs. No ART	e.g.<br>-Colorectal adenoma vs. Cancer

ART: antiretroviral therapy; BoE: bodies of evidence; CRC: colorectal cancer; CPR: cardiopulmonary resuscitation; DDP-4: dipeptidyl peptidase 4; HF: heart failure; PCI: percutaneous coronary intervention; PI/ECO: population, intervention/ exposure, comparator, outcome; RCT: randomized controlled trial; SGA: second-generation antipsychotic; SSBs: sugar-sweetened beverages; TAVI: transcatheter aortic valve replacement; UKA: unicompartimental knee arthroplasty

Table S2: Ratings of Population (P), Intervention/Exposure (I/E), Comparator (C), Outcome (O) similarity degree for all identified body of evidence-pairs

Reference	**Intervention/ Exposure** (as defined by the authors)	**Intervention / Exposure type**	**Outcome** (as defined by the authors)	**Outcome-category** (e.g., cancer outcomes, cardiovascular disease, all-cause mortality)	**Rating of PI/ECO-similarity degree** Patients/ Population	Intervention/ Exposure	Comparator	Outcome	Overall	**Included in the pooling scenario (Yes/No)**
Abou-Setta 2011 (1)	Nerve block	Invasive	Delirium	Neurological	2	2	2	1	2	No
Abou-Setta 2011 (1)	Spinal anaesthesia	Invasive	All-cause mortality	All-cause mortality	2	1	1	1	2	No
Aburto 2013 (2)	Low sodium	Nutrition	All-cause mortality	All-cause mortality	2	2	2	1	2	Yes
Aburto 2013 (2)	Low sodium	Nutrition	Cardiovascular disease	Cardiovascular disease	2	2	2	2	2	Yes
Ahmad 2015 (3)	Intra-aortic balloon pump	Invasive	All-cause mortality	All-cause mortality	1	1	1	1	1	Yes
Alexander 2017 (4)	High DHA and EPA	Nutrition	Coronary heart disease	Cardiovascular disease	2	2	2	1	2	No
Alexander 2017 (4)	High DHA and EPA	Nutrition	Coronary heart disease mortality	Cardiovascular disease	2	2	2	1	2	No
Alexander 2017 (4)	High DHA and EPA	Nutrition	Coronary heart disease incidence	Cardiovascular disease	2	2	2	1	2	No
Alipanah 2018 (5)	Self-administered therapy	Drug	Treatment success	Infectiological	3	2	1	1	3	Yes
Alipanah 2018 (5)	Self-administered therapy	Drug	Treatment completion	Drug safety	3	2	1	1	3	Yes
Alipanah 2018 (5)	Self-administered therapy	Drug	All-cause mortality	All-cause mortality	3	2	1	1	3	Yes
Anglemyer 2013 (6)	Antiretroviral therapy	Drug	HIV infection	Infectiological	2	2	3	1	3	Yes
Azad 2017 (7)	Nonnutritive sweeteners	Nutrition	Body Mass Index	Metabolic	2	2	1	1	2	Yes

Barnard 2015 (8)	Surgical abortion by mid-level providers	Invasive	Failure or incomplete abortion	Obstetrical	2	2	1	1	2	Yes
Barnard 2015 (8)	Surgical abortion by mid-level providers	Invasive	Complications	Obstetrical	2	2	1	1	2	Yes
Barnard 2015 (8)	Surgical abortion by mid-level providers	Invasive	Abortion failure and complications	Obstetrical	2	2	1	1	2	Yes
Bellemain-Appaix 2012 (9)	Clopidogrel pretreatment for percutaneous coronary intervention	Drug	All-cause mortality	All-cause mortality	2	2	2	1	2	Yes
Bellemain-Appaix 2012 (9)	Clopidogrel pretreatment for percutaneous coronary intervention	Drug	Major bleeding	Drug safety	2	2	2	1	2	Yes
Bellemain-Appaix 2012 (9)	Clopidogrel pretreatment for percutaneous coronary intervention	Drug	Coronary heart disease	Cardiovascular disease	2	2	2	1	2	Yes
Bellemain-Appaix 2014 (10)	P2Y12 inhibitor pretreatment in non-ST elevation acute coronary syndrome	Drug	All-cause mortality	All-cause mortality	2	2	2	1	2	Yes
Bellemain-Appaix 2014 (10)	P2Y12 inhibitor pretreatment in non-ST elevation acute coronary syndrome	Drug	Major bleeding	Drug safety	2	2	2	1	2	Yes
Bellemain-Appaix 2014 (10)	P2Y12 inhibitor pretreatment in non-ST elevation acute coronary syndrome	Drug	Main composite ischemic endpoint	Cardiovascular disease	2	2	2	1	2	Yes
Bloomfield 2016 (11)	Mediterranean diet	Nutrition	Breast cancer	Oncological	2	2	2	1	2	Yes

Bolland 2015 (12)	High calcium	Nutrition	All fractures	Orthopaedic	1	2	1	1	2	Yes
Bolland 2015 (12)	High calcium	Nutrition	Vertebral fracture	Orthopaedic	1	2	1	1	2	Yes
Bolland 2015 (12)	High calcium	Nutrition	Hip fracture	Orthopaedic	1	2	1	1	2	Yes
Brenner 2014 (13)	Sigmoidoscopy	Invasive	Colorectal cancer mortality	Oncological	1	1	1	1	1	Yes
Brenner 2014 (13)	Sigmoidoscopy	Invasive	Colorectal cancer incidence	Oncological	1	1	1	2	2	Yes
Chowdhury 2012 (14)	High omega-3	Nutrition	Cerebrovascular disease	Cardiovascular disease	2	2	1	1	2	Yes
Chowdhury 2014a (15)	High α-linolenic acid	Nutrition	Coronary heart disease	Cardiovascular disease	3	2	2	1	3	Yes
Chowdhury 2014a (15)	High omega-3	Nutrition	Coronary heart disease	Cardiovascular disease	3	2	2	1	3	Yes
Chowdhury 2014a (15)	High omega-6	Nutrition	Coronary heart disease	Cardiovascular disease	3	2	2	1	3	Yes
Chowdhury 2014b (16)	High vitamin D	Nutrition	All-cause mortality	All-cause mortality	2	3	3	1	3	No
Chung 2011 (17)	High vitamin D	Nutrition	Colorectal cancer	Oncological	2	3	3	1	3	No
Chung 2011 (17)	High vitamin D	Nutrition	Breast cancer	Oncological	2	3	3	1	3	No
Chung 2016 (18)	High calcium	Nutrition	Cardiovascular mortality	Cardiovascular disease	2	2	1	1	2	Yes
Ding 2017 (19)	High dairy	Nutrition	Systolic blood pressure	Cardiovascular disease	2	1	1	1	2	Yes
Fenton 2018 (20)	Radiation therapy	Invasive	Erectile dysfunction	Urological	1	1	2	1	2	Yes
Fenton 2018 (20)	Radical Prostatectomy	Invasive	Urinary incontinence	Urological	1	1	2	1	2	Yes
Fenton 2018 (20)	Radical Prostatectomy	Invasive	Erectile dysfunction	Urological	1	1	2	1	2	Yes
Filippini 2017 (21)	Disease-modifying drugs	Drug	Conversion to clinically definite multiple sclerosis	Neurological	1	2	1	1	2	Yes

Fluri 2010 (22)	Extracranial-intracranial arterial bypass	Invasive	All-cause mortality	All-cause mortality	2	1	2	1	2	Yes
Fluri 2010 (22)	Extracranial-intracranial arterial bypass	Invasive	Stroke	Cardiovascular disease	2	1	2	1	2	Yes
Fluri 2010 (22)	Extracranial-intracranial arterial bypass	Invasive	Stroke mortality or dependency	Cardiovascular disease	1	1	2	1	2	Yes
Gargiulo 2016 (23)	Transcatheter aortic valve implantation	Invasive	Early all-cause mortality	All-cause mortality	2	2	1	1	2	Yes
Gargiulo 2016 (23)	Transcatheter aortic valve implantation	Invasive	Mid-term all-cause mortality	All-cause mortality	2	2	1	1	2	Yes
Gargiulo 2016 (23)	Transcatheter aortic valve implantation	Invasive	Long-term all-cause mortality	All-cause mortality	2	1	1	1	2	Yes
Hartling 2013 (24)	Treating gestational diabetes mellitus	Nutrition	High birth weight	Obstetrical	2	1	1	1	2	Yes
Hartling 2013 (24)	Treating gestational diabetes mellitus	Nutrition	Large-for-gestational age neonate	Obstetrical	2	1	1	1	2	Yes
Hartling 2013 (24)	Treating gestational diabetes mellitus	Nutrition	Shoulder dystocia	Obstetrical	2	1	1	1	2	Yes
Henderson 2019 (25)	Treating asymptomatic bacteriuria	Drug	Pyelonephritis	Infectiological	2	2	3	1	3	Yes
Higgins 2016 (26)	Bacillus Calmette-Guérin vaccination	Vaccine	All-cause mortality	All-cause mortality	3	1	2	1	3	Yes
Higgins 2016 (26)	Measles containing vaccines	Vaccine	All-cause mortality	All-cause mortality	3	2	2	1	3	Yes
Hopley 2010 (27)	Total hip arthroplasty	Invasive	Reoperation	Orthopaedic	2	1	1	1	2	Yes
Hopley 2010 (27)	Total hip arthroplasty	Invasive	Dislocation	Orthopaedic	2	1	1	1	2	Yes
Hopley 2010 (27)	Total hip arthroplasty	Invasive	Deep infection	Infectiological	2	2	1	1	2	Yes
Hüpfl 2010 (28)	Chest-compression-only cardiopulmo-	Cardiopulmonary	Survival	All-cause mortality	1	3	3	1	3	Yes

	nary resuscitation	resuscitation								
Jamal 2013 (29)	Non-calcium-based phosphate binders	Drug	All-cause mortality	All-cause mortality	2	2	1	1	2	Yes
Jefferson 2010 (30)	Parenteral influenza vaccine	Vaccine	Influenza-like illness	Infectiological	2	2	3	1	3	Yes
Jefferson 2010 (30)	Parenteral influenza vaccine	Vaccine	Influenza	Infectiological	2	2	1	1	2	Yes
Jefferson 2012 (31)	Inactivated influenza vaccines	Vaccine	Influenza	Infectiological	1	2	1	1	2	Yes
Jefferson 2012 (31)	Inactivated influenza vaccines	Vaccine	Influenza-like illness	Infectiological	1	2	1	1	2	Yes
Jin 2012 (32)	High total flavonoids	Nutrition	Colorectal neoplasms	Oncological	3	2	2	3	3	Yes
Johnston 2019 (33)	Red meat	Nutrition	All-cause mortality	All-cause mortality	2	2	2	1	2	No
Johnston 2019 (33)	Red meat	Nutrition	Cardiovascular mortality	Cardiovascular disease	2	2	2	1	2	No
Johnston 2019 (33)	Red meat	Nutrition	Cardiovascular disease	Cardiovascular disease	2	2	2	1	2	No
Kansagara 2013 (34)	Transfusion	Transfusion	All-cause mortality	All-cause mortality	2	3	3	2	3	Yes
Keag 2018 (35)	Caesarean section	Invasive	Urinary incontinence	Obstetrical	3	2	2	1	3	Yes
Keag 2018 (35)	Caesarean section	Invasive	Fecal incontinence	Obstetrical	3	2	2	1	3	Yes
Kredo 2014 (36)	Antiretroviral therapy by nurses	Drug	All-cause mortality	All-cause mortality	2	3	1	1	3	Yes
Kredo 2014 (36)	Antiretroviral therapy by nurses	Drug	Attrition	Drug safety	2	3	1	1	3	Yes
Kredo 2014 (36)	Nurses for maintenance of antiretroviral therapy	Drug	All-cause mortality	All-cause mortality	2	3	2	1	3	Yes
Li 2014 (37)	Exenatide	Drug	Acute pancreatitis	Drug safety	2	1	2	2	2	Yes
Li 2016 (38)	DDP-4 inhibitors	Drug	Heart failure	Drug safety	2	2	2	1	2	Yes

Li 2016 (38)	DDP-4 inhibitors	Drug	Hospital admission for heart failure	Drug safety	2	2	2	1	2	Yes
Matthews 2018 (39)	Tamoxifen	Drug	Heart failure	Drug safety	2	3	1	1	3	Yes
Menne 2019 (40)	SGLT-2 inhibitors	Drug	Acute kidney injury	Drug safety	2	2	2	1	2	Yes
Mesgarpour 2017 (41)	Erythropoiesis stimulating agents	Drug	Venous thrombo-embolism	Drug safety	2	2	2	1	2	Yes
Mesgarpour 2017 (41)	Erythropoiesis stimulating agents	Drug	All-cause mortality	All-cause mortality	2	2	2	1	2	Yes
Moberley 2013 (42)	Pneumococcal polysaccharide vaccines	Vaccine	Invasive pneumococcal disease	Infectiological	2	2	1	1	2	Yes
Molnar 2015 (43)	Neoral (Cyclosporin)	Drug	Acute rejection of kidney transplant	Drug safety	2	1	2	1	2	Yes
Navarese 2013 (44)	Early intervention for NSTE-ACS	Invasive	All-cause mortality	All-cause mortality	2	2	1	1	2	Yes
Navarese 2013 (44)	Early intervention for NSTE-ACS	Invasive	Myocardial infarction	Cardiovascular disease	2	2	1	1	2	Yes
Navarese 2013 (44)	Early intervention for NSTE-ACS	Invasive	Major bleeding	Drug safety	2	2	1	1	2	Yes
Nelson 2010 (45)	Caesarean section	Invasive	Anal incontinence, feces	Obstetrical	3	2	2	1	3	Yes
Nelson 2010 (45)	Caesarean section	Invasive	Anal incontinence, flatus	Obstetrical	3	2	2	1	3	Yes
Nieuwenhuijse 2014 (46)	Ceramic-on-ceramic bearings for total hip arthroplasty	Invasive	Harris Hip Score	Orthopaedic	2	1	1	1	2	Yes
Nieuwenhuijse 2014 (46)	High-flexion total knee arthroplasty	Invasive	Flexion	Orthopaedic	2	1	1	1	2	Yes
Nieuwenhuijse 2014 (46)	Gender-specific total knee arthroplasty	Invasive	Flexion-extension range	Orthopaedic	2	1	1	1	2	Yes
Nikooie 2019 (47)	Second generation antipsychotics	Drug	Sedation	Drug safety	2	2	1	2	2	Yes

Nikooie 2019 (47)	Second generation antipsychotics	Drug	Neurologic outcomes	Drug safety	2	2	1	2	2	Yes
Ochen 2019 (48)	Surgery for achilles tendon rupture	Invasive	Re-rupture	Orthopaedic	1	2	2	1	2	Yes
Ochen 2019 (48)	Surgery for achilles tendon rupture	Invasive	Complications	Orthopaedic	1	2	2	1	2	Yes
Pittas 2010 (49)	High vitamin D	Nutrition	Hypertension	Cardiovascular disease	2	3	3	1	3	Yes
Raman 2013 (50)	Carotid endarterectomy	Invasive	Ipsilateral stroke	Cardiovascular disease	2	1	2	1	2	Yes
Raman 2013 (50)	Carotid endarterectomy	Invasive	Stroke	Cardiovascular disease	2	1	2	1	2	Yes
Raman 2013 (50)	Carotid artery stenting	Invasive	Periprocedural stroke	Cardiovascular disease	2	2	2	1	2	Yes
Schweizer 2013 (51)	Nasal deconolization	Drug	Surgical site infection	Infectiological	2	2	1	1	2	Yes
Schweizer 2013 (51)	Glycopeptide prophylaxis	Drug	Surgical site infection	Infectiological	2	2	2	1	2	Yes
Silvain 2012 (52)	Enoxaparin	Drug	All-cause mortality	All-cause mortality	2	1	2	1	2	Yes
Silvain 2012 (52)	Enoxaparin	Drug	Major bleeding	Drug safety	2	1	2	1	2	Yes
Silvain 2012 (52)	Enoxaparin	Drug	All-cause mortality or myo-cardial infarction	Cardiovascular disease	2	1	2	1	2	Yes
Suthar 2012 (53)	Antiretroviral therapy	Drug	Tuberculosis infection	Infectiological	2	3	3	1	3	Yes
Te Morenga 2013 (54)	High sugar intake	Nutrition	Weight gain	Metabolic	2	1	2	1	2	Yes
Te Morenga 2013 (54)	High sugar intake	Nutrition	Body Mass Index	Metabolic	2	2	2	1	2	Yes
Thomas 2010 (55)	Influenza vaccines	Vaccine	Influenza-like illness	Infectiological	2	3	3	1	3	Yes
Tickell-Painter 2017 (56)	Mefloquine	Drug	Discontinuation due to adverse effects	Drug safety	2	1	1	1	2	Yes

Tickell-Painter 2017 (56)	Mefloquine	Drug	Serious adverse events or effects	Drug safety	3	1	1	2	3	Yes
Tickell-Painter 2017 (56)	Mefloquine	Drug	Nausea	Drug safety	3	1	1	1	3	Yes
Tricco 2018 (57)	Live-attenuated zoster vaccines	Vaccine	Suspected Herpes Zoster	Infectiological	2	2	2	1	2	Yes
Vinceti 2018 (58)	High selenium	Nutrition	Cancer	Oncological	2	3	3	2	3	Yes
Vinceti 2018 (58)	High selenium	Nutrition	Cancer mortality	Oncological	2	3	3	1	3	Yes
Vinceti 2018 (58)	High selenium	Nutrition	Colorectal cancer	Oncological	2	3	3	1	3	Yes
Wilson 2011 (59)	Training for traditional birth attendants/ assistance by traditional birth attendants	Birth assistance	Perinatal mortality	All-cause mortality	1	2	3	1	3	Yes
Wilson 2011 (59)	Training for traditional birth attendants/ assistance by traditional birth attendants	Birth assistance	Neonatal mortality	All-cause mortality	1	2	3	1	3	Yes
Wilson 2019 (60)	Unicompartimental knee arthroplasty	Invasive	Venous thrombo-embolism	Orthopaedic	2	1	1	1	2	Yes
Wilson 2019 (60)	Unicompartimental knee arthroplasty	Invasive	Flexion-extension range	Orthopaedic	2	1	1	1	2	Yes
Wilson 2019 (60)	Unicompartimental knee arthroplasty	Invasive	Operation duration	Orthopaedic	2	1	1	1	2	Yes
Yank 2011 (61)	Recombinant factor VII	Drug	All-cause mortality	All-cause mortality	2	2	1	1	2	Yes
Yank 2011 (61)	Recombinant factor VII	Drug	Thrombo-embolism	Drug safety	2	2	1	1	2	Yes
Zhang 2016 (62)	Everolimus-eluting bioresorbable vascular scaffold	Invasive	Stent thrombosis	Cardiovascular disease	2	1	1	1	2	Yes

Zhang 2016 (62)	Everolimus-eluting bioresorbable vascular scaffold	Invasive	All-cause mortality	All-cause mortality	2	1	1	1	2	Yes
Zhang 2016 (62)	Everolimus-eluting bioresorbable vascular scaffold	Invasive	Coronary heart disease mortality	Cardiovascular disease	2	1	1	1	2	Yes
Zhang 2017 (63)	Percutaneous coronary intervention	Invasive	All-cause mortality	All-cause mortality	2	2	1	1	2	Yes
Zhang 2017 (63)	Percutaneous coronary intervention	Invasive	Cardiovascular mortality	Cardiovascular disease	2	2	1	1	2	Yes
Zhang 2017 (63)	Percutaneous coronary intervention	Invasive	Myocardial infarction	Cardiovascular disease	2	2	1	1	2	Yes
Ziff 2015 (64)	Digoxin	Drug	All-cause mortality	All-cause mortality	3	1	1	1	3	Yes
Ziff 2015 (64)	Digoxin	Drug	Cardiovascular mortality	Cardiovascular disease	3	1	1	1	3	Yes
Ziff 2015 (64)	Digoxin	Drug	Hospital admission	Drug safety	2	1	1	1	2	Yes

DDP 4: Dipeptidylpeptidase-4; DHA and EPA: Docosahexaenoic acid and eicosapentaenoic acid; HIV: human immunodeficiency virus; NSTE-ACS: Non–ST-Segment Elevation Acute Coronary Syndromes; PI/ECO: population – intervention/exposure – comparator – outcome ; SGLT-2: Sodium dependent glucose transporter 2.

Table S3: Differences between published (reported) effect estimates and re-calculated effect estimates

Reference	Intervention / Exposure (as defined by the authors)	Outcome (as defined by the authors)	RCTs		Cohort Studies	
			Reported*	Recalculated (RE and inverse-variance model)	Reported*	Recalculated (RE and inverse-variance model)
			RR/HR/OR or MD (95% CI)	RR/HR/OR or MD (95% CI)	RR/HR/OR or MD (95% CI)	RR/HR/OR or MD (95% CI)
Aburto 2013 (2)	Low sodium	Cardiovascular disease	-	-	RR: 0.89 (0.75, 1.08)	RR: 0.90 (0.75, 1.08)
Ahmad 2015 (3)	Intra-aortic balloon pump	Mortality	-	-	-	OR: 1.02 (0.67, 1.56)[1]
Alipanah 2018 (5)	Self-administered therapy	Treatment success	-	RR: 0.95 (0.87, 1.03)[1]	RR: 0.81 (0.73, 0.89)	RR: 0.81 (0.74, 0.88)
Alipanah 2018 (5)	Self-administered therapy	Treatment completion	RR: 0.79 (0.56, 1.11)	RR: 0.79 (0.57, 1.09)	RR: 1.10 (0.90, 1.35)	RR: 1.10 (0.91, 1.33)
Alipanah 2018 (5)	Self-administered therapy	Mortality	-	-	RR: 1.35 (1.00, 1.84)	RR: 1.35 (1.00, 1.83)
Anglemyer 2013 (6)	Antiretroviral therapy	HIV Infection	RR: 0.11 (0.04, 0.32)	RR: 0.11 (0.04, 0.30)	RR: 0.58 (0.35, 0.96)	RR: 0.59 (0.36, 0.97)
Barnard 2015 (8)	Surgical abortion by mid-level providers	Failure or incomplete abortion	RR: 2.97 (0.21, 41.82)	RR: 2.84 (0.24, 32.97)	-	RR: 2.47 (1.44, 4.23)[1]
Barnard 2015 (8)	Surgical abortion by mid-level providers	Complications	RR: 0.99 (0.17, 5.7)	RR: 0.94 (0.14, 6.44)	-	RR: 1.30 (0.57, 2.96)[1]
Barnard 2015 (8)	Surgical abortion by mid-level providers	Abortion failure and complications	RR: 3.07 (0.16, 59.08)	RR: 2.93 (0.19, 44.15)	-	RR: 1.33 (0.78, 2.27)[1]
Bellemain-Appaix 2012 (9)	Clopidogrel pretreatment for percutaneous coronary intervention	All-cause mortality	OR: 0.80 (0.57, 1.11)	OR: 0.80 (0.58, 1.10)	-	OR: 0.79 (0.53, 1.18)[2]
Bellemain-Appaix 2012 (9)	Clopidogrel pretreatment for percutaneous coronary intervention	Major bleeding	-	-	-	OR: 1.03 (0.69, 1.53)[2]

Bellemain-Appaix 2012 (9)	Clopidogrel pretreatment for percutaneous coronary intervention	Coronary heart disease	-	-	-	OR: 0.76 (0.60, 0.95)[2]
Bellemain-Appaix 2014 (10)	P2Y12 inhibitor pretreatment in non-ST elevation acute coronary syndrome	All-cause mortality	OR: 0.92 (0.43, 1.98)	OR: 0.90 (0.71, 1.14)	-	OR: 0.69 (0.35, 1.32)[2]
Bellemain-Appaix 2014 (10)	P2Y12 inhibitor pretreatment in non-ST elevation acute coronary syndrome	Major bleeding	OR: 1.45 (0.97, 2.15)	OR: 1.43 (1.16, 1.76)	-	OR: 1.13 (0.92, 1.39)[2]
Bellemain-Appaix 2014 (10)	P2Y12 inhibitor pretreatment in non-ST elevation acute coronary syndrome	Main composite ischemic endpoint	OR: 0.85 (0.67, 1.07)	OR: 0.87 (0.73, 1.04)	-	OR: 0.78 (0.56, 1.08)
Bolland 2015 (12)	High-calcium	All fractures	RR: 0.90 (0.83, 0.96)	RR: 0.90 (0.83, 0.97)	-	RR: 1.02 (0.93, 1.12)
Bolland 2015 (12)	High-calcium	Vertebral fracture	-	-	RR: 1.40 (1.10, 1.90)	RR: 1.40 (1.10, 1.78)
Bolland 2015 (12)	High-calcium	Hip fracture	-	-	-	RR: 1.09 (0.91, 1.30)[3]
Brenner 2014 (13)	Sigmoidoscopy	Colorectal cancer mortality	-	-	RR: 0.59 (0.45, 0.76)	RR: 0.59 (0.45, 0.77)
Brenner 2014 (13)	Sigmoidoscopy	Colorectal cancer incidence	RR: 0.82 (0.75, 0.89)	RR: 0.82 (0.75, 0.90)	-	RR: 0.50 (0.37, 0.67)[1]
Chowdhury 2012 (14)	High omega-3	Cerebrovascular disease	RR: 0.98 (0.89, 1.08)	RR: 0.99 (0.90, 1.08)	RR: 0.90 (0.80, 1.01)	RR: 0.89 (0.89, 0.99)
Chowdhury 2014a (15)	High α-linolenic acid	Coronary heart disease	-	-	RR: 0.99 (0.86, 1.14)	RR: 0.99 (0.87, 1.13)
Chung 2016 (18)	High calcium	Cardiovascular mortality	-	RR: 1.05 (0.82, 1.33)[3]		RR: 0.97 (0.86, 1.09)

Fenton 2018 (20)	Radiation therapy	Erectile dysfunction	-	-	-	RR: 1.30 (1.19, 1.43)[1]
Fenton 2018 (20)	Radical Prostatectomy	Urinary incontinence	RR: 2.27 (1.82, 2.84)	RR: 2.25 (1.80, 2.82)	-	RR: 2.91 (1.80, 4.71)[1]
Fenton 2018 (20)	Radical Prostatectomy	Erectile dysfunction	RR: 1.60 (1.23, 2.07)	RR: 1.60 (1.24, 2.07)	-	RR: 1.49 (1.33, 1.66)[1]
Filippini 2017 (21)	Disease-modifying drugs	Conversion to clinically definite multiple sclerosis	-	HR: 0.52 (0.46, 0.60)[4]	-	HR: 0.48 (0.30, 0.78)[4]
Fluri 2010 (22)	Extracranial-intracranial arterial bypass	Mortality	-	-	-	OR: 0.97 (0.58, 1.62)[1,7]
Fluri 2010 (22)	Extracranial-intracranial arterial bypass	Any stroke	-	OR: 0.44 (0.06, 3.24)[7]	-	OR: 0.76 (0.49, 1.17)[1,7]
Fluri 2010 (22)	Extracranial-intracranial arterial bypass	Death or dependency	-	-	OR: 0.80 (0.50, 1.29)[7]	OR: 0.81 (0.50, 1.31)[7]
Gargiulo 2016 (23)	Transcatheter aortic valve implantation	Early mortality	OR: 0.80 (0.51, 1.25)	OR: 0.80 (0.58, 1.11)	-	OR: 1.08 (0.84, 1.39)[1]
Gargiulo 2016 (23)	Transcatheter aortic valve implantation	Mid-term mortality	OR: 0.90 (0.64, 1.26)	OR: 0.90 (0.71, 1.13)	-	-
Gargiulo 2016 (23)	Transcatheter aortic valve implantation	Long-term mortality	OR: 1.03 (0.65, 1.62)	OR: 1.03 (0.77, 1.37)	OR: 1.70 (1.23, 2.35)	OR: 1.70 (1.31, 2.20)
Hartling 2013 (24)	Treating Gestational Diabetes Mellitus	Birth weight > 4000g	-	-	-	RR: 0.69 (0.31, 1.54)[3]
Hartling 2013 (24)	Treating Gestational Diabetes Mellitus	Shoulder dystocia	-	-	RR: 0.38 (0.19, 0.78)	RR: 0.38 (0.18, 0.80)
Henderson 2019 (25)	Treating asymptomatic bacteriuria	Pyelonephritis	-	-	-	RR: 0.29 (0.15, 0.57)[3]
Higgins 2016 (26)	Bacillus Calmette-Guérin vaccination	Mortality	-	RR: 0.67 (0.40, 1.14)[1]	-	-
Higgins 2016 (26)	Measles containing vaccines	Mortality	-	-	-	RR: 0.53 (0.40, 0.70)[1]
Hopley 2010 (27)	Total hip arthroplasty	Reoperation	-	-	-	RR: 0.45 (0.19, 1.08)[1]

Hopley 2010 (27)	Total hip arthroplasty	Dislocation	-	-	-	RR: 0.79 (0.27, 2.35)[1]
Jamal 2013 (29)	Non-calcium-based phosphate binders	Mortality	RR: 0.78 (0.61, 0.98)	RR: 0.78 (0.62, 0.98)	-	-
Jefferson 2010 (30)	Parenteral influenza vaccine	Influenza-like illness	-	-	-	RR: 0.76 (0.66, 0.87)[3]
Jefferson 2010 (30)	Parenteral influenza vaccine	Influenza	-	-	-	RR: 0.51 (0.27, 0.97)[3]
Jefferson 2012 (31)	Inactivated influenza vaccines	Influenza	-	-	-	RR: 0.20 (0.10, 0.39)[1]
Jefferson 2012 (31)	Inactivated influenza vaccines	Influenza-like illness	-	-	-	RR: 0.29 (0.07, 1.15)[1]
Kansagara 2013 (34)	Transfusion	Mortality	RR: 0.94 (0.61, 1.42)	RR: 0.94 (0.62, 1.43)	-	RR: 2.49 (1.40, 4.43)[1]
Keag 2018 (35)	Caesarean section	Urinary incontinence	-	-	OR: 0.56 (0.47, 0.66)	OR: 0.56 (0.48, 0.66)
Keag 2018 (35)	Caesarean section	Fecal incontinence	OR: 3.07 (0.90, 10.49)	OR: 3.07 (0.90, 10.47)	-	-
Li 2014 (37)	Exenatide	Acute pancreatitis/ Admission for acute pancreatitis	RR: 0.86 (0.22, 3.37)	RR: 0.86 (0.22, 3.39)	-	RR: 0.92 (0.69, 1.22)[3]
Li 2016 (38)	DDP-4 Inhibitors	Heart failure	RR: 0.90 (0.61, 1.35)	RR: 0.95 (0.60, 1.50)	-	RR: 1.10 (1.04, 1.17)[3]
Matthews 2018 (39)	Tamoxifen	Heart failure	RR: 0.52 (0.33, 0.71)	RR: 0.52 (0.33, 0.79)	RR: 0.84 (0.65, 1.07)	RR: 0.85 (0.66, 1.09)
Menne 2019 (40)	SGLT-2 inhibitors	Acute kidney injury	-	-	OR: 0.40 (0.33, 0.48)	OR: 0.40 (0.31, 0.52)
Mesgarpour 2017 (41)	Erythropoesis stimulating agents	Venous thromboembolism	-	-	RR: 1.87 (0.59, 5.92)	RR: 1.92 (0.64, 5.76)
Mesgarpour 2017 (41)	Erythropoesis stimulating agents	Mortality	RR: 0.81 (0.71, 0.93)	RR: 0.82 (0.71, 0.93)	RR: 1.07 (0.65, 1.77)	RR: 1.08 (0.66, 1.78)
Molnar 2015 (43)	Neoral (cyclosporine)	Acute rejection of kidney transplant	OR: 1.23 (0.64, 2.36)	OR: 1.25 (0.61, 2.56)	-	OR: 0.46 (0.25, 0.86)[1]

Navarese 2013 (44)	Early intervention for NSTE-ACS	Myocardial infarction	OR: 1.15 (0.65, 2.01)	OR: 1.16 (0.67, 2.00)	-	-
Nelson 2010 (45)	Caesarean section	Anal incontinence, feces	-	-	-	OR: 0.91 (0.72, 1.16)[1]
Nelson 2010 (45)	Caesarean section	Anal incontinence, flatus	-	-	-	OR: 1.02 (0.87, 1.20)[1]
Nieuwenhuijse 2014 (46)	Ceramic-on-ceramic bearings for total hip arthroplasty	Harris Hip Score	-	MD: -0.23 (-1.09, 0.63)[4]	-	MD: -0.50 (-2.09, 1.09)[1,4]
Nieuwenhuijse 2014 (46)	High-flexion total knee arthroplasty	Flexion (degrees)	-	MD: 1.68 (0.28, 3.08)[4]	-	MD: 3.78 (1.64, 5.92)[1,4]
Nieuwenhuijse 2014 (46)	Gender-specific total knee arthroplasty	Flexion-extension range (degrees)	-	MD: 1.40 (-0.18, 2.99)[1,4]	-	MD: 3.15 (-0.03, 6.34)[1,4]
Nikooie 2019 (47)	Second generation antipsychotics	Sedation	-	-	-	RR: 1.84 (0.40, 8.54)[3]
Nikooie 2019 (47)	Second generation antipsychotics	Neurologic outcomes	-	-	-	RR: 0.76 (0.59, 0.99)[3]
Ochen 2019 (48)	Surgery for achilles tendon rupture	Re-rupture	-	-	RR: 0.42 (0.28, 0.64)	RR: 0.42 (0.28, 0.65)
Ochen 2019 (48)	Surgery for achilles tendon rupture	Complications	RR: 3.26 (1.26, 8.41)	RR: 3.13 (1.33, 7.38)	-	-
Raman 2013 (50)	Carotid endarterectomy	Ipsilateral stroke	-	-	-	RR: 0.47 (0.05, 4.46)[1]
Raman 2013 (50)	Carotid endarterectomy	Any stroke	-	-	-	RR: 0.73 (0.43, 1.22)[1]
Raman 2013 (50)	Carotid artery stenting	Periprocedural stroke	-	RR: 1.75 (0.87, 3.52)[3]	-	RR: 1.91 (1.72, 2.11)[1]
Schweizer 2013 (51)	Nasal deconolization	Surgical site infection	RR: 0.63 (0.63, 1.13)	RR: 0.63 (0.36, 1.12)	-	RR: 0.40 (0.28, 0.57)[1]
Schweizer 2013 (51)	Glycopeptide prophylaxis	Surgical site infection	-	-	RR: 0.34 (0.11, 1.10)	RR: 0.35 (0.12, 1.03)
Silvain 2012 (52)	Enoxaparin	Mortality	-	RR: 0.88 (0.70, 1.10)[4]		RR: 0.50 (0.40, 0.62)[1,4]

Silvain 2012 (52)	Enoxaparin	Major bleeding	-	RR: 0.88 (0.62, 1.24)[4]		RR: 0.72 (0.56, 0.93) [1,4]
Silvain 2012 (52)	Enoxaparin	Death or Myocardial infarction	-	RR: 0.86 (0.74, 0.99)[4]		RR: 0.44 (0.35, 0.55) [1,4]
Suthar 2012 (53)	Antiretroviral therapy	Tuberculosis infection	-	HR: 0.50 (0.34, 0.75)[1]	-	-
Te Morenga 2013 (54)	High sugar intake	Weight gain (kg)	MD: 0.75 (0.30, 1.19)	MD: 0.74 (0.30, 1.19)	-	-
Te Morenga 2013 (54)	High sugar intake	Body Mass Index (kg/m^2)	-	MD: -0.06 (-0.15, 0.04)[1]	-	-
Tickell-Painter 2017 (56)	Mefloquine	Discontinuation due to adverse effects	-	-	RR: 2.73 (1.83, 4.08)	RR: 2.73 (1.84, 4.06)
Tickell-Painter 2017 (56)	Mefloquine	Serious adverse events or effects	RR: 0.70 (0.14, 3.53)	RR: 0.68 (0.11, 4.27)	RR: 3.08 (0.39, 24.11)	RR: 3.09 (0.38, 24.95)
Tickell-Painter 2017 (56)	Mefloquine	Nausea	RR: 1.35 (1.05, 1.73)	RR: 1.34 (1.04, 1.71)	RR: 1.85 (1.42, 2.43)	RR: 1.86 (1.42, 2.42)
Tricco 2018 (57)	Live-attenuated zoster vaccines	Suspected Herpes Zoster	RR: 0.61 (0.48, 0.93)	RR: 0.60 (0.54, 0.66)	RR: 0.48 (0.27, 0.84)	RR: 0.48 (0.27, 0.83)[5]
Wilson 2011 (59)	Training for traditional birth attendants/ assistance by traditional birth attendants	Perinatal mortality	RR: 0.76 (0.64, 0.88)	RR: 0.77 (0.66, 0.89)	-	RR: 0.82 (0.38, 1.78)[1]
Wilson 2011 (59)	Training for traditional birth attendants/ assistance by traditional birth attendants	Neonatal mortality	RR: 0.79 (0.69, 0.88)	RR: 0.80 (0.71, 0.90)	-	RR: 0.80 (0.47, 1.37)[1]
Wilson 2019 (60)	Unilateral knee arthroplasty	Venous thromboembolism	-	-	-	RR: 0.42 (0.30, 0.57)[1]
Wilson 2019 (60)	Unilateral knee arthroplasty	Range of movement (degrees)	-	-	-	MD: -8.43 (-10.15, -6.71)[1]
Wilson 2019 (60)	Unilateral knee arthroplasty	Operation duration (minutes)	-	-	-	MD: -23.80 (-40.43, -1.17)[1]

Yank 2011 (61)	Recombinant factor VII	Mortality	-	RR: 1.40 (0.49, 4.02)[4,6]	-	RR: 0.91 (0.39, 2.12)[4,6]
Yank 2011 (61)	Recombinant factor VII	Thromboembolic events	-	RR: 2.04 (0.51, 8.84)[4,6]	-	RR: 1.81 (0.67, 4.87)[4,6]
Zhang 2016 (62)	Everolimus-eluting bioresorbable vascular scaffold	Stent thrombosis	OR: 2.05 (0.95, 4.43)[7]	OR: 1.97 (0.90, 4.29)	OR: 2.32 (1.06, 5.07)[7]	OR: 2.22 (1.00, 4.93)
Zhang 2016 (62)	Everolimus-eluting bioresorbable vascular scaffold	Mortality	OR: 0.96 (0.46, 2.00)[7]	OR: 0.71 (0.17, 3.01)	OR: 0.57 (0.23, 1.44)[7]	OR: 0.63 (0.24, 1.63)
Zhang 2016 (62)	Everolimus-eluting bioresorbable vascular scaffold	Cardiac death	OR: 1.40 (0.45, 4.33)[7]	OR: 1.39 (0.17, 11.14)	OR: 0.81 (0.38, 1.70)[7]	OR: 0.94 (0.43, 2.06)
Zhang 2017 (63)	Percutaneous coronary intervention	Mortality	-	-	HR: 1.08 (0.92, 1.26)	HR: 1.07 (0.92, 1.26)
Zhang 2017 (63)	Percutaneous coronary intervention	Cardiovascular mortality	HR: 1.00 (0.72, 1.39)	HR: 0.99 (0.71, 1.39)	HR: 1.08 (0.51, 2.29)	HR: 1.08 (0.51, 2.28)
Zhang 2017 (63)	Percutaneous coronary intervention	Myocardial infarction	HR: 1.39 (0.85, 2.27)	HR: 1.39 (0.86, 2.26)	HR: 2.01 (1.64, 2.45)	HR: 2.00 (1.65, 2.44)
Ziff 2015 (64)	Digoxin	Mortality	-	-	RR: 1.61 (1.31, 1.97)	RR: 1.60 (1.31, 1.96)
Ziff 2015 (64)	Digoxin	Cardiovascular mortality	RR: 1.01 (0.94, 1.08)	RR: 1.01 (0.94, 1.09)	RR: 2.53 (1.12, 5.71)	RR: 2.53 (1.12, 5.70)
Ziff 2015 (64)	Digoxin	Hospital admission	RR: 0.94 (0.90, 0.99)	RR: 0.96 (0.87, 1.05)	RR: 0.91 (0.87, 0.95)	RR: 0.92 (0.85, 0.99)

CI: confidence interval; DDP 4: Dipeptidylpeptidase-4; HIV: human immunodeficiency virus; HR: hazard ratio; MD: mean difference; NSTE-ACS: Non–ST-Segment Elevation Acute Coronary Syndromes; OR: odds ratio; RCT: randomized controlled trial; RE: random effects; RR: risk ratio; SGLT-2: Sodium dependent glucose transporter 2. *some estimates were converted (detailed description is reported elsewhere (65)).

[1] Only re-calculated data is shown since we excluded some primary studies from the original estimate due to inappropriate study design.

[2] Pooled estimate includes observational analysis of randomized controlled trials.

[3] Primary studies were not pooled in the original paper.

[4] In the original paper cohort studies and randomized controlled trials were pooled together (sometimes without a subgroup).

[5] Re-analysis with unpublished data.

[6] Data converted (risk difference to risk ratio).

[7] In the original paper common effects were reported, we calculated estimates with random effects model.

Table S4: Reason for exclusion from the pooling scenario

Systematic Review	Intervention/Exposure	Outcome	Reason for exclusion
Abou-Setta 2011 (1)	Nerve block	Delirium (OR)	Forest plots not available
Abou-Setta 2011 (1)	Spinal anaesthesia	Mortality (OR)	Forest plots not available
Alexander 2017 (4)	High docosahexaenoic acid and eicosapentaenoic acid	Any coronary heart disease event (RR)	Forest plots not available
Alexander 2017 (4)	High docosahexaenoic acid and eicosapentaenoic acid	Fatal coronary heart disease events (RR)	Forest plots not available
Alexander 2017 (4)	High docosahexaenoic acid and eicosapentaenoic acid	Non-fatal coronary heart disease events (RR)	Forest plots not available
Chowdhury 2014b (16)	High vitamin D	Mortality (RR)	Forest plot not available for RCTs
Chung 2011 (17)	High vitamin D	Colorectal cancer (RR)	Forest plots not available
Chung 2011 (17)	High vitamin D	Breast cancer (RR)	Forest plots not available
Johnston 2019 (33)	Low red meat	Mortality (HR)	Forest plot not available for cohort studies
Johnston 2019 (33)	Low red meat	Cardiovascular mortality (HR)	Forest plot not available for cohort studies
Johnston 2019 (33)	Low red meat	Cardiovascular disease (HR)	Forest plot not available for cohort studies

HR: Hazard ratio; OR: Odds ratio; RCT: randomized controlled trial; RR: Risk ratio;

Table S5: Pooling results of bodies of evidence from cohort studies with RCTs based on random effects and common effect model, 95% prediction intervals, heterogeneity, test for subgroup difference, and Population (P), Intervention (I) /Exposure (E), Comparator (C) Outcome similarity degree.

Author, year, and reference	Intervention/ Exposure	Outcome	BoE CSs, n	Effect estimate (95% CI)	I^2 (%)/ tau^2	Pooled effect estimate (95%) RE (95% prediction interval)	I^2 (%)/ tau^2	Weight CS (%)	CS conclusion modified	Pooled effect estimate (95%) CE	Degree of PI/ECO similarity*
Aburto 2013 (2)	Low sodium	Mortality	7	RR: 0.94 (0.83, 1.06)	61/ 0.02	RR: 0.93 (0.83, 1.04) (0.68, 1.26)	47/ 0.02	95.0	No	RR: 0.94 (0.88, 1.00)	2
Aburto 2013 (2)	Low sodium	Cardiovascular disease	9	RR: 0.90 (0.75, 1.08)	78/ 0.07	RR: 0.89 (0.75, 1.06) (0.49, 1.62)	74/ 0.07	91.3	No	RR: 0.86 (0.80, 0.93)	2
Ahmad 2015 (3)	Intra-aortic balloon pump	Mortality	14	OR: 1.02 (0.57, 1.82)	97/ 1.03	OR: 1.02 (0.67, 1.56) (0.14, 7.32)	95/ 0.86	62.2	No	OR: 0.76 (0.72, 0.82)	1
Alipanah 2018 (5)	Self-administered therapy	Treatment success	16	RR: 0.81 (0.74, 0.88)	91/ 0.02	RR: 0.84 (0.78, 0.90) (0.62, 1.14)	89/ 0.02	80.9	No	RR: 0.92 (0.90, 0.94)	3
Alipanah 2018 (5)	Self-administered therapy	Treatment completion	14	RR: 1.10 (0.91, 1.33)	86/ 0.07	RR: 1.02 (0.84, 1.23) (0.51, 2.02)	88/0.10	75.6	No	RR: 1.12 (1.07, 1.17)	3
Alipanah 2018 (5)	Self-administered therapy	Mortality	23	RR: 1.35 (1.00, 1.83)	90/ 0.34	RR: 1.26 (0.95, 1.67) (0.37, 4.28)	88/ 0.33	90.2	No	RR: 1.26 (1.18, 1.34)	3
Anglemyer 2013 (6)	Antiretroviral therapy	HIV infection	9	RR: 0.59 (0.36, 0.97)	63/ 0.25	RR: 0.45 (0.26, 0.78) (0.09, 2.31)	75/ 0.42	88.2	No	RR: 0.72 (0.64, 0.82)	3
Azad 2017 (7)	Non-nutritive sweeteners	BMI	1	MD: 0.77 (0.47, 1.07)	NA	MD: 0.23 (-0.77, 1.23) (-3.88, 4.34)	79/ 0.65	38.6	Yes	MD: 0.53 (0.26, 0.80)	2
Barnard 2015 (8)	Surgical abortion by mid-level providers	Failure or incomplete abortion	2	RR: 2.47 (1.44, 4.23)	0/ 0.00	RR: 2.23 (1.15, 4.32) (0.24, 20.54)	33/ 0.15	65.5	No	RR: 2.14 (1.35, 3.39)	2
Barnard 2015 (8)	Surgical abortion by mid-level providers	Complications	2	RR: 1.30 (0.57, 2.96)	70/ 0.26	RR: 1.31 (0.70, 2.42) (0.17, 10.11)	32/ 0.13	90.3	No	RR: 1.51 (1.05, 2.17)	2

Barnard 2015 (8)	Surgical abortion by mid-level providers	Abortion failure and complications	3	RR: 1.33 (0.78, 2.27)	74/ 0.16	RR: 1.36 (0.83, 2.21) (0.29, 6.32)	65/ 0.17	80.5	No	RR: 1.43 (1.12, 1.82)	2
Bellemain-Appaix 2012 (9)	Clopidogrel pretreatment for percutaneous coronary intervention	Mortality	8	OR: 0.79 (0.53, 1.18)	79/ 0.23	OR: 0.77 (0.57, 1.04) (0.30, 2.02)	66/ 0.17	69.3	No	OR: 0.65 (0.57, 0.75)	2
Bellemain-Appaix 2012 (9)	Clopidogrel pretreatment for percutaneous coronary intervention	Major bleeding	8	OR: 1.03 (0.69, 1.53)	64/ 0.16	OR: 1.04 (0.81, 1.33) (0.54, 2.03)	46/ 0.08	59.6	No	OR: 1.07 (0.92, 1.24)	2
Bellemain-Appaix 2012 (9)	Clopidogrel pretreatment for percutaneous coronary intervention	Major coronary event	8	OR: 0.76 (0.60, 0.95)	82/ 0.08	OR: 0.76 (0.65, 0.89) (0.45, 1.29)	69/ 0.05	64.0	No	OR: 0.78 (0.73, 0.85)	2
Bellemain-Appaix 2014 (10)	P2Y12 inhibitor pretreatment in non-ST elevation acute coronary syndrome	Mortality	4	OR: 0.69 (0.35, 1.32)	35/ 0.17	OR: 0.90 (0.75, 1.07) (0.65, 1.24)	10/ 0.01	33.2	No	OR: 0.91 (0.80, 1.04)	2
Bellemain-Appaix 2014 (10)	P2Y12 inhibitor pretreatment in non-ST elevation acute coronary syndrome	Major bleeding	4	OR: 1.13 (0.92, 1.39)	0/ 0.00	OR: 1.27 (1.10, 1.47) (1.05, 1.54)	0/ 0.00	50.3	No	OR: 1.27 (1.10, 1.47)	2
Bellemain-Appaix 2014 (10)	P2Y12 inhibitor pretreatment in non-ST elevation acute coronary syndrome	Main composite ischemic endpoint	4	OR: 0.78 (0.56, 1.08)	65/ 0.07	OR: 0.84 (0.72, 0.98) (0.56, 1.26)	52/ 0.02	45.0	Yes	OR: 0.85 (0.78, 0.93)	2
Bloomfield 2016 (11)	Mediterranean diet	Breast cancer	13	RR: 0.96 (0.90, 1.03)	52/ 0.01	RR: 0.95 (0.89, 1.02) (0.78, 1.17)	57/ 0.01	99.1	No	RR: 0.98 (0.95, 1.02)	2
Bolland 2015 (12)	High calcium	All fractures	5	RR: 1.02 (0.93, 1.12)	68/ 0.01	RR: 0.94 (0.88, 1.00) (0.78, 1.14)	50/ 0.01	42.0	No	RR: 0.99 (0.96, 1.02)	2
Bolland 2015 (12)	High calcium	Vertebral fracture	1	RR: 1.40 (1.10, 1.78)	NA	RR: 0.94 (0.79, 1.11) (0.65, 1.34)	22/ 0.02	23.3	Yes	RR: 0.98 (0.87, 1.11)	2
Bolland 2015 (12)	High calcium	Hip fracture	6	RR: 1.09 (0.91, 1.30)	50/ 0.03	RR: 1.02 (0.89, 1.18) (0.67, 1.56)	46/ 0.04	57.2	No	RR: 0.98 (0.91, 1.07)	2

Brenner 2014 (13)	Sigmoidoscopy, screening for CRC	Colorectal cancer mortality	1	RR: 0.59 (0.45, 0.77)	NA	RR: 0.70 (0.64, 0.77) (0.60, 0.82)	0/ 0.00	12.7	No	RR: 0.70 (0.64, 0.77)	1
Brenner 2014 (13)	Sigmoidoscopy, screening for CRC	Colorectal cancer incidence	2	RR: 0.50 (0.37, 0.69)	0/ 0.00	RR: 0.78 (0.69, 0.89) (0.55, 1.11)	65/ 0.01	11.0	No	RR: 0.79 (0.74, 0.84)	2
Chowdhury 2012 (14)	High omega-3-fatty acids	Cerebrovascular disease	10	RR: 0.89 (0.80, 0.99)	17/ 0.01	RR: 0.93 (0.85, 1.01) (0.78, 1.10)	21/ 0.00	59.3	Yes	RR: 0.95 (0.89, 1.01)	2
Chowdhury 2014a (15)	High α-linolenic acid	Coronary event	7	RR: 0.99 (0.88, 1.11)	61/ 0.02	RR: 0.99 (0.88, 1.11) (0.72, 1.37)	54/ 0.02	78.6	No	RR: 1.01 (0.95, 1.08)	3
Chowdhury 2014a (15)	High omega-3-fatty acids	Coronary event	16	RR: 0.87 (0.78, 0.97)	76/ 0.03	RR: 0.90 (0.83, 0.97) (0.66, 1.22)	61/ 0.02	61.9	No	RR: 0.93 (0.89, 0.97)	3
Chowdhury 2014a (15)	High omega-6-fatty acids	Coronary event	8	RR: 0.98 (0.90, 1.06)	54/ 0.01	RR: 0.94 (0.87, 1.03) (0.73, 1.21)	56/0.01	70.0	No	RR: 0.96 (0.94, 1.01)	3
Chung 2016 (18)	High calcium	Cardiovascular disease mortality	6	RR: 0.97 (0.86, 1.09)	37/ 0.01	RR: 0.99 (0.92, 1.07) (0.86, 1.15)	11/ 0.00	89.9	No	RR: 1.01 (0.95, 1.07)	2
Ding 2017 (19)	High dairy	Systolic blood pressure	27	MD: -0.11 (-0.20, -0.02)	30/ 0.01	MD: -0.11 (-0.20, -0.03) (-0.34, 0.11)	24/ 0.01	98.8	No	MD: -0.16 (-0.21, -0.11)	2
Fenton 2018 (20)	Radiation therapy	Erectile dysfunction	7	RR: 1.30 (1.19, 1.43)	31/ 0.00	RR: 1.24 (1.09, 1.41) (0.83, 1.86)	70/ 0.02	85.7	No	RR: 1.23 (1.15, 1.32)	2
Fenton 2018 (20)	Radical Prostatectomy	Urinary incontinence	5	RR: 2.91 (1.80, 4.71)	67/ 0.18	RR: 2.54 (1.97, 3.27) (1.28, 5.03)	51/ 0.06	52.1	No	RR: 2.46 (2.08, 2.90)	2
Fenton 2018 (20)	Radical Prostatectomy	Erectile dysfunction	6	RR: 1.49 (1.33, 1.66)	63/ 0.01	RR: 1.53 (1.37, 1.70) (1.07, 2.18)	75/ 0.02	65.1	No	RR: 1.50 (1.42, 1.58)	2
Filippini 2017 (21)	Disease-modifying drugs	Conversion to clinically definite multiple sclerosis	2	HR: 0.48 (0.30, 0.78)	62/ 0.08	HR: 0.53 (0.47, 0.59) (0.46, 0.61)	0/ 0.00	70.0	No	HR: 0.53 (0.47, 0.59)	2
Fluri 2010 (22)	Extracranial-intracranial arterial bypass	Mortality	11	OR: 0.97 (0.58, 1.62)	0/ 0.00	OR: 0.84 (0.66, 1.06) (0.64, 1.09)	0/ 0.00	20.3	No	OR: 0.84 (0.66, 1.06)	2

Fluri 2010 (22)	Extracranial-intracranial arterial bypass	Any stroke	15	OR: 0.76 (0.49, 1.17)	2/ 0.02	OR: 0.77 (0.50, 1.17) (0.29, 2.05)	29/ 0.17	67.5	No	OR: 0.95 (0.78, 1.16)	2
Fluri 2010 (22)	Extracranial-intracranial arterial bypass	Death or dependency	8	OR: 0.81 (0.50, 1.31)	0/ 0.00	OR: 0.91 (0.73, 1.14) (0.70, 1.19)	0/ 0.00	20.6	No	OR: 0.91 (0.73, 1.14)	2
Gargiulo 2016 (23)	Transcatheter aortic valve implantation	Early mortality	29	OR: 1.08 (0.84, 1.39)	41/ 0.16	OR: 1.01 (0.81, 1.26) (0.47, 2.20)	39/ 0.13	81.6	No	OR: 1.02 (0.88, 1.19)	2
Gargiulo 2016 (23)	Transcatheter aortic valve implantation	Mid-term mortality	18	OR: 1.00 (0.81, 1.24)	46/ 0.08	OR: 0.96 (0.82, 1.13) (0.59, 1.58)	40/ 0.05	71.0	No	OR: 0.93 (0.83, 1.04)	2
Gargiulo 2016 (23)	Transcatheter aortic valve implantation	Long-term mortality	6	OR: 1.70 (1.31, 2.20)	0/ 0.00	OR: 1.28 (1.00, 1.65) (0.62, 2.66)	62/ 0.08	46.7	Yes	OR: 1.18 (1.03, 1.35)	2
Hartling 2013 (24)	Treating Gestational Diabetes Mellitus	Birth weight > 4000g	6	RR: 0.69 (0.31, 1.54)	88/ 0.64	RR: 0.58 (0.40, 0.86) (0.17, 2.01)	79/ 0.25	48.3	Yes	RR: 0.54 (0.46, 0.63)	2
Hartling 2013 (24)	Treating Gestational Diabetes Mellitus	Large-for-gestational age neonate	4	RR: 0.43 (0.27, 0.70)	58/ 0.13	RR: 0.47 (0.36, 0.62) (0.22, 1.02)	60/ 0.07	49.8	No	RR: 0.45 (0.39, 0.52)	2
Hartling 2013 (24)	Treating Gestational Diabetes Mellitus	Shoulder dystocia	4	RR: 0.38 (0.19, 0.75)	16/ 0.09	RR: 0.39 (0.26, 0.60) (0.23, 0.68)	0/ 0.00	50.1	No	RR: 0.39 (0.26, 0.60)	2
Henderson 2019 (25)	Treating asymptomatic bacteriuria	Pyelonephritis	2	RR: 0.29 (0.15, 0.57)	0/ 0.00	RR: 0.25 (0.16, 0.39) (0.07, 0.87)	48/ 0.28	15.1	No	RR: 0.30 (0.23, 0.40)	3
Higgins 2016 (26)	BCG	Mortality	8	RR: 0.46 (0.30, 0.69)	63/ 0.19	RR: 0.51 (0.36, 0.72) (0.18, 1.46)	67/ 0.19	70.1	No	RR: 0.57 (0.48, 0.68)	3
Higgins 2016 (26)	Measles containing vaccines	Mortality	13	RR: 0.53 (0.40, 0.70)	67/ 0.14	RR: 0.57 (0.45, 0.72) (0.27, 1.21)	58/ 0.11	80.4	No	RR: 0.65 (0.57, 0.74)	3
Hopley 2010 (27)	Total hip arthroplasty	Reoperation	6	RR: 0.45 (0.19, 1.08)	23/ 0.28	RR: 0.66 (0.33, 1.32) (0.13, 3.42)	34/ 0.39	56.1	No	RR: 0.72 (0.43, 1.20)	2
Hopley 2010 (27)	Total hip arthroplasty	Dislocation	5	RR: 0.79 (0.27, 2.35)	18/ 0.28	RR: 1.20 (0.52, 2.76) (0.28, 5.08)	12/ 0.17	63.7	No	RR: 1.16 (0.54 2.52)	2

Hopley 2010 (27)	Total hip arthroplasty	Deep infection	4	RR: 0.91 (0.25, 3.28)	0/ 0.00	RR: 1.37 (0.64, 2.94) (0.50, 3.73)	0/ 0.00	35.9	No	RR: 1.37 (0.64, 2.94)	2
Hüpfl 2010 (28)	Chest-compression-only cardio-pulmonary resuscitation	Survival	7	RR: 0.96 (0.83, 1.11)	0/ 0.00	RR: 1.04 (0.92, 1.19) (0.83, 1.31)	13/ 0.01	61.9	No	RR: 1.05 (0.93, 1.18)	3
Jamal 2013 (29)	Non-calcium-based phosphate binders	Mortality	3	RR: 0.89 (0.78, 1.00)	0/ 0.00	RR: 0.87 (0.77, 0.97) (0.67, 1.12)	28/ 0.01	49.1	Yes	RR: 0.89 (0.82, 0.96)	2
Jefferson 2010 (30)	Parenteral influenza vaccine	Influenza-like illness	30	RR: 0.76 (0.66, 0.87)	57/ 0.07	RR: 0.73 (0.64, 0.82) (0.43, 1.22)	54/ 0.06	85.6	No	RR: 0.70 (0.65, 0.75)	3
Jefferson 2010 (30)	Parenteral influenza vaccine	Influenza	10	RR: 0.51 (0.27, 0.97)	64/ 0.52	RR: 0.51 (0.32, 0.80) (0.13, 2.02)	59/ 0.34	68.7	No	RR: 0.60 (0.47, 0.78)	2
Jefferson 2012 (31)	Inactivated influenza vaccines	Influenza	1	RR: 0.20 (0.10, 0.39)	NA	RR: 0.37 (0.26, 0.53) (0.15, 0.92)	44/ 0.11	15.2	No	RR: 0.34 (0.27, 0.43)	2
Jefferson 2012 (31)	Inactivated influenza vaccines	Influenza-like illness	2	RR: 0.29 (0.07, 1.15)	95/ 1.43	RR: 0.56 (0.46, 0.68) (0.33, 0.94)	87/ 0.04	34.8	Yes	RR: 0.74 (0.71, 0.77)	2
Jin 2012 (32)	High total flavonoids	Colorectal neoplasms	3	RR: 1.00 (0.80, 1.25)	66/ 0.02	RR: 1.03 (0.88, 1.20) (0.56, 1.88)	56/ 0.01	69.6	No	RR: 1.02 (0.93, 1.13)	3
Kansagara 2013 (34)	Transfusion	Mortality	11	RR: 2.49 (1.40, 4.43)	97/ 0.94	RR: 1.84 (1.10, 3.07) (0.20, 16.54)	96/ 1.00	74.4	No	RR: 3.32 (3.03, 3.65)	3
Keag 2018 (35)	Caesarean section	Urinary incontinence	8	OR: 0.56 (0.48, 0.66)	70/ 0.04	OR: 0.58 (0.50, 0.68) (0.36, 0.94)	68/ 0.04	90.0	No	OR: 0.62 (0.57, 0.67)	3
Keag 2018 (35)	Caesarean section	Fecal incontinence	5	OR: 1.04 (0.73, 1.48)	72/ 0.10	OR: 1.11 (0.78, 1.58) (0.38, 3.26)	71/ 0.12	93.6	No	OR: 1.11 (0.94, 1.31)	3
Kredo 2014 (36)	Antiretroviral therapy by nurses	Mortality	2	RR: 1.23 (1.14, 1.33)	0/ 0.00	RR: 1.13 (0.94, 1.36) (0.13, 9.93)	76/ 0.02	64.7	Yes	RR: 1.17 (1.10, 1.26)	3

Kredo 2014 (36)	Antiretroviral therapy by nurses	Attrition	2	RR: 0.30 (0.05, 1.94)	98/ 1.77	RR: 0.43 (0.21, 0.86) (0.00, 2691.24)	95/ 0.35	65.6	Yes	RR: 0.75 (0.71, 0.79)	3
Kredo 2014 (36)	Nurses for maintenance of antiretroviral therapy	Mortality	1	RR: 0.19 (0.05, 0.78)	NA	RR: 0.61 (0.28, 1.35) (0.00, 2756.46)	56/ 0.28	20.2	Yes	RR: 0.79 (0.54, 1.16)	3
Li 2014 (37)	Exenatide	Acute pancreatitis/ Admission for acute pancreatitis	2	RR: 0.92 (0.69, 1.22)	0/ 0.00	RR: 0.92 (0.69, 1.22) (0.64, 1.32)	0/ 0.00	96.0	No	RR: 0.92 (0.69, 1.21)	2
Li 2016 (38)	DDP-4 Inhibitors	Heart failure	4	RR: 1.10 (1.04, 1.17)	0/ 0.00	RR: 1.10 (1.04, 1.17) (1.03, 1.16)	0/ 0.00	98.4	No	RR: 1.10 (1.04, 1.17)	2
Li 2016 (38)	DDP-4 Inhibitors	Hospital admission for heart failure	6	OR: 0.85 (0.74, 0.97)	33/ 0.01	OR: 0.94 (0.83, 1.08) (0.66, 1.36)	55/ 0.02	58.1	Yes	OR: 0.97 (0.90, 1.05)	2
Matthews 2018 (39)	Tamoxifen	Heart failure	2	RR: 0.85 (0.66, 1.09)	10/ 0.00	RR: 0.74 (0.53, 1.04) (0.02, 29.27)	59/ 0.05	70.5	No	RR: 0.75 (0.61, 0.92)	3
Menne 2019 (40)	SGLT-2 inhibitors	Acute kidney injury	5	OR: 0.40 (0.31, 0.52)	39/ 0.03	OR: 0.58 (0.49, 0.69) (0.41, 0.99)	27/ 0.05	36.9	No	OR: 0.62 (0.56, 0.68)	2
Mesgarpour 2017 (41)	Erythropoesis stimulating agents	Venous thromboembolism	5	RR: 1.92 (0.64, 5.76)	75/ 1.03	RR: 1.26 (0.76, 2.10) (0.20, 8.14)	84/ 0.70	28.4	No	RR: 1.71 (1.45, 2.01)	2
Mesgarpour 2017 (41)	Erythropoesis stimulating agents	Mortality	7	RR: 1.08 (0.66, 1.78)	91/ 0.35	RR: 0.88 (0.64, 1.21) (0.21, 3.71)	92/ 0.46	33.5	No	RR: 2.20 (2.15, 2.25)	2
Moberley 2013 (42)	Pneumococcal polysaccharide vaccines	Invasive pneumococcal disease	2	OR: 0.57 (0.36, 0.89)	0/ 0.00	OR: 0.40 (0.26, 0.61) (0.18, 0.85)	12/ 0.07	51.9	No	OR: 0.42 (0.29, 0.59)	2
Molnar 2015 (43)	Neoral (cyclosporine)	Acute rejection of kidney transplant	2	OR: 0.46 (0.25, 0.86)	5/ 0.02	OR: 0.74 (0.36, 1.54) (0.04, 12.62)	56/ 0.29	50.4	Yes	OR: 0.71 (0.46, 1.10)	2
Navarese 2013 (44)	Early intervention for NSTE-ACS	Mortality	4	OR: 0.80 (0.63, 1.02)	78/ 0.04	OR: 0.82 (0.69, 0.97) (0.54, 1.24)	45/ 0.03	74.8	Yes	OR: 0.86 (0.80, 0.94)	2
Navarese 2013 (44)	Early intervention for NSTE-ACS	Myocardial infarction	3	OR: 0.86 (0.69, 1.08)	86/ 0.03	OR: 0.97 (0.77, 1.22) (0.48, 1.94)	81/ 0.08	49.4	No	OR: 0.90 (0.83, 0.97)	2

Navarese 2013 (44)	Early intervention for NSTE-ACS	Major bleeding	3	OR: 1.12 (0.69, 1.82)	92/ 0.17	OR: 0.92 (0.68, 1.24) (0.39, 2.15)	70/ 0.11	56.3	No	OR: 1.00 (0.88, 1.13)	2
Nelson 2010 (45)	Caesarean section	Anal incontinence, feces	12	OR: 0.91 (0.72, 1.16)	0/ 0.00	OR: 0.92 (0.74, 1.16) (0.72, 1.19)	0/ 0.00	90.0	No	OR: 0.92 (0.74, 1.16)	3
Nelson 2010 (45)	Caesarean section	Anal incontinence, flatus	4	OR: 1.02 (0.87, 1.20)	0/ 0.00	OR: 1.00 (0.86, 1.16) (0.78, 1.28)	0/ 0.00	90.3	No	OR: 1.00 (0.86, 1.16)	3
Nieuwenhuijse 2014 (46)	Ceramic-on-ceramic bearings for total hip arthroplasty	Harris Hip Score	3	MD: -0.50 (-2.09, 1.09)	62/ 1.08	MD: -0.29 (-0.96, 0.38) (-1.81, 1.22)	32/ 0.31	40.7	No	MD: -0.20 (-0.66, 0.26)	2
Nieuwenhuijse 2014 (46)	High-flexion total knee arthroplasty	Flexion (degrees)	26	MD: 3.78 (1.64, 5.92)	78/ 19.12	MD: 2.91 (1.56, 4.27) (-4.42, 10.25)	73/ 12.7	53.2	No	MD: 2.49 (1.84, 3.14)	2
Nieuwenhuijse 2014 (46)	Gender-specific total knee arthroplasty	Flexion-extension range (degrees)	2	MD: 3.15 (-0.03, 6.34)	29/ 1.58	MD: 1.80 (0.40, 3.21) (-0.53, 4.14)	9/ 0.40	25.6	Yes	MD: 1.85 (0.54, 3.16)	2
Nikooie 2019 (47)	Second generation antipsychotics	Sedation	3	RR: 1.84 (0.40, 8.54)	34/ 0.84	RR: 1.29 (0.95, 1.74) (0.91, 1.83)	0/ 0.00	6.0	No	RR: 1.29 (0.95, 1.74)	2
Nikooie 2019 (47)	Second generation antipsychotics	Neurologic outcomes	5	RR: 0.76 (0.59, 0.99)	0/ 0.00	RR: 0.73 (0.57, 0.93) (0.56, 0.95)	0/ 0.00	91.0	No	RR: 0.73 (0.57, 0.93)	2
Ochen 2019 (48)	Surgery for achilles tendon rupture	Re-rupture	18	RR: 0.42 (0.28, 0.65)	30/ 0.19	RR: 0.43 (0.31, 0.60) (0.20, 0.96)	21/ 0.12	69.6	No	RR: 0.65 (0.54, 0.79)	2
Ochen 2019 (48)	Surgery for achilles tendon rupture	Complications	15	RR: 2.93 (2.28, 3.75)	0/ 0.00	RR: 2.72 (1.84, 4.02) (0.84, 8.82)	41/ 0.28	57.8	No	RR: 2.63 (2.13, 3.27)	2
Pittas 2010 (49)	High vitamin D	Hypertension	3	RR: 0.57 (0.41, 0.79)	0/ 0.00	RR: 0.68 (0.43, 1.07) (0.10, 4.51)	77/ 0.14	61.8	Yes	RR: 1.00 (0.95, 1.05)	3
Raman 2013 (50)	Carotid endarterectomy	Ipsilateral stroke	2	RR: 0.47 (0.05, 4.46)	83/ 2.19	RR: 0.70 (0.51, 0.97) (0.29, 1.69)	38/ 0.05	11.9	Yes	RR: 0.72 (0.58, 0.89)	2
Raman 2013 (50)	Carotid endarterectomy	Any stroke	3	RR: 0.73 (0.43, 1.22)	0/ 0.00	RR: 0.67 (0.57, 0.79) (0.53, 0.84)	0/ 0.00	9.7	Yes	RR: 0.67 (0.57, 0.79)	2

Raman 2013 (50)	Carotid artery stenting	Periprocedural stroke	5	RR: 1.91 (1.72, 2.11)	7/ 0.00	RR: 1.91 (1.74, 2.10) (1.69, 2.16)	0/ 0.00	98.2	No	RR: 1.91 (1.74, 2.10)	2
Schweizer 2013 (51)	Nasal deconolization	Surgical site infection	6	RR: 0.40 (0.28, 0.57)	0/ 0.00	RR: 0.48 (0.33, 0.69) (0.18, 1.26)	44/ 0.15	52.4	No	RR: 0.54 (0.42, 0.69)	2
Schweizer 2013 (51)	Glycopeptide prophylaxis	Surgical site infection	7	RR: 0.35 (0.12, 1.03)	80/ 1.44	RR: 0.71 (0.48, 1.05) (0.22, 2.27)	62/ 0.25	38.5	No	RR: 1.04 (0.66, 1.24)	2
Silvain 2012 (52)	Enoxaparin	Mortality	7	RR: 0.50 (0.40, 0.62)	0/ 0.00	RR: 0.64 (0.49, 0.82) (0.32, 1.26)	46/ 0.08	48.9	No	RR: 0.66 (0.56, 0.77)	2
Silvain 2012 (52)	Enoxaparin	Major bleeding	7	RR: 0.72 (0.56, 0.93)	0/ 0.00	RR: 0.81 (0.66, 1.00) (0.49, 1.37)	30/ 0.05	42.5	Yes	RR: 0.84 (0.72, 0.98)	2
Silvain 2012 (52)	Enoxaparin	Death or Myocardial infarction	7	RR: 0.44 (0.35, 0.55)	0/ 0.00	RR: 0.67 (0.55, 0.81) (0.37, 1.21)	58/ 0.07	34.3	No	RR: 0.77 (0.71, 0.85)	2
Suthar 2012 (53)	Antiretroviral therapy	Tuberculosis infection	9	HR: 0.32 (0.25, 0.41)	27/ 0.03	HR: 0.35 (0.29, 0.44) (0.22, 0.57)	26/ 0.03	78.9	No	HR: 0.37 (0.31, 0.44)	3
Te Morenga 2013 (54)	High sugar intake	Weight gain (kg)	4	MD: 0.31 (-0.07, 0.68)	99/ 0.14	MD: 0.51 (0.26, 0.75) (-0.36, 1.37)	99/ 0.14	42.0	Yes	MD: 0.59 (0.58, 0.60)	2
Te Morenga 2013 (54)	High sugar intake	BMI (kg/m^2)	4	MD: -0.02 (-0.05, 0.00)	74/ 0.00	MD: -0.02 (-0.05,-0.00) (-0.05, 0.09)	58/ 0.00	5.0	No	MD: -0.01 (-0.03, -0.00)	2
Thomas 2010 (55)	Influenza vaccines	Influenza-like illness	1	RR: 0.31 (0.26, 0.36)	NA	RR: 0.53 (0.31, 0.89) (0.08, 3.48)	94/ 0.28	24.5	No	RR: 0.48 (0.43, 0.53)	3
Tickell-Painter 2017 (56)	Mefloquine	Discontinuation due to adverse effects	9	RR: 2.73 (1.84, 4.06)	31/ 0.11	RR: 2.78 (2.05, 3.77) (1.57, 4.91)	15/ 0.04	79.2	No	RR: 2.85 (2.19, 3.71)	2
Tickell-Painter 2017 (56)	Mefloquine	Serious adverse events or effects	2	RR: 3.09 (0.38, 24.95)	0/ 0.00	RR: 1.31 (0.33, 5.23) (0.14, 12.39)	0/ 0.00	43.7	No	RR: 1.31 (0.33, 5.23)	3
Tickell-Painter 2017 (56)	Mefloquine	Nausea	3	RR: 1.86 (1.42, 2.42)	0/ 0.00	RR: 1.56 (1.30, 1.87) (1.16, 2.09)	0/ 0.00	46.3	No	RR: 1.56 (1.30, 1.87)	3

Tricco 2018 (57)	Live-attenuated zoster vaccines	Suspected Herpes Zoster	3	RR: 0.48 (0.27, 0.83)	99/ 0.24	RR: 0.55 (0.40, 0.77) (0.20, 1.52)	97/ 0.14	56.6	No	RR: 0.72 (0.70, 0.74)	2
Vinceti 2018 (58)	Selenium	Any cancer	7	RR: 0.72 (0.55, 0.93)	46/ 0.06	RR: 0.86 (0.73, 1.01) (0.52, 1.42)	64/ 0.04	45.7	Yes	RR: 0.94 (0.88, 1.01)	3
Vinceti 2018 (58)	Selenium	Cancer mortality	7	RR: 0.76 (0.59, 0.97)	66/ 0.07	RR: 0.78 (0.64, 0.95) (0.44, 1.39)	65/ 0.05	73.2	No	RR: 0.88 (0.80, 0.96)	3
Vinceti 2018 (58)	Selenium	Colorectal cancer	6	RR: 0.82 (0.72, 0.94)	0/ 0.00	RR: 0.83 (0.74, 0.94) (0.73, 0.95)	0/ 0.00	85.6	No	RR: 0.83 (0.74, 0.94)	3
Wilson 2011 (59)	Training for traditional birth attendants/ assistance by traditional birth attendants	Perinatal mortality	1	RR: 0.82 (0.38, 1.78)	NA	RR: 0.77 (0.67, 0.89) (0.53, 1.13)	52/ 0.01	3.0	Yes	RR: 0.79 (0.73, 0.86)	3
Wilson 2011 (59)	Training for traditional birth attendants/ assistance by traditional birth attendants	Neonatal mortality	2	RR: 0.80 (0.47, 1.37)	0/ 0.00	RR: 0.80 (0.73, 0.88) (0.67, 0.95)	14.0/ 0.00	3.0	Yes	RR: 0.80 (0.74, 0.87)	3
Wilson 2019 (60)	Unilateral knee arthroplasty	Venous thromboembolism	8	RR: 0.42 (0.30, 0.57)	24/ 0.04	RR: 0.43 (0.33, 0.55) (0.29, 0.64)	8/ 0.01	98.1	No	RR: 0.45 (0.37, 0.54)	2
Wilson 2019 (60)	Unilateral knee arthroplasty	Range of movement (degrees)	11	MD: -8.43 (-10.15, -6.71)	86/ 6.20	MD: -7.60 (-9.27, -5.93) (-13.98, -1.22)	91/ 7.85	77.8	No	MD: -8.29 (-8.63, -7.95)	2
Wilson 2019 (60)	Unilateral knee arthroplasty	Operation duration (minutes)	8	MD: -23.80 (-40.43, -7.17)	99/ 491.19	MD: -17.07 (-29.11, -5.04) (-63.37, 29.23)	98/ 365.45	69.8	No	MD: -11.25 (-12.71, -9.97)	2
Yank 2011 (61)	Recombinant factor VII	Mortality	2	RR: 0.91 (0.39, 2.12)	0/ 0.00	RR: 1.08 (0.56, 2.09) (0.25, 4.59)	0/ 0.00	60.6	No	RR: 1.08 (0.56, 2.09)	2
Yank 2011 (61)	Recombinant factor VII	Thromboembolic events	2	RR: 1.81 (0.67, 4.87)	0/ 0.00	RR: 1.88 (0.85, 4.16) (0.33, 10.76)	0/ 0.00	64.5	No	RR: 1.88 (0.85, 4.16)	2

Zhang 2016 (62)	Everolimus-eluting bioresorbable vascular scaffold	Stent thrombosis	3	OR: 2.22 (1.00, 4.93)	0/ 0.00	OR: 2.09 (1.20, 3.64) (1.04, 4.18)	0/ 0.00	48.9	Yes	OR: 2.09 (1.20, 3.64)	2
Zhang 2016 (62)	Everolimus-eluting bioresorbable vascular scaffold	Mortality	4	OR: 0.63 (0.24, 1.63)	0/ 0.00	OR: 0.73 (0.34, 1.57) (0.18, 2.97)	15/ 0.20	48.3	No	OR: 0.82 (0.42, 1.60)	2
Zhang 2016 (62)	Everolimus-eluting bioresorbable vascular scaffold	Cardiac death	4	OR: 0.94 (0.43, 2.06)	0/ 0.00	OR: 1.05 (0.53, 2.12) (0.42, 2.63)	0/ 0.00	78.6	No	OR: 1.05 (0.53, 2.12)	2
Zhang 2017 (63)	Percutaneous coronary intervention	Mortality	17	HR: 1.07 (0.92, 1.26)	37/ 0.03	HR: 1.05 (0.93, 1.20) (0.73, 1.52)	32/ 0.03	74.4	No	HR: 1.08 (0.98, 1.19)	2
Zhang 2017 (63)	Percutaneous coronary intervention	Cardiovascular mortality	5	HR: 1.08 (0.51, 2.28)	78/ 0.49	HR: 1.05 (0.69, 1.59) (0.29, 3.81)	72/ 0.25	48.8	No	HR: 1.33 (1.09, 1.62)	2
Zhang 2017 (63)	Percutaneous coronary intervention	Myocardial infarction	5	HR: 2.00 (1.65, 2.44)	0/ 0.00	HR: 1.69 (1.22, 2.33) (0.71, 4.03)	57/ 0.12	46.3	No	HR: 1.66 (1.42, 1.94)	2
Ziff 2015 (64)	Digoxin	Mortality	8	RR: 1.60 (1.31, 1.96)	63/ 0.05	RR: 1.38 (1.15, 1.66) (0.77, 2.49)	75/ 0.06	69.8	No	RR: 1.08 (1.03, 1.14)	3
Ziff 2015 (64)	Digoxin	Cardiovascular mortality	3	RR: 2.53 (1.12, 5.70)	96/ 0.48	RR: 1.71 (1.04, 2.80) (0.26, 11.38)	96/ 0.29	58.1	No	RR: 1.15 (1.08, 1.22)	3
Ziff 2015 (64)	Digoxin	Hospital admission	4	RR: 0.92 (0.85, 0.99)	64/ 0.00	RR: 0.93 (0.88, 0.98) (0.80, 1.09)	61/ 0.00	62.2	No	RR: 0.92 (0.89, 0.95)	2

BCG: Bacillus Calmette-Guérin; BMI: Body Mass Index; BoE: Bodies of Evidence; CE: Common Effects; CI: Confidence Interval; CRC: Colorectal Cancer; CS: Cohort Studies; DDP 4: Dipeptidylpeptidase-4; HIV: Human Immunodeficiency Virus; HR: Hazard Ratio; MD: Mean Difference; NA: Not Applicable; NSTE-ACS: Non–ST-Segment Elevation Acute Coronary Syndromes; OR: Odds Ratio; PI/ECO: Population – Intervention/Exposure – Comparator – Outcome; RCT: Randomized Controlled Trial; RE: Random Effects; RR: Risk Ratio; SGLT-2: Sodium Dependent Glucose Transporter 2;

* PI/ECO similarity degree: 1=more or less identical; 2=similar but not identical; 3=broadly similar;

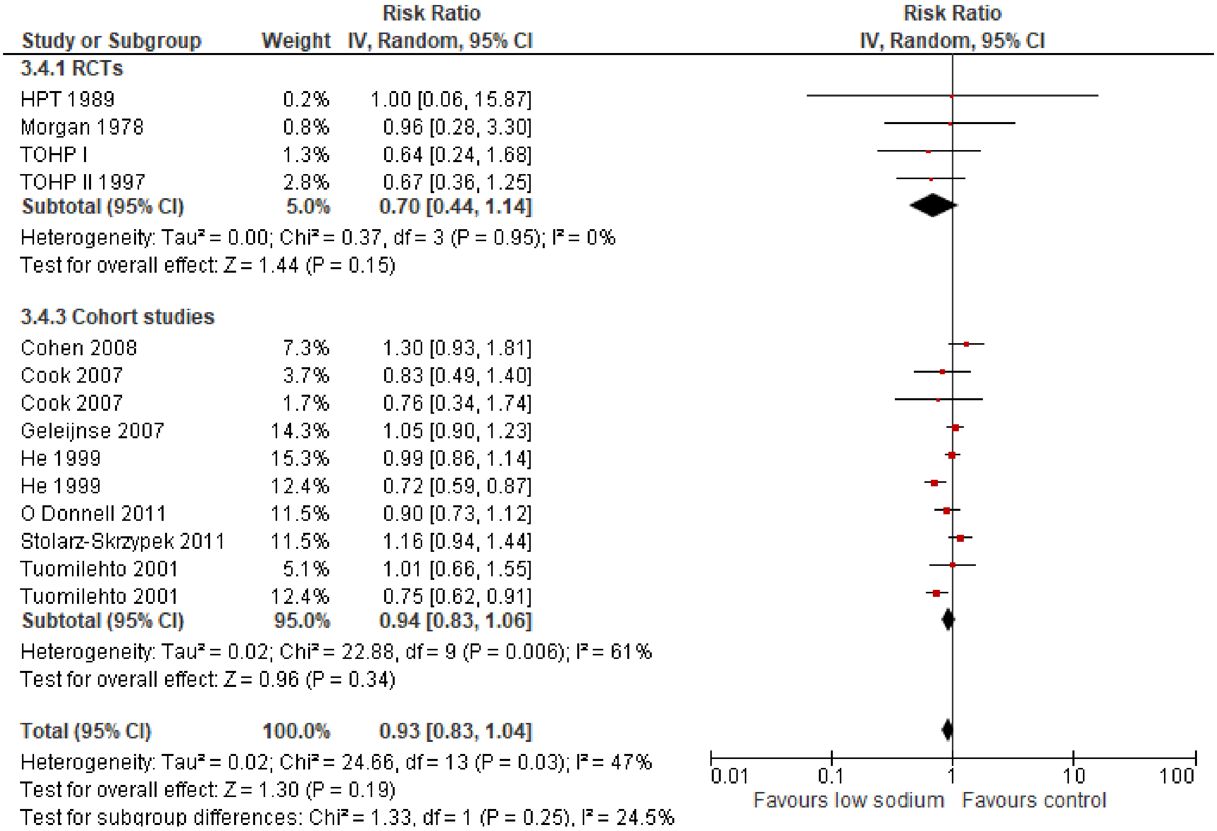

Figure S1: Aburto 2013; Intervention/ Exposure: Low sodium; Outcome: All-cause mortality

Study or Subgroup	Weight	Risk Ratio IV, Random, 95% CI
**3.5.1 RCTs**		
Morgan 1978	2.0%	1.16 [0.39, 3.45]
TONE 1998	6.7%	0.80 [0.53, 1.21]
**Subtotal (95% CI)**	**8.7%**	**0.84 [0.57, 1.23]**
Heterogeneity: $Tau^2 = 0.00$; $Chi^2 = 0.39$, df = 1 (P = 0.53); $I^2 = 0\%$		
Test for overall effect: Z = 0.88 (P = 0.38)		
**3.5.2 Cohort studies**		
Alderman 1995	1.4%	0.40 [0.11, 1.48]
Alderman 1995	4.7%	2.69 [1.49, 4.85]
Cohen 2008	5.5%	1.88 [1.13, 3.13]
Cook 2007	7.7%	0.79 [0.56, 1.10]
Cook 2007	4.2%	0.48 [0.25, 0.92]
Geleijnse 2007	8.8%	1.30 [1.01, 1.67]
He 1999	9.0%	0.86 [0.68, 1.09]
He 1999	7.9%	0.66 [0.48, 0.90]
O Donnell 2011	9.8%	0.84 [0.71, 1.01]
Stolarz-Skrzypek 2011	9.3%	1.07 [0.86, 1.33]
Tuomilehto 2001	4.5%	0.64 [0.35, 1.18]
Tuomilehto 2001	8.8%	0.70 [0.54, 0.90]
Umesawa 2008	9.8%	0.70 [0.59, 0.84]
**Subtotal (95% CI)**	**91.3%**	**0.90 [0.75, 1.08]**
Heterogeneity: $Tau^2 = 0.07$; $Chi^2 = 53.38$, df = 12 (P < 0.00001); $I^2 = 78\%$		
Test for overall effect: Z = 1.18 (P = 0.24)		
**Total (95% CI)**	**100.0%**	**0.89 [0.75, 1.06]**
Heterogeneity: $Tau^2 = 0.07$; $Chi^2 = 53.79$, df = 14 (P < 0.00001); $I^2 = 74\%$		
Test for overall effect: Z = 1.31 (P = 0.19)		
Test for subgroup differences: $Chi^2 = 0.08$, df = 1 (P = 0.78), $I^2 = 0\%$		

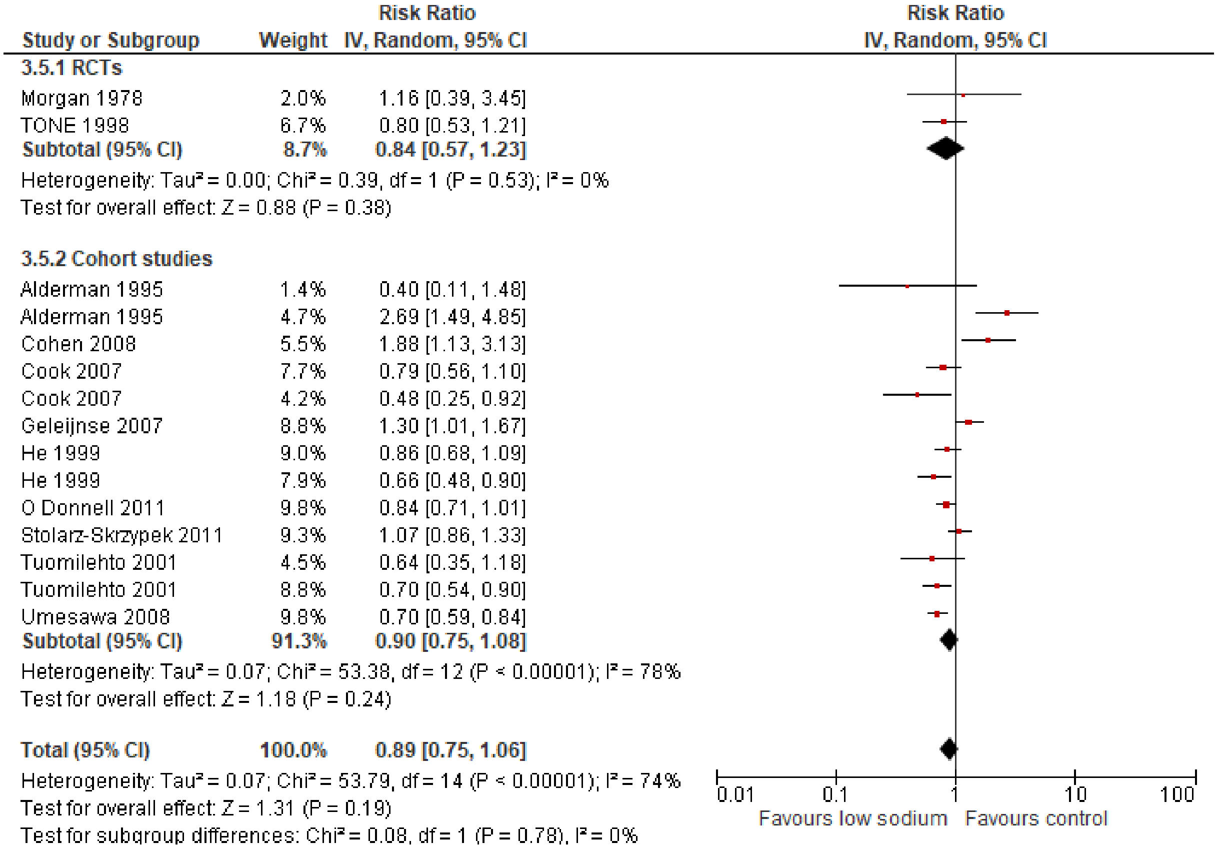

Figure S2: Aburto 2013; Intervention/ Exposure: Low-sodium; Outcome: Cardiovascular disease

Study or Subgroup	Weight	Odds Ratio IV, Random, 95% CI
**4.3.1 RCTs**		
Kono 1996		Not estimable
Flaherty 1985	3.5%	0.25 [0.07, 0.96]
Gu 2011	3.4%	0.46 [0.12, 1.80]
O'Rourke 1981	3.9%	0.73 [0.23, 2.27]
Ohman 1994	2.5%	0.89 [0.12, 6.48]
Ohman 2005	5.2%	0.94 [0.67, 1.30]
Patel 2011	3.3%	1.29 [0.30, 5.43]
Prondzinsky 2010	4.1%	1.39 [0.51, 3.81]
Stone 1997	4.3%	1.40 [0.57, 3.45]
Thiele 2012	3.5%	1.46 [0.39, 5.51]
van 't Hof 1999	2.6%	1.56 [0.24, 9.91]
Vijayalakshmi 2007	1.4%	7.97 [0.38, 167.52]
**Subtotal (95% CI)**	**37.8%**	**0.96 [0.74, 1.24]**
Heterogeneity: Tau² = 0.00; Chi² = 9.09, df = 10 (P = 0.52); I² = 0%		
Test for overall effect: Z = 0.33 (P = 0.74)		
**4.3.2 Cohort studies**		
Moulopoulos 1986	1.5%	0.08 [0.00, 1.38]
Kovack 1997	3.6%	0.23 [0.07, 0.81]
Waksman 1993	3.4%	0.29 [0.07, 1.17]
Stomel 1994	3.4%	0.37 [0.09, 1.49]
Sanborn 2000	5.3%	0.38 [0.29, 0.51]
Gu 2010	4.4%	0.40 [0.17, 0.94]
Zeymer 2013	5.4%	0.57 [0.53, 0.63]
Sjauw 2012	4.9%	0.65 [0.37, 1.14]
Zeymer 2011	4.9%	0.70 [0.40, 1.22]
Ohman 1991	5.3%	1.29 [1.05, 1.59]
Anderson 1997	5.0%	2.26 [1.33, 3.85]
Barron 2001	5.2%	2.33 [1.62, 3.35]
Bengtson1992	4.9%	11.59 [6.41, 20.96]
Brodie 1999	5.1%	13.63 [9.11, 20.40]
**Subtotal (95% CI)**	**62.2%**	**1.02 [0.57, 1.82]**
Heterogeneity: Tau² = 1.03; Chi² = 430.59, df = 13 (P < 0.00001); I² = 97%		
Test for overall effect: Z = 0.06 (P = 0.95)		
**Total (95% CI)**	**100.0%**	**1.02 [0.67, 1.56]**
Heterogeneity: Tau² = 0.86; Chi² = 442.58, df = 24 (P < 0.00001); I² = 95%		
Test for overall effect: Z = 0.09 (P = 0.92)		
Test for subgroup differences: Chi² = 0.04, df = 1 (P = 0.85), I² = 0%		

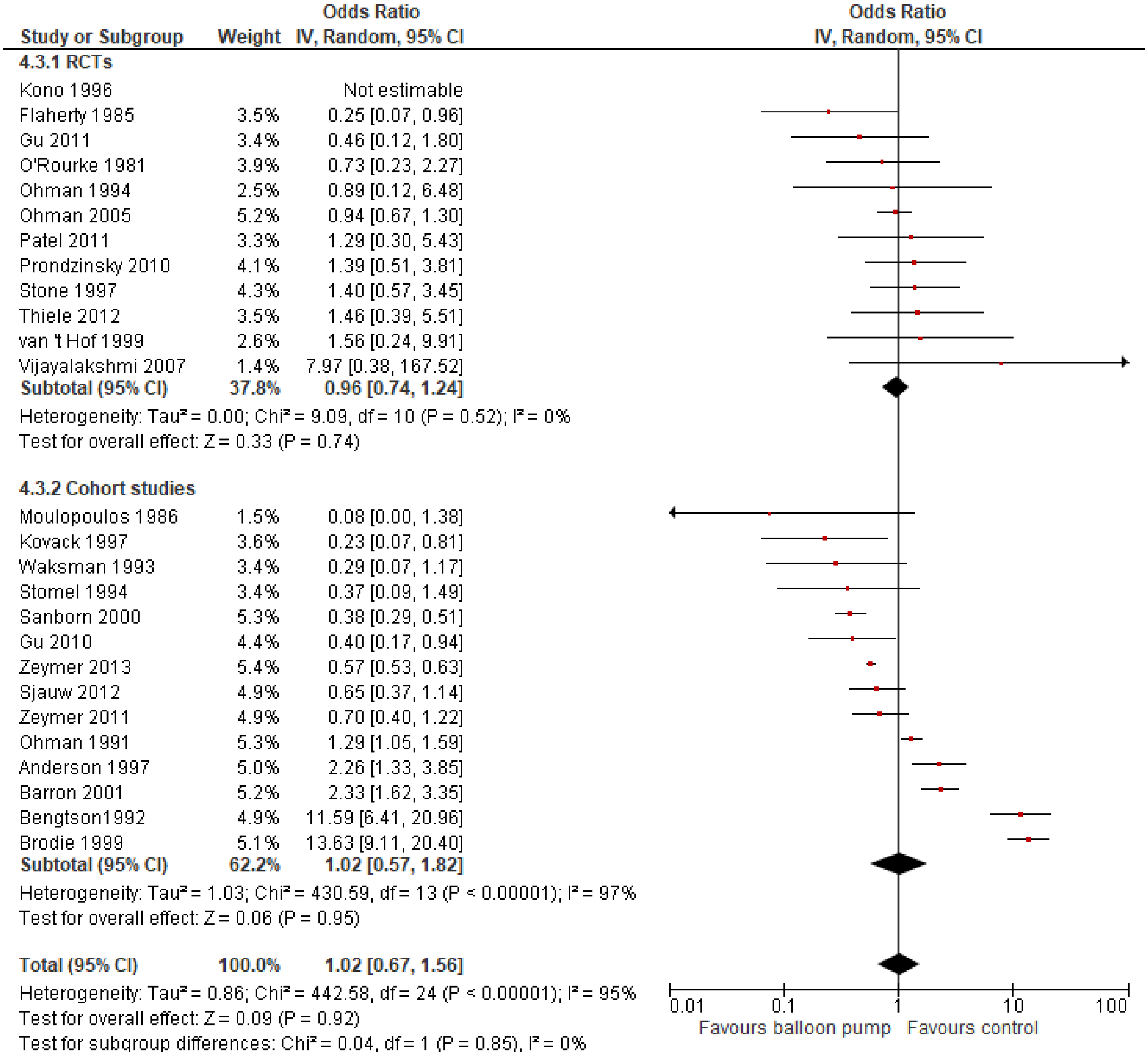

Figure S3: Ahmad 2015; Intervention/ Exposure: Intra-aortic balloon pump; Outcome: All-cause mortality

Study or Subgroup	Weight	Risk Ratio IV, Random, 95% CI
**6.1.1 RCTs**		
Kamolratanakul	6.8%	0.90 [0.84, 0.97]
Walley 2001a	5.8%	1.01 [0.88, 1.15]
Zwarenstein 1998	2.9%	1.19 [0.85, 1.66]
Zwarenstein 2000	3.6%	0.91 [0.68, 1.20]
**Subtotal (95% CI)**	**19.1%**	**0.95 [0.87, 1.03]**
Heterogeneity: Tau² = 0.00; Chi² = 3.98, df = 3 (P = 0.26); I² = 25%		
Test for overall effect: Z = 1.22 (P = 0.22)		
**6.1.2 Cohort studies**		
Akkslip 1999	5.9%	0.83 [0.73, 0.94]
Anuwatnonthakate 2008	7.0%	0.78 [0.74, 0.83]
Cavalcante 2007	7.0%	0.96 [0.92, 1.01]
Chung 2007	5.5%	0.79 [0.68, 0.92]
Daniel 2006	5.0%	0.67 [0.56, 0.81]
Das 2014	1.8%	0.77 [0.47, 1.24]
Ershova 2014	1.1%	0.69 [0.36, 1.31]
Juan 2006	3.0%	0.36 [0.26, 0.49]
Mathema 2001	6.6%	0.86 [0.79, 0.94]
Mohr 2017	4.7%	1.02 [0.83, 1.25]
Nirupa 2005	4.5%	0.96 [0.77, 1.19]
Olle-Goig 2001	5.5%	0.67 [0.57, 0.78]
Szczesniak 2009	5.3%	0.77 [0.65, 0.92]
Tsuchida 2003	4.1%	0.78 [0.62, 1.00]
Xu 2009	6.8%	0.84 [0.79, 0.91]
Yen 2013	7.1%	1.04 [1.01, 1.08]
**Subtotal (95% CI)**	**80.9%**	**0.81 [0.74, 0.88]**
Heterogeneity: Tau² = 0.02; Chi² = 176.26, df = 15 (P < 0.00001); I² = 91%		
Test for overall effect: Z = 4.73 (P < 0.00001)		
**Total (95% CI)**	**100.0%**	**0.84 [0.78, 0.90]**
Heterogeneity: Tau² = 0.02; Chi² = 180.41, df = 19 (P < 0.00001); I² = 89%		
Test for overall effect: Z = 4.60 (P < 0.00001)		
Test for subgroup differences: Chi² = 6.33, df = 1 (P = 0.01), I² = 84.2%		

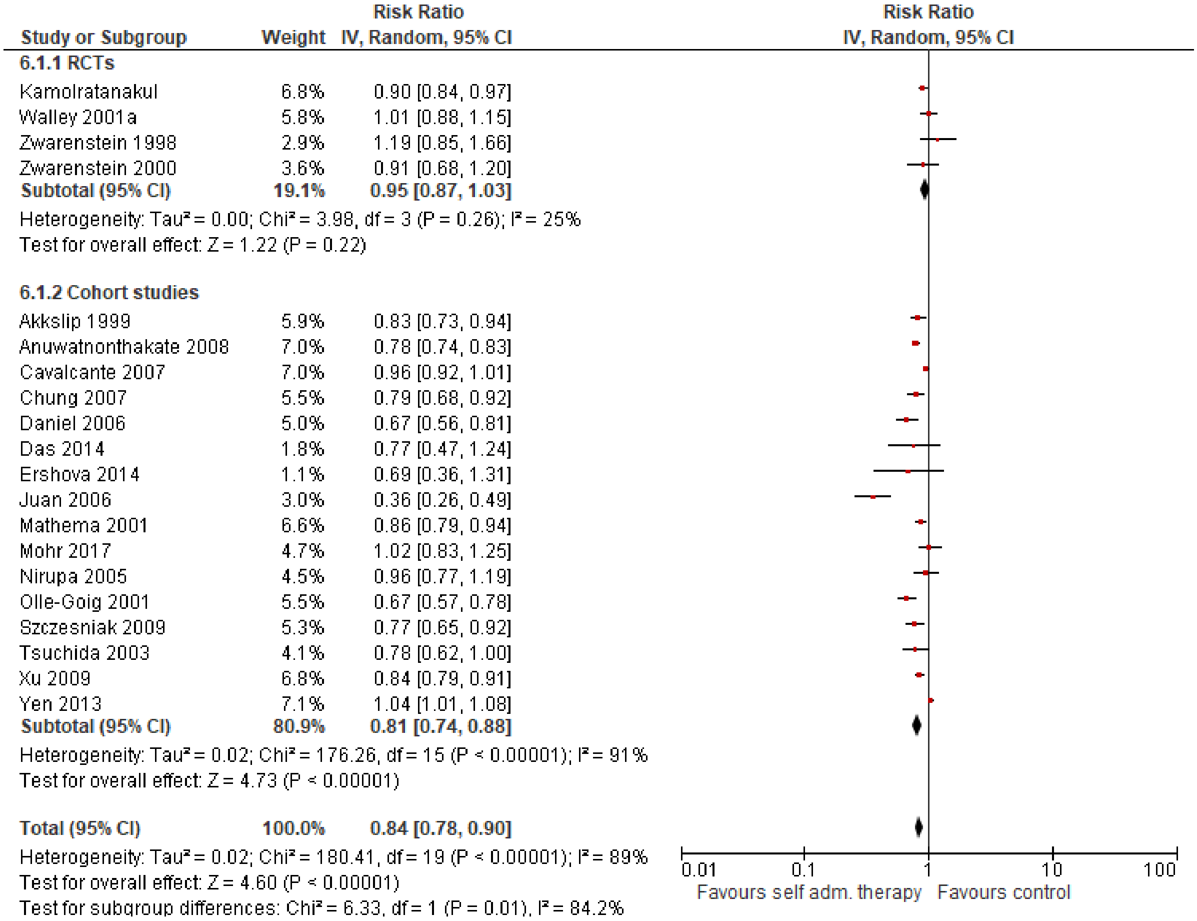

Figure S4: Alipanah 2018; Intervention/ Exposure: Self-administered therapy; Outcome: Treatment success (Risk Ratio >1 indicates a favorable effect)

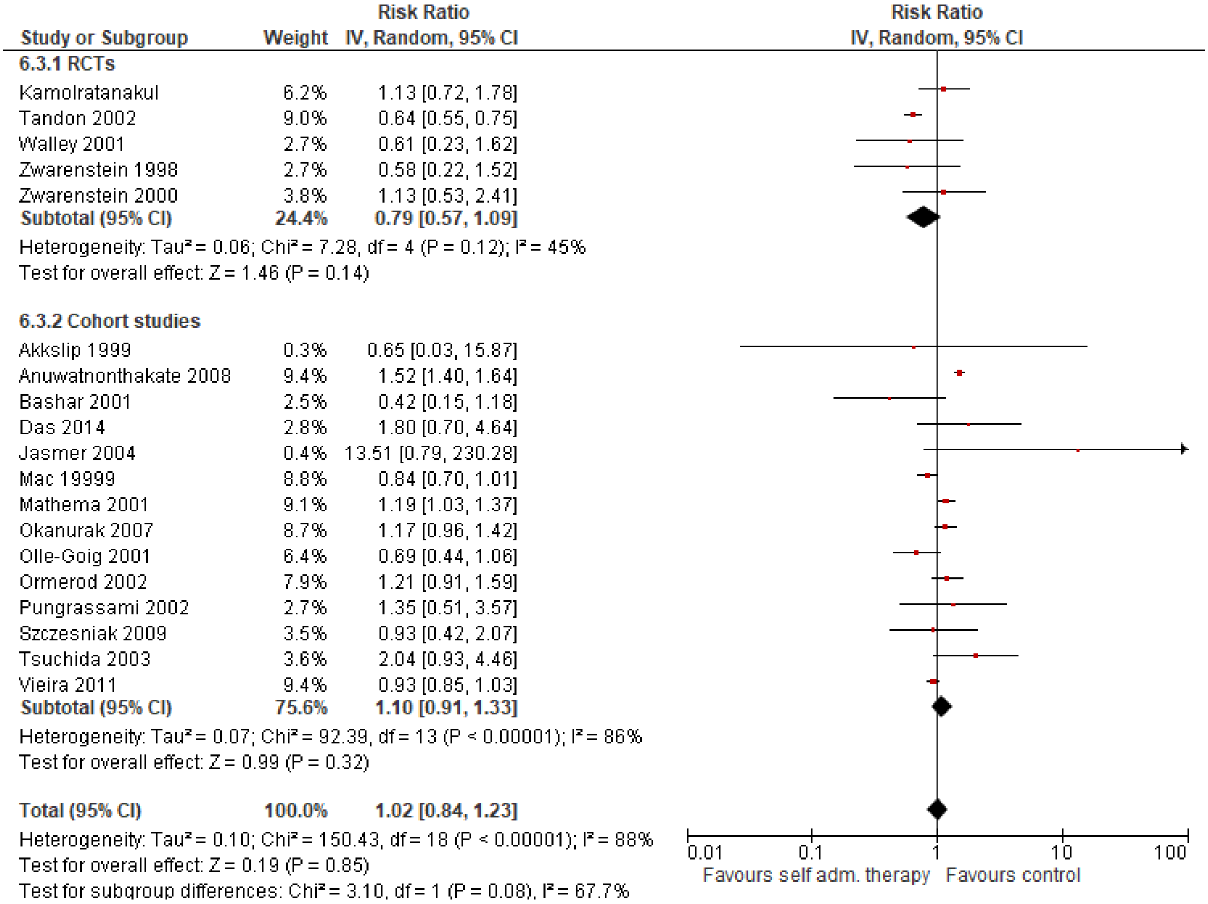

Figure S5: Alipanah 2018; Intervention/ Exposure: Self-administered therapy; Outcome: Treatment completion (Risk Ratio >1 indicates a favorable effect)

Study or Subgroup	Weight	Risk Ratio IV, Random, 95% CI
**6.4.1 RCTs**		
Kamolratanakul	5.1%	0.79 [0.45, 1.39]
Walley 2001	2.9%	0.48 [0.14, 1.65]
Zwarenstein 1998	0.7%	0.29 [0.01, 6.98]
Zwarenstein 2000	1.2%	1.27 [0.12, 13.68]
**Subtotal (95% CI)**	**9.8%**	**0.73 [0.45, 1.19]**
Heterogeneity: Tau² = 0.00; Chi² = 1.07, df = 3 (P = 0.79); I² = 0%		
Test for overall effect: Z = 1.26 (P = 0.21)		
**6.4.2 Cohort studies**		
Abuaku 2010	4.2%	0.41 [0.18, 0.91]
Akkslip 1999	4.5%	1.57 [0.76, 3.24]
Alwood 1994	4.2%	2.97 [1.34, 6.60]
Anuwatnonthakate 2008	6.0%	0.39 [0.31, 0.50]
Bashar 2001	1.5%	6.18 [0.79, 48.30]
Cavalcante 2007	4.9%	3.12 [1.71, 5.70]
Cayla 2004	4.2%	0.20 [0.09, 0.44]
Daniel 2006	5.4%	2.15 [1.34, 3.46]
Das 2014	1.3%	1.08 [0.12, 9.70]
Jasmer 2004	4.0%	3.50 [1.50, 8.18]
Juan 2006	2.0%	1.80 [0.34, 9.64]
Mathema 2001	3.3%	0.64 [0.22, 1.90]
Mohr 2015	3.8%	1.70 [0.68, 4.24]
Nirupa 2005	3.2%	3.69 [1.20, 11.35]
Okanurak 2007	4.4%	0.99 [0.47, 2.10]
Olle-Goig 2001	3.6%	3.28 [1.24, 8.65]
Ormerod 2002	0.9%	6.03 [0.38, 96.13]
Pungrassami 2002	5.1%	0.74 [0.43, 1.27]
Snyder 2016	6.0%	2.23 [1.73, 2.86]
Szczesniak 2009	3.7%	1.32 [0.52, 3.36]
Tsuchida 2003	1.4%	3.80 [0.44, 32.57]
Yen 2013	6.2%	0.83 [0.70, 0.97]
Yen 2017	6.3%	1.47 [1.36, 1.59]
**Subtotal (95% CI)**	**90.2%**	**1.35 [1.00, 1.83]**
Heterogeneity: Tau² = 0.34; Chi² = 220.10, df = 22 (P < 0.00001); I² = 90%		
Test for overall effect: Z = 1.96 (P = 0.05)		
**Total (95% CI)**	**100.0%**	**1.26 [0.95, 1.67]**
Heterogeneity: Tau² = 0.33; Chi² = 225.93, df = 26 (P < 0.00001); I² = 88%		
Test for overall effect: Z = 1.60 (P = 0.11)		
Test for subgroup differences: Chi² = 4.40, df = 1 (P = 0.04), I² = 77.3%		

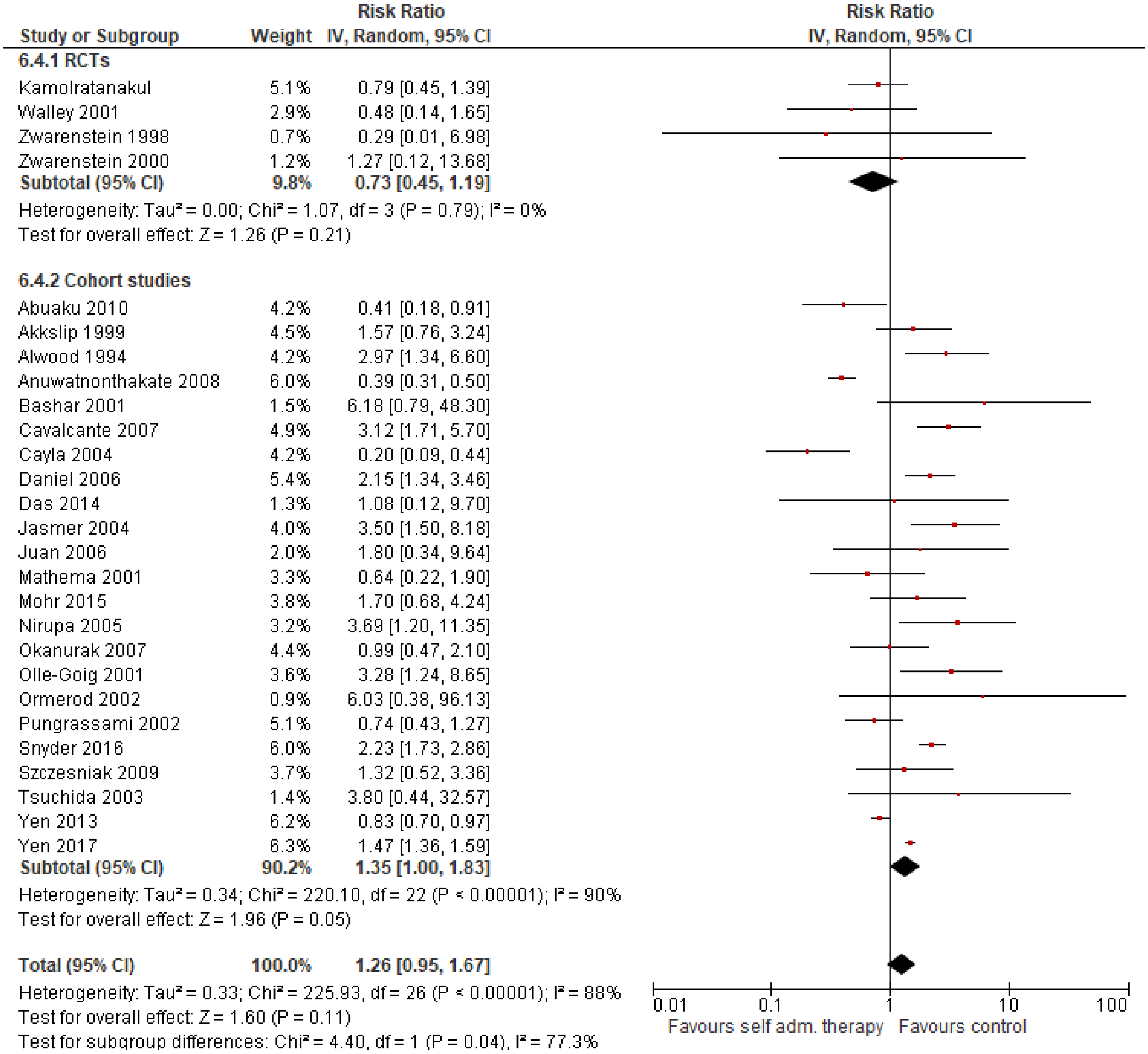

Figure S6: Alipanah 2018; Intervention/ Exposure: Self-administered therapy; Outcome: All-cause mortality

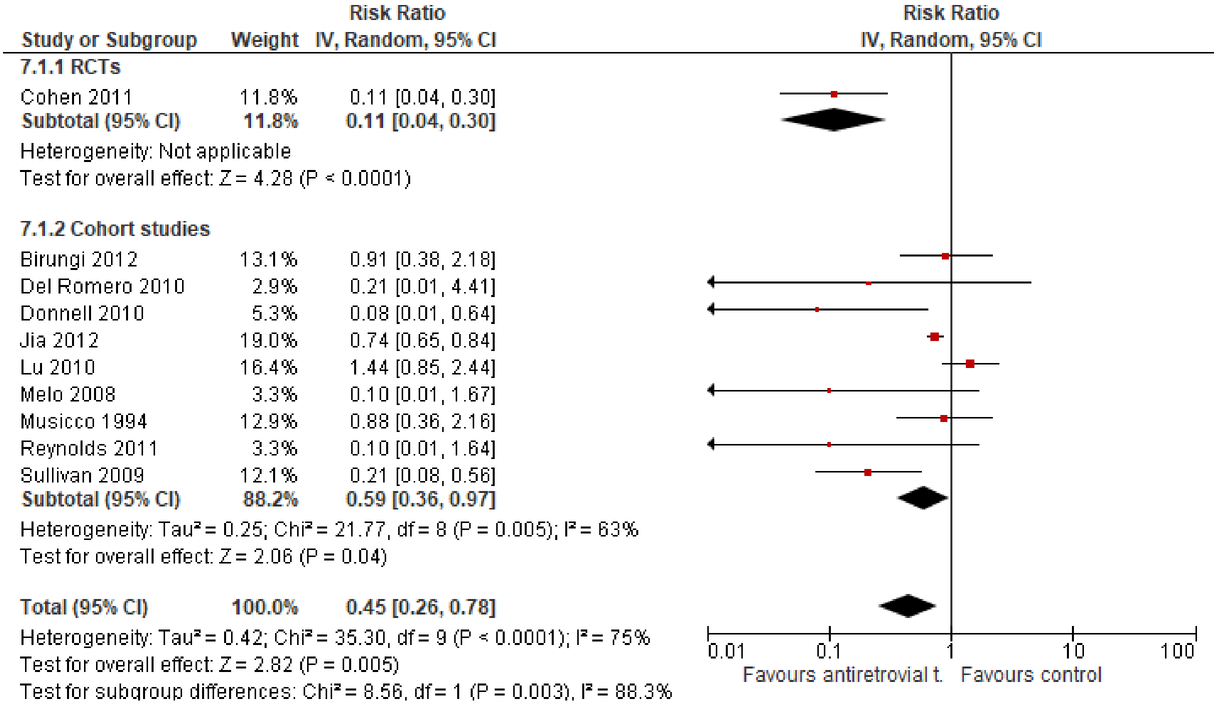

Figure S7: Anglemyer 2013; Intervention/ Exposure: Antiretroviral therapy; Outcome: HIV infection

Study or Subgroup	Weight	Mean Difference IV, Random, 95% CI
**8.1.1 RCTs**		
Ferri 2006	8.4%	1.60 [-1.46, 4.66]
Hsieh 2003	33.9%	-0.60 [-1.27, 0.07]
Madjd 2015	19.1%	0.00 [-1.65, 1.65]
**Subtotal (95% CI)**	**61.4%**	**-0.37 [-1.10, 0.36]**
Heterogeneity: Tau² = 0.07; Chi² = 2.20, df = 2 (P = 0.33); I² = 9%		
Test for overall effect: Z = 1.00 (P = 0.32)		
**8.1.2 Cohort studies**		
Fowler 2008	38.6%	0.77 [0.47, 1.07]
**Subtotal (95% CI)**	**38.6%**	**0.77 [0.47, 1.07]**
Heterogeneity: Not applicable		
Test for overall effect: Z = 5.03 (P < 0.00001)		
**Total (95% CI)**	**100.0%**	**0.23 [-0.77, 1.23]**
Heterogeneity: Tau² = 0.65; Chi² = 14.35, df = 3 (P = 0.002); I² = 79%		
Test for overall effect: Z = 0.45 (P = 0.65)		
Test for subgroup differences: Chi² = 8.03, df = 1 (P = 0.005), I² = 87.6%		

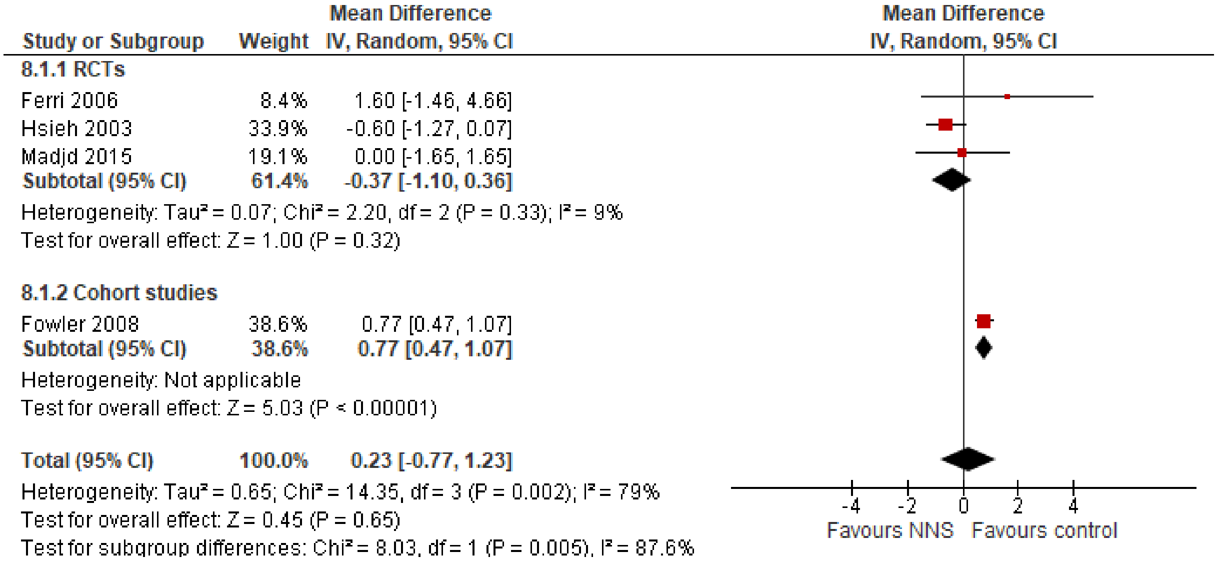

Figure S8: Azad 2017; Intervention/ Exposure: Nonnutritive sweeteners; Outcome: Body Mass Index

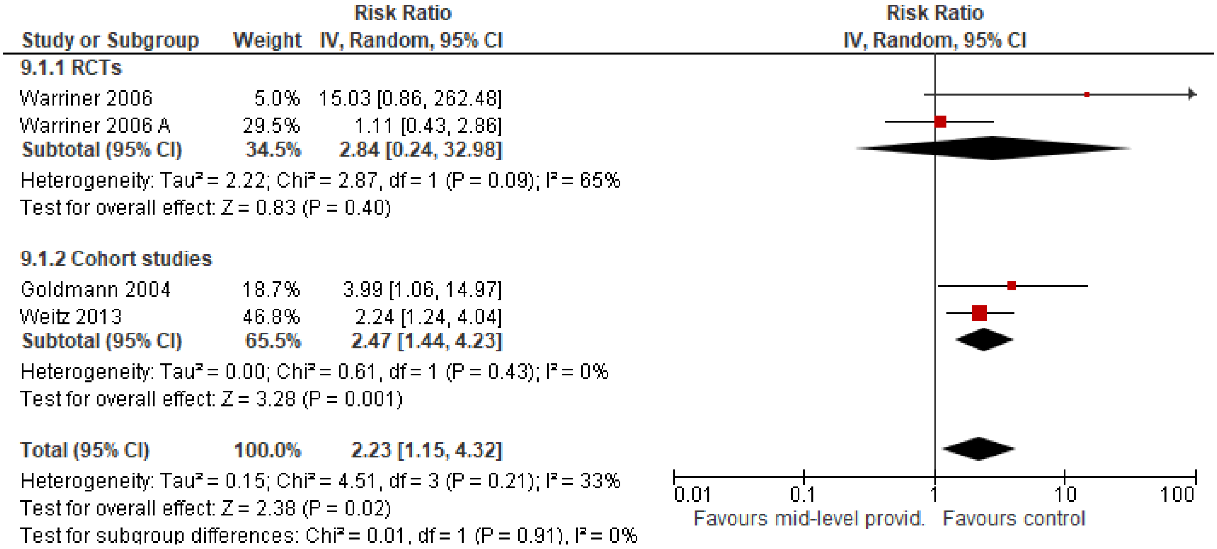

Figure S9: Barnard 2015; Intervention/ Exposure: Surgical abortion by mid-level providers; Outcome: Failure or incomplete abortion

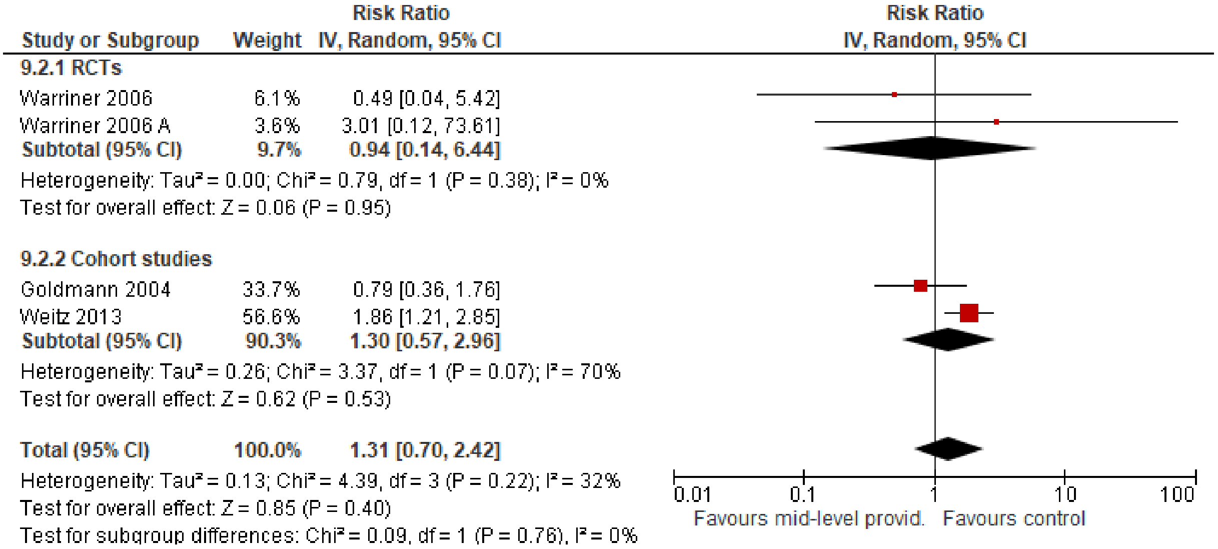

Figure S10: Barnard 2015; Intervention/ Exposure: Surgical abortion by mid-level providers; Outcome: Complications

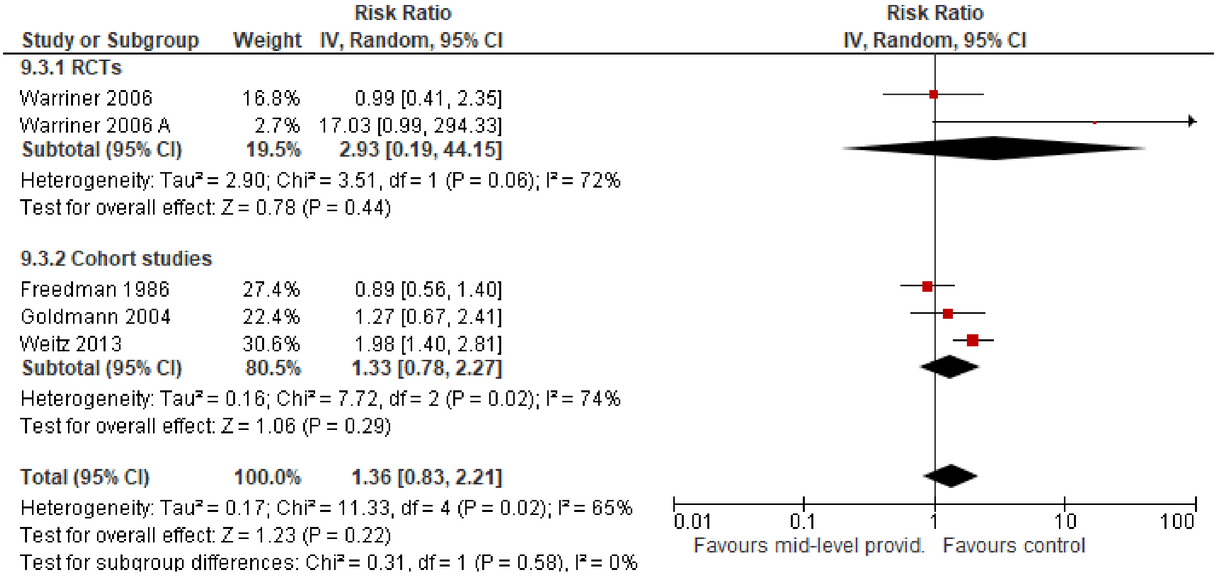

Figure S11: Barnard 2015; Intervention/ Exposure: Surgical abortion by mid-level providers; Outcome: Abortion failure and complications

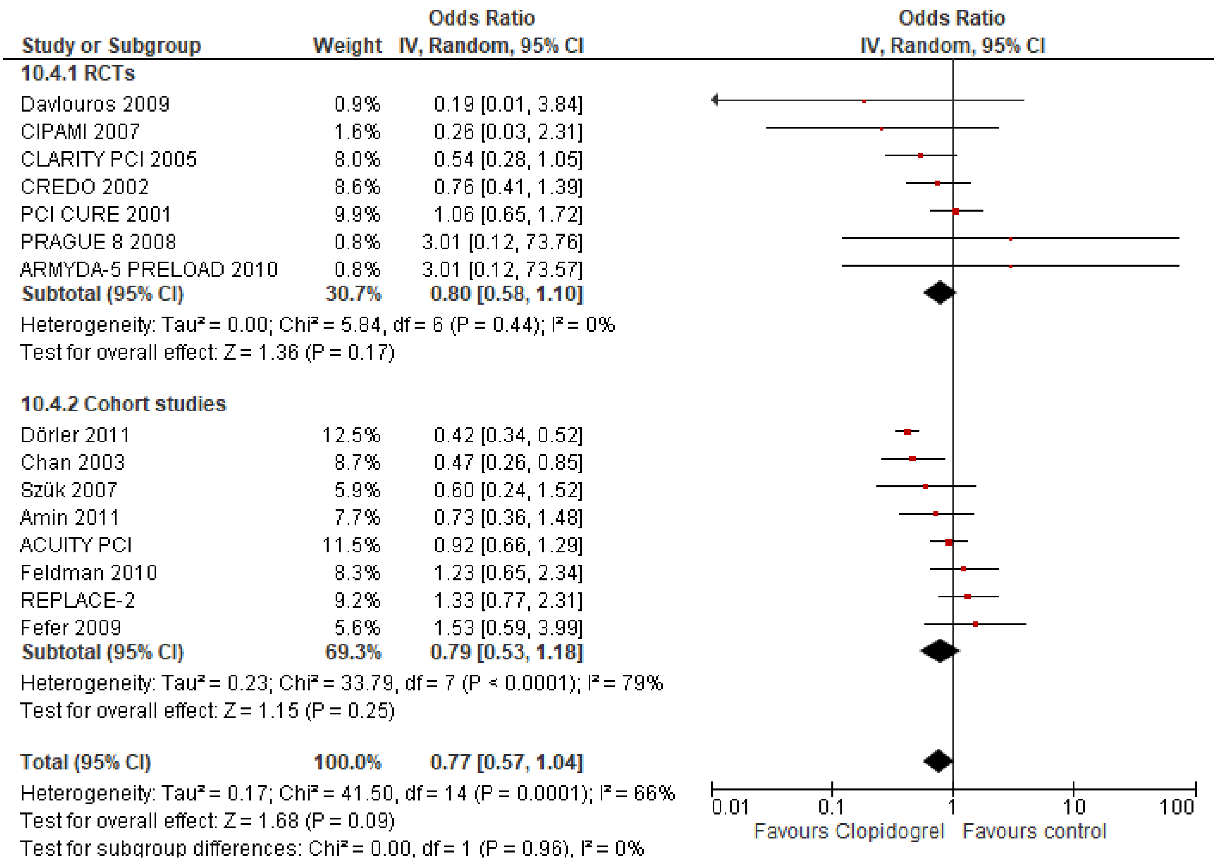

Figure S12: Bellemain-Appaix 2012; Intervention/ Exposure: Clopidogrel pretreatment for percutaneous coronary intervention; Outcome: All-cause mortality

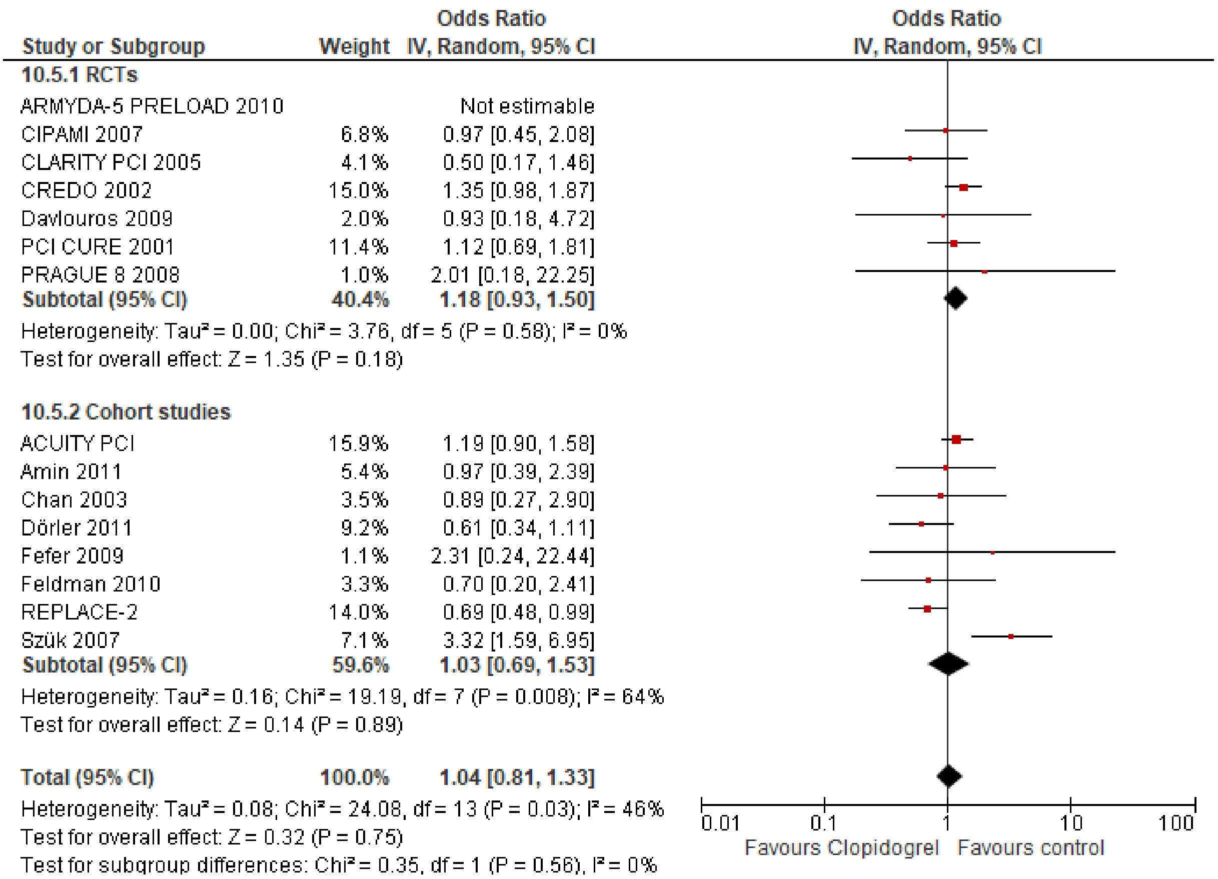

Figure S13: Bellemain-Appaix 2012; Intervention/ Exposure: Clopidogrel pretreatment for percutaneous coronary intervention; Outcome: Major bleeding

Study or Subgroup	Weight	Odds Ratio IV, Random, 95% CI
**10.6.1 RCTs**		
ARMYDA-5 PRELOAD 2010	3.7%	1.19 [0.62, 2.31]
CIPAMI 2007	1.8%	0.42 [0.14, 1.21]
CLARITY PCI 2005	6.0%	0.57 [0.37, 0.88]
CREDO 2002	8.2%	0.71 [0.53, 0.95]
Davlouros 2009	2.8%	1.09 [0.49, 2.42]
PCI CURE 2001	9.8%	0.81 [0.67, 0.98]
PRAGUE 8 2008	3.7%	0.89 [0.46, 1.74]
**Subtotal (95% CI)**	**36.0%**	**0.77 [0.66, 0.89]**
Heterogeneity: Tau² = 0.00; Chi² = 6.25, df = 6 (P = 0.40); I² = 4%		
Test for overall effect: Z = 3.48 (P = 0.0005)		
**10.6.2 Cohort studies**		
ACUITY PCI	10.3%	1.10 [0.94, 1.29]
Amin 2011	7.7%	0.92 [0.66, 1.27]
Chan 2003	8.3%	0.71 [0.53, 0.95]
Dörler 2011	9.9%	0.57 [0.47, 0.69]
Fefer 2009	5.7%	0.54 [0.34, 0.86]
Feldman 2010	5.7%	1.18 [0.75, 1.87]
REPLACE-2	9.2%	0.72 [0.57, 0.90]
Szük 2007	7.1%	0.57 [0.40, 0.82]
**Subtotal (95% CI)**	**64.0%**	**0.76 [0.60, 0.95]**
Heterogeneity: Tau² = 0.08; Chi² = 39.46, df = 7 (P < 0.00001); I² = 82%		
Test for overall effect: Z = 2.39 (P = 0.02)		
**Total (95% CI)**	**100.0%**	**0.76 [0.65, 0.89]**
Heterogeneity: Tau² = 0.05; Chi² = 45.80, df = 14 (P < 0.0001); I² = 69%		
Test for overall effect: Z = 3.45 (P = 0.0006)		
Test for subgroup differences: Chi² = 0.01, df = 1 (P = 0.92), I² = 0%		

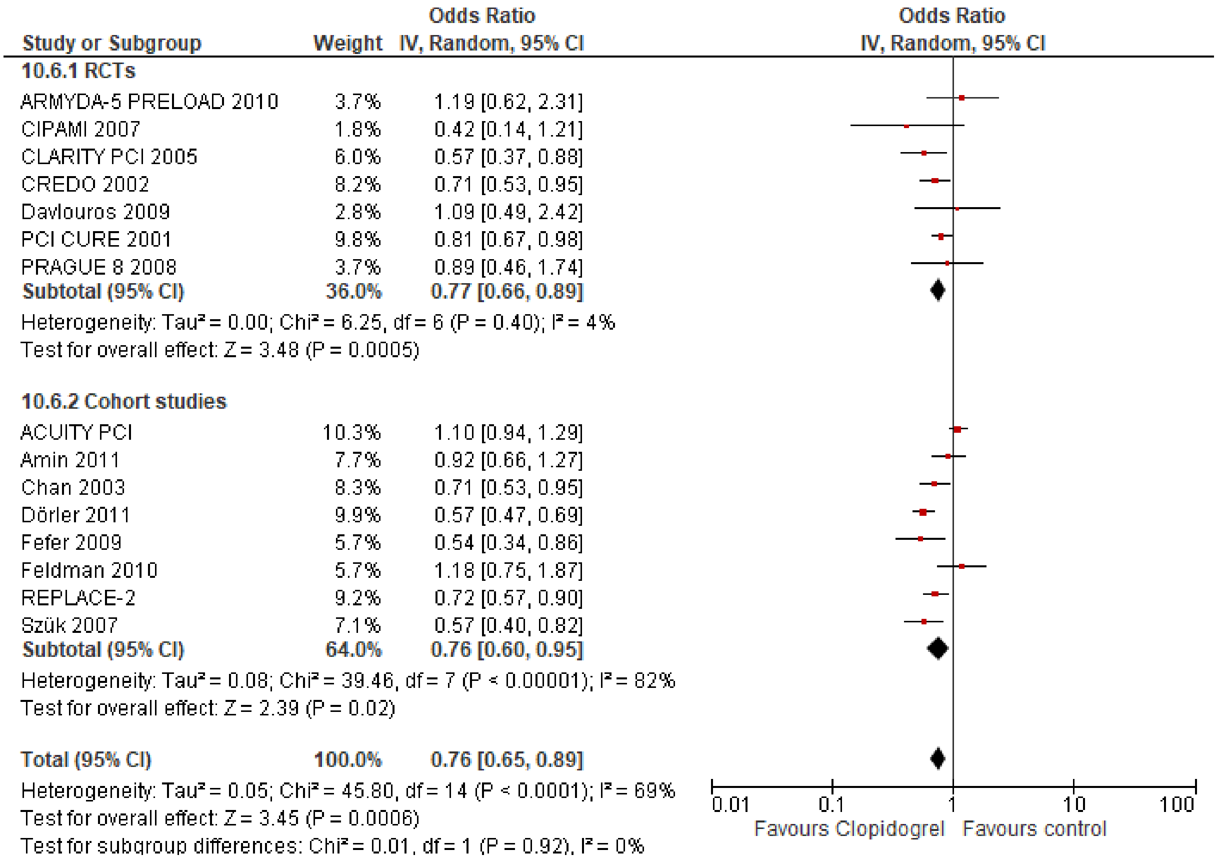

Figure S14: Bellemain-Appaix 2012; Intervention/ Exposure: Clopidogrel pretreatment for percutaneous coronary intervention; Outcome: Coronary heart disease

Study or Subgroup	Weight	Odds Ratio IV, Random, 95% CI
**11.4.1 RCTs**		
ACCOAST	3.6%	0.78 [0.31, 1.99]
CREDO	0.4%	0.11 [0.01, 2.09]
CURE	62.8%	0.92 [0.80, 1.07]
**Subtotal (95% CI)**	**66.8%**	**0.90 [0.71, 1.14]**
Heterogeneity: Tau² = 0.01; Chi² = 2.09, df = 2 (P = 0.35); I² = 5%		
Test for overall effect: Z = 0.87 (P = 0.39)		
**11.4.2 Cohort studies**		
ACUITY (cohort analysis)	29.1%	0.98 [0.74, 1.30]
Assali	1.0%	0.17 [0.03, 1.07]
Chan	2.1%	0.47 [0.14, 1.59]
Feldman	1.0%	0.82 [0.14, 4.92]
**Subtotal (95% CI)**	**33.2%**	**0.69 [0.35, 1.32]**
Heterogeneity: Tau² = 0.17; Chi² = 4.60, df = 3 (P = 0.20); I² = 35%		
Test for overall effect: Z = 1.13 (P = 0.26)		
**Total (95% CI)**	**100.0%**	**0.90 [0.75, 1.07]**
Heterogeneity: Tau² = 0.01; Chi² = 6.70, df = 6 (P = 0.35); I² = 10%		
Test for overall effect: Z = 1.18 (P = 0.24)		
Test for subgroup differences: Chi² = 0.59, df = 1 (P = 0.44), I² = 0%		

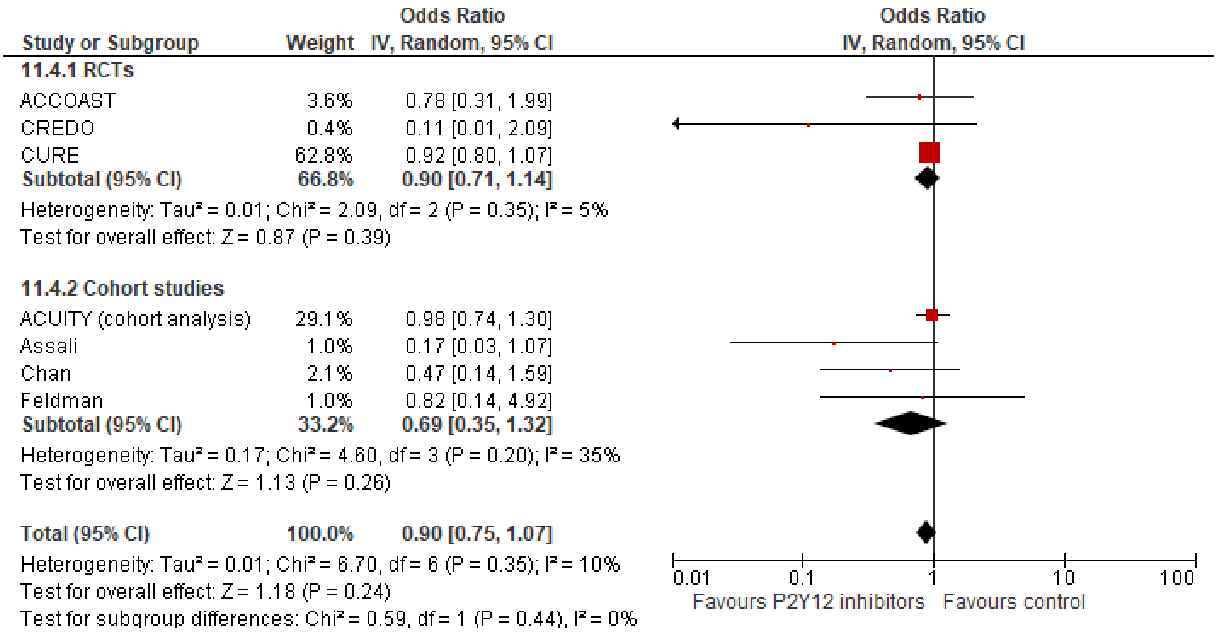

Figure S15: Bellemain-Appaix 2014; Intervention/ Exposure: P2Y12 inhibitor pretreatment in non-ST elevation acute coronary syndrome; Outcome: All-cause mortality

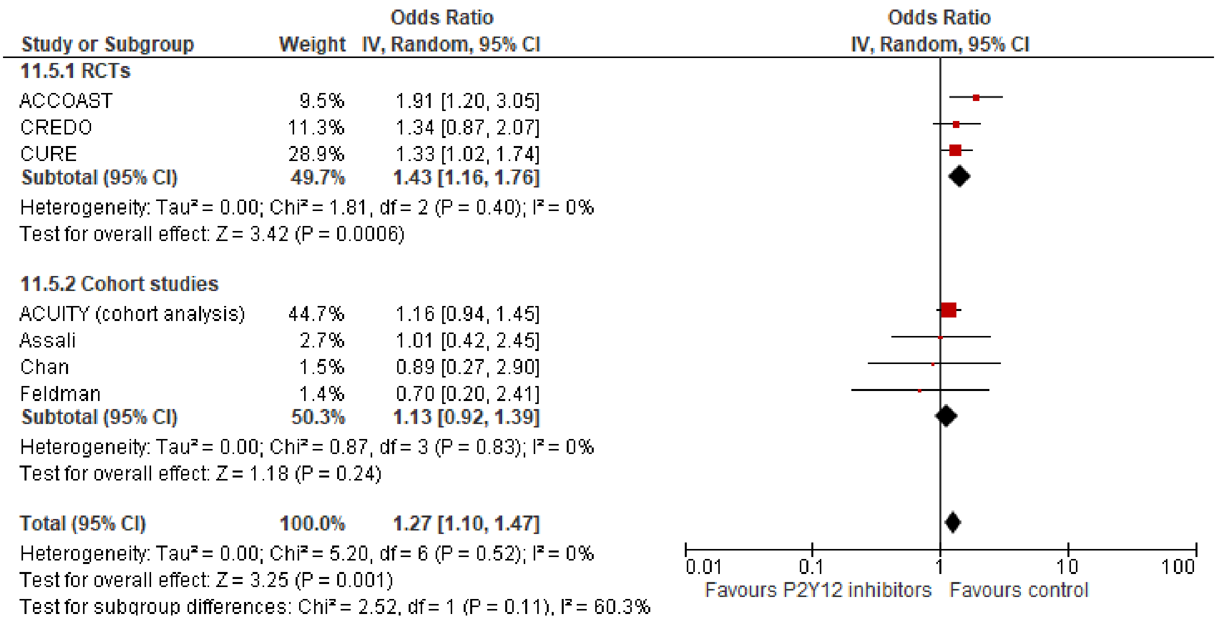

Figure S16: Bellemain-Appaix 2014; Intervention/ Exposure: P2Y12 inhibitor pretreatment in non-ST elevation acute coronary syndrome; Outcome: Major bleeding

Study or Subgroup	Weight	Odds Ratio IV, Random, 95% CI
**11.6.1 RCTs**		
ACCOAST	19.9%	1.02 [0.83, 1.26]
CREDO	11.8%	0.80 [0.57, 1.14]
CURE	23.3%	0.79 [0.67, 0.93]
**Subtotal (95% CI)**	**55.0%**	**0.87 [0.73, 1.04]**
Heterogeneity: Tau² = 0.01; Chi² = 3.84, df = 2 (P = 0.15); I² = 48%		
Test for overall effect: Z = 1.51 (P = 0.13)		
**11.6.2 Cohort studies**		
ACUITY (cohort analysis)	23.4%	0.88 [0.75, 1.03]
Assali	2.6%	0.36 [0.15, 0.88]
Chan	10.8%	0.61 [0.42, 0.89]
Feldman	8.2%	1.18 [0.75, 1.87]
**Subtotal (95% CI)**	**45.0%**	**0.78 [0.56, 1.08]**
Heterogeneity: Tau² = 0.07; Chi² = 8.65, df = 3 (P = 0.03); I² = 65%		
Test for overall effect: Z = 1.49 (P = 0.14)		
**Total (95% CI)**	**100.0%**	**0.84 [0.72, 0.98]**
Heterogeneity: Tau² = 0.02; Chi² = 12.59, df = 6 (P = 0.05); I² = 52%		
Test for overall effect: Z = 2.24 (P = 0.02)		
Test for subgroup differences: Chi² = 0.35, df = 1 (P = 0.55), I² = 0%		

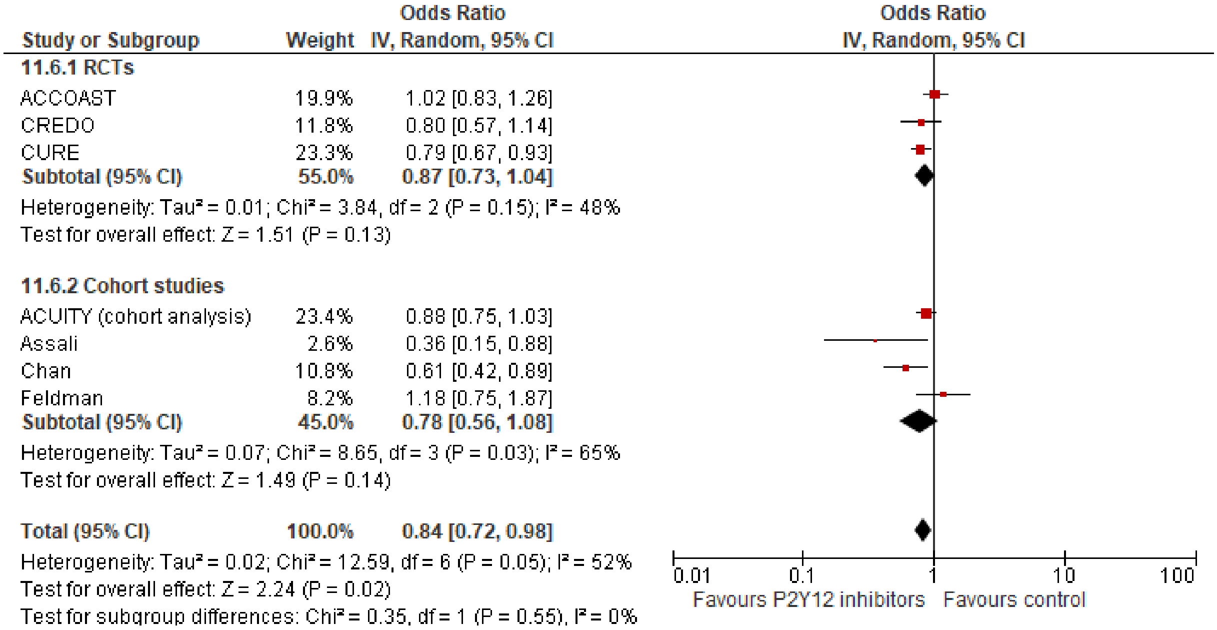

Figure S17: Bellemain-Appaix 2014; Intervention/ Exposure: P2Y12 inhibitor pretreatment in non-ST elevation acute coronary syndrome; Outcome: Main composite ischemic endpoint

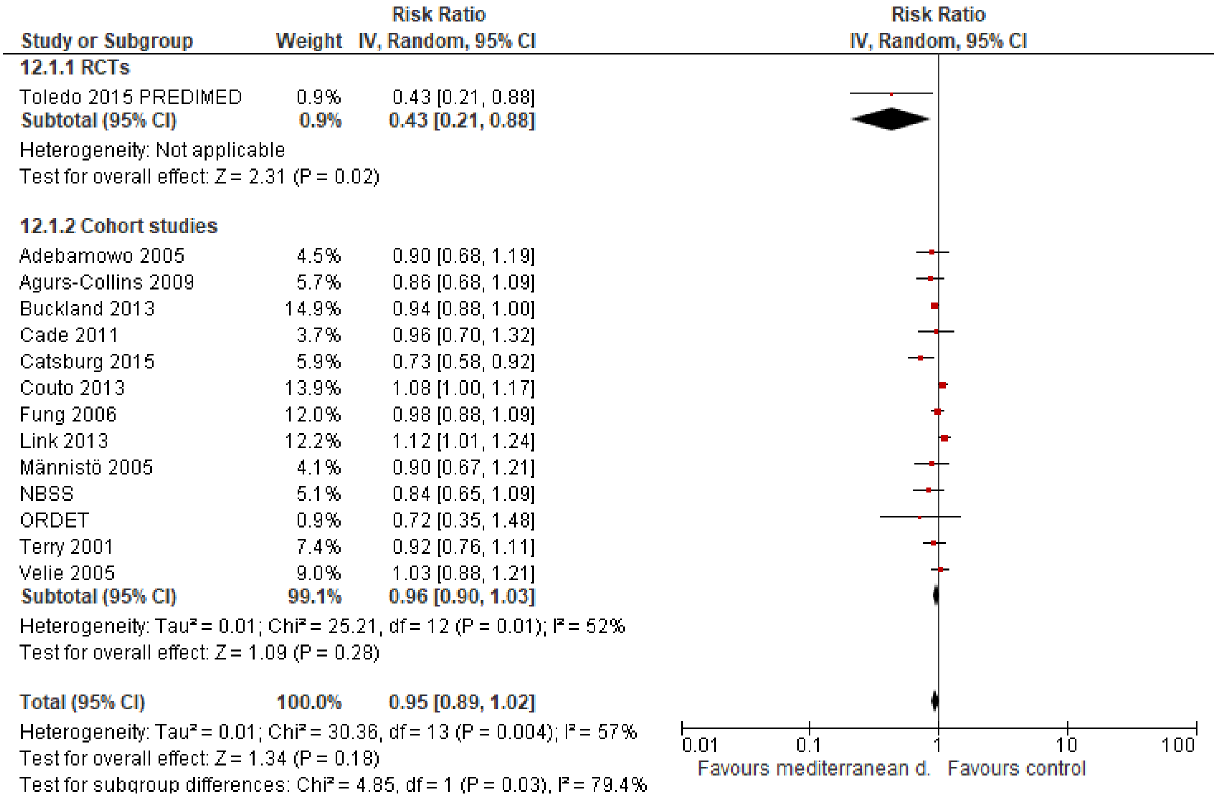

Figure S18: Bloomfield 2016; Intervention/ Exposure: Mediterranean diet; Outcome: Breast cancer

Study or Subgroup	Weight	Risk Ratio IV, Random, 95% CI
**13.2.1 RCTs**		
Avenell 2004	0.5%	1.23 [0.51, 3.00]
Baron 1999	0.3%	0.29 [0.10, 0.87]
Bolton-Smith 2007	0.1%	0.98 [0.14, 6.76]
Bonnick 2007	0.7%	0.32 [0.15, 0.67]
Chapuy 1994	7.4%	0.83 [0.71, 0.97]
Chapuy 2002	2.4%	1.01 [0.70, 1.47]
Chevalley 1994	0.1%	0.50 [0.07, 3.38]
Dawson-Huges 1997	0.8%	0.46 [0.23, 0.90]
Grant 2005	8.5%	0.93 [0.82, 1.06]
Harwood 2004	0.3%	1.20 [0.38, 3.76]
Inkovaara 1983	0.1%	0.33 [0.04, 3.08]
Jackson 2006	12.1%	0.97 [0.92, 1.03]
Larsen 2004	5.7%	1.00 [0.82, 1.22]
Peacock 2000	0.6%	1.18 [0.52, 2.68]
Porthouse 2005	3.0%	0.96 [0.70, 1.33]
Prince 2006	4.7%	0.87 [0.69, 1.10]
Reid 1993	0.4%	0.59 [0.23, 1.54]
Reid 2006	5.4%	0.92 [0.75, 1.14]
Reid 2008	0.4%	0.56 [0.22, 1.40]
Riggs 1998	0.6%	0.90 [0.41, 1.96]
Salovaara 2010	3.4%	0.83 [0.62, 1.11]
Sambrook 2012	0.7%	0.72 [0.34, 1.54]
**Subtotal (95% CI)**	**58.0%**	**0.90 [0.83, 0.97]**
Heterogeneity: Tau² = 0.00; Chi² = 27.12, df = 21 (P = 0.17); I² = 23%		
Test for overall effect: Z = 2.83 (P = 0.005)		
**13.2.2 Cohort studies**		
Cumming 1997	10.4%	1.10 [1.00, 1.20]
Dargent-Molina 2008	8.0%	0.83 [0.72, 0.96]
Prentice 2013	10.9%	0.99 [0.91, 1.07]
Prentice 2013	11.9%	1.07 [1.01, 1.14]
Samieri 2013	0.2%	2.20 [0.50, 9.68]
Thomas-John 2009	0.6%	1.70 [0.77, 3.79]
**Subtotal (95% CI)**	**42.0%**	**1.02 [0.93, 1.12]**
Heterogeneity: Tau² = 0.01; Chi² = 15.82, df = 5 (P = 0.007); I² = 68%		
Test for overall effect: Z = 0.34 (P = 0.74)		
**Total (95% CI)**	**100.0%**	**0.94 [0.88, 1.00]**
Heterogeneity: Tau² = 0.01; Chi² = 54.07, df = 27 (P = 0.001); I² = 50%		
Test for overall effect: Z = 1.88 (P = 0.06)		
Test for subgroup differences: Chi² = 4.09, df = 1 (P = 0.04), I² = 75.5%		

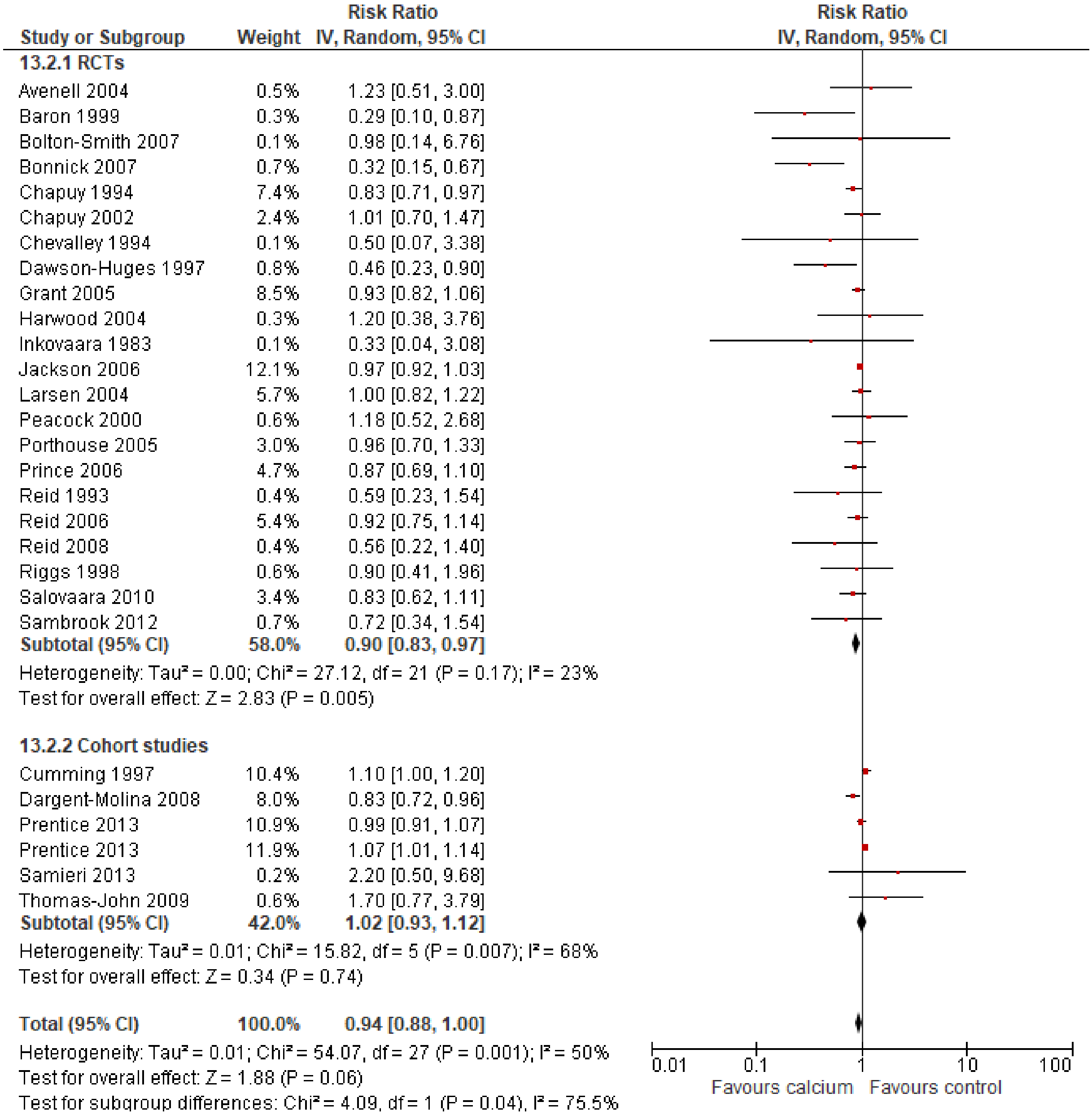

Figure S19: Bolland 2015; Intervention/ Exposure: High calcium; Outcome: All fractures

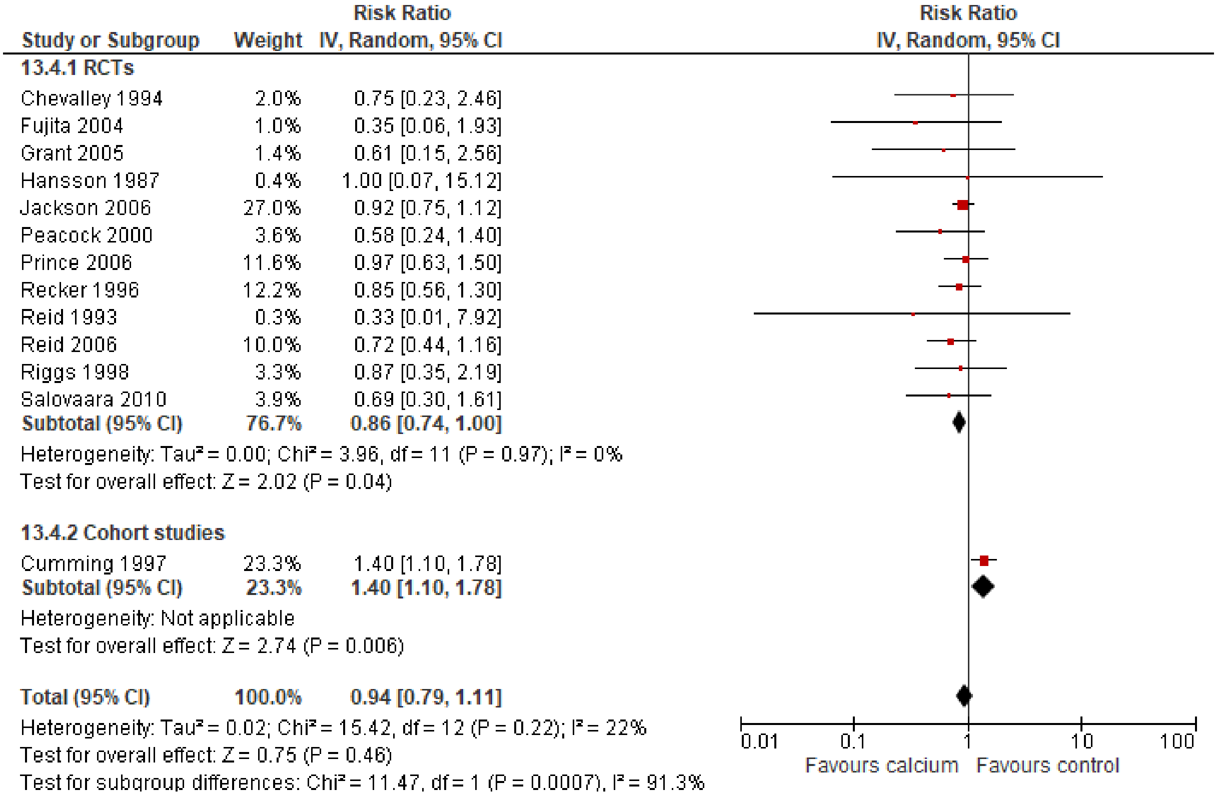

Figure S20: Bolland 2015; Intervention/ Exposure: High calcium; Outcome: Vertebral fracture

Study or Subgroup	Weight	Risk Ratio IV, Random, 95% CI
**13.5.1 RCTs**		
Avenell 2004	0.4%	3.28 [0.35, 30.75]
Baron 1999	0.2%	3.01 [0.12, 73.77]
Chapuy 1994	10.6%	0.77 [0.62, 0.96]
Chapuy 2002	4.5%	0.64 [0.37, 1.10]
Dawson-Huges 1997	0.2%	0.36 [0.01, 8.78]
Grant 2005	9.0%	1.10 [0.83, 1.47]
Harwood 2004	0.3%	1.00 [0.06, 15.70]
Jackson 2006	11.0%	0.88 [0.72, 1.07]
Porthouse 2005	2.3%	0.71 [0.31, 1.64]
Prince 2006	1.7%	1.83 [0.68, 4.93]
Reid 1993	0.2%	0.20 [0.01, 4.03]
Reid 2006	1.7%	3.43 [1.27, 9.26]
Salovaara 2010	0.6%	2.00 [0.37, 10.88]
**Subtotal (95% CI)**	**42.8%**	**0.95 [0.76, 1.18]**
Heterogeneity: Tau² = 0.04; Chi² = 18.63, df = 12 (P = 0.10); I² = 36%		
Test for overall effect: Z = 0.48 (P = 0.63)		
**13.5.2 Cohort studies**		
Cumming 1997	8.5%	1.50 [1.10, 2.02]
Feskanich 2003	11.3%	1.01 [0.84, 1.23]
Koh 2009 - men	2.1%	0.84 [0.35, 2.05]
Koh 2009 - women	7.7%	1.43 [1.01, 2.01]
Owusu 1997	1.5%	0.60 [0.20, 1.75]
Paganini-Hill 1991 - men	2.2%	1.16 [0.49, 2.78]
Paganini-Hill 1991 - women	5.6%	1.45 [0.92, 2.30]
Prentice 2013	8.0%	0.82 [0.59, 1.14]
Prentice 2013	10.3%	0.88 [0.70, 1.11]
**Subtotal (95% CI)**	**57.2%**	**1.09 [0.91, 1.30]**
Heterogeneity: Tau² = 0.03; Chi² = 16.15, df = 8 (P = 0.04); I² = 50%		
Test for overall effect: Z = 0.90 (P = 0.37)		
**Total (95% CI)**	**100.0%**	**1.02 [0.89, 1.18]**
Heterogeneity: Tau² = 0.04; Chi² = 38.77, df = 21 (P = 0.01); I² = 46%		
Test for overall effect: Z = 0.34 (P = 0.74)		
Test for subgroup differences: Chi² = 0.90, df = 1 (P = 0.34), I² = 0%		

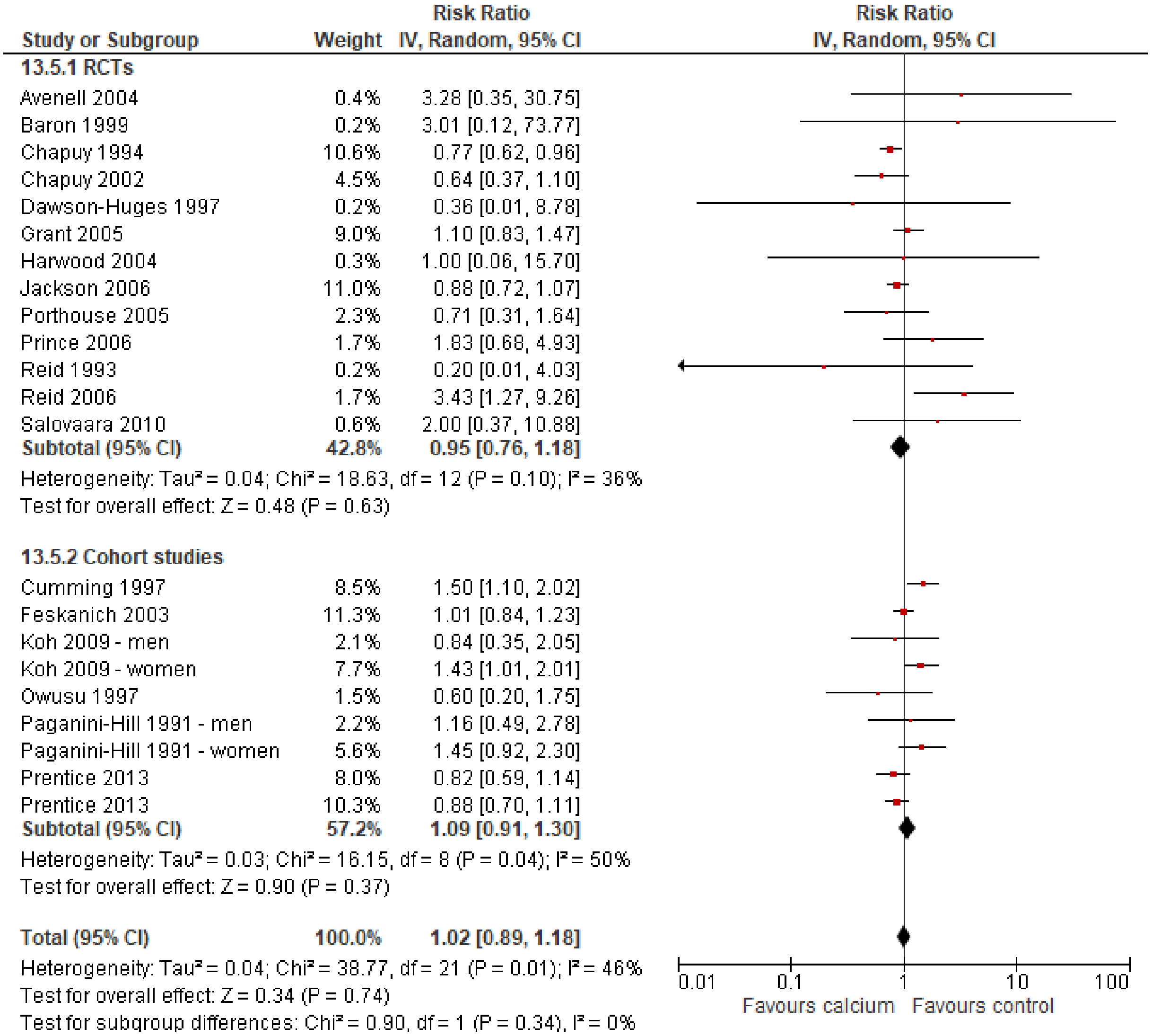

Figure S21: Bolland 2015; Intervention/ Exposure: High calcium; Outcome: Hip fracture

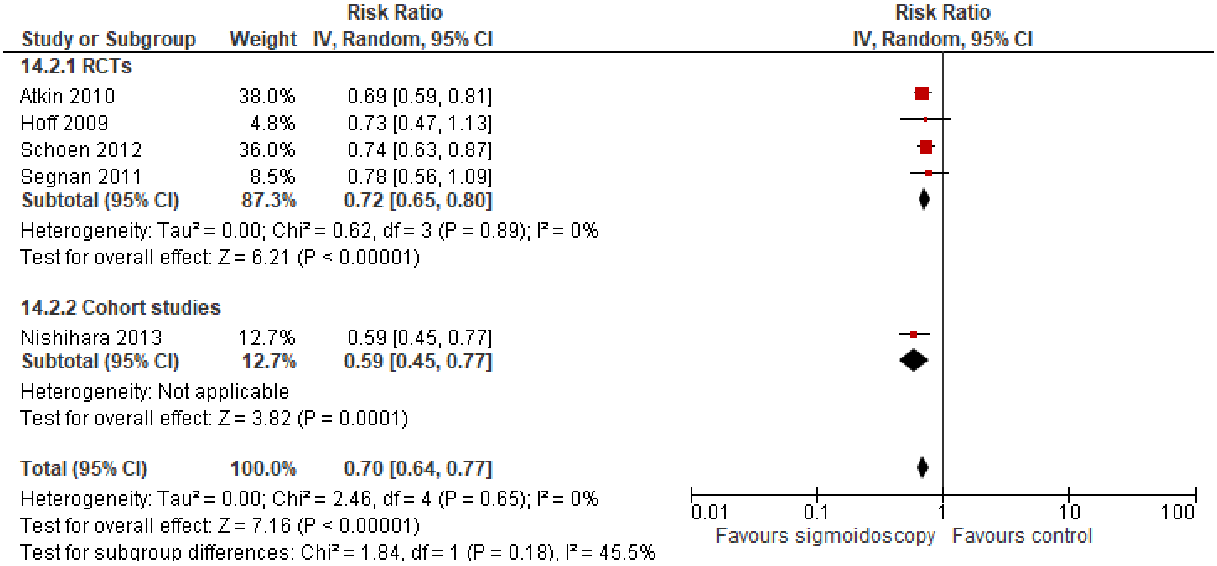

Figure S22: Brenner 2014; Intervention/ Exposure: Sigmoidoscopy; Outcome: Colorectal cancer mortality

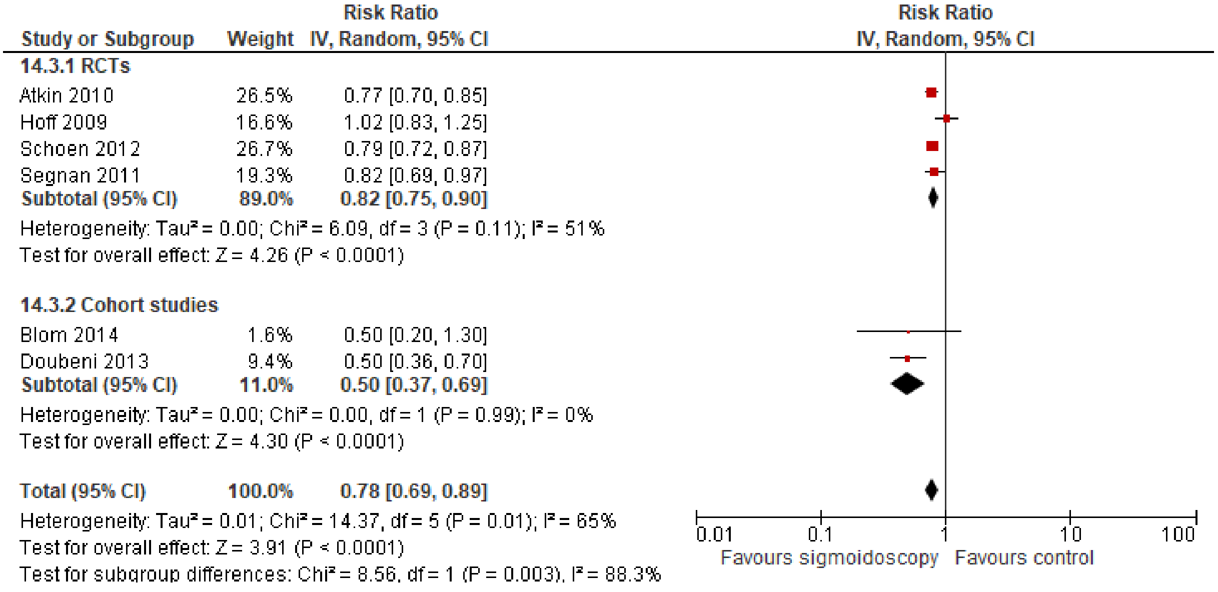

Figure S23: Brenner 2014; Intervention/ Exposure: Sigmoidoscopy; Outcome: Colorectal cancer incidence

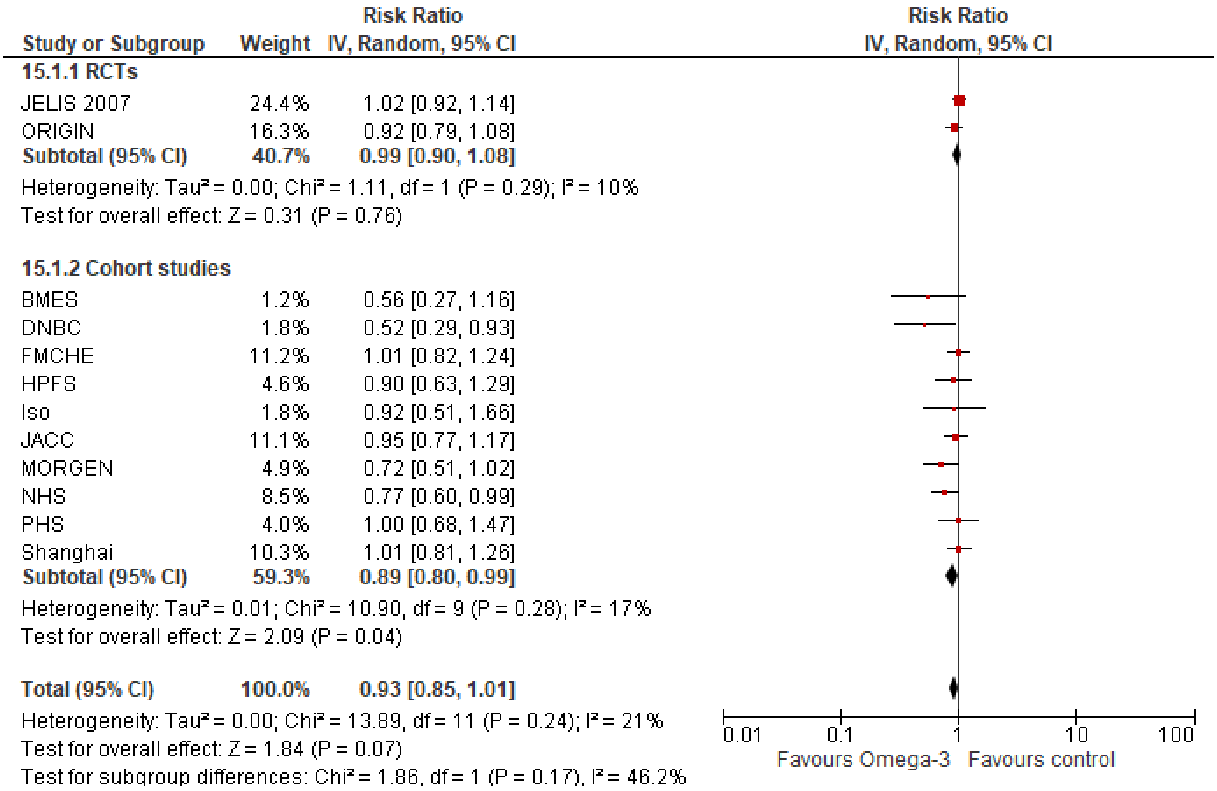

Figure S24: Chowdhury 2012; Intervention/ Exposure: High omega-3; Outcome: Cerebrovascular disease

Study or Subgroup	Weight	Risk Ratio IV, Random, 95% CI
**16.4.1 RCTs**		
Alpha Omega	8.0%	0.92 [0.66, 1.28]
Borchgrevnik	3.4%	1.18 [0.66, 2.11]
LDHS	0.7%	0.20 [0.05, 0.82]
NorVegExp	9.4%	1.13 [0.85, 1.51]
**Subtotal (95% CI)**	**21.4%**	**0.97 [0.69, 1.36]**
Heterogeneity: Tau² = 0.06; Chi² = 6.25, df = 3 (P = 0.10); I² = 52%		
Test for overall effect: Z = 0.18 (P = 0.86)		
**16.4.2 Cohort studies**		
ATBC	16.2%	0.97 [0.84, 1.12]
Glostrup	7.9%	0.89 [0.64, 1.24]
HPFS	14.0%	0.83 [0.69, 1.00]
KIHD	13.1%	1.20 [0.98, 1.47]
MRFIT	6.5%	0.71 [0.48, 1.04]
NHS	18.2%	1.09 [0.98, 1.21]
Zutphen study	2.7%	1.68 [0.86, 3.28]
**Subtotal (95% CI)**	**78.6%**	**0.99 [0.87, 1.13]**
Heterogeneity: Tau² = 0.02; Chi² = 15.41, df = 6 (P = 0.02); I² = 61%		
Test for overall effect: Z = 0.17 (P = 0.86)		
**Total (95% CI)**	**100.0%**	**0.99 [0.88, 1.11]**
Heterogeneity: Tau² = 0.02; Chi² = 21.67, df = 10 (P = 0.02); I² = 54%		
Test for overall effect: Z = 0.15 (P = 0.88)		
Test for subgroup differences: Chi² = 0.01, df = 1 (P = 0.92), I² = 0%		

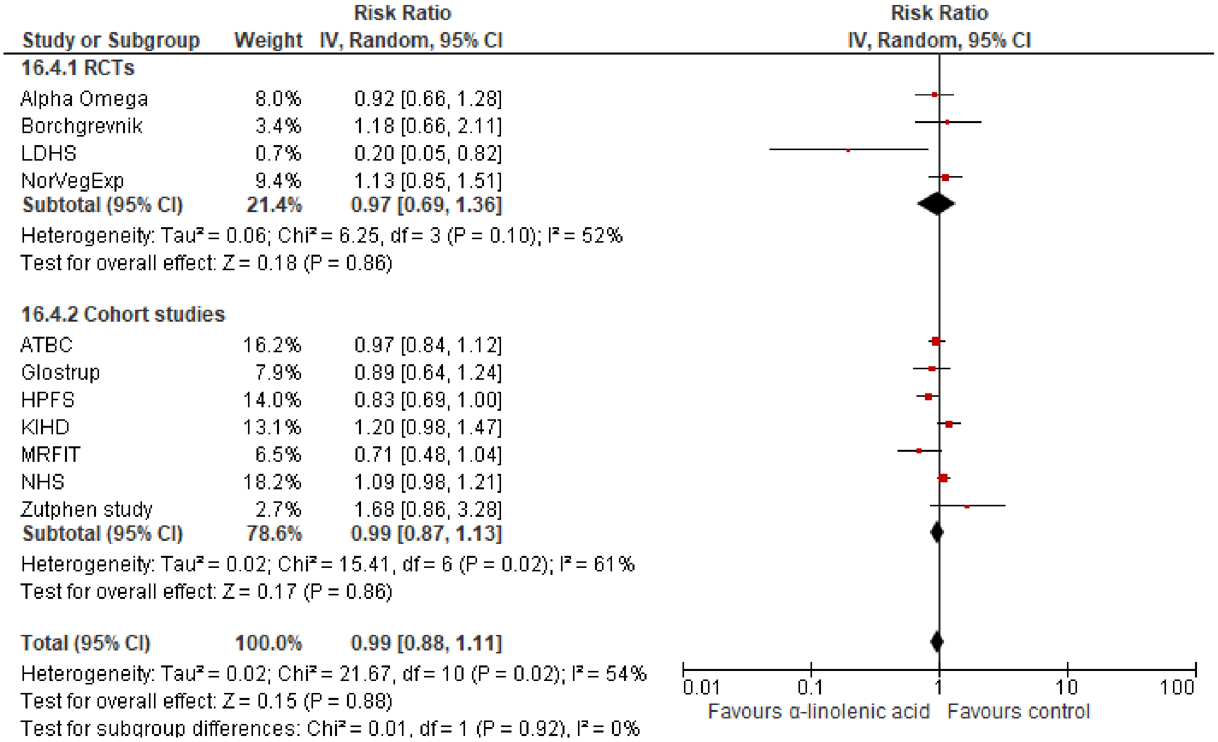

Figure S25: Chowdhury 2014a; Intervention/ Exposure: High α-linolenic acid; Outcome: Coronary heart disease

Study or Subgroup	Weight	Risk Ratio IV, Random, 95% CI
**16.3.1 RCTs**		
Alpha Omega	2.9%	0.95 [0.68, 1.34]
GISSI-HF	3.7%	0.82 [0.63, 1.07]
GISSI-P	5.6%	0.86 [0.75, 0.99]
Grundt	1.9%	1.42 [0.89, 2.27]
HARP	0.9%	0.66 [0.31, 1.40]
JELIS	3.1%	0.76 [0.56, 1.04]
Leng	1.0%	0.78 [0.38, 1.58]
Nilsen	1.1%	1.43 [0.74, 2.76]
Omega	2.1%	1.09 [0.70, 1.69]
OPACH	0.7%	0.40 [0.17, 0.96]
OPERA	0.7%	1.00 [0.41, 2.42]
ORIGIN	5.2%	1.09 [0.93, 1.28]
Raitt	0.1%	0.33 [0.03, 3.19]
RPS	5.2%	0.96 [0.82, 1.12]
SOFA	0.1%	0.33 [0.02, 4.45]
SUFOLOM3	2.4%	0.96 [0.65, 1.42]
von Schacky	1.6%	1.10 [0.66, 1.84]
**Subtotal (95% CI)**	**38.1%**	**0.94 [0.86, 1.03]**
Heterogeneity: Tau² = 0.01; Chi² = 19.32, df = 16 (P = 0.25); I² = 17%		
Test for overall effect: Z = 1.35 (P = 0.18)		
**16.3.2 Cohort studies**		
ATBC	5.6%	1.12 [0.98, 1.28]
CHS	5.9%	0.86 [0.77, 0.96]
DNBC	1.1%	0.35 [0.18, 0.68]
FCHES	4.4%	1.05 [0.85, 1.30]
Glostrup	3.5%	0.69 [0.52, 0.92]
JACC	6.0%	1.02 [0.92, 1.13]
JHPC	3.3%	0.55 [0.41, 0.74]
KIHD	4.9%	1.10 [0.92, 1.32]
MALMO	5.0%	1.02 [0.86, 1.21]
MORGEN	3.7%	0.90 [0.69, 1.17]
NHS	5.4%	0.73 [0.63, 0.85]
NHSS	3.9%	1.00 [0.78, 1.28]
SANG	1.8%	0.52 [0.32, 0.85]
TAKA	1.9%	0.89 [0.56, 1.41]
WENBIT	3.6%	0.96 [0.73, 1.26]
Zutphen study	1.9%	0.64 [0.40, 1.02]
**Subtotal (95% CI)**	**61.9%**	**0.87 [0.78, 0.97]**
Heterogeneity: Tau² = 0.03; Chi² = 61.81, df = 15 (P < 0.00001); I² = 76%		
Test for overall effect: Z = 2.60 (P = 0.009)		
**Total (95% CI)**	**100.0%**	**0.90 [0.83, 0.97]**
Heterogeneity: Tau² = 0.02; Chi² = 81.28, df = 32 (P < 0.00001); I² = 61%		
Test for overall effect: Z = 2.78 (P = 0.005)		
Test for subgroup differences: Chi² = 1.28, df = 1 (P = 0.26), I² = 22.1%		

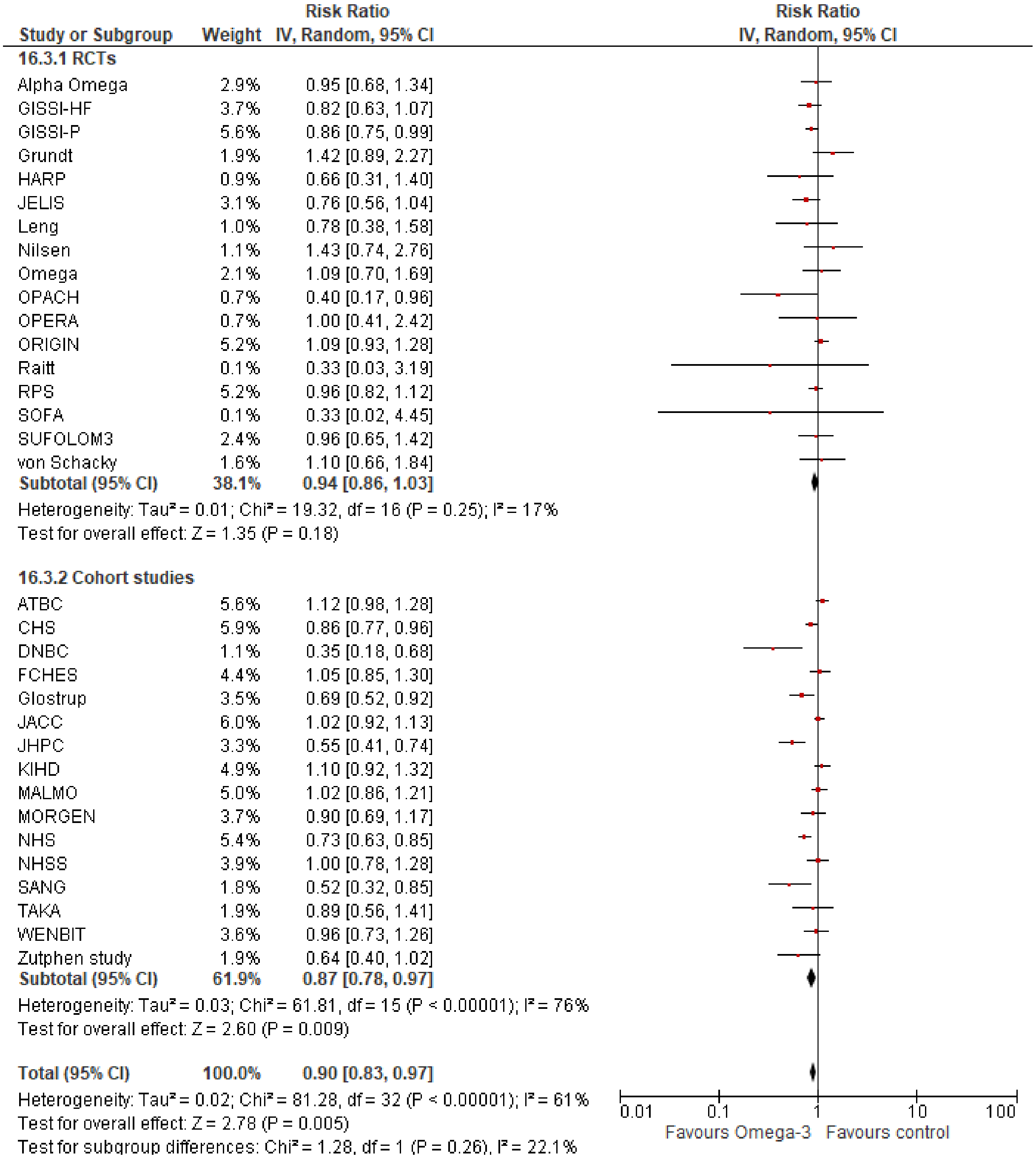

Figure S26: Chowdhury 2014a; Intervention/ Exposure: High omega-3; Outcome: Coronary heart disease

Study or Subgroup	Weight	Risk Ratio IV, Random, 95% CI
**16.2.1 RCTs**		
DART	6.9%	0.91 [0.72, 1.15]
FMHS	2.7%	0.59 [0.37, 0.93]
LA Veteran	4.6%	0.74 [0.53, 1.03]
MCS	6.7%	1.08 [0.84, 1.37]
MRC	4.2%	0.86 [0.61, 1.22]
OSLO	2.3%	0.56 [0.33, 0.94]
SDHS	2.3%	1.74 [1.04, 2.92]
STARS	0.3%	0.41 [0.09, 1.96]
**Subtotal (95% CI)**	**30.0%**	**0.86 [0.69, 1.07]**
Heterogeneity: Tau² = 0.05; Chi² = 17.20, df = 7 (P = 0.02); I² = 59%		
Test for overall effect: Z = 1.37 (P = 0.17)		
**16.2.2 Cohort studies**		
ATBC	11.4%	1.05 [0.92, 1.19]
Glostrup	5.2%	0.93 [0.69, 1.26]
HPFS	14.9%	0.97 [0.93, 1.02]
KIHD	8.3%	1.19 [0.98, 1.46]
MALMO	10.3%	1.08 [0.93, 1.26]
MORGEN	5.7%	0.95 [0.72, 1.26]
MRFIT	4.5%	0.70 [0.50, 0.98]
NHS	9.7%	0.82 [0.69, 0.96]
**Subtotal (95% CI)**	**70.0%**	**0.98 [0.90, 1.06]**
Heterogeneity: Tau² = 0.01; Chi² = 15.26, df = 7 (P = 0.03); I² = 54%		
Test for overall effect: Z = 0.50 (P = 0.62)		
**Total (95% CI)**	**100.0%**	**0.94 [0.87, 1.03]**
Heterogeneity: Tau² = 0.01; Chi² = 34.40, df = 15 (P = 0.003); I² = 56%		
Test for overall effect: Z = 1.33 (P = 0.18)		
Test for subgroup differences: Chi² = 1.21, df = 1 (P = 0.27), I² = 17.1%		

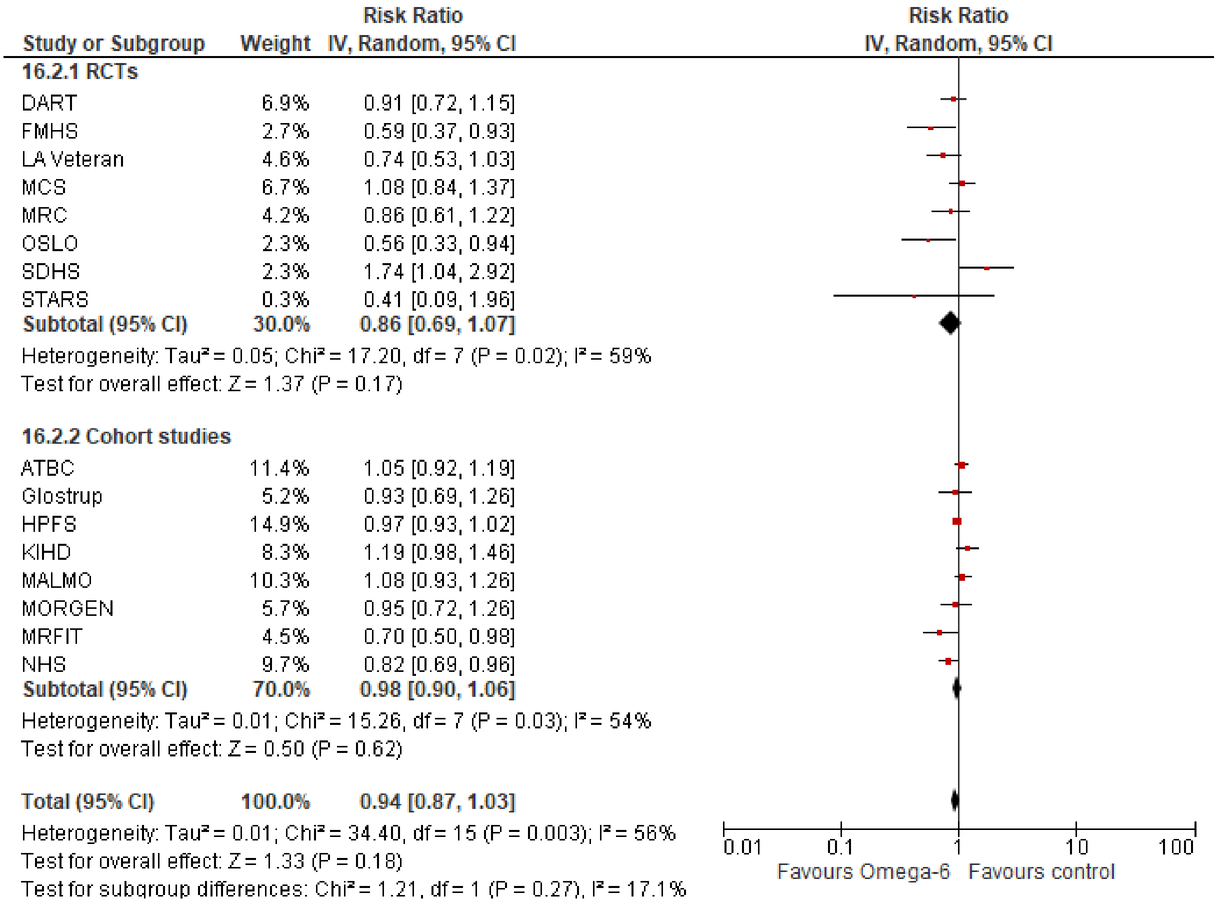

Figure S27: Chowdhury 2014a; Intervention/ Exposure: Omega-6; Outcome: Coronary heart disease

Study or Subgroup	Weight	Risk Ratio IV, Random, 95% CI
**19.2.1 RCTs**		
Avenell 2012	7.2%	1.09 [0.82, 1.45]
Lewis 2011	2.9%	0.94 [0.60, 1.49]
**Subtotal (95% CI)**	**10.1%**	**1.05 [0.82, 1.33]**
Heterogeneity: Tau² = 0.00; Chi² = 0.28, df = 1 (P = 0.60); I² = 0%		
Test for overall effect: Z = 0.37 (P = 0.71)		
**19.2.2 Cohort studies**		
Al-Delamy 2003	8.3%	1.04 [0.80, 1.35]
Bonthuls 2013	0.6%	1.94 [0.68, 5.53]
Bostick 1999	9.3%	0.82 [0.64, 1.05]
Chan 2003		Not estimable
Michaelsson 2013	58.2%	1.03 [0.96, 1.11]
Van Hemelrljck 2013	13.5%	0.88 [0.72, 1.08]
**Subtotal (95% CI)**	**89.9%**	**0.97 [0.86, 1.09]**
Heterogeneity: Tau² = 0.01; Chi² = 6.32, df = 4 (P = 0.18); I² = 37%		
Test for overall effect: Z = 0.52 (P = 0.60)		
**Total (95% CI)**	**100.0%**	**0.99 [0.92, 1.07]**
Heterogeneity: Tau² = 0.00; Chi² = 6.71, df = 6 (P = 0.35); I² = 11%		
Test for overall effect: Z = 0.17 (P = 0.86)		
Test for subgroup differences: Chi² = 0.31, df = 1 (P = 0.58), I² = 0%		

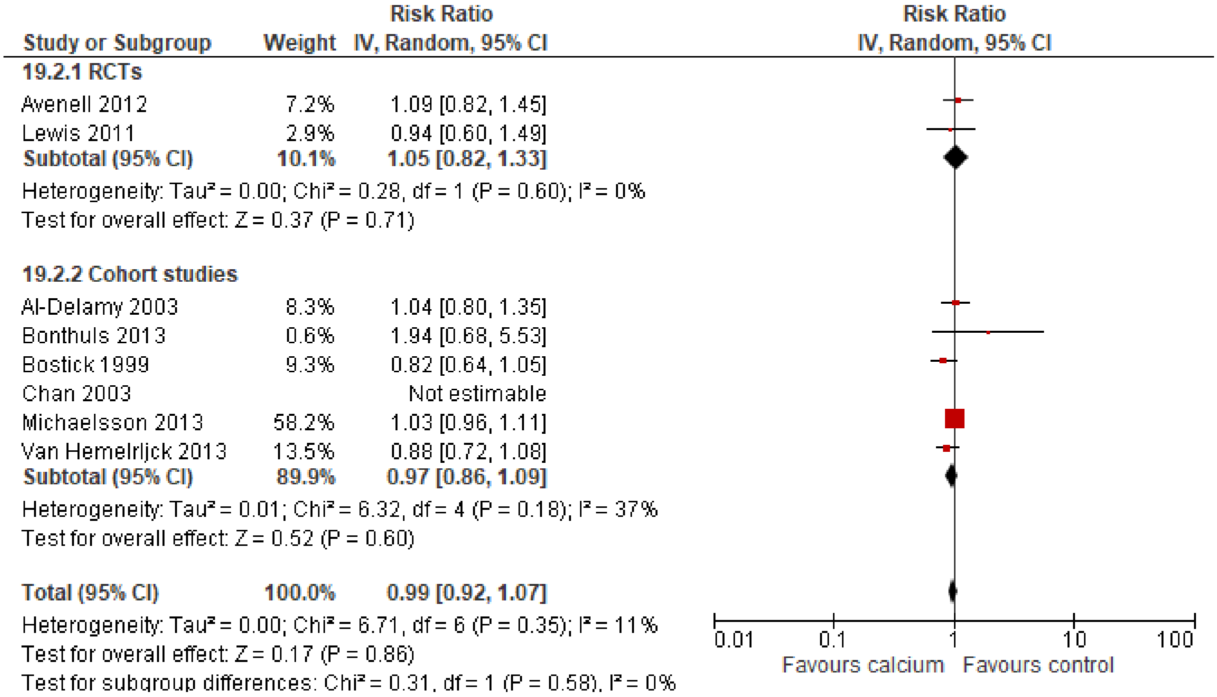

Figure S28: Chung 2016; Intervention/ Exposure: High calcium; Outcome: Cardiovascular mortality

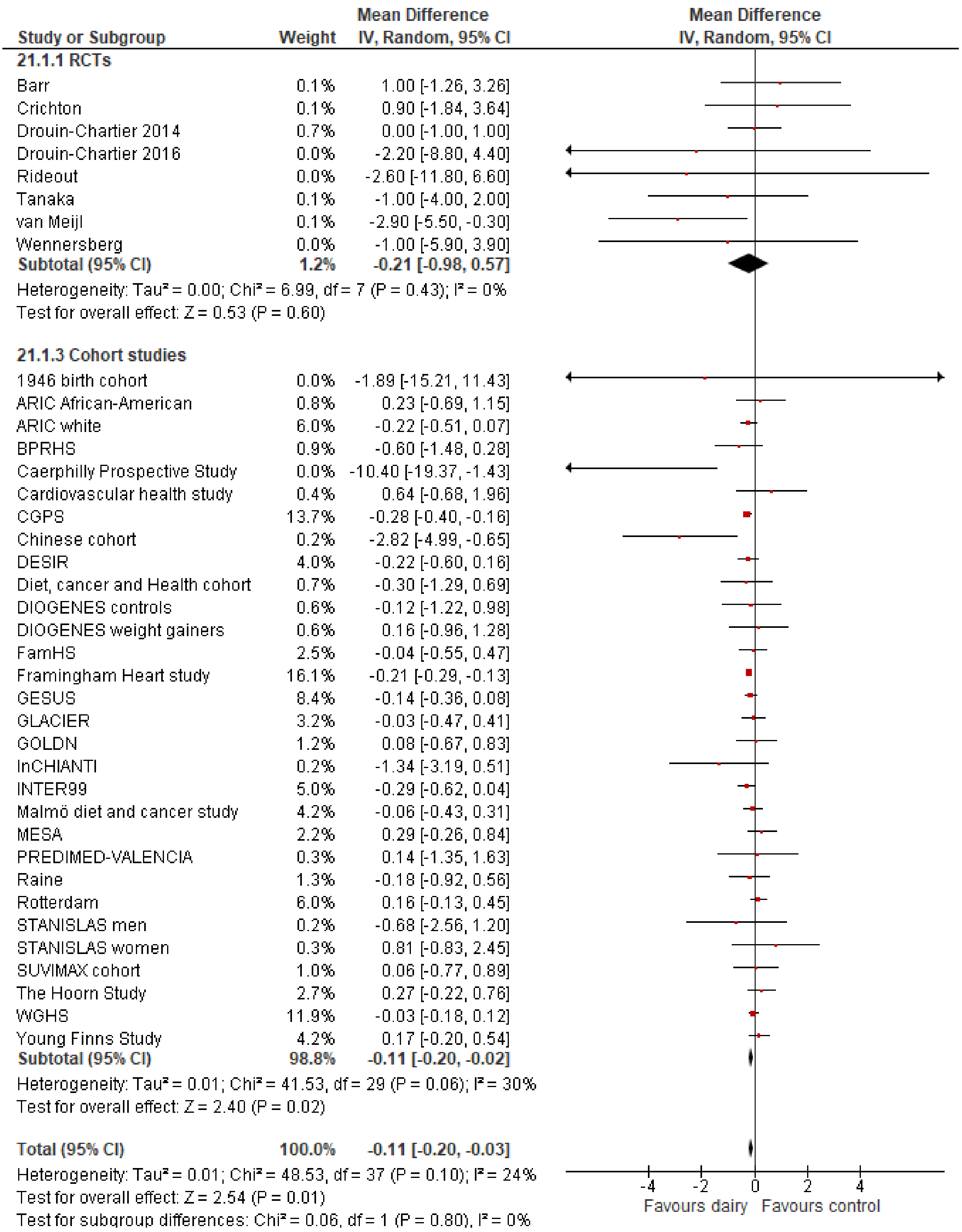

Figure S29: Ding 2017; Intervention/ Exposure: High dairy; Outcome: Systolic blood pressure

Study or Subgroup	Weight	Risk Ratio IV, Random, 95% CI
**24.5.1 RCTs**		
Donovan 2016	14.3%	0.91 [0.77, 1.08]
**Subtotal (95% CI)**	**14.3%**	**0.91 [0.77, 1.08]**
Heterogeneity: Not applicable		
Test for overall effect: Z = 1.09 (P = 0.27)		
**24.5.2 cohort studies**		
Barocas 2017	16.1%	1.41 [1.24, 1.59]
Chen 2017	14.8%	1.11 [0.95, 1.30]
Hoffman 2003	11.5%	1.50 [1.18, 1.91]
Schapira 2001	8.7%	1.10 [0.80, 1.52]
Siegel 2001	13.2%	1.37 [1.12, 1.66]
Smith 2009	12.6%	1.25 [1.01, 1.54]
Thong 2010	8.8%	1.46 [1.06, 2.01]
**Subtotal (95% CI)**	**85.7%**	**1.30 [1.19, 1.43]**
Heterogeneity: Tau² = 0.00; Chi² = 8.75, df = 6 (P = 0.19); I² = 31%		
Test for overall effect: Z = 5.60 (P < 0.00001)		
**Total (95% CI)**	**100.0%**	**1.24 [1.09, 1.41]**
Heterogeneity: Tau² = 0.02; Chi² = 23.60, df = 7 (P = 0.001); I² = 70%		
Test for overall effect: Z = 3.24 (P = 0.001)		
Test for subgroup differences: Chi² = 13.37, df = 1 (P = 0.0003), I² = 92.5%		

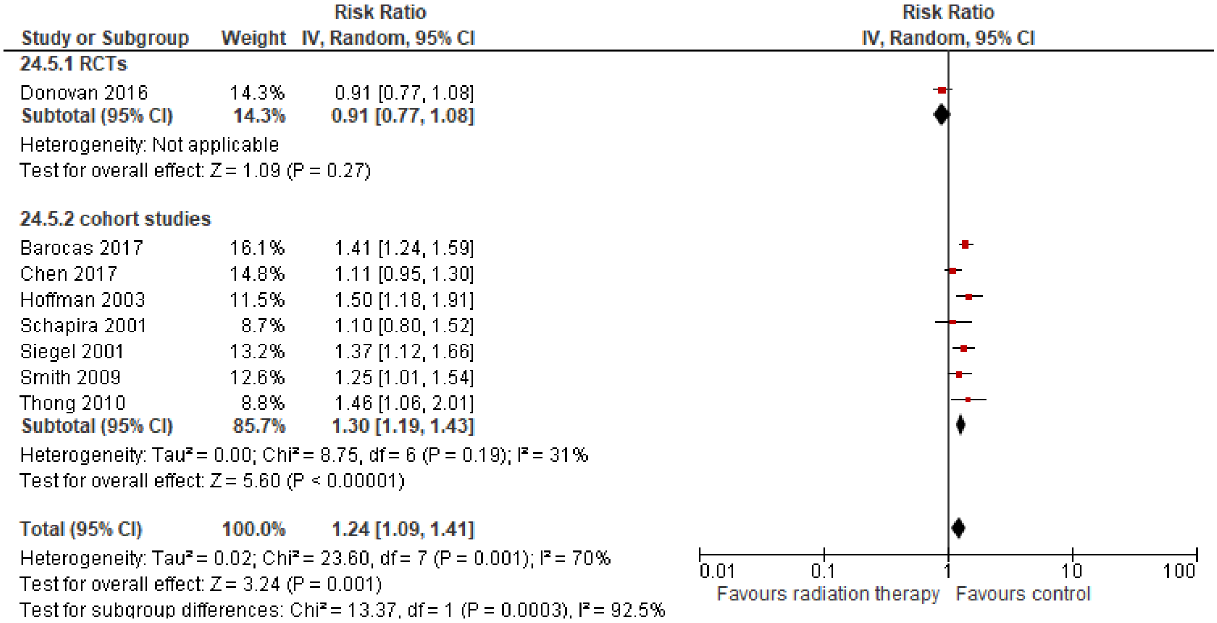

Figure S30: Fenton 2018; Intervention/ Exposure: Radiation therapy; Outcome: Erectile dysfunction

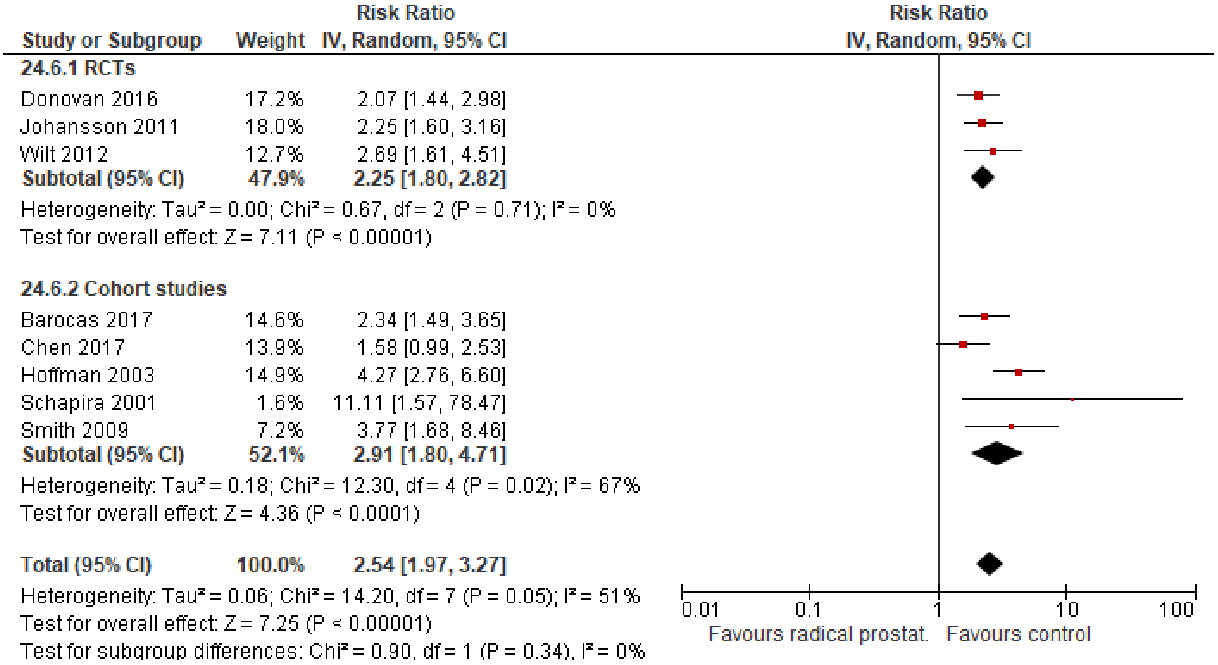

Figure S31: Fenton 2018; Intervention/ Exposure: Radical prostatectomy; Outcome: Urinary incontinence

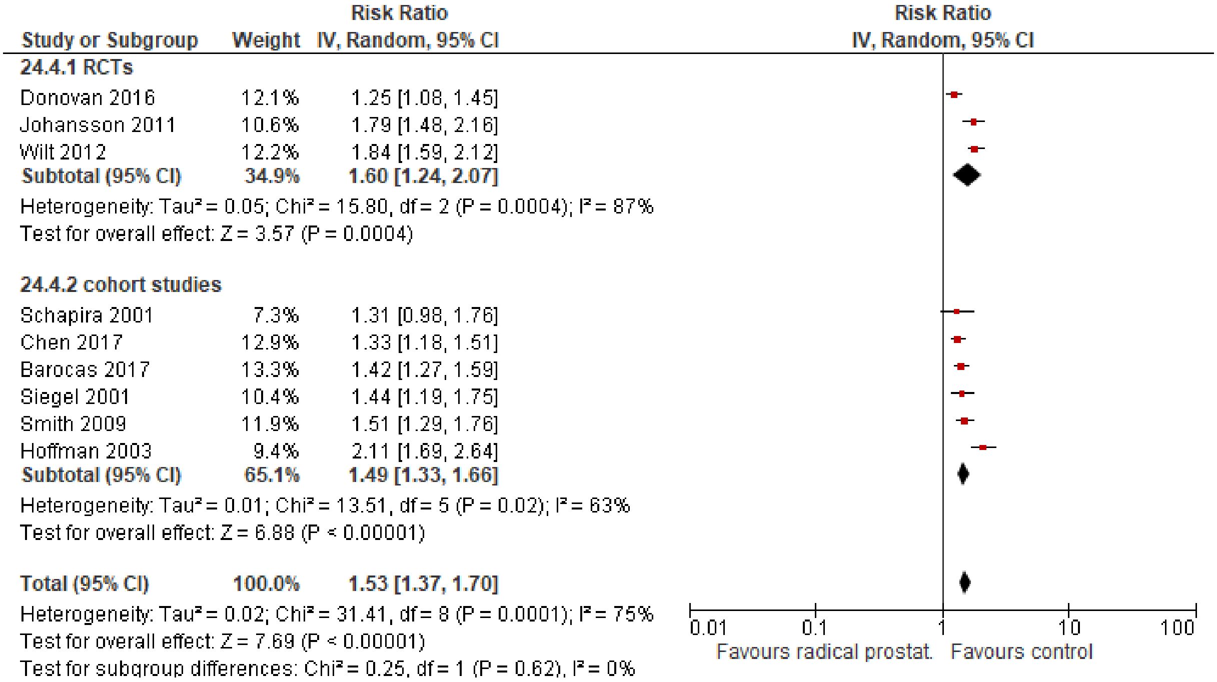

Figure S32: Fenton 2018; Intervention/ Exposure: Radical Prostatectomy; Outcome: Erectile dysfunction

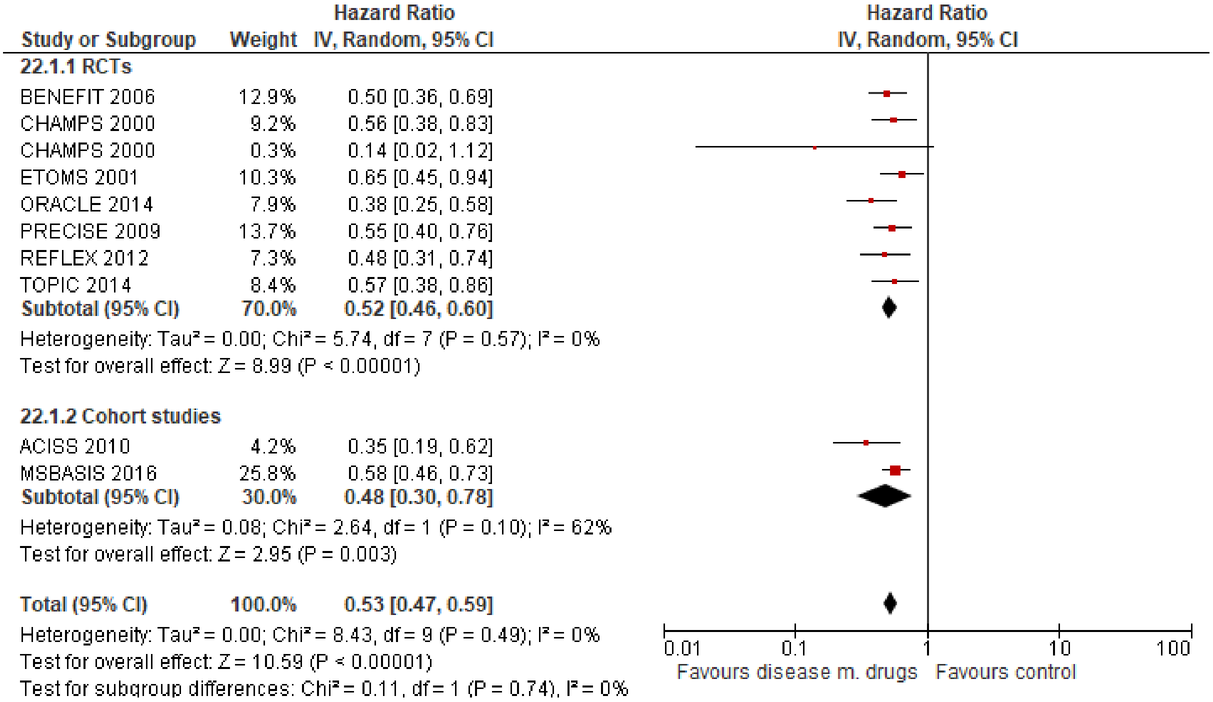

Figure S33: Filippini 2017; Intervention/ Exposure: Disease-modifying drugs; Outcome: Conversion to clinically definite multiple sclerosis

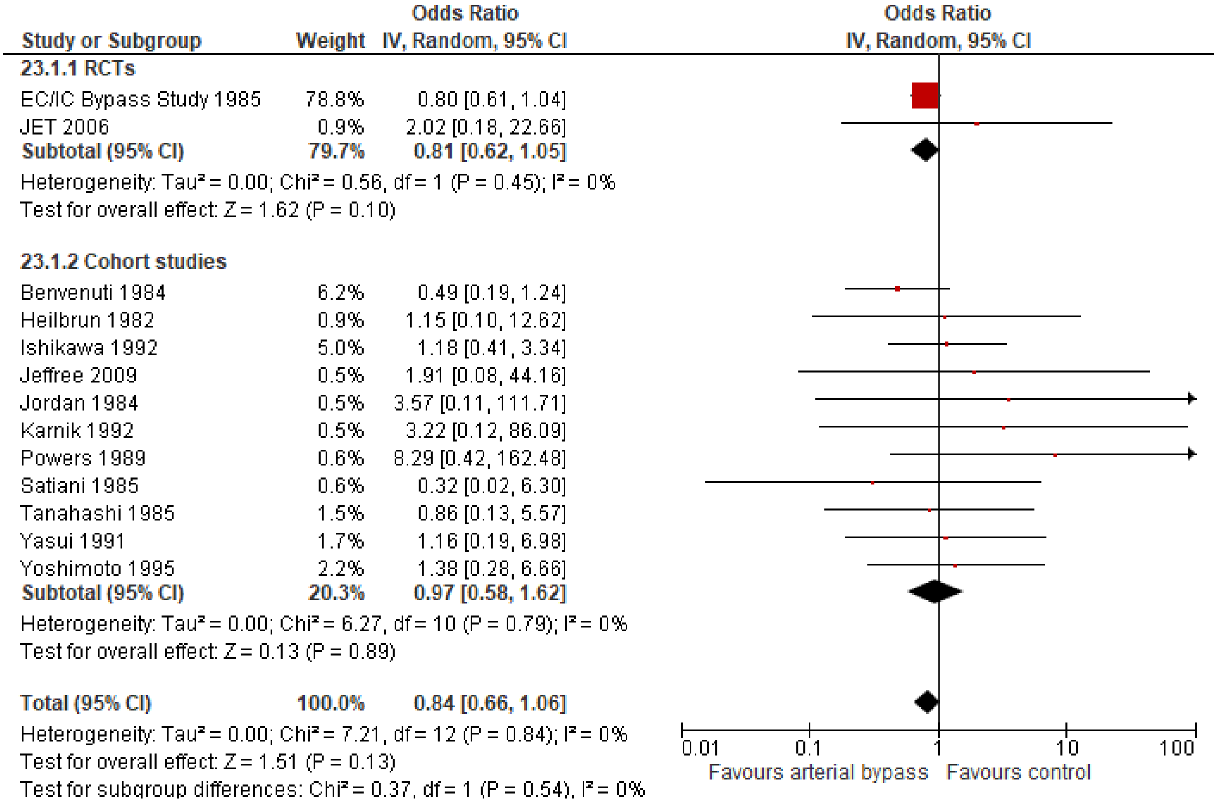

Figure S34: Fluri 2010; Intervention/ Exposure: Extracranial-intracranial arterial bypass; Outcome: All-cause mortality

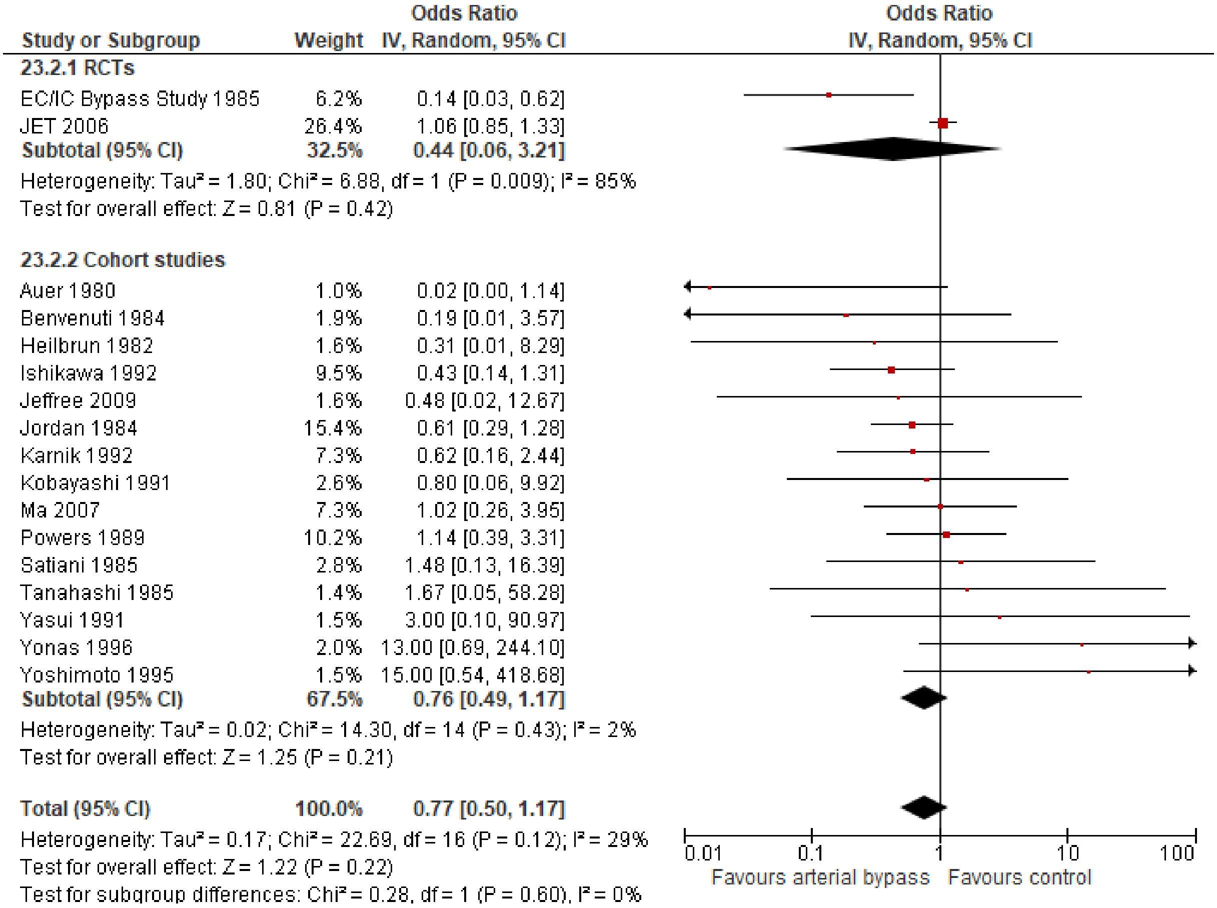

Figure S35: Fluri 2010; Intervention/ Exposure: Extracranial-intracranial arterial bypass; Outcome: Stroke

Study or Subgroup	Weight	Odds Ratio IV, Random, 95% CI
**23.5.1 RCTs**		
EC/IC Bypass Study 1985	79.4%	0.94 [0.74, 1.21]
**Subtotal (95% CI)**	**79.4%**	**0.94 [0.74, 1.21]**
Heterogeneity: Not applicable		
Test for overall effect: Z = 0.47 (P = 0.64)		
**23.5.2 Cohort studies**		
Auer 1980	0.6%	1.57 [0.09, 27.21]
Ishikawa 1992	4.8%	0.96 [0.35, 2.62]
Jeffree 2009	0.8%	1.07 [0.09, 12.83]
Kobayashi 1991	0.4%	0.10 [0.00, 3.24]
Ma 2007	0.8%	1.33 [0.11, 15.70]
Tanahashi 1985	3.4%	0.95 [0.29, 3.11]
Yasui 1991	4.8%	1.14 [0.42, 3.09]
Yoshimoto 1995	5.1%	0.43 [0.16, 1.15]
**Subtotal (95% CI)**	**20.6%**	**0.81 [0.50, 1.31]**
Heterogeneity: Tau² = 0.00; Chi² = 4.04, df = 7 (P = 0.78); I² = 0%		
Test for overall effect: Z = 0.85 (P = 0.39)		
**Total (95% CI)**	**100.0%**	**0.91 [0.73, 1.14]**
Heterogeneity: Tau² = 0.00; Chi² = 4.33, df = 8 (P = 0.83); I² = 0%		
Test for overall effect: Z = 0.80 (P = 0.42)		
Test for subgroup differences: Chi² = 0.30, df = 1 (P = 0.59), I² = 0%		

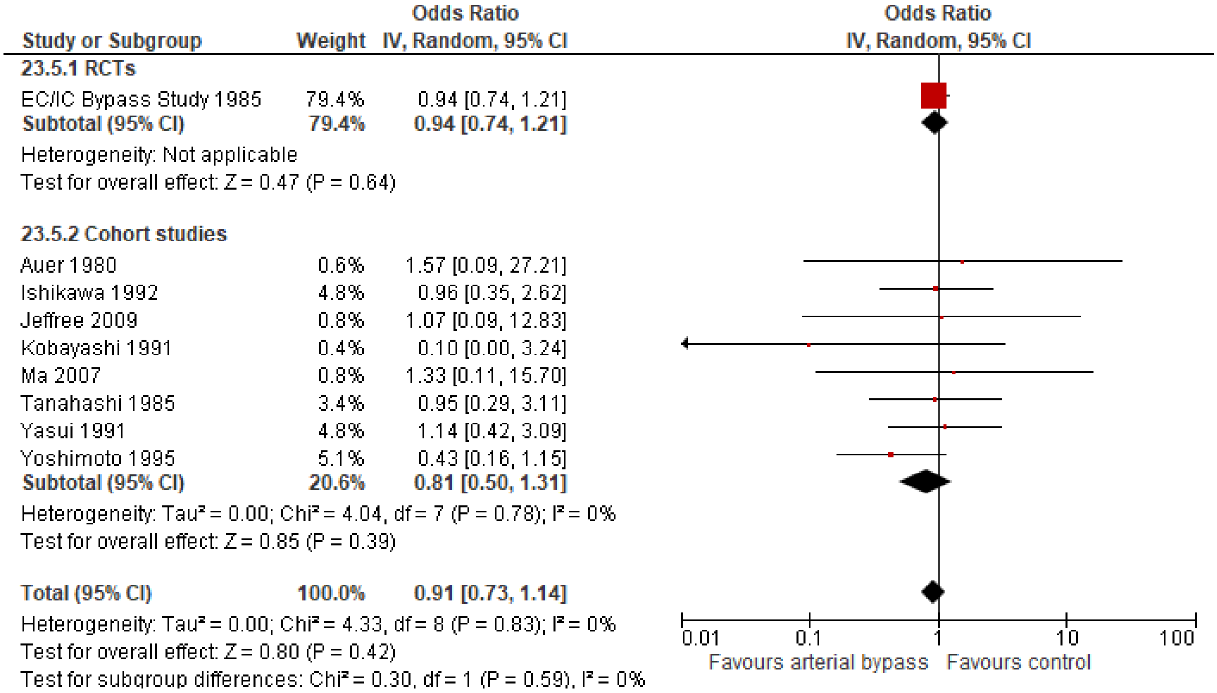

Figure S36: Fluri 2010; Intervention/ Exposure: Extracranial-intracranial arterial bypass; Outcome: Stroke mortality or dependency

Study or Subgroup	Weight	Odds Ratio IV, Random, 95% CI
**20.4.1 RCTs**		
NOTION	1.9%	0.57 [0.13, 2.45]
PARTNER	4.7%	0.53 [0.26, 1.10]
PARTNER 2A	6.8%	0.96 [0.61, 1.50]
STACCATO	0.5%	5.62 [0.26, 121.33]
U.S. CoreValve	4.5%	0.73 [0.35, 1.55]
**Subtotal (95% CI)**	**18.4%**	**0.80 [0.58, 1.11]**
Heterogeneity: Tau² = 0.00; Chi² = 3.64, df = 4 (P = 0.46); I² = 0%		
Test for overall effect: Z = 1.32 (P = 0.19)		
**20.4.2 Cohort studies**		
Ailawadi	5.9%	1.61 [0.92, 2.81]
Appel	1.2%	1.54 [0.24, 9.66]
Biancari	1.7%	5.30 [1.14, 24.63]
Conradi	2.7%	0.85 [0.27, 2.63]
D'Onofrio	0.5%	5.27 [0.24, 113.60]
Fusari	0.5%	0.19 [0.01, 4.06]
Guarracino	0.8%	3.22 [0.32, 32.89]
Hannan	5.2%	1.00 [0.52, 1.92]
Higgins	2.1%	1.58 [0.41, 6.00]
Holzhey	4.6%	0.76 [0.36, 1.58]
Johansson	1.8%	1.00 [0.23, 4.31]
Jones		Not estimable
Kobrin	4.8%	1.37 [0.68, 2.77]
Latib	1.1%	1.00 [0.14, 7.23]
Minutello	6.1%	1.34 [0.79, 2.30]
Muneretto	5.2%	2.23 [1.16, 4.27]
Onorati	0.4%	3.11 [0.12, 79.63]
Osnabrugge	1.2%	0.65 [0.10, 4.10]
Papadopoulos	1.8%	0.46 [0.11, 1.98]
Piazza	6.1%	1.35 [0.79, 2.31]
Santarpino	1.8%	0.59 [0.14, 2.53]
Schymik	2.2%	0.32 [0.09, 1.21]
Stöhr	4.7%	1.70 [0.82, 3.51]
Tamburino	5.5%	0.83 [0.45, 1.51]
Thongprayoon	1.3%	1.51 [0.25, 9.12]
Thourani	5.2%	0.27 [0.14, 0.52]
Walther	3.9%	0.63 [0.27, 1.48]
Wendt	2.0%	2.72 [0.69, 10.63]
Zweng	1.1%	1.00 [0.13, 7.43]
**Subtotal (95% CI)**	**81.6%**	**1.08 [0.84, 1.39]**
Heterogeneity: Tau² = 0.16; Chi² = 46.10, df = 27 (P = 0.01); I² = 41%		
Test for overall effect: Z = 0.58 (P = 0.56)		
**Total (95% CI)**	**100.0%**	**1.01 [0.81, 1.26]**
Heterogeneity: Tau² = 0.13; Chi² = 52.41, df = 32 (P = 0.01); I² = 39%		
Test for overall effect: Z = 0.12 (P = 0.91)		
Test for subgroup differences: Chi² = 1.96, df = 1 (P = 0.16), I² = 48.9%		

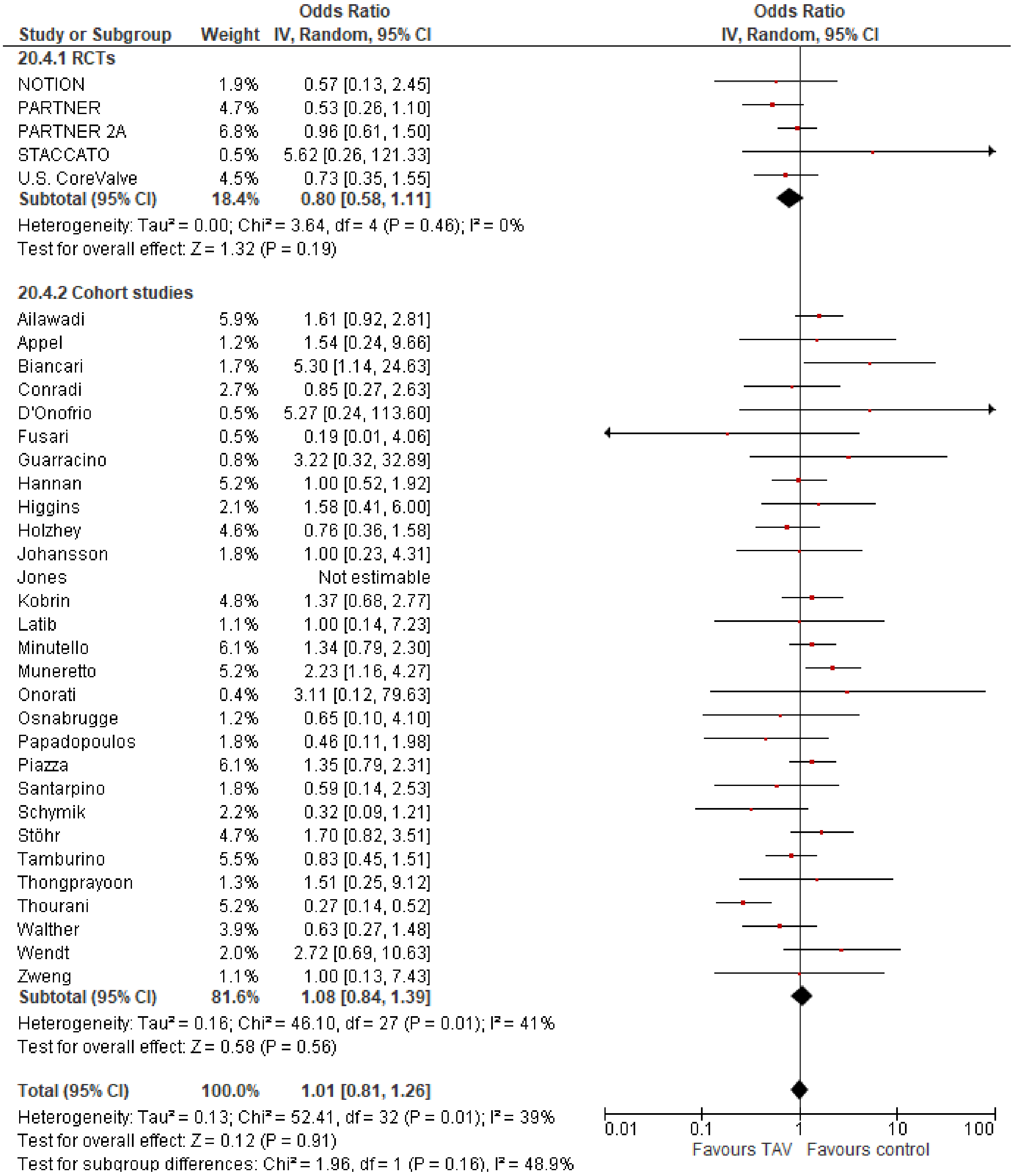

Figure S37: Gargiulo 2016; Intervention/ Exposure: Transcatheter aortic valve implantation; Outcome: Early all-cause mortality

Study or Subgroup	Weight	Odds Ratio IV, Random, 95% CI
**21.2.1 RCTs**		
NOTION	2.2%	0.66 [0.24, 1.80]
PARTNER	8.6%	0.94 [0.66, 1.32]
PARTNER 2A	10.1%	1.00 [0.77, 1.31]
STACCATO	0.3%	10.77 [0.56, 208.08]
U.S. CoreValve	7.7%	0.71 [0.48, 1.05]
**Subtotal (95% CI)**	**29.0%**	**0.90 [0.71, 1.13]**
Heterogeneity: Tau² = 0.01; Chi² = 5.14, df = 4 (P = 0.27); I² = 22%		
Test for overall effect: Z = 0.94 (P = 0.35)		
**21.2.2 Cohort studies**		
Appel	0.9%	2.69 [0.49, 14.64]
Conradi	3.2%	1.09 [0.49, 2.43]
Ewe	4.1%	0.74 [0.38, 1.46]
Fusari	0.5%	0.17 [0.02, 1.58]
Guarracino	0.5%	3.22 [0.32, 32.89]
Hannan	7.7%	1.22 [0.82, 1.82]
Holzhey	6.1%	1.50 [0.92, 2.46]
Johansson	2.0%	1.16 [0.40, 3.40]
Latib	2.1%	0.76 [0.27, 2.13]
Osnabrugge	1.5%	1.48 [0.43, 5.10]
Papadopoulos	1.4%	0.52 [0.14, 1.95]
Piazza	8.2%	1.07 [0.74, 1.55]
Schymik	4.2%	1.64 [0.85, 3.16]
Tamburino	8.9%	1.01 [0.73, 1.41]
Thongprayoon	3.3%	0.73 [0.34, 1.59]
Thourani	9.5%	0.54 [0.40, 0.73]
Walther	4.7%	0.87 [0.47, 1.59]
Wendt	2.2%	1.83 [0.68, 4.96]
**Subtotal (95% CI)**	**71.0%**	**1.00 [0.81, 1.24]**
Heterogeneity: Tau² = 0.08; Chi² = 31.22, df = 17 (P = 0.02); I² = 46%		
Test for overall effect: Z = 0.02 (P = 0.98)		
**Total (95% CI)**	**100.0%**	**0.96 [0.82, 1.13]**
Heterogeneity: Tau² = 0.05; Chi² = 36.47, df = 22 (P = 0.03); I² = 40%		
Test for overall effect: Z = 0.46 (P = 0.64)		
Test for subgroup differences: Chi² = 0.49, df = 1 (P = 0.49), I² = 0%		

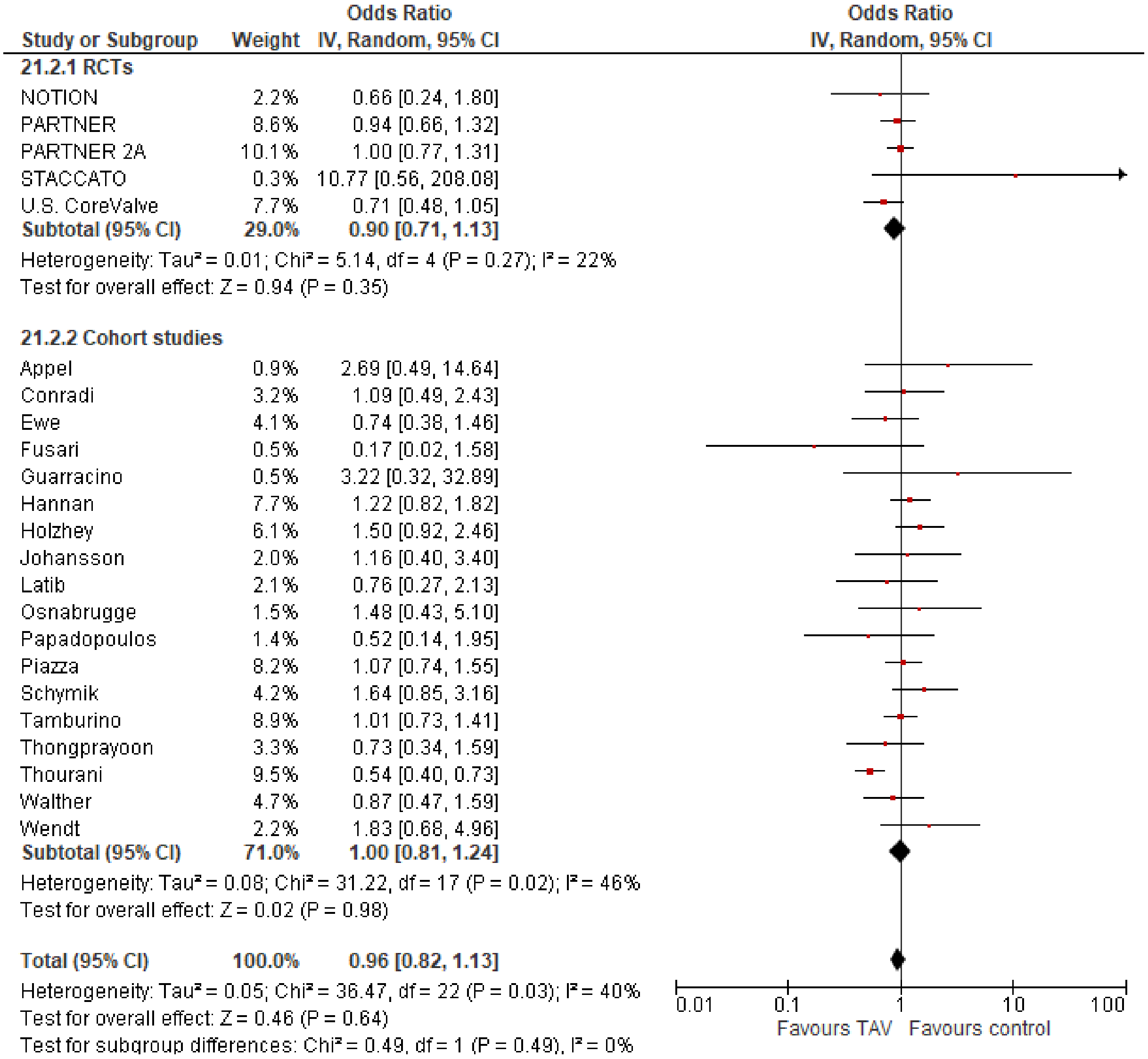

Figure S38: Gargiulo 2016; Intervention/ Exposure: Transcatheter aortic valve implantation; Outcome: Mid-term all-cause mortality

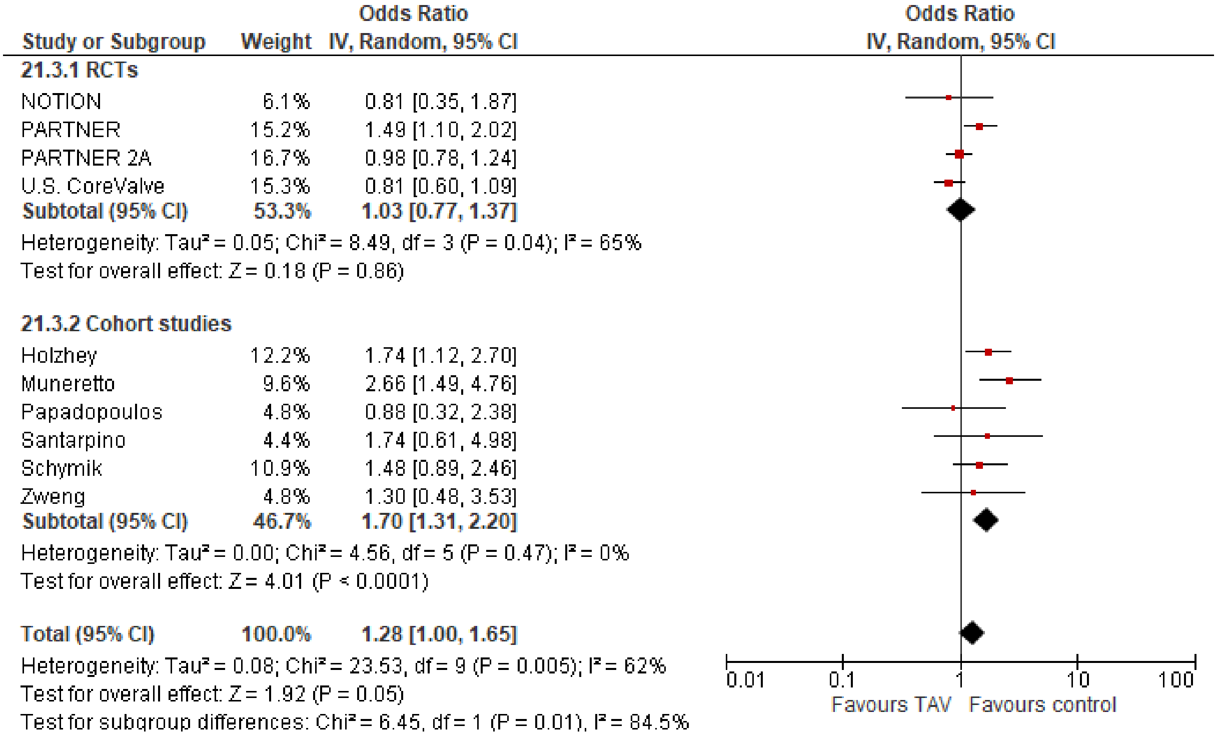

Figure S39: Gargiulo 2016; Intervention/ Exposure: Transcatheter aortic valve implantation; Outcome: Long-term all-cause mortality

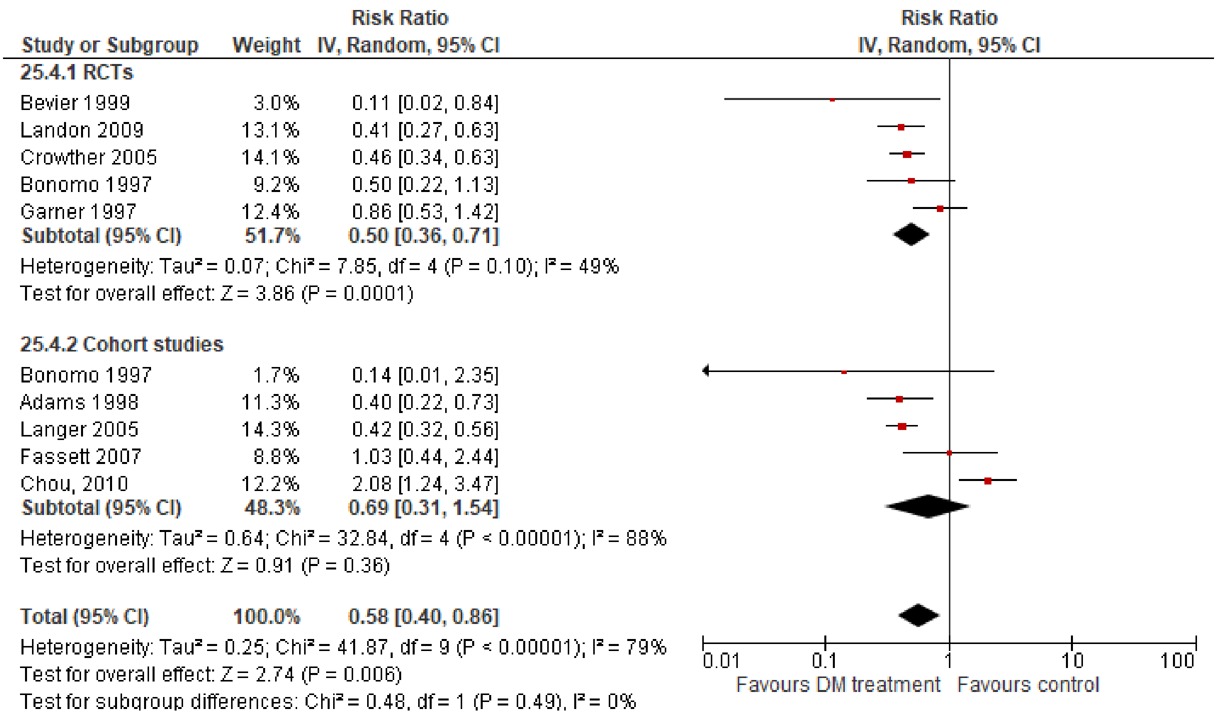

Figure S40: Hartling 2013; Intervention/ Exposure: Treating gestational diabetes mellitus; Outcome: High birth weight

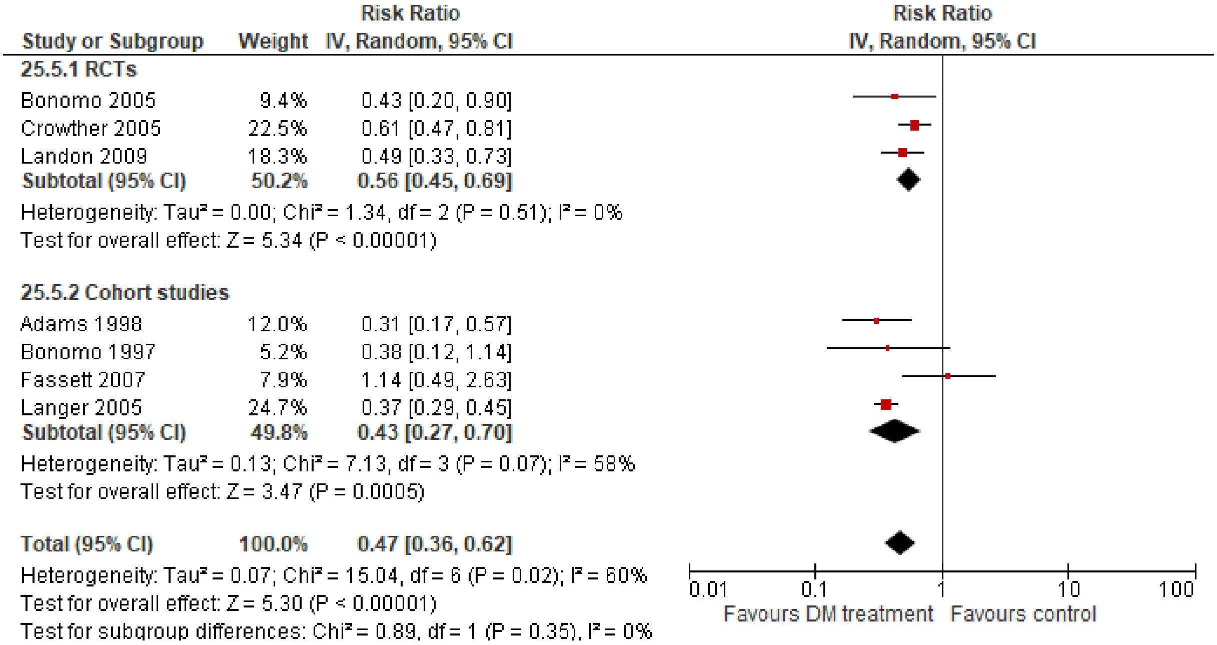

Figure S41: Hartling 2013; Intervention/ Exposure: Treating gestational diabetes mellitus; Outcome: Large-for-gestational age neonate

Study or Subgroup	Weight	Risk Ratio IV, Random, 95% CI
**25.6.1 RCTs**		
Bevier 1999	3.2%	0.69 [0.06, 7.27]
Crowther 2005	22.9%	0.45 [0.19, 1.09]
Landon 2009	23.8%	0.37 [0.16, 0.88]
**Subtotal (95% CI)**	**49.9%**	**0.42 [0.23, 0.77]**
Heterogeneity: Tau² = 0.00; Chi² = 0.27, df = 2 (P = 0.87); I² = 0%		
Test for overall effect: Z = 2.83 (P = 0.005)		
**25.6.2 Cohort Studies**		
Adams 1998	13.4%	0.19 [0.06, 0.59]
Chou, 2010	4.6%	1.27 [0.18, 8.98]
Fassett 2007	4.8%	0.83 [0.12, 5.68]
Langer 2005	27.4%	0.36 [0.16, 0.80]
**Subtotal (95% CI)**	**50.1%**	**0.38 [0.19, 0.75]**
Heterogeneity: Tau² = 0.09; Chi² = 3.57, df = 3 (P = 0.31); I² = 16%		
Test for overall effect: Z = 2.77 (P = 0.006)		
**Total (95% CI)**	**100.0%**	**0.39 [0.26, 0.60]**
Heterogeneity: Tau² = 0.00; Chi² = 3.96, df = 6 (P = 0.68); I² = 0%		
Test for overall effect: Z = 4.34 (P < 0.0001)		
Test for subgroup differences: Chi² = 0.06, df = 1 (P = 0.81), I² = 0%		

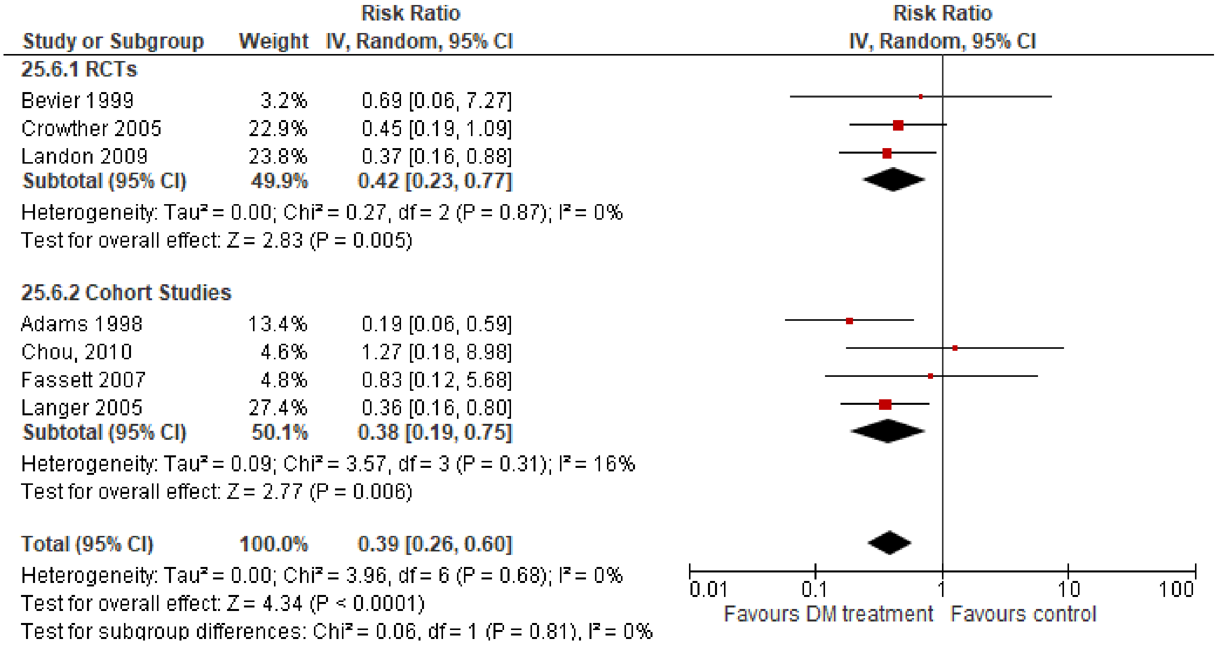

Figure S42: Hartling 2013; Intervention/ Exposure: Treating gestational diabetes mellitus; Outcome: Shoulder dystocia

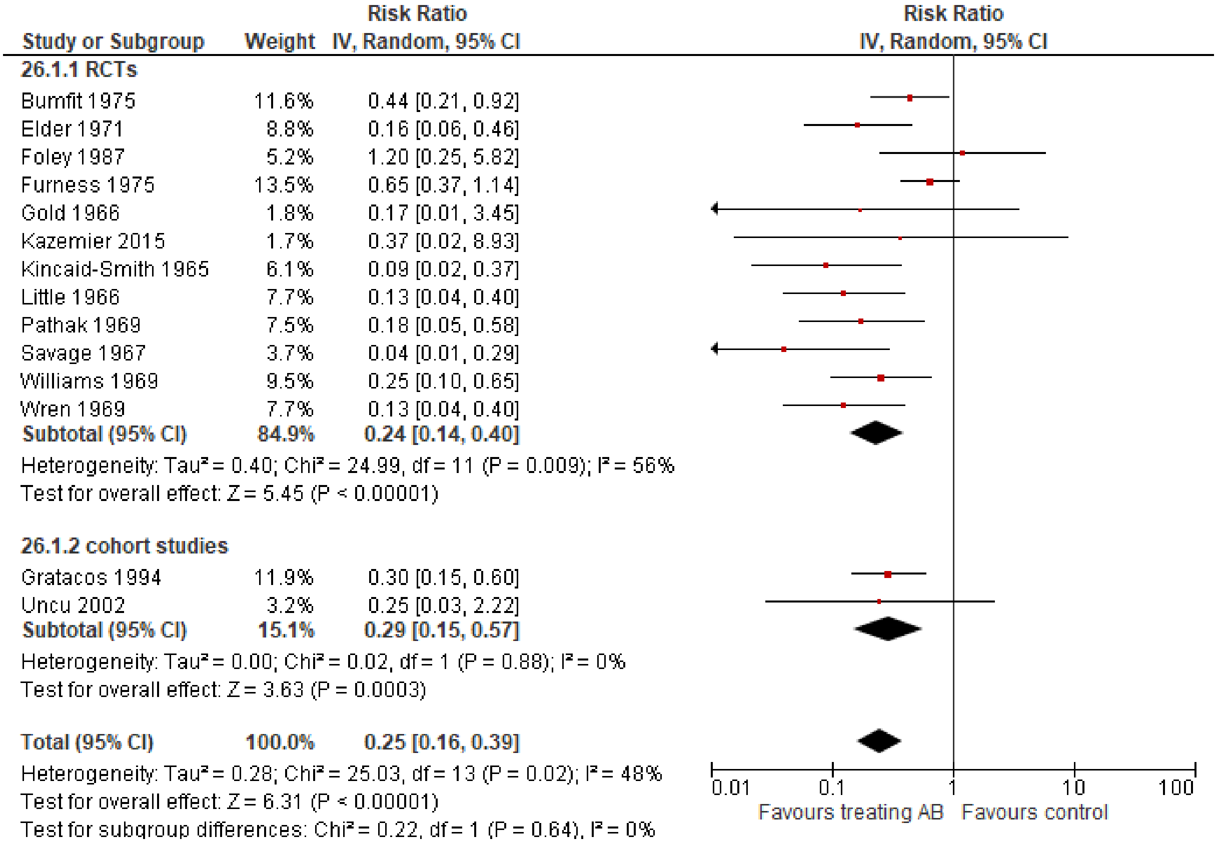

Figure S43: Henderson 2019; Intervention/ Exposure: Treating asymptomatic bacteriuria; Outcome: Pyelonephritis

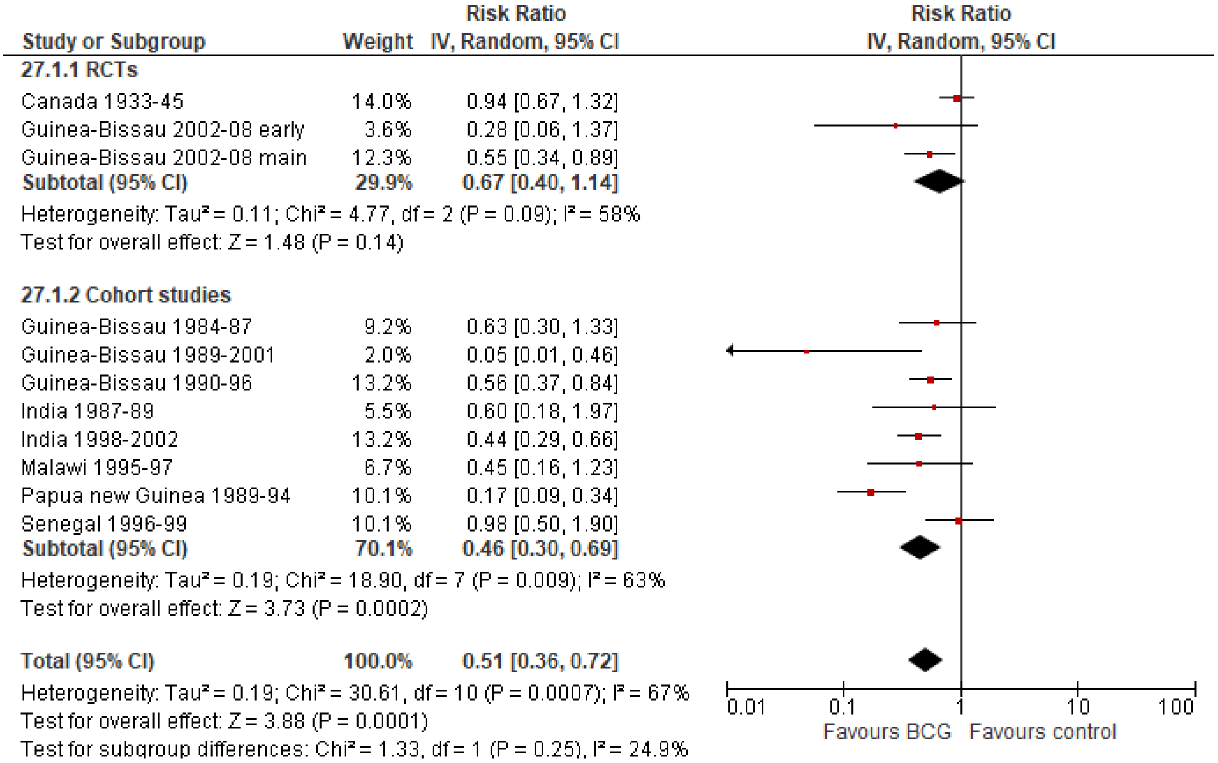

Figure S44: Higgins 2016; Intervention/ Exposure: Bacillus Calmette-Guérin vaccination; Outcome: All-cause mortality

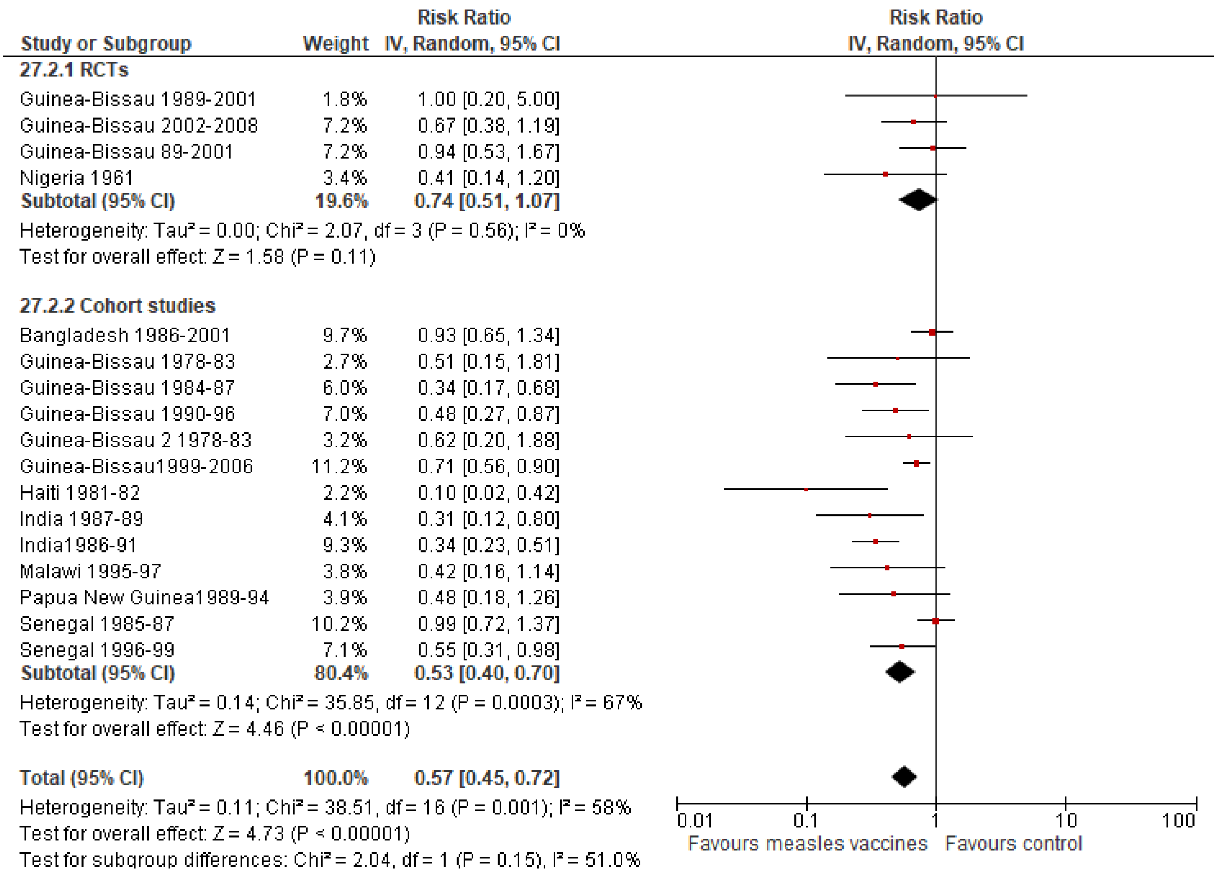

Figure S45: Higgins 2016; Intervention/ Exposure: Measles containing vaccines; Outcome: All-cause mortality

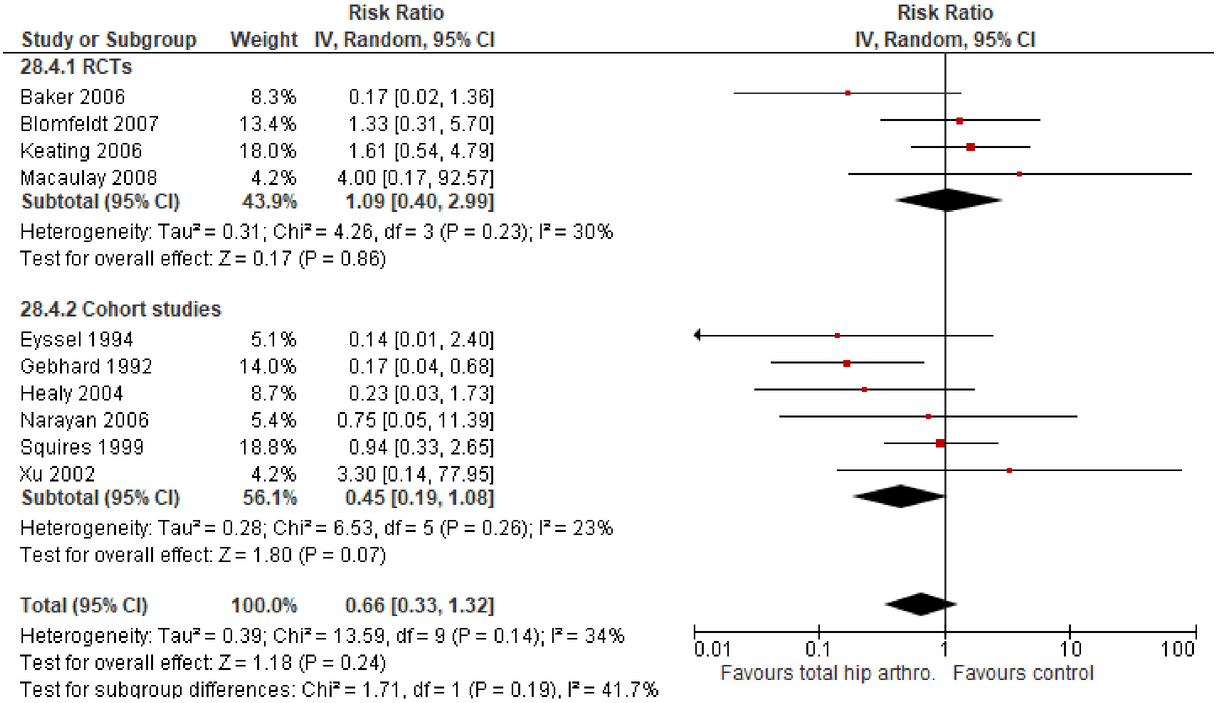

Figure S46: Hopley 2010; Intervention/ Exposure: Total hip arthroplasty; Outcome: Reoperation

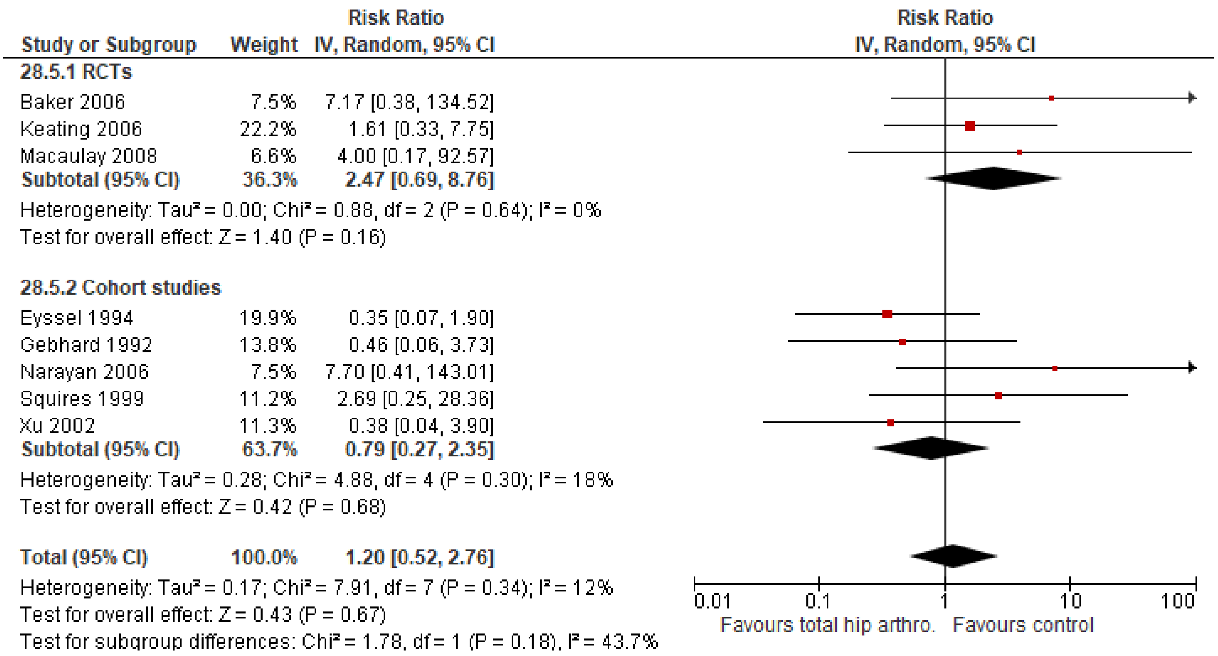

Figure S47: Hopley 2010; Intervention/ Exposure: Total hip arthroplasty; Outcome: Dislocation

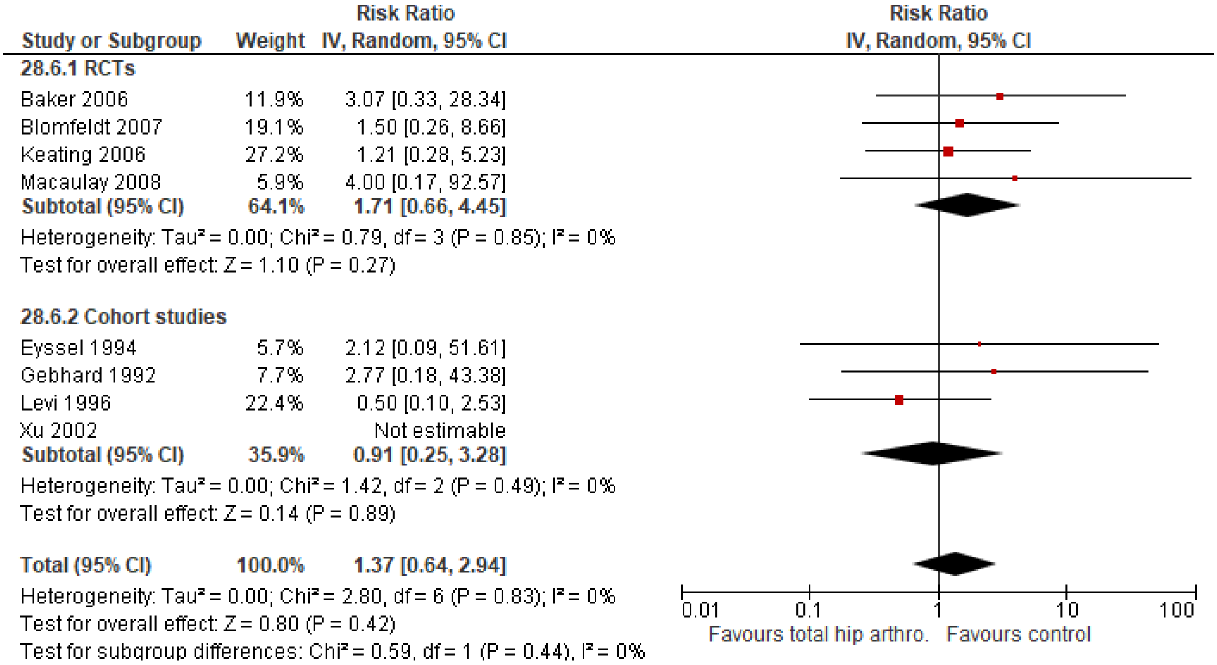

Figure S48: Hopley 2010; Intervention/ Exposure: Total hip arthroplasty; Outcome: Deep infection

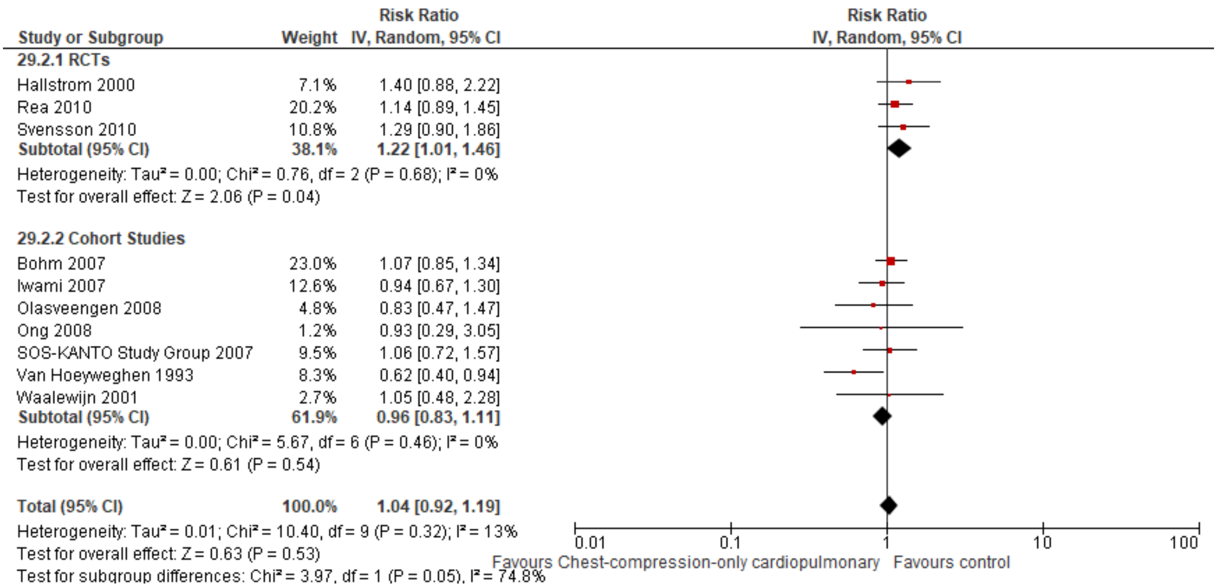

Figure S49: Hüpfl 2010; Intervention/ Exposure: Chest-compression-only cardiopulmonary resuscitation; Outcome: Survival (Risk Ratio >1 indicates a favorable effect)

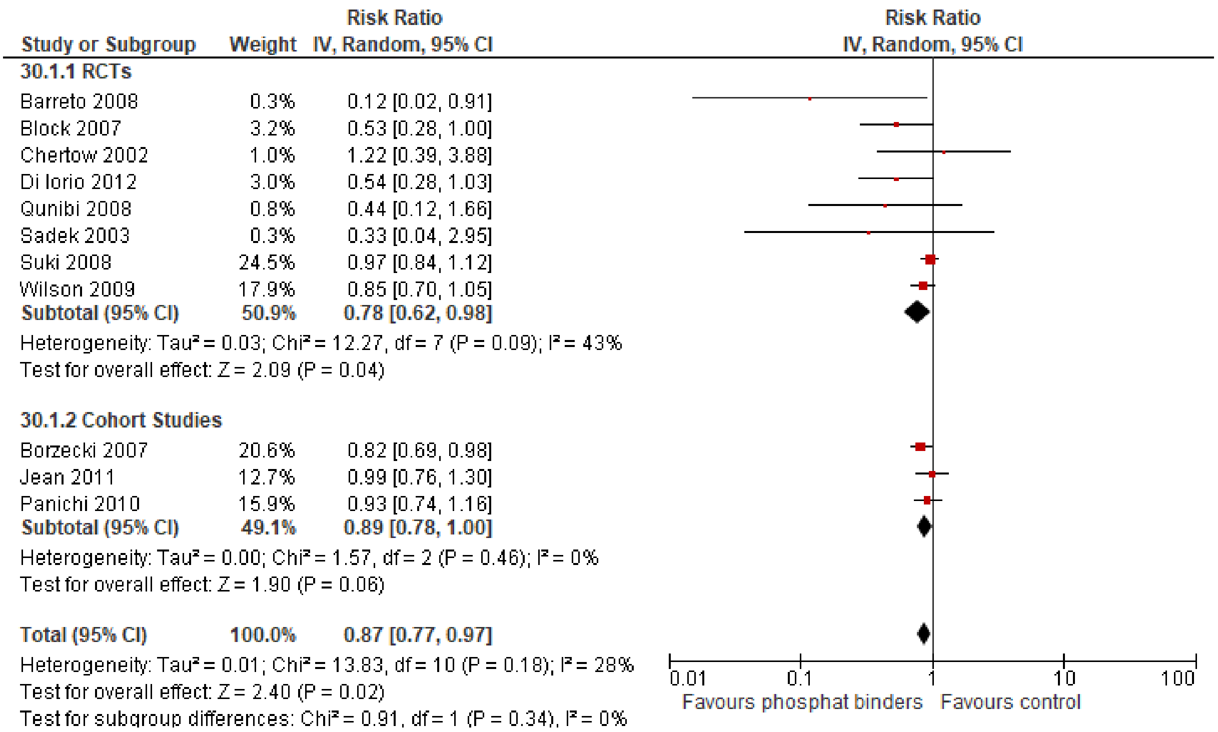

Figure S50: Jamal 2013; Intervention/ Exposure: Non-calcium-based phosphat binders; Outcome: All-cause mortality

Study or Subgroup	Weight	Risk Ratio IV, Random, 95% CI
**31.3.1 RCTs**		
Allsup 2004	2.5%	0.48 [0.26, 0.87]
Edmondscon 1971	1.3%	0.35 [0.13, 0.92]
Govaert 1994	4.0%	0.62 [0.42, 0.90]
Govaert 1994 risk groups	2.7%	0.87 [0.49, 1.53]
Stuart 1969	3.9%	0.54 [0.37, 0.80]
**Subtotal (95% CI)**	**14.4%**	**0.59 [0.47, 0.73]**
Heterogeneity: Tau² = 0.00; Chi² = 3.67, df = 4 (P = 0.45); I² = 0%		
Test for overall effect: Z = 4.78 (P < 0.00001)		
**31.3.2 Cohort studies**		
Arden 1988	1.4%	0.58 [0.23, 1.45]
Arroyo 1984	2.9%	0.79 [0.46, 1.34]
Caminiti 1994	1.9%	0.43 [0.20, 0.92]
Cartter 1990a	0.9%	1.82 [0.56, 5.92]
Cartter 1990b	2.4%	1.57 [0.84, 2.95]
Cartter 1990c	3.8%	1.14 [0.76, 1.70]
Coles 1992	1.2%	1.21 [0.44, 3.37]
Comeri 1995	1.5%	1.40 [0.59, 3.32]
Currier 1988	3.4%	0.94 [0.60, 1.48]
D'Alessio 1969	2.0%	0.98 [0.47, 2.03]
Fyson 1983a	3.0%	0.55 [0.33, 0.93]
Gavira Iglesias 1987	0.5%	0.85 [0.16, 4.55]
Goodman 1982	1.7%	0.58 [0.26, 1.30]
Hara 2006	2.5%	0.44 [0.24, 0.81]
Horman 1986	2.4%	1.08 [0.58, 2.02]
Isaacs 1997	3.5%	0.73 [0.47, 1.14]
Kawai 2003	1.4%	0.81 [0.33, 2.03]
Leung 2007	3.1%	0.96 [0.59, 1.58]
Meiklejohn 1987	3.4%	0.46 [0.29, 0.73]
Monto 2001	5.3%	0.91 [0.73, 1.13]
Morens 1995	0.5%	0.83 [0.15, 4.49]
Mukerjee 1994	5.1%	0.44 [0.35, 0.57]
Murayama 1999	4.1%	0.75 [0.52, 1.08]
Patriarca 1985a	5.4%	0.63 [0.51, 0.77]
Patriarca 1985b	3.1%	0.65 [0.39, 1.07]
Ruben 1974	4.3%	0.51 [0.36, 0.72]
Saito 2002a	4.8%	0.58 [0.44, 0.76]
Saito 2002b	2.8%	1.22 [0.70, 2.12]
Strassburg 1986	3.4%	0.90 [0.58, 1.41]
Taylor 1992	4.0%	1.07 [0.74, 1.55]
**Subtotal (95% CI)**	**85.6%**	**0.76 [0.66, 0.87]**
Heterogeneity: Tau² = 0.07; Chi² = 67.85, df = 29 (P < 0.0001); I² = 57%		
Test for overall effect: Z = 4.00 (P < 0.0001)		
**Total (95% CI)**	**100.0%**	**0.73 [0.64, 0.82]**
Heterogeneity: Tau² = 0.06; Chi² = 74.40, df = 34 (P < 0.0001); I² = 54%		
Test for overall effect: Z = 5.09 (P < 0.00001)		
Test for subgroup differences: Chi² = 3.81, df = 1 (P = 0.05), I² = 73.8%		

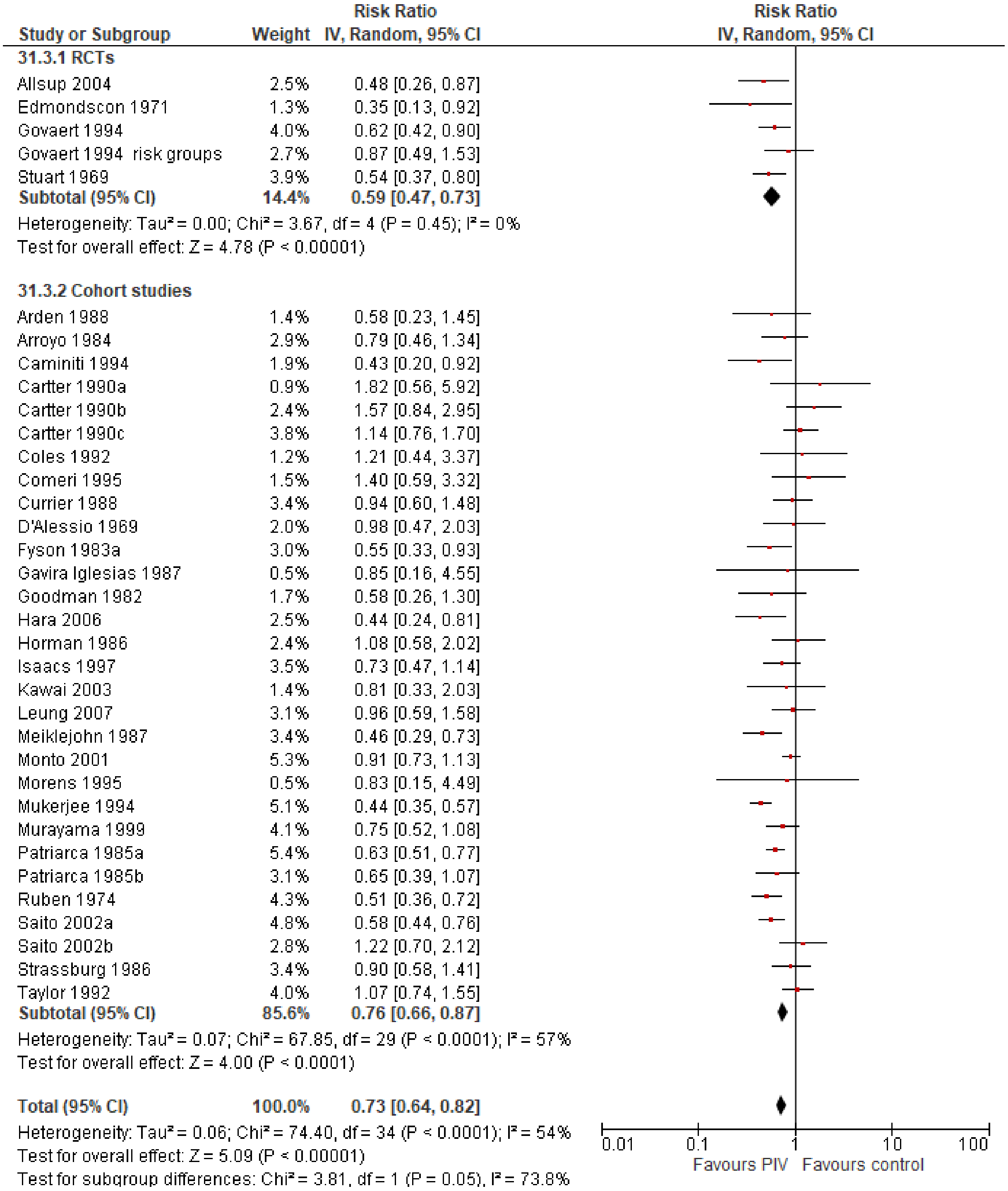

Figure S51: Jefferson 2010; Intervention/ Exposure: Parenteral influenza vaccine; Outcome: Influenza-like illness

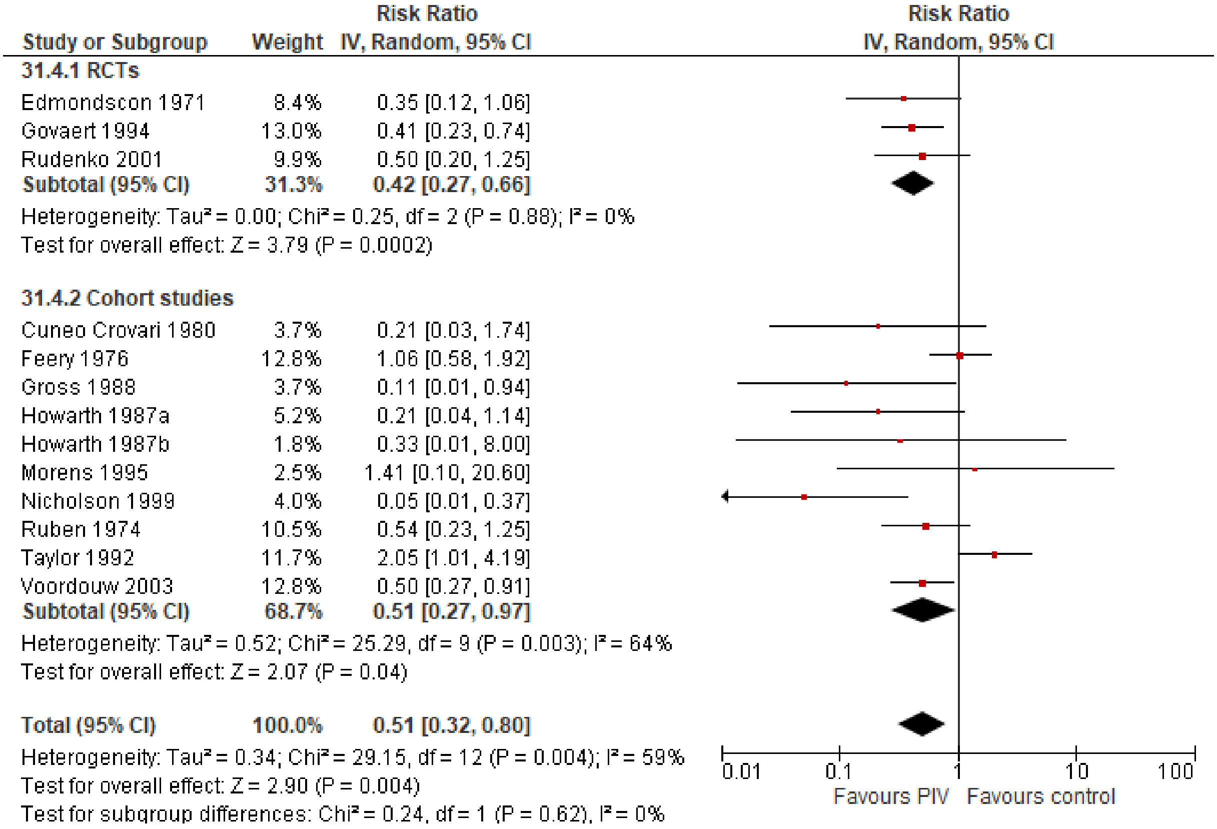

Figure S52: Jefferson 2010; Intervention/ Exposure: Parenteral influenza vaccine; Outcome: Influenza

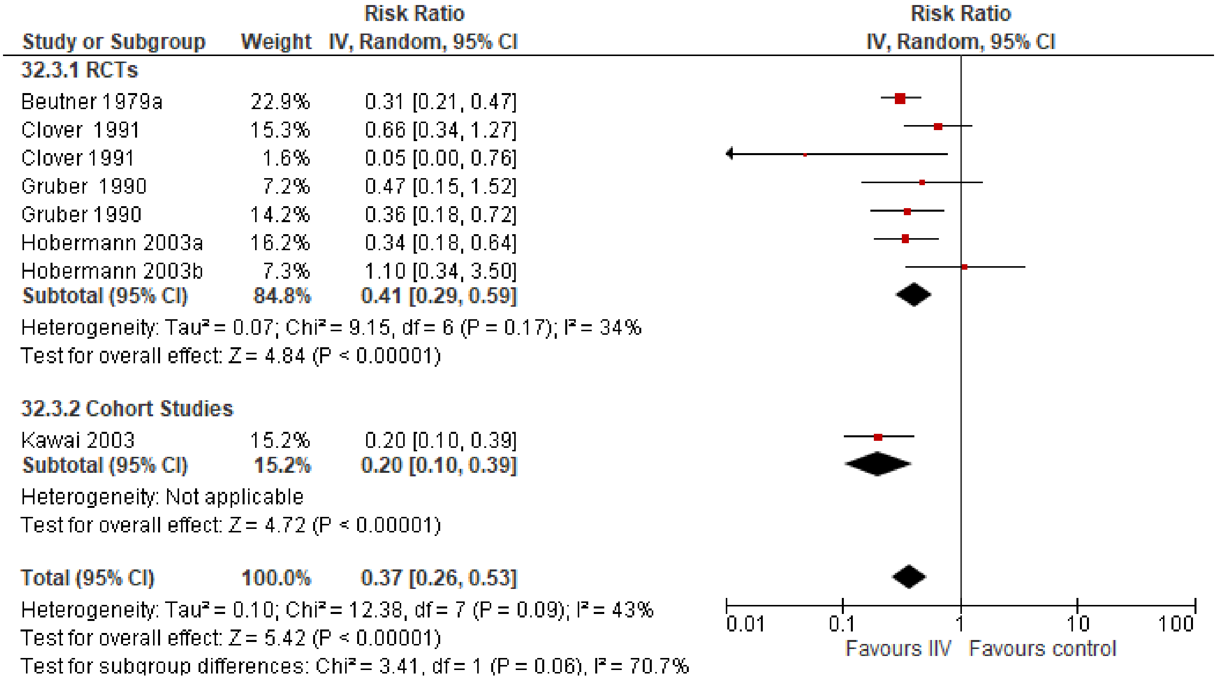

Figure S53: Jefferson 2012; Intervention/ Exposure: Inactivated influenza vaccines; Outcome: Influenza

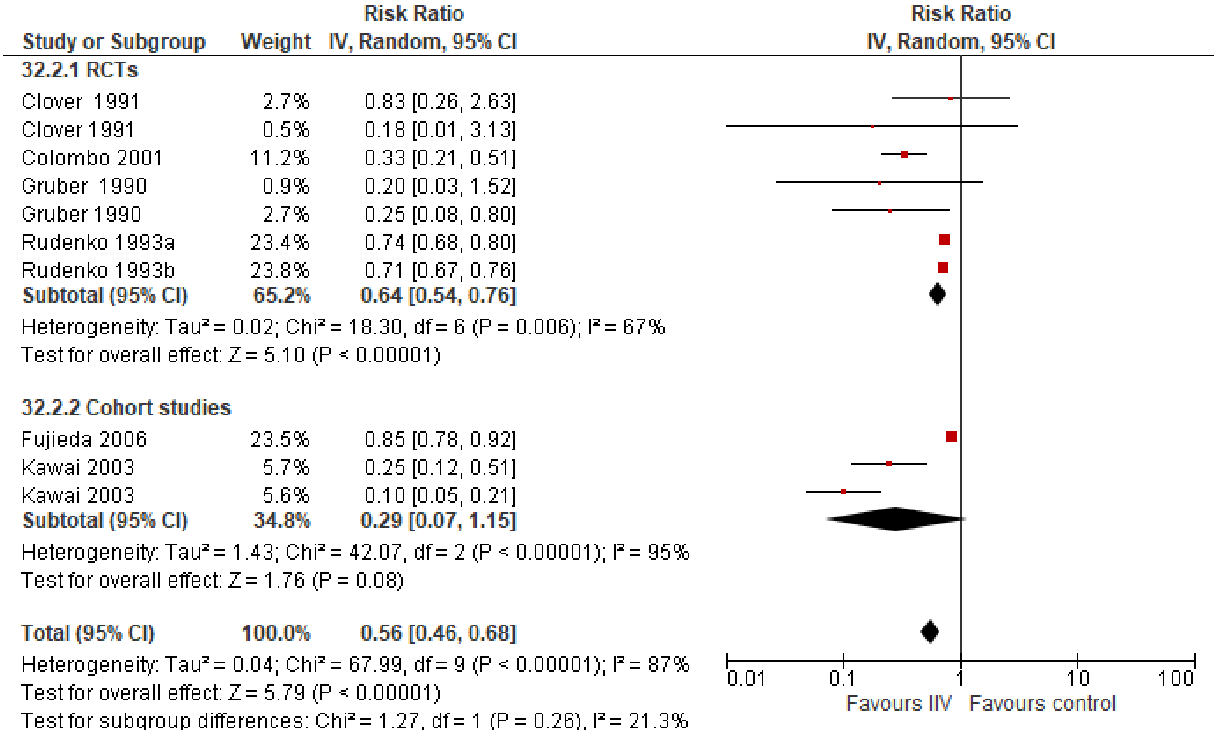

Figure S54: Jefferson 2012; Intervention/ Exposure: Inactivated influenza vaccines; Outcome: Influenza-like illness

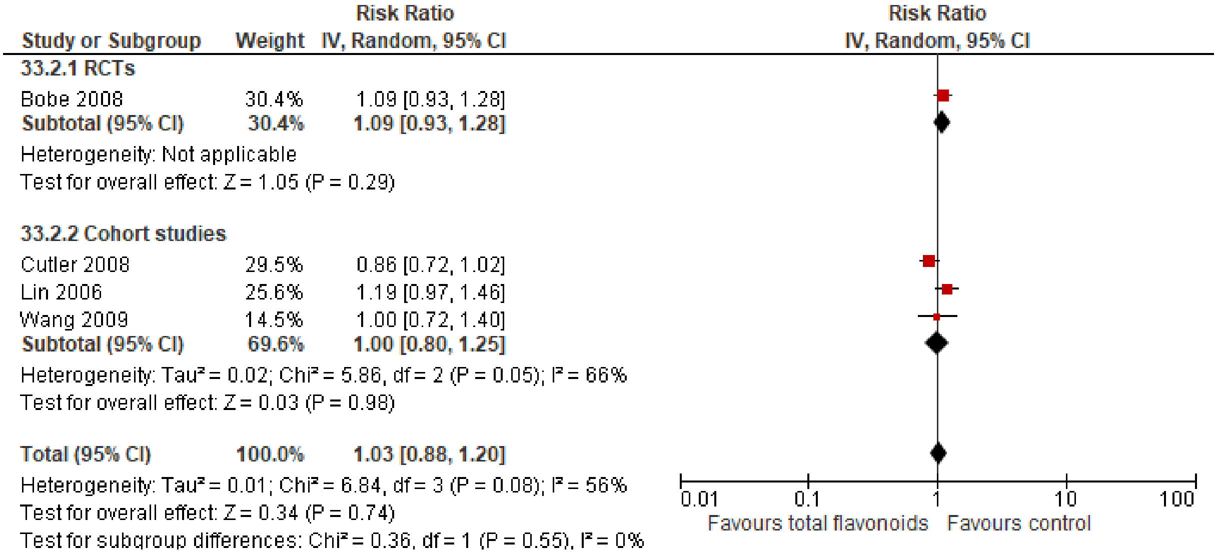

Figure S55: Jin 2012; Intervention/ Exposure: High total flavonoids; Outcome: Colorectal neoplasms

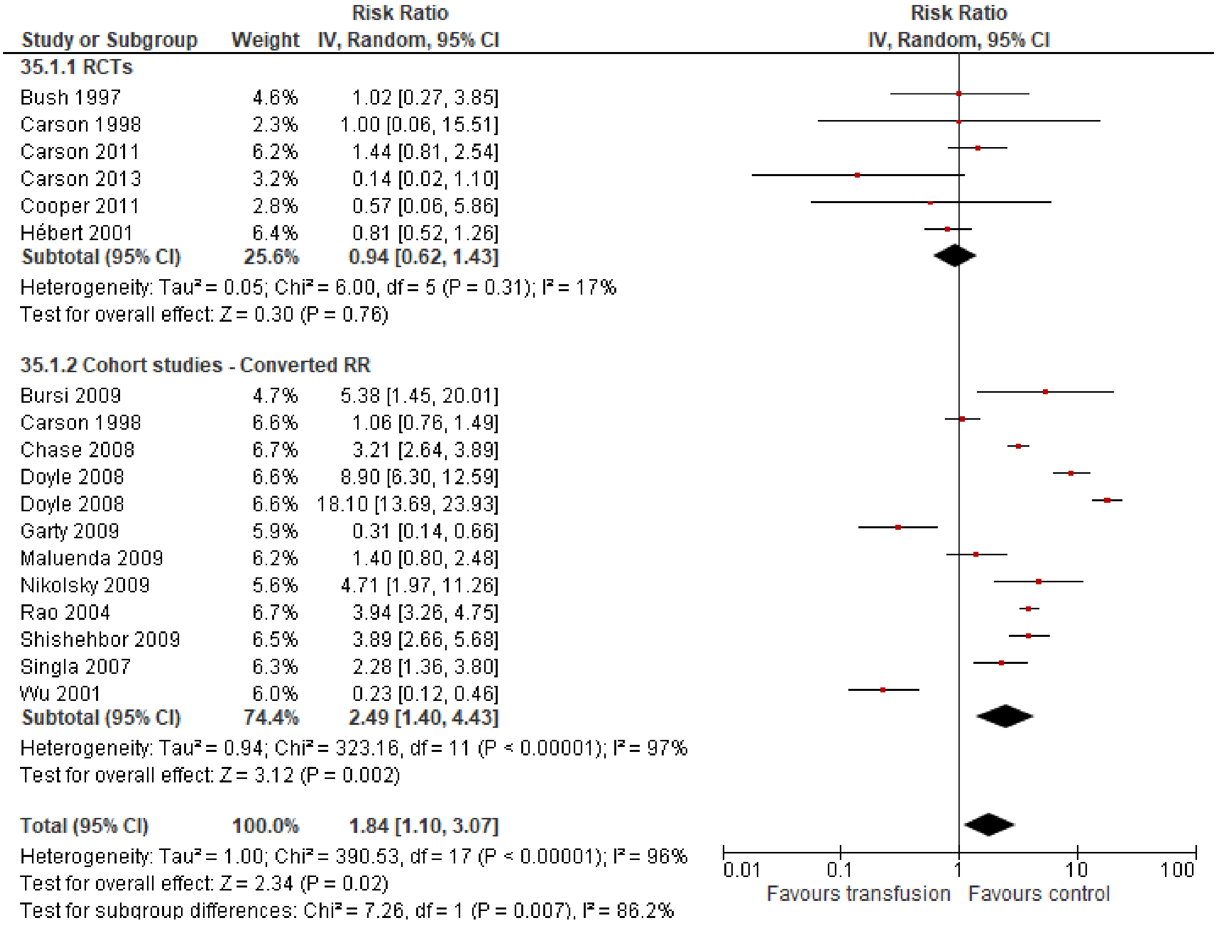

Figure S56: Kansagara 2013; Intervention/ Exposure: Transfusion; Outcome: All-cause mortality

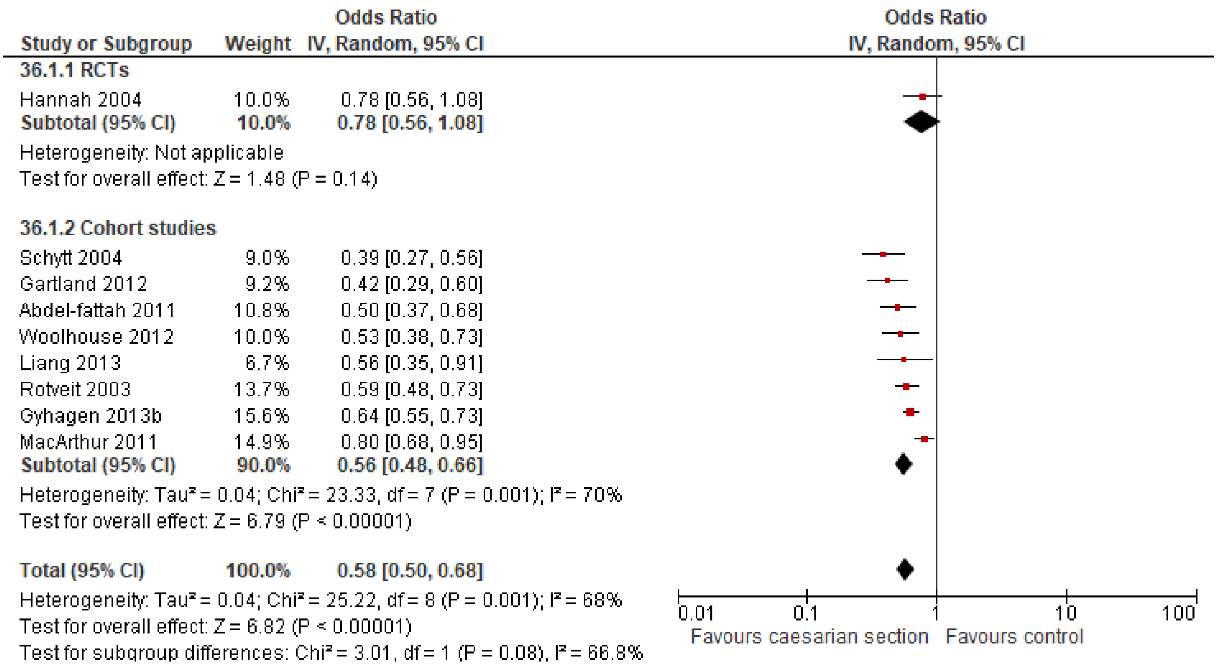

Figure S57: Keag 2018; Intervention/ Exposure: Caesarean section; Outcome: Urinary incontinence

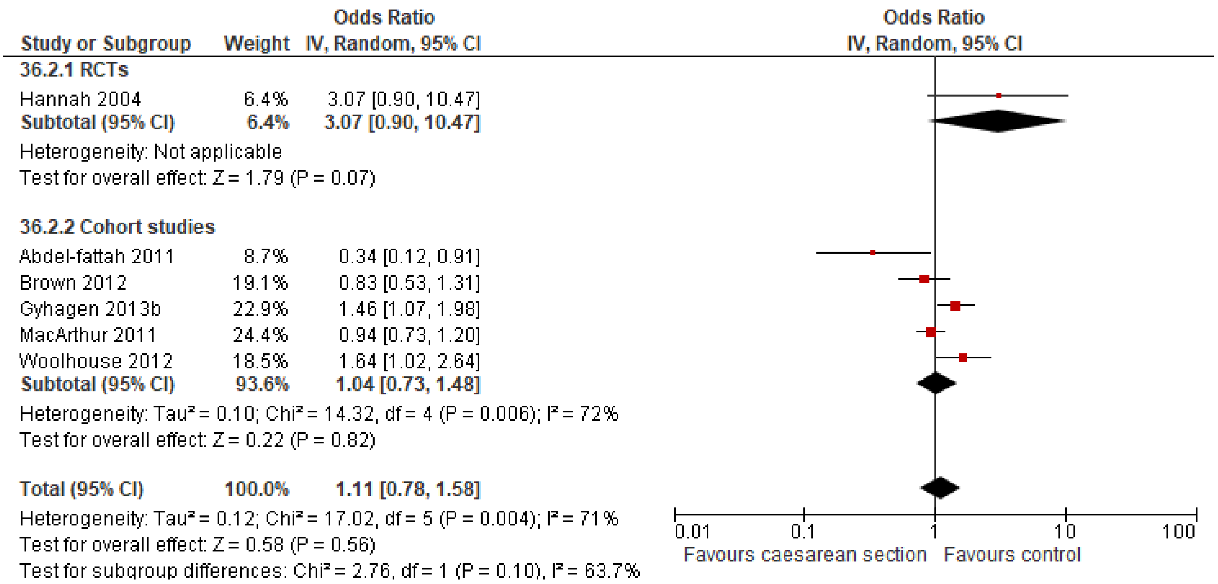

Figure S58: Keag 2018; Intervention/ Exposure: Caesarean section; Outcome: Fecal incontinence

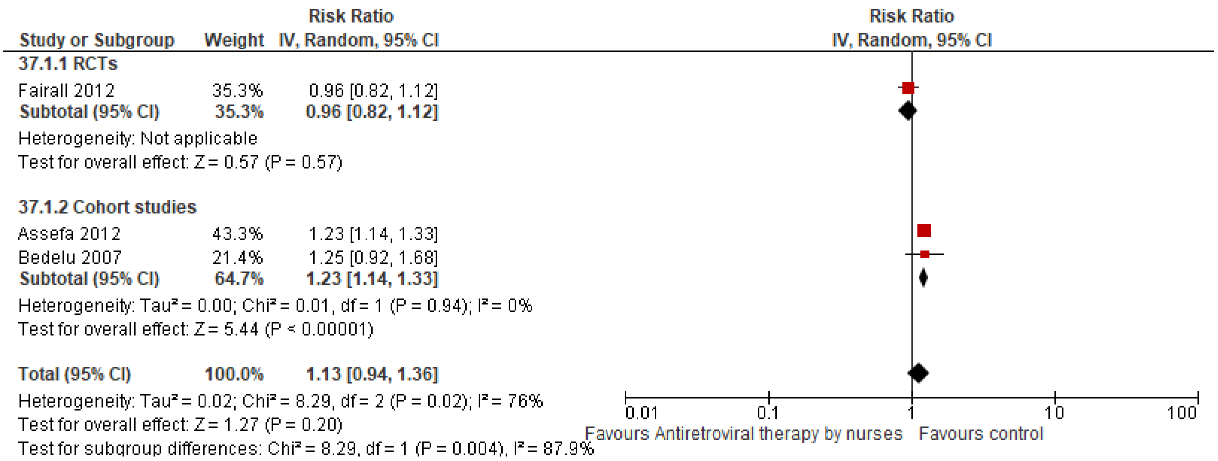

Figure S59: Kredo 2014; Intervention/ Exposure: Antiretroviral therapy by nurses; Outcome: All-cause mortality

Study or Subgroup	Weight	Risk Ratio IV, Random, 95% CI
**37.2.1 RCTs**		
Fairall 2012	34.4%	0.73 [0.55, 0.97]
**Subtotal (95% CI)**	**34.4%**	**0.73 [0.55, 0.97]**
Heterogeneity: Not applicable		
Test for overall effect: Z = 2.16 (P = 0.03)		
**37.2.2 Cohort Studies**		
Assefa 2012	36.4%	0.76 [0.72, 0.80]
Bedelu 2007	29.3%	0.11 [0.06, 0.20]
**Subtotal (95% CI)**	**65.6%**	**0.30 [0.05, 1.94]**
Heterogeneity: Tau² = 1.77; Chi² = 42.34, df = 1 (P < 0.00001); I² = 98%		
Test for overall effect: Z = 1.27 (P = 0.21)		
**Total (95% CI)**	**100.0%**	**0.43 [0.21, 0.86]**
Heterogeneity: Tau² = 0.35; Chi² = 42.37, df = 2 (P < 0.00001); I² = 95%		
Test for overall effect: Z = 2.38 (P = 0.02)		
Test for subgroup differences: Chi² = 0.86, df = 1 (P = 0.35), I² = 0%		

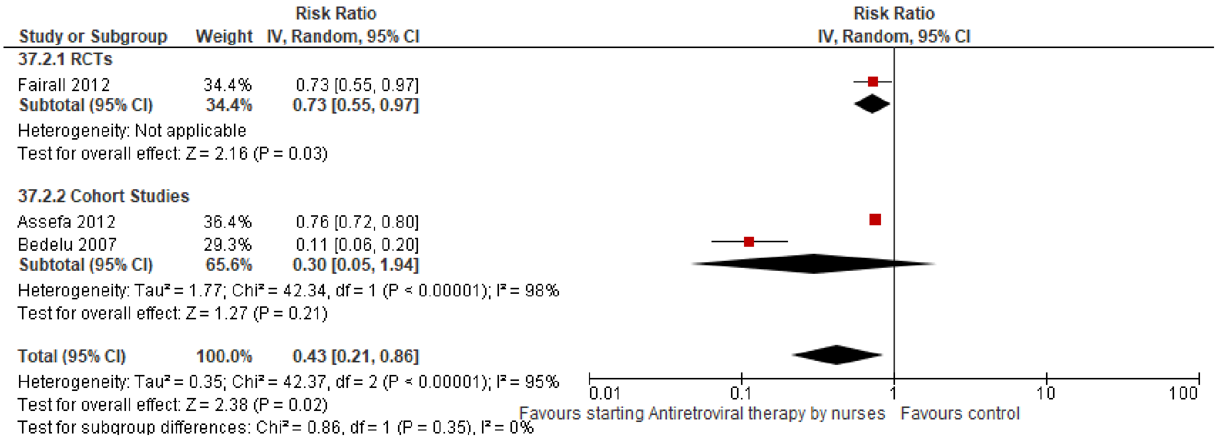

Figure S60: Kredo 2014; Intervention/ Exposure: Antiretroviral therapy by nurses; Outcome: Attrition

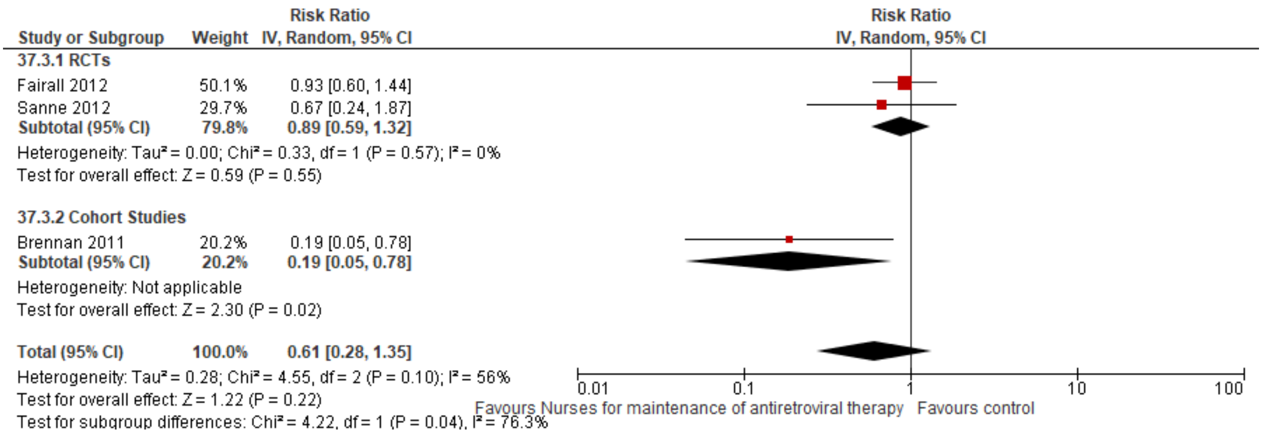

Figure S61: Kredo 2014; Intervention/ Exposure: Nurses for maintenance of antiretroviral therapy; Outcome: All-cause mortality

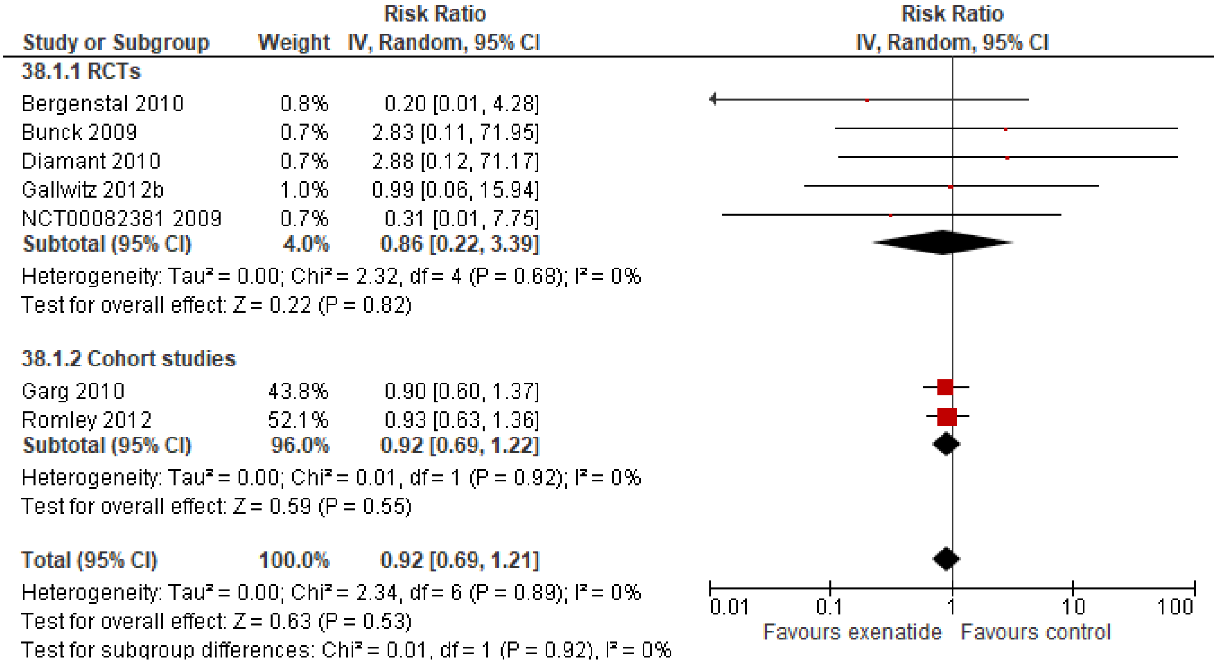

Figure S62: Li 2014; Intervention/ Exposure: Exenatide; Outcome: Acute pancreatitis

Study or Subgroup	Weight	Risk Ratio IV, Random, 95% CI
**39.2.1 RCTs**		
Fonseca 2013		Not estimable
NCT 00327015 2009	0.0%	0.10 [0.00, 2.12]
Arjona Fereira 2013b	0.0%	0.11 [0.01, 2.06]
NCT 00295633 2009	0.0%	0.16 [0.01, 3.94]
NCT 01263483 2011	0.0%	0.16 [0.01, 3.94]
Yang 2015	0.0%	0.20 [0.01, 4.75]
NCT 00395343 2009	0.0%	0.20 [0.01, 4.11]
NCT 01189890 2013	0.0%	0.33 [0.01, 7.97]
NCT 01006603 2013	0.1%	0.33 [0.03, 3.19]
NCT 00121667 2011	0.1%	0.48 [0.08, 2.83]
Garber 2007	0.0%	0.52 [0.03, 8.25]
NCT 00286442 2011	0.0%	0.74 [0.03, 18.11]
NCT 00286468 2011	0.0%	0.75 [0.03, 18.18]
Iwamoto 2010	0.0%	0.76 [0.03, 18.54]
NCT 00121641 2011	0.0%	0.94 [0.04, 22.84]
NCT 0070993 2013	0.0%	0.99 [0.06, 15.67]
Ferannini 2009	0.1%	1.00 [0.14, 7.06]
NCT 00575588 2010	0.0%	1.00 [0.06, 16.01]
Arjona Ferreira 2013a	0.1%	1.02 [0.15, 6.99]
NCT 00838903 2014	0.0%	1.35 [0.08, 21.51]
NCT 00954447 2012	0.1%	1.50 [0.25, 8.93]
NCT 00622284 2011	0.1%	1.50 [0.25, 8.94]
NCT 00856284 2013	0.1%	1.50 [0.16, 14.44]
Seino 2012	0.0%	1.60 [0.07, 38.99]
Pratley 2009	0.0%	1.72 [0.09, 33.09]
NCT 00642278 2013	0.0%	1.95 [0.08, 47.48]
Bosi 2011	0.1%	1.98 [0.18, 21.70]
NCT 00094770 2009	0.1%	1.99 [0.18, 21.85]
NCT 00757588 2011	0.0%	2.49 [0.12, 51.58]
NCT 00798161 2011	0.0%	2.55 [0.10, 62.29]
NCT 00103857 2009	0.0%	2.94 [0.12, 71.82]
NCT 00482729 2009	0.0%	2.98 [0.12, 73.03]
NCT 00614939 2010	0.0%	3.00 [0.12, 72.62]
Henry 2014	0.0%	5.01 [0.24, 104.26]
NCT 01289990 2014	0.0%	9.07 [0.37, 221.78]
**Subtotal (95% CI)**	**1.6%**	**0.95 [0.60, 1.50]**
Heterogeneity: Tau² = 0.00; Chi² = 17.76, df = 33 (P = 0.99); I² = 0%		
Test for overall effect: Z = 0.22 (P = 0.83)		
**39.2.2 Cohort studies**		
Eurich 2014	0.0%	0.39 [0.02, 7.41]
NCT01357135 2014	0.7%	0.75 [0.38, 1.46]
Gitt 2013	0.2%	0.88 [0.22, 3.48]
Kannan 2015	97.4%	1.10 [1.04, 1.17]
**Subtotal (95% CI)**	**98.4%**	**1.10 [1.04, 1.17]**
Heterogeneity: Tau² = 0.00; Chi² = 1.87, df = 3 (P = 0.60); I² = 0%		
Test for overall effect: Z = 3.16 (P = 0.002)		
**Total (95% CI)**	**100.0%**	**1.10 [1.03, 1.16]**
Heterogeneity: Tau² = 0.00; Chi² = 20.01, df = 37 (P = 0.99); I² = 0%		
Test for overall effect: Z = 3.10 (P = 0.002)		
Test for subgroup differences: Chi² = 0.39, df = 1 (P = 0.53), I² = 0%		

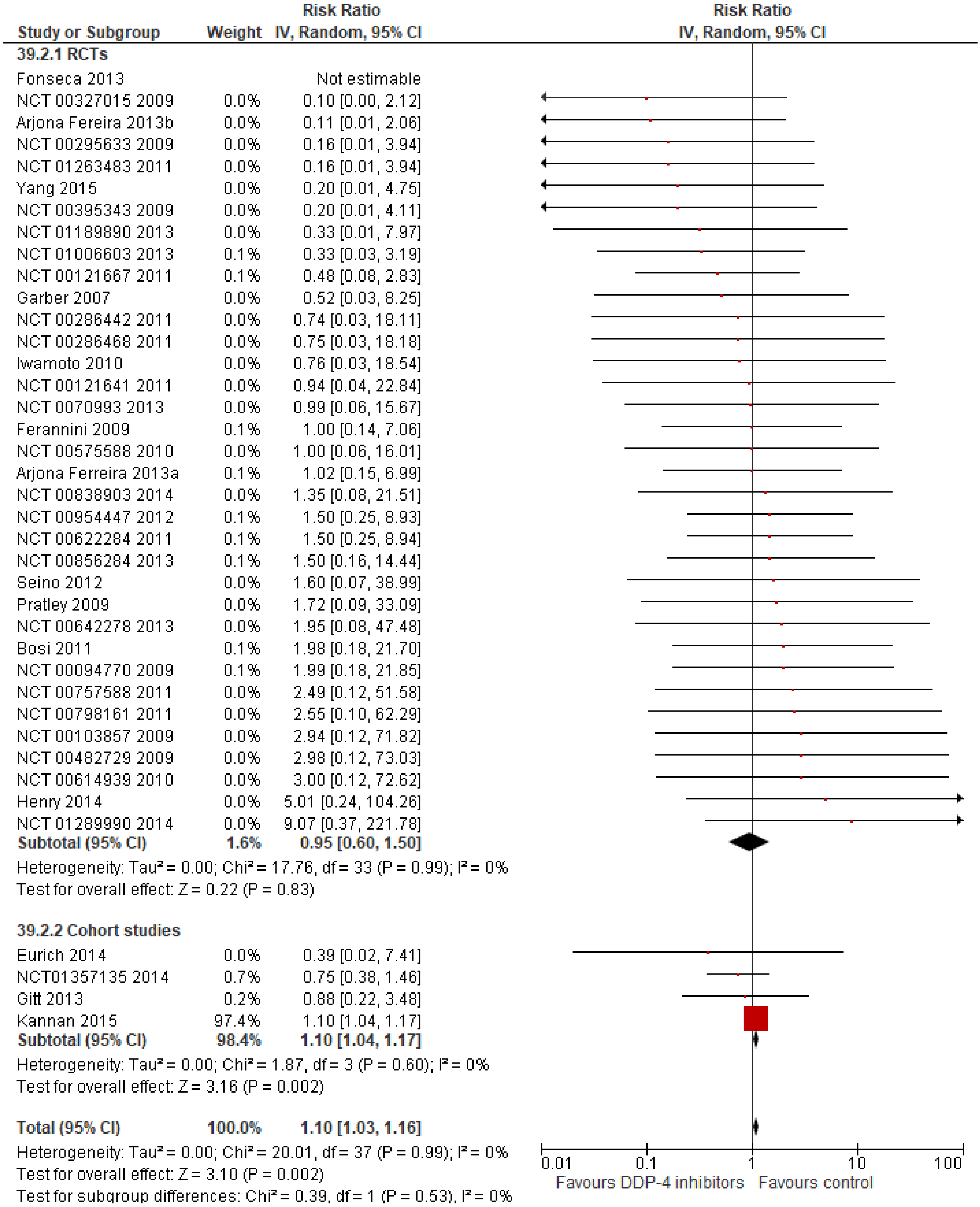

Figure S63: Li 2016; Intervention/ Exposure: DDP-4 inhibitors; Outcome: Heart failure

Study or Subgroup	Weight	Odds Ratio IV, Random, 95% CI
**39.1.1 RCTs**		
Green 2015	14.4%	1.00 [0.83, 1.20]
Krum 2014	2.0%	1.29 [0.54, 3.06]
Laakso 2015	1.3%	1.25 [0.41, 3.85]
Scirica 2013	14.8%	1.27 [1.06, 1.51]
Zannad 2015	9.4%	1.07 [0.78, 1.46]
**Subtotal (95% CI)**	**41.9%**	**1.13 [1.00, 1.27]**
Heterogeneity: Tau² = 0.00; Chi² = 3.59, df = 4 (P = 0.46); I² = 0%		
Test for overall effect: Z = 1.99 (P = 0.05)		
**39.1.2 Cohort studies**		
Fadini 2015	12.5%	0.78 [0.62, 0.98]
Fu 2015	14.9%	0.87 [0.73, 1.04]
Seong 2015	0.8%	0.44 [0.10, 1.91]
Suh 2015	14.8%	0.99 [0.83, 1.18]
Velez 2015	6.5%	0.58 [0.38, 0.88]
Yu 2015	8.7%	0.88 [0.63, 1.23]
**Subtotal (95% CI)**	**58.1%**	**0.85 [0.74, 0.97]**
Heterogeneity: Tau² = 0.01; Chi² = 7.42, df = 5 (P = 0.19); I² = 33%		
Test for overall effect: Z = 2.41 (P = 0.02)		
**Total (95% CI)**	**100.0%**	**0.94 [0.83, 1.08]**
Heterogeneity: Tau² = 0.02; Chi² = 22.15, df = 10 (P = 0.01); I² = 55%		
Test for overall effect: Z = 0.87 (P = 0.39)		
Test for subgroup differences: Chi² = 9.75, df = 1 (P = 0.002), I² = 89.7%		

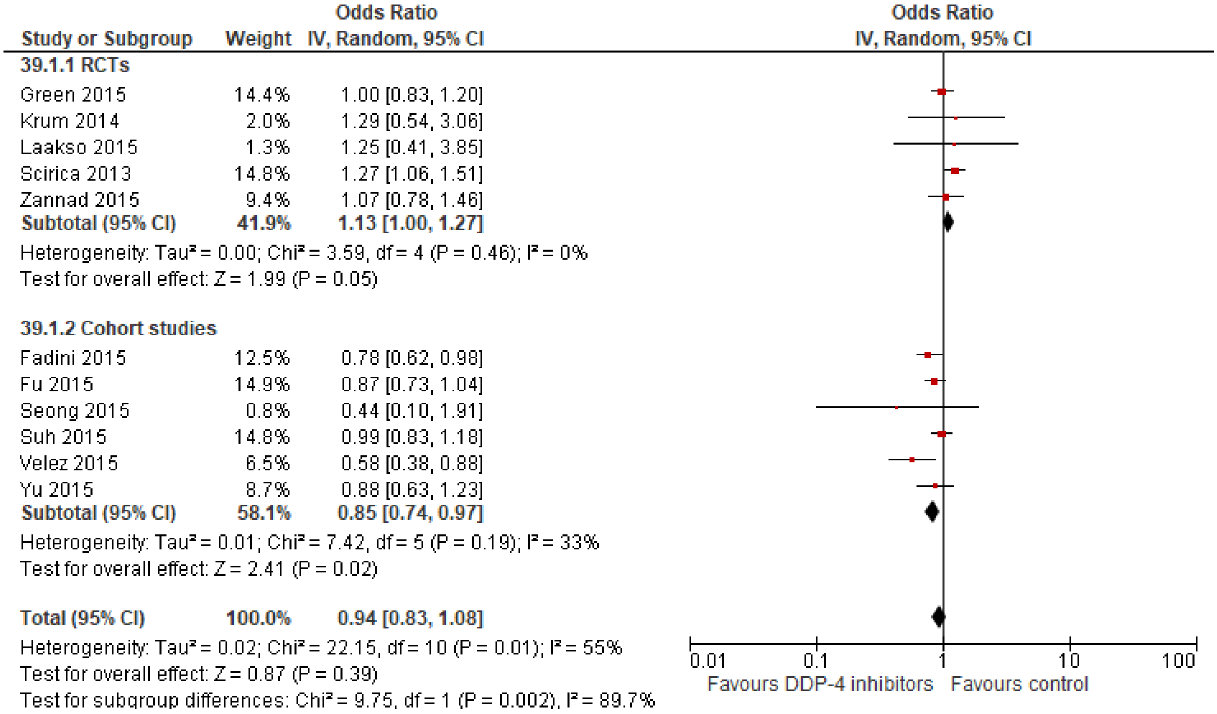

Figure S64: Li 2016; Intervention/ Exposure: DDP-4 inhibitors; Outcome: Hospital admission for heart failure

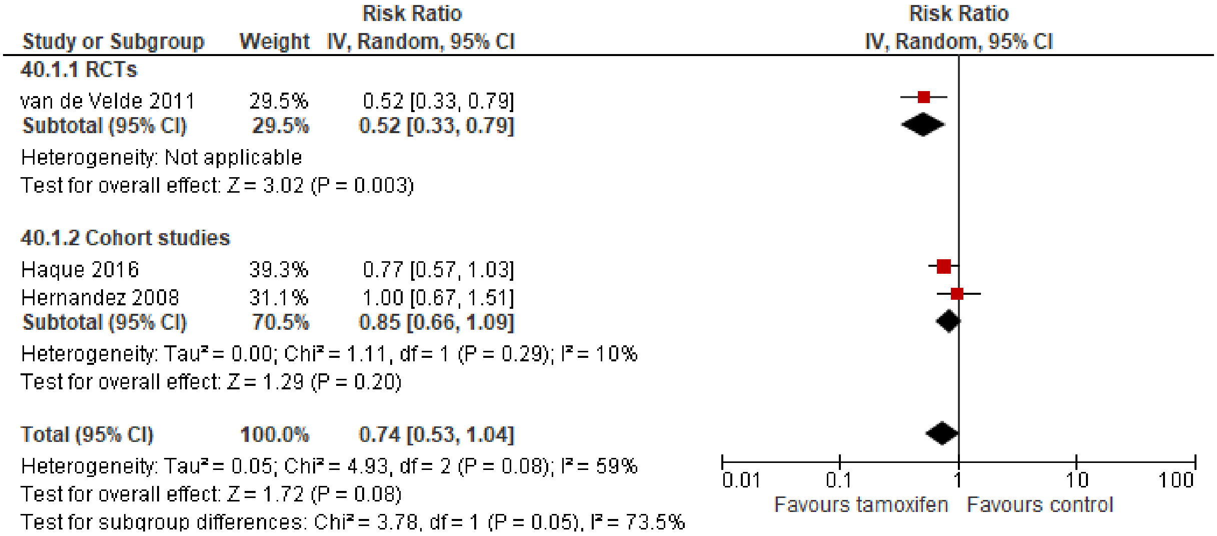

Figure S65: Matthews 2018; Intervention/ Exposure: Tamoxifen; Outcome: Heart failure

Study or Subgroup	Weight	Odds Ratio IV, Random, 95% CI
**41.1.1 RCTs**		
2010-019797-32	0.3%	3.10 [0.13, 76.38]
2014-004599-49	0.3%	1.51 [0.06, 37.25]
Bailey 2013	0.3%	1.01 [0.04, 24.93]
CANTATA-D	0.3%	0.25 [0.01, 6.11]
CANTATA-M	0.3%	1.48 [0.06, 36.38]
CANTATA-SU	0.3%	1.50 [0.06, 36.79]
CANVAS	4.7%	0.76 [0.40, 1.47]
CANVAS R	3.0%	0.47 [0.19, 1.14]
CompoSIT-R	0.3%	3.02 [0.12, 74.41]
CREDENCE	10.4%	0.87 [0.65, 1.17]
DAPA-HF	6.6%	0.49 [0.30, 0.82]
DECLARE-TIMI58	11.8%	0.71 [0.56, 0.89]
DELIGHT	0.3%	0.20 [0.01, 4.23]
DEPICT - 2	0.3%	1.51 [0.06, 37.25]
DEPICT-2		Not estimable
DIA3004	0.5%	1.01 [0.09, 11.24]
DURATION-8	0.5%	0.49 [0.04, 5.46]
EASE-2 and 3	1.3%	0.66 [0.16, 2.77]
EMPA-REG BASAL	0.3%	2.64 [0.13, 55.38]
EMPA-REG H2H-SU	0.3%	0.20 [0.01, 4.24]
EMPA-REG MDI	0.3%	1.51 [0.06, 37.24]
EMPA-REG MONO	0.3%	0.33 [0.01, 8.24]
EMPA-REG RENAL	1.1%	0.76 [0.15, 3.77]
EMPA-REG-MET	0.3%	0.10 [0.00, 2.00]
EMPA-REG-OUTCOME	12.3%	0.78 [0.63, 0.96]
MB102029	0.3%	1.51 [0.06, 37.55]
NCT 01422876	0.3%	0.16 [0.01, 3.97]
NCT 02715258		Not estimable
NCT00528372	0.3%	0.55 [0.02, 13.71]
NCT00660907	0.3%	3.02 [0.12, 74.41]
NCT00663260	0.3%	0.17 [0.01, 4.10]
NCT01031680	0.3%	7.08 [0.36, 137.39]
NCT01042977	0.3%	0.33 [0.01, 8.20]
NCT01422876		Not estimable
NCT01734785	0.3%	0.16 [0.01, 3.99]
NCT02284893	0.3%	2.97 [0.12, 73.38]
NCT02551874	0.3%	0.33 [0.01, 8.06]
NCT02715258	2.2%	1.33 [0.45, 3.94]
PIONEER 2	0.5%	0.50 [0.05, 5.54]
SUSTAIN - 8	0.3%	0.11 [0.01, 2.05]
SUSTAIN-8		Not estimable
VERTIS - SU	0.3%	1.48 [0.06, 36.38]
VERTIS FACTORIAL	0.3%	0.16 [0.01, 4.06]
VERTIS RENAL	0.3%	2.48 [0.12, 51.97]
VERTIS-SU		Not estimable
**Subtotal (95% CI)**	**63.1%**	**0.75 [0.66, 0.84]**
Heterogeneity: Tau² = 0.00; Chi² = 23.65, df = 39 (P = 0.98); I² = 0%		
Test for overall effect: Z = 4.68 (P < 0.00001)		
**41.1.2 Cohort studies**		
Cahn 2019	7.1%	0.26 [0.16, 0.42]
Nadkarni 2017 Geisinger cohort	5.2%	0.41 [0.22, 0.75]
Nadkarni 2017 Mt. sinai cohort	6.0%	0.52 [0.30, 0.90]
Norhammer 2017	10.6%	0.37 [0.28, 0.49]
Ueda 2018	8.0%	0.55 [0.36, 0.83]
**Subtotal (95% CI)**	**36.9%**	**0.40 [0.31, 0.52]**
Heterogeneity: Tau² = 0.03; Chi² = 6.52, df = 4 (P = 0.16); I² = 39%		
Test for overall effect: Z = 7.04 (P < 0.00001)		
**Total (95% CI)**	**100.0%**	**0.58 [0.49, 0.69]**
Heterogeneity: Tau² = 0.05; Chi² = 59.97, df = 44 (P = 0.05); I² = 27%		
Test for overall effect: Z = 6.13 (P < 0.00001)		
Test for subgroup differences: Chi² = 18.30, df = 1 (P < 0.0001), I² = 94.5%		

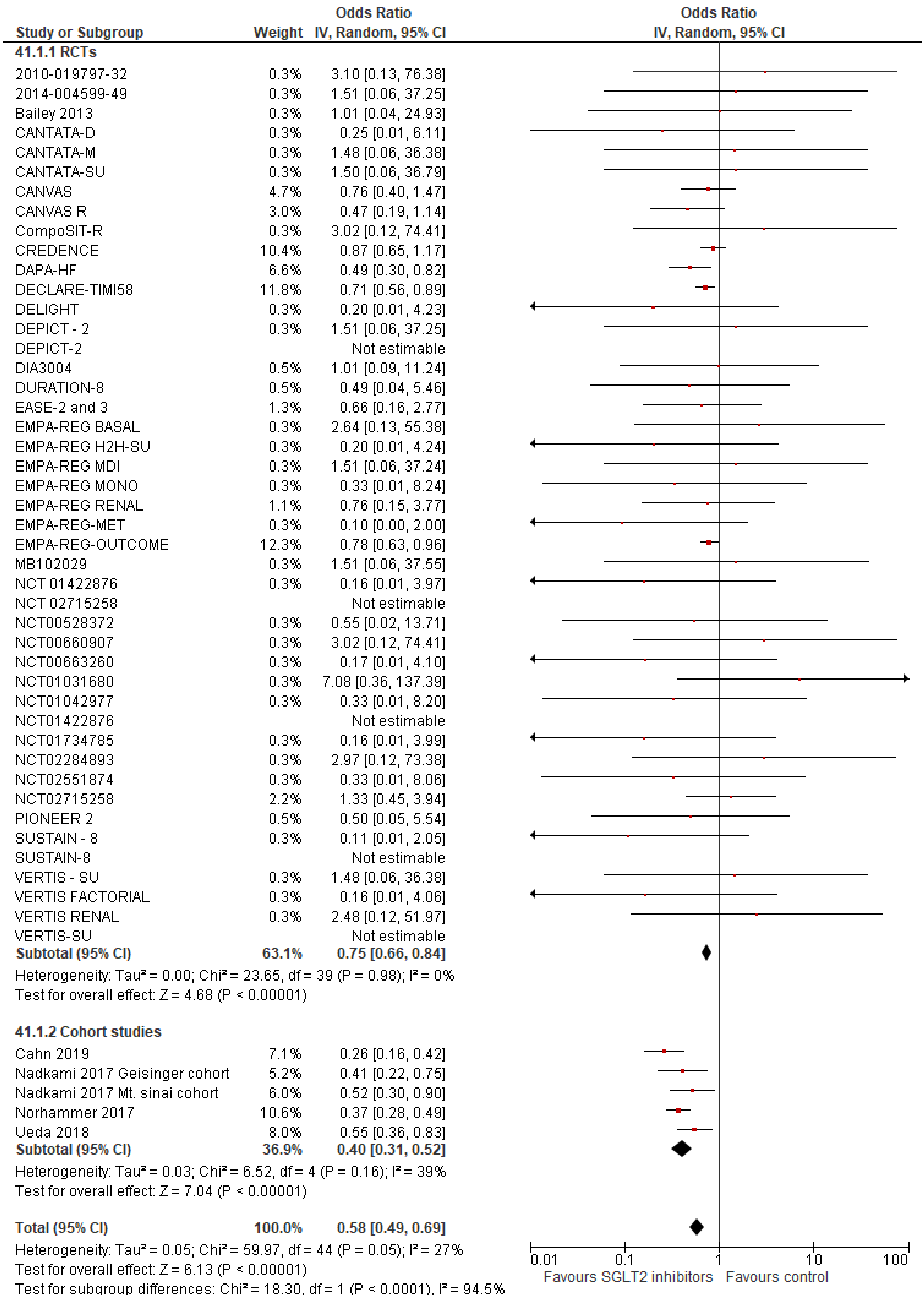

Figure S66: Menne 2019; Intervention/ Exposure: SGLT-2 inhibitors; Outcome: Acute kidney injury

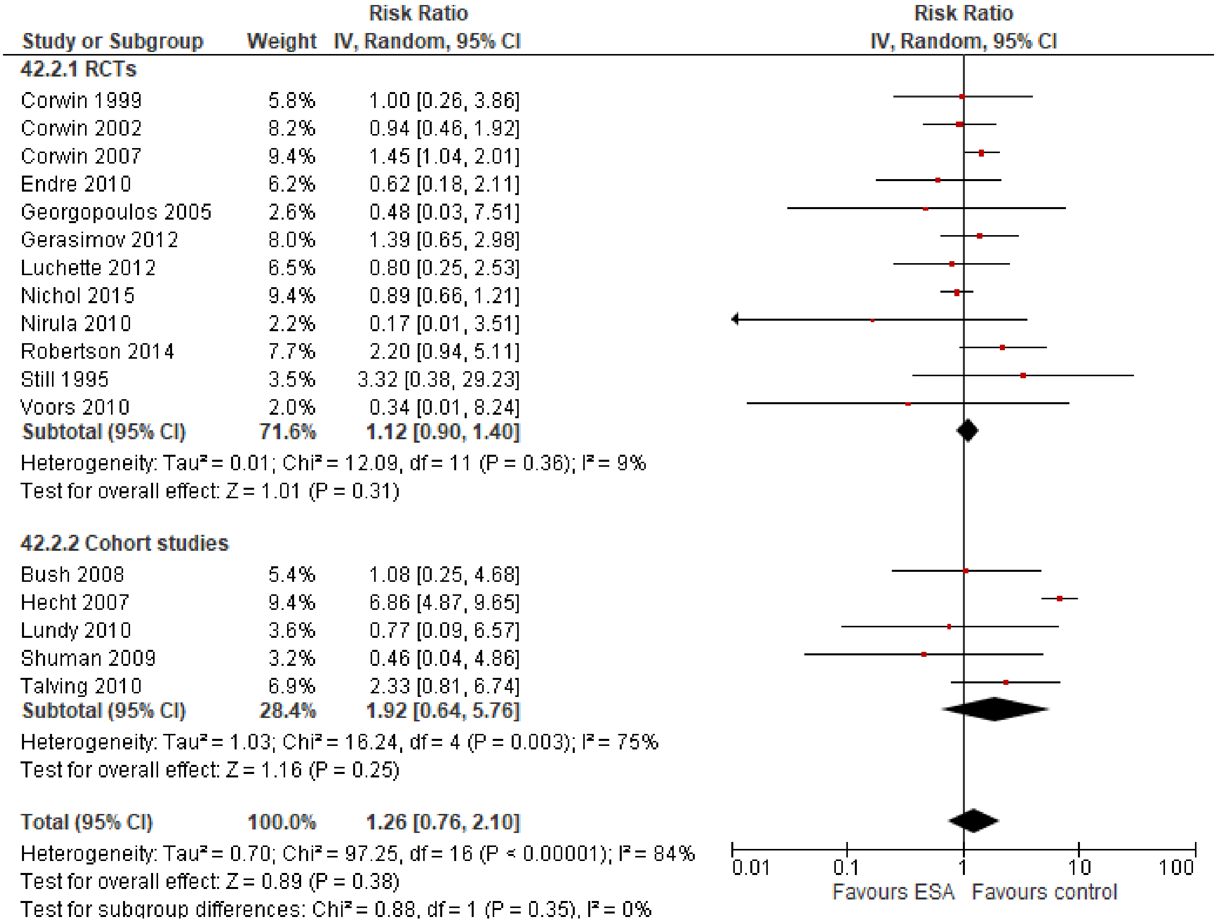

Figure S67: Mesgarpour 2017; Intervention/ Exposure: Erythropoiesis stimulating agents; Outcome: Venous thromboembolism

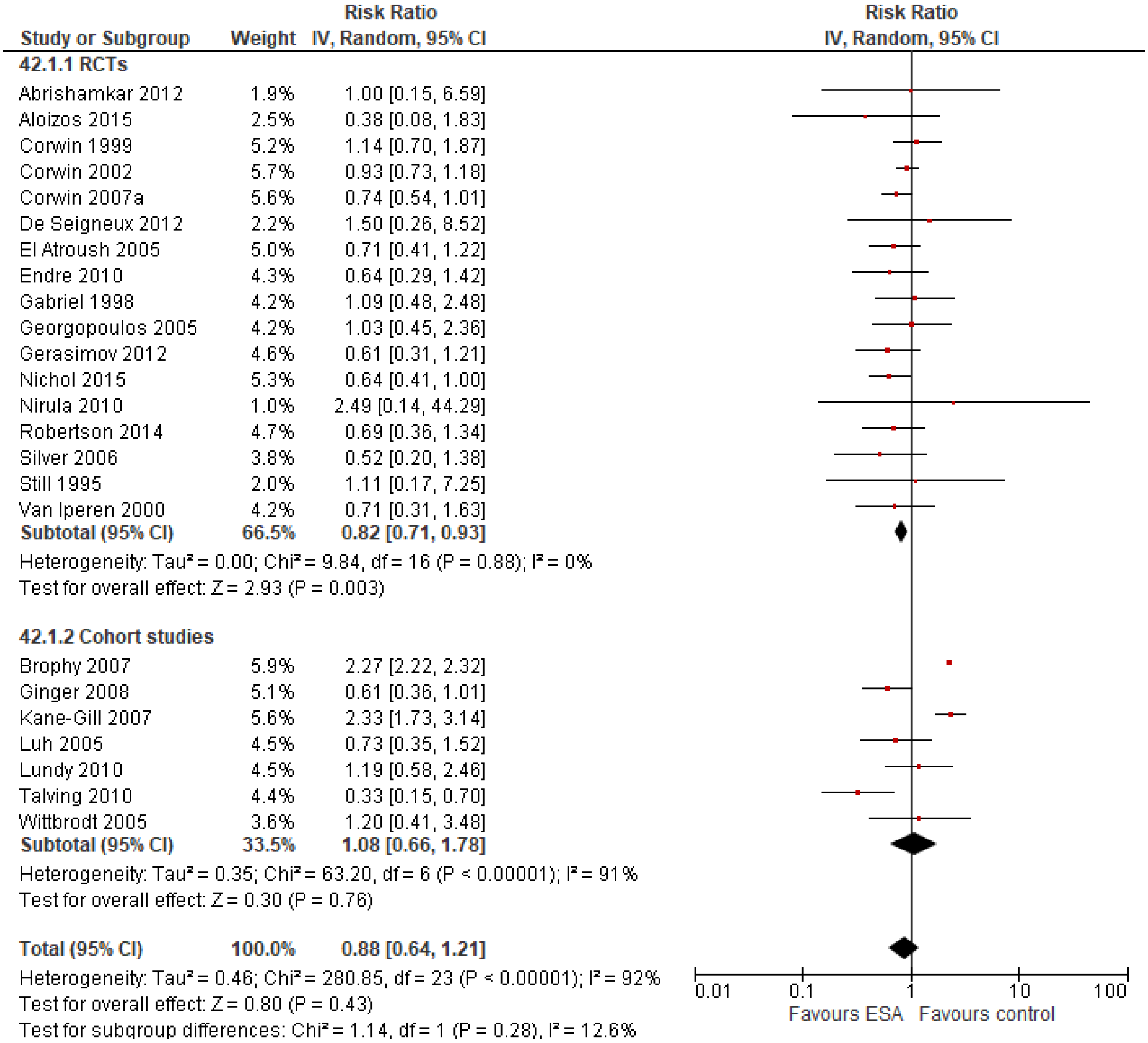

Figure S68: Mesgarpour 2017; Intervention/ Exposure: Erythropoiesis stimulating agents; Outcome: All-cause mortality

Study or Subgroup	Weight	Odds Ratio IV, Random, 95% CI
**43.1.1 RCTs**		
Austrian 1980b	2.2%	0.11 [0.01, 2.07]
Riley 1977	7.7%	0.14 [0.03, 0.61]
Maruyama 2010	2.1%	0.14 [0.01, 2.77]
Ortqvist 1998	3.9%	0.21 [0.02, 1.77]
Kaufman 1947	22.1%	0.21 [0.10, 0.45]
Gaillat 1985	1.8%	0.27 [0.01, 6.54]
Klastersky 1986	2.3%	0.80 [0.05, 13.60]
Simberkoff 1986	2.4%	1.00 [0.06, 16.08]
Leech 1987	1.8%	3.20 [0.13, 79.48]
Davis 1987	1.8%	3.24 [0.13, 81.47]
**Subtotal (95% CI)**	**48.1%**	**0.26 [0.14, 0.45]**
Heterogeneity: Tau² = 0.00; Chi² = 7.71, df = 9 (P = 0.56); I² = 0%		
Test for overall effect: Z = 4.74 (P < 0.00001)		
**43.1.2 Cohort studies**		
Jackson 2003	36.7%	0.56 [0.34, 0.93]
Vila-Corcoles 2006	15.3%	0.60 [0.22, 1.61]
**Subtotal (95% CI)**	**51.9%**	**0.57 [0.36, 0.89]**
Heterogeneity: Tau² = 0.00; Chi² = 0.01, df = 1 (P = 0.91); I² = 0%		
Test for overall effect: Z = 2.45 (P = 0.01)		
**Total (95% CI)**	**100.0%**	**0.40 [0.26, 0.61]**
Heterogeneity: Tau² = 0.07; Chi² = 12.49, df = 11 (P = 0.33); I² = 12%		
Test for overall effect: Z = 4.15 (P < 0.0001)		
Test for subgroup differences: Chi² = 4.77, df = 1 (P = 0.03), I² = 79.0%		

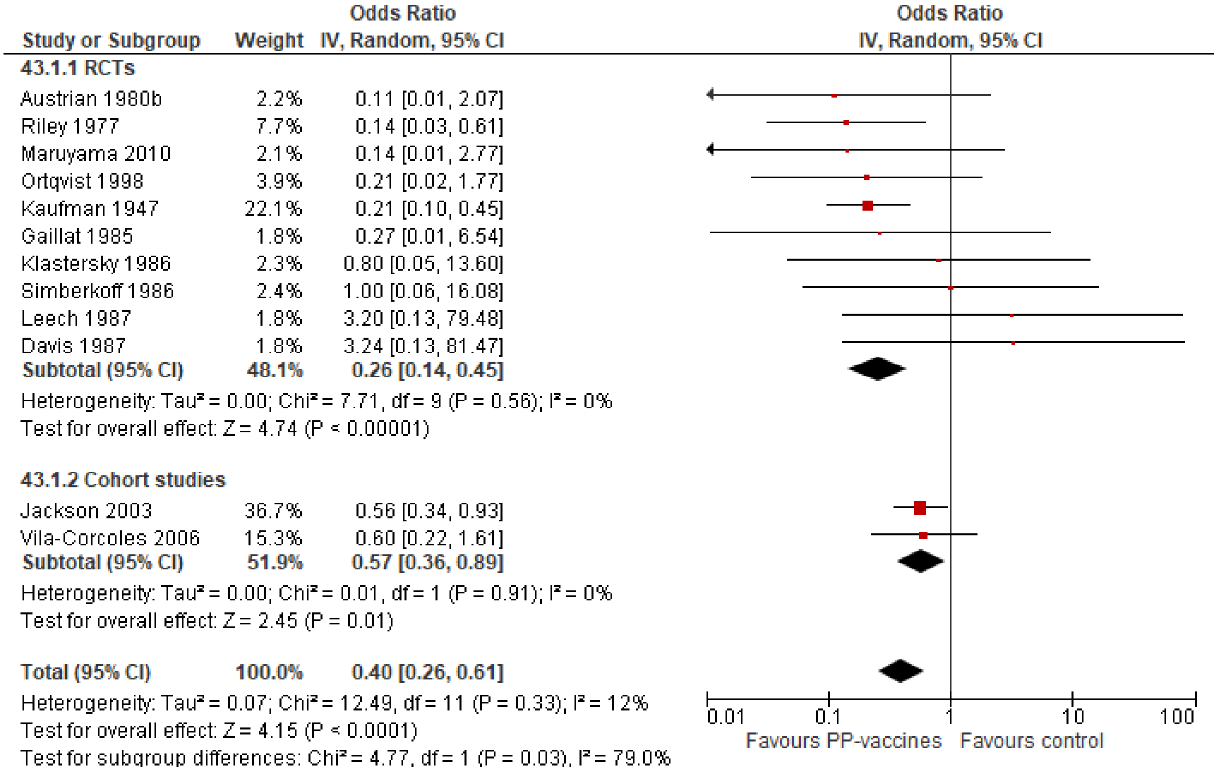

Figure S69: Moberley 2013; Intervention/ Exposure: Pneumococcal polysaccharide vaccines; Outcome: Invasive pneumococcal disease

Study or Subgroup	Weight	Odds Ratio IV, Random, 95% CI	Odds Ratio IV, Random, 95% CI
**44.2.1 RCTs**			
Khatami 2013	31.5%	1.01 [0.48, 2.14]	
Stephan 1998	18.1%	2.31 [0.60, 8.94]	
**Subtotal (95% CI)**	**49.6%**	**1.25 [0.61, 2.56]**	
Heterogeneity: Tau² = 0.03; Chi² = 1.10, df = 1 (P = 0.29); I² = 9%			
Test for overall effect: Z = 0.62 (P = 0.53)			
**44.2.2 Cohort studies**			
Spasovski 2008	15.3%	0.22 [0.05, 1.03]	
Taber 2005	35.1%	0.53 [0.28, 0.99]	
**Subtotal (95% CI)**	**50.4%**	**0.46 [0.25, 0.86]**	
Heterogeneity: Tau² = 0.02; Chi² = 1.06, df = 1 (P = 0.30); I² = 5%			
Test for overall effect: Z = 2.43 (P = 0.02)			
**Total (95% CI)**	**100.0%**	**0.74 [0.36, 1.54]**	
Heterogeneity: Tau² = 0.29; Chi² = 6.85, df = 3 (P = 0.08); I² = 56%			
Test for overall effect: Z = 0.80 (P = 0.42)			
Test for subgroup differences: Chi² = 4.29, df = 1 (P = 0.04), I² = 76.7%			

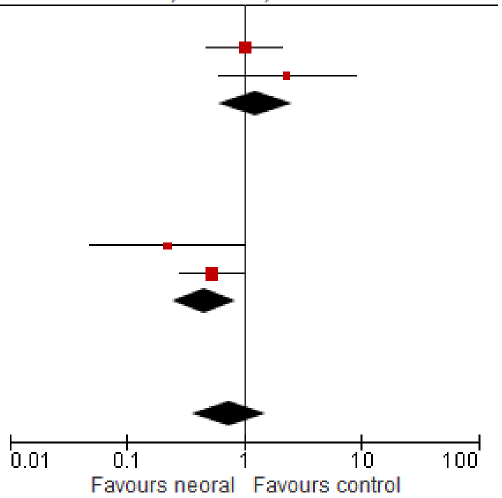

Figure S70: Molnar 2015; Intervention/ Exposure: Neoral (Cyclosporin); Outcome: Acute rejection of kidney transplant

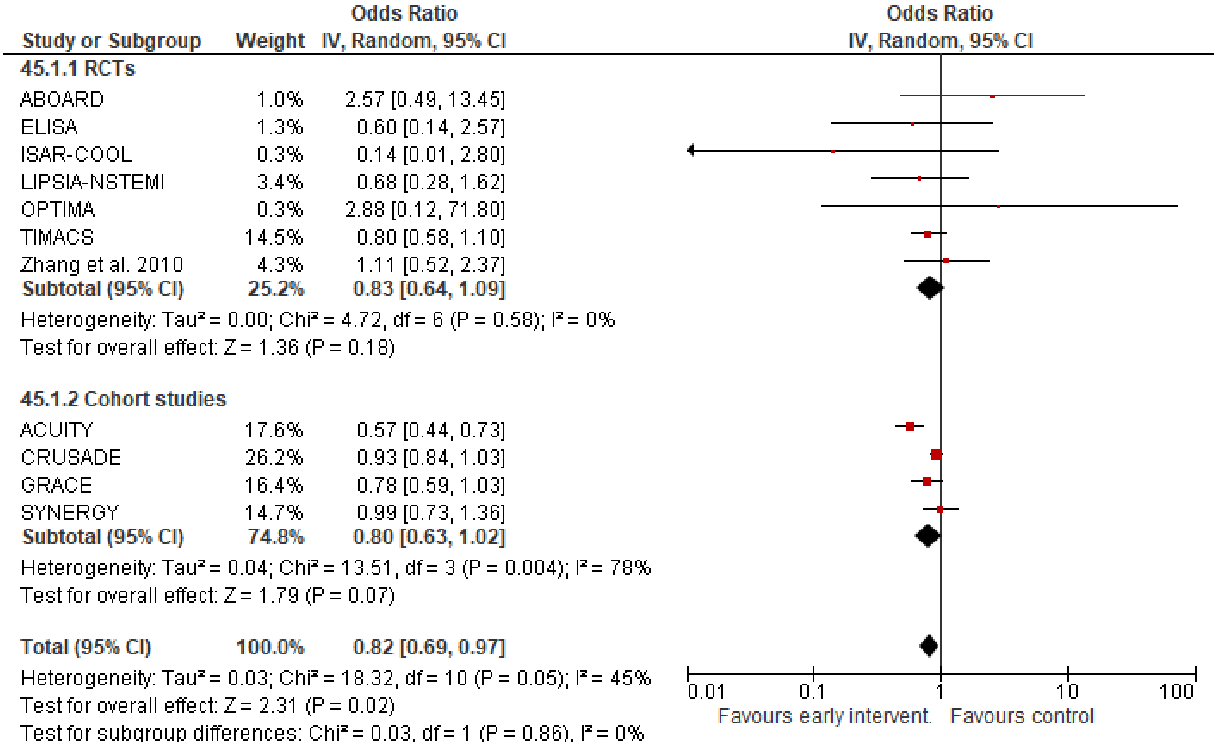

Figure S71: Navarese 2013; Intervention/ Exposure: Early intervention for NSTE-ACS; Outcome: All-cause mortality

Study or Subgroup	Weight	Odds Ratio IV, Random, 95% CI
**45.2.1 RCTs**		
ABOARD	4.9%	2.13 [0.89, 5.10]
ELISA	3.4%	1.20 [0.39, 3.70]
ISAR-COOL	6.2%	0.56 [0.27, 1.16]
LIPSIA-NSTEMI	7.0%	2.84 [1.45, 5.58]
OPTIMA	7.0%	2.36 [1.20, 4.63]
TIMACS	13.1%	0.83 [0.60, 1.14]
Zhang et al. 2010	9.0%	0.45 [0.26, 0.76]
**Subtotal (95% CI)**	**50.6%**	**1.16 [0.67, 2.00]**
Heterogeneity: Tau² = 0.41; Chi² = 30.88, df = 6 (P < 0.0001); I² = 81%		
Test for overall effect: Z = 0.54 (P = 0.59)		
**45.2.2 Cohort studies**		
ACUITY	16.3%	0.70 [0.60, 0.82]
CRUSADE	16.8%	1.04 [0.91, 1.17]
SYNERGY	16.4%	0.87 [0.75, 1.00]
**Subtotal (95% CI)**	**49.4%**	**0.86 [0.69, 1.08]**
Heterogeneity: Tau² = 0.03; Chi² = 14.64, df = 2 (P = 0.0007); I² = 86%		
Test for overall effect: Z = 1.32 (P = 0.19)		
**Total (95% CI)**	**100.0%**	**0.97 [0.77, 1.22]**
Heterogeneity: Tau² = 0.08; Chi² = 46.30, df = 9 (P < 0.00001); I² = 81%		
Test for overall effect: Z = 0.29 (P = 0.78)		
Test for subgroup differences: Chi² = 1.00, df = 1 (P = 0.32), I² = 0%		

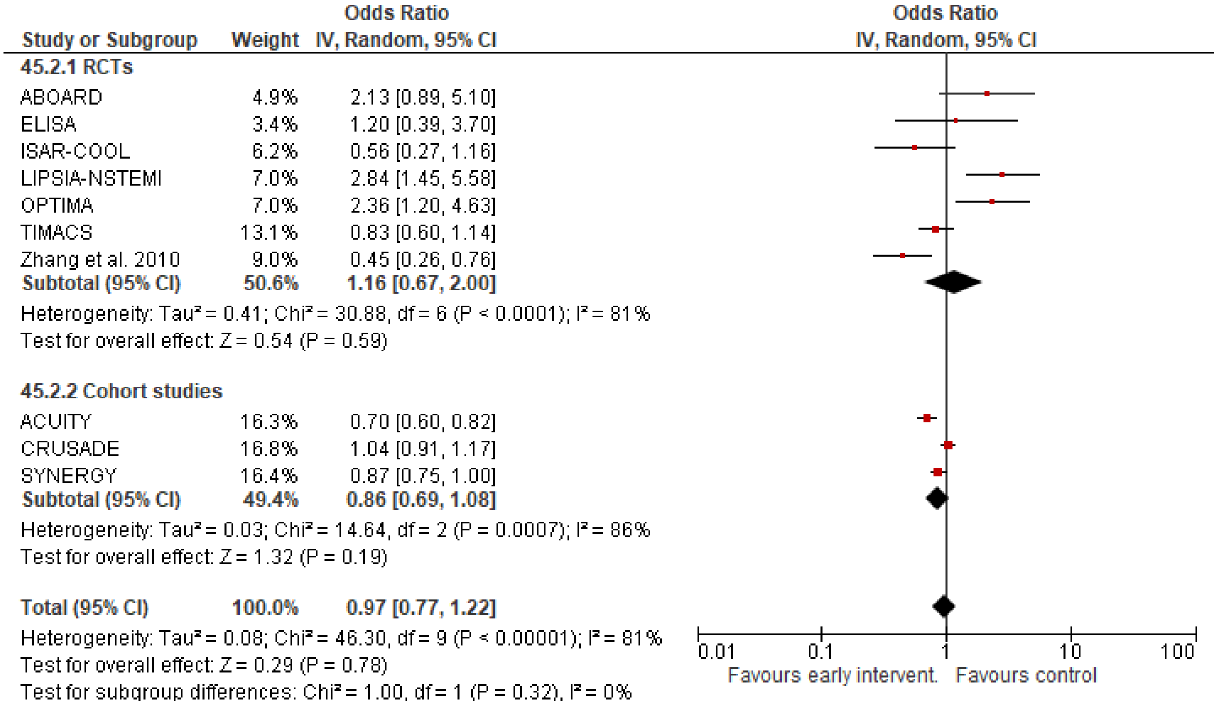

Figure S72: Navarese 2013; Intervention/ Exposure: Early intervention for NSTE-ACS; Outcome: Myocardial infarction

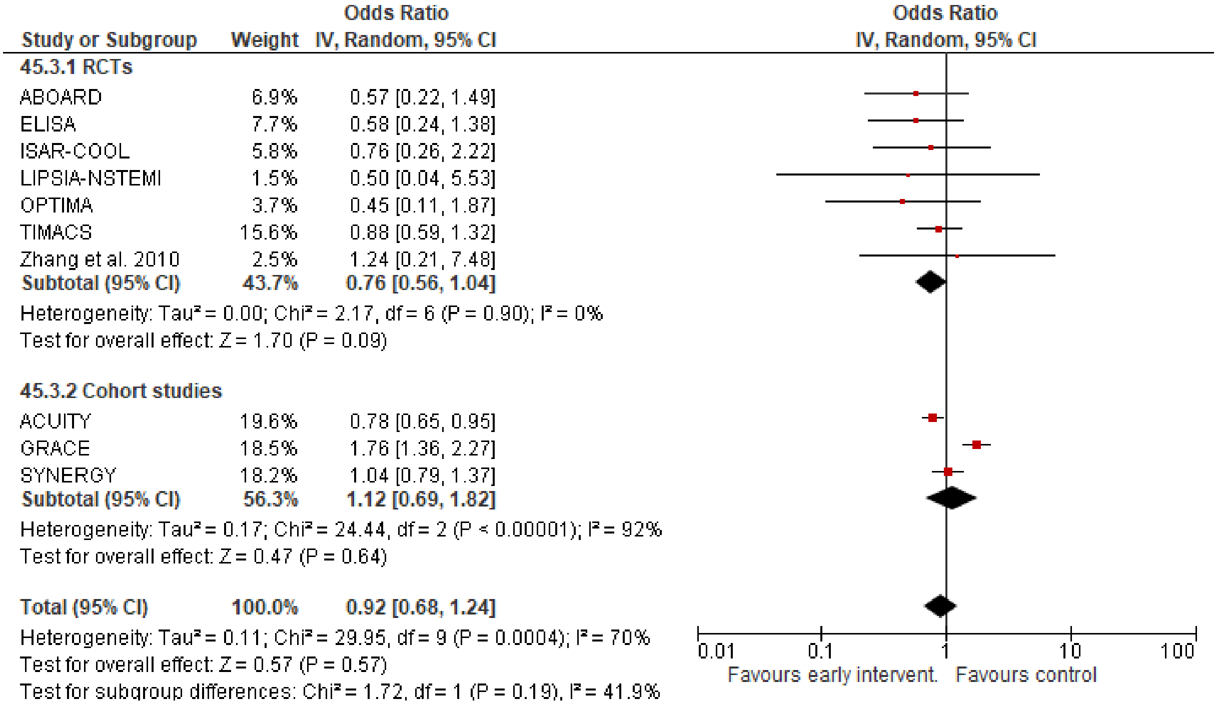

Figure S73: Navarese 2013; Intervention/ Exposure: Early intervention for NSTE-ACS; Outcome: Major bleeding

Study or Subgroup	Weight	Odds Ratio IV, Random, 95% CI
**46.1.1 RCTs**		
Hannah 2002	3.0%	0.81 [0.22, 2.97]
Hannah 2004	7.0%	1.10 [0.47, 2.58]
**Subtotal (95% CI)**	**10.0%**	**1.00 [0.49, 2.05]**
Heterogeneity: Tau² = 0.00; Chi² = 0.15, df = 1 (P = 0.70); I² = 0%		
Test for overall effect: Z = 0.01 (P = 0.99)		
**46.1.2 Cohort studies**		
Altman 2007	1.0%	1.04 [0.11, 10.04]
Burgio 2007	1.3%	1.01 [0.14, 7.14]
Casey 2005	1.4%	0.58 [0.08, 3.93]
Chaliha 2001	5.0%	1.56 [0.57, 4.29]
Eason 2002	20.1%	0.81 [0.49, 1.34]
Fritel 2008	1.6%	1.22 [0.21, 7.12]
Lal 2003	7.8%	0.61 [0.27, 1.37]
MacArthur 1997	6.4%	0.79 [0.32, 1.93]
MacArthur 2005	37.6%	1.04 [0.72, 1.50]
MacArthur 2011	4.5%	0.93 [0.32, 2.69]
McKinnie 2005	1.8%	0.52 [0.10, 2.81]
Torrosi 2007	1.5%	0.82 [0.13, 5.14]
**Subtotal (95% CI)**	**90.0%**	**0.91 [0.72, 1.16]**
Heterogeneity: Tau² = 0.00; Chi² = 3.64, df = 11 (P = 0.98); I² = 0%		
Test for overall effect: Z = 0.74 (P = 0.46)		
**Total (95% CI)**	**100.0%**	**0.92 [0.74, 1.16]**
Heterogeneity: Tau² = 0.00; Chi² = 3.85, df = 13 (P = 0.99); I² = 0%		
Test for overall effect: Z = 0.70 (P = 0.49)		
Test for subgroup differences: Chi² = 0.06, df = 1 (P = 0.81), I² = 0%		

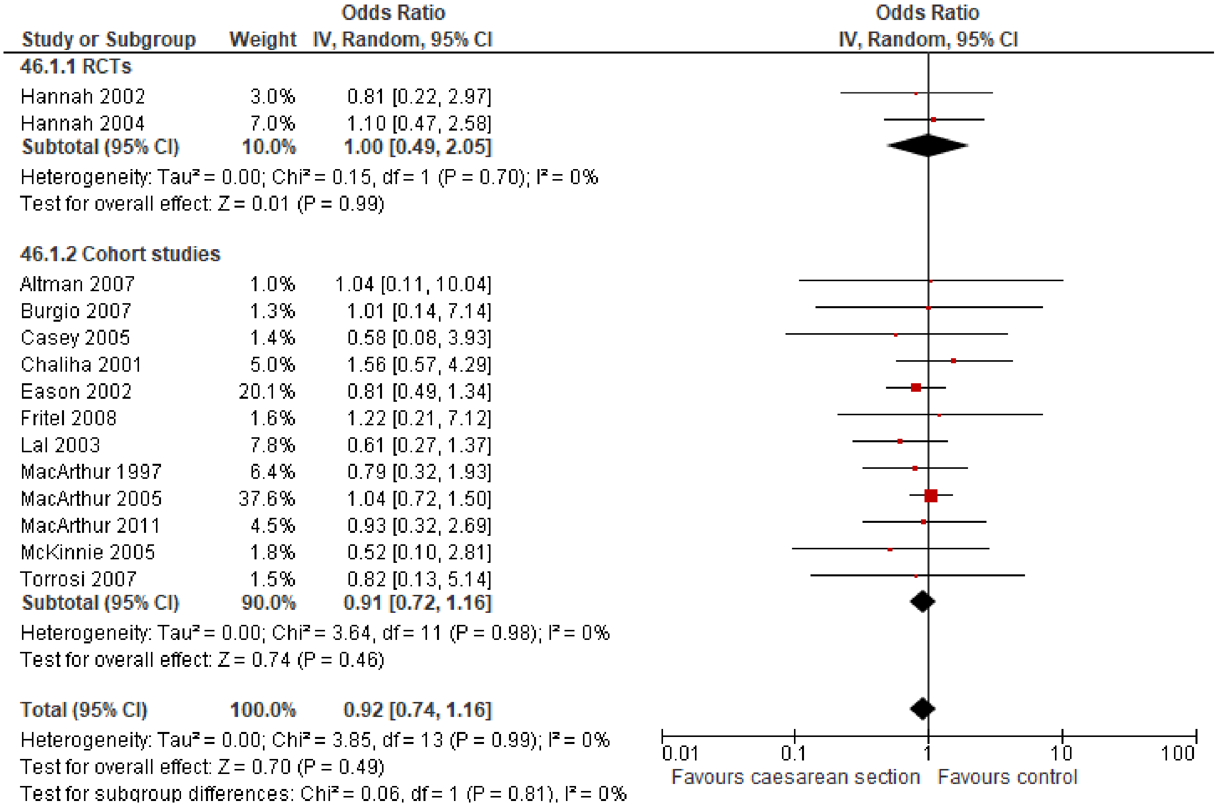

Figure S74: Nelson 2010; Intervention/ Exposure: Caesarean section; Outcome: Anal incontinence, feces

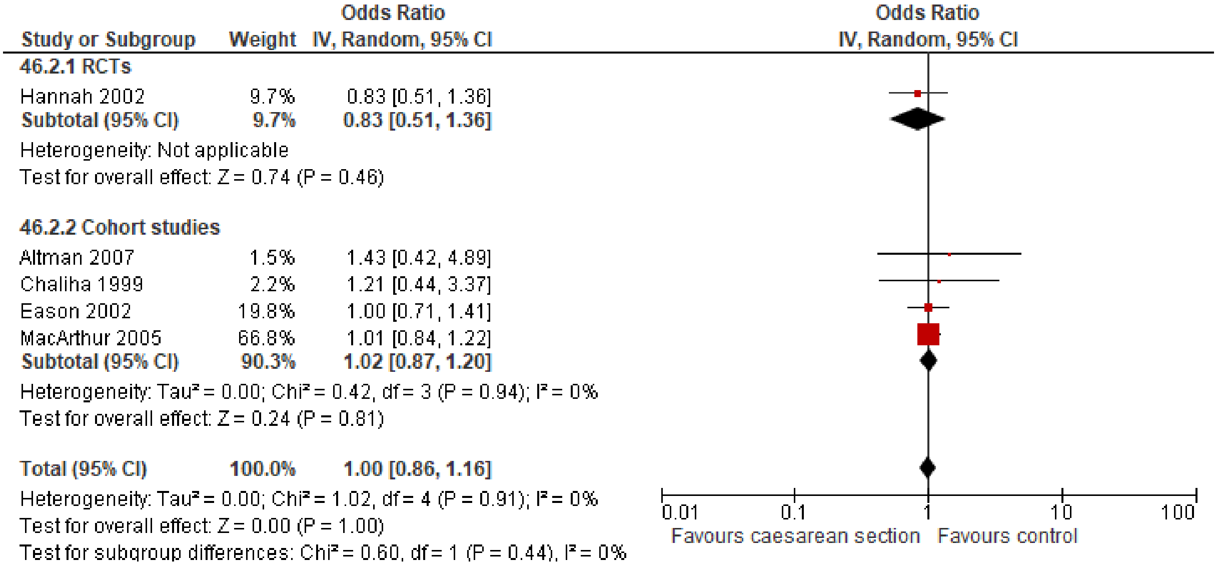

Figure S75: Nelson 2010; Intervention/ Exposure: Caesarean section; Outcome: Anal incontinence, flatus

Study or Subgroup	Weight	Mean Difference IV, Random, 95% CI
**47.4.1 RCTs**		
Amantullah 2011		Not estimable
Bal 2005		Not estimable
Bascarevic 2010	14.2%	1.30 [-0.09, 2.69]
Capello 2005 (A)		Not estimable
D'Antonio 2002		Not estimable
D'Antonio 2012	10.7%	0.00 [-1.72, 1.72]
Hamilton 2010		Not estimable
Kim 2009	20.2%	-1.00 [-2.00, -0.00]
Kim 2013	6.1%	-1.00 [-3.47, 1.47]
Nikolau 2012	2.0%	-0.50 [-5.08, 4.08]
Ochs 2007	0.4%	2.00 [-8.41, 12.41]
Poggie 2007	5.6%	-1.00 [-3.61, 1.61]
Schwämmlein 2002		Not estimable
**Subtotal (95% CI)**	**59.3%**	**-0.23 [-1.09, 0.63]**
Heterogeneity: Tau² = 0.31; Chi² = 7.87, df = 6 (P = 0.25); I² = 24%		
Test for overall effect: Z = 0.52 (P = 0.60)		
**47.4.2 Cohort studies**		
Epinette 2014	26.5%	0.20 [-0.49, 0.89]
Plate 2013	1.2%	1.00 [-5.08, 7.08]
Schmidt 2002	13.0%	-1.70 [-3.19, -0.21]
**Subtotal (95% CI)**	**40.7%**	**-0.50 [-2.09, 1.09]**
Heterogeneity: Tau² = 1.08; Chi² = 5.28, df = 2 (P = 0.07); I² = 62%		
Test for overall effect: Z = 0.62 (P = 0.54)		
**Total (95% CI)**	**100.0%**	**-0.29 [-0.96, 0.38]**
Heterogeneity: Tau² = 0.31; Chi² = 13.27, df = 9 (P = 0.15); I² = 32%		
Test for overall effect: Z = 0.86 (P = 0.39)		
Test for subgroup differences: Chi² = 0.09, df = 1 (P = 0.77), I² = 0%		

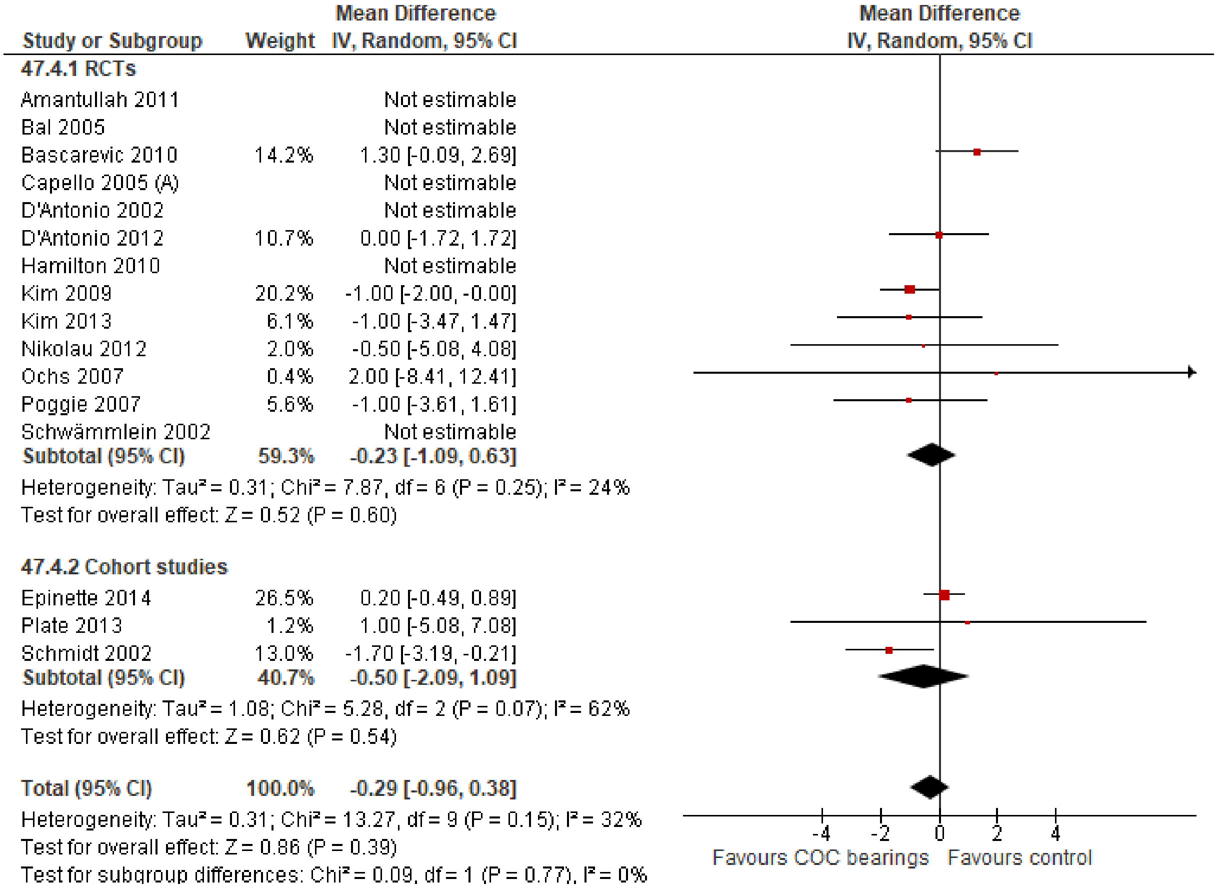

Figure S76: Nieuwenhuijse 2014; Intervention/ Exposure: Ceramic-on-ceramic bearings for total hip arthroplasty; Outcome: Harris Hip Score

Study or Subgroup	Weight	Mean Difference IV, Random, 95% CI
**47.5.1 RCTs**		
Ahmed 2009	1.5%	3.00 [-5.39, 11.39]
Biant 2009		Not estimable
Choi 2010	2.9%	-2.00 [-5.63, 1.63]
Dennis 2013	3.2%	1.00 [-1.93, 3.93]
Fisher 2013	2.2%	-4.00 [-9.84, 1.84]
Guild 2014	3.6%	-0.10 [-1.66, 1.46]
Hamilton 2011	2.1%	0.20 [-5.92, 6.32]
Kim 2005	1.8%	3.00 [-4.11, 10.11]
Kim 2009	3.2%	2.00 [-0.84, 4.84]
Kim 2012 (B)	2.7%	2.00 [-2.47, 6.47]
Lutzer 2014	2.6%	0.80 [-3.96, 5.56]
McCalden 2009	1.6%	1.00 [-7.17, 9.17]
Nieuwenhuijse 2013	1.7%	-2.30 [-9.75, 5.15]
Nutton 2012	2.3%	6.00 [0.61, 11.39]
Radetzki 2013	2.1%	0.70 [-5.45, 6.85]
Seng 2011	2.0%	11.00 [4.57, 17.43]
Seon 2009	3.0%	1.00 [-2.37, 4.37]
Thomsen 2013	2.8%	7.00 [2.96, 11.04]
Weeden 2007	0.9%	13.00 [0.38, 25.62]
Wohlrab 2005	2.4%	3.00 [-2.33, 8.33]
Wohlrab 2009	2.1%	-0.80 [-6.94, 5.34]
**Subtotal (95% CI)**	**46.8%**	**1.68 [0.28, 3.08]**
Heterogeneity: Tau² = 3.83; Chi² = 34.67, df = 19 (P = 0.02); I² = 45%		
Test for overall effect: Z = 2.35 (P = 0.02)		
**47.5.2 Cohort studies**		
Bajammal 2006	3.1%	0.10 [-3.17, 3.37]
Boese 2011	2.6%	-1.70 [-6.31, 2.91]
Coughlin 2007	2.1%	-1.00 [-7.31, 5.31]
Crow 2010	3.0%	7.80 [4.47, 11.13]
Endres 2011	2.7%	2.00 [-2.39, 6.39]
Goebel 2008	2.4%	15.00 [9.71, 20.29]
Gupta 2006	2.8%	9.00 [5.06, 12.94]
Huang 2005	3.0%	12.00 [8.39, 15.61]
Kim 2012	1.3%	10.00 [0.63, 19.37]
Klein 2004	2.6%	5.40 [0.72, 10.08]
Kwak 2012	2.0%	-6.40 [-13.08, 0.28]
Lee 2011	2.5%	10.00 [5.18, 14.82]
Lee 2012	2.3%	-1.00 [-6.51, 4.51]
Li 2007		Not estimable
Malik 2010	2.5%	2.00 [-2.94, 6.94]
Minoda 2010	2.5%	-0.50 [-5.48, 4.48]
Nam 2012	1.8%	1.30 [-6.03, 8.63]
Ng 2008	3.4%	1.00 [-1.31, 3.31]
Seon 2005	1.9%	2.20 [-4.60, 9.00]
Stormont 2009	2.7%	5.40 [1.13, 9.67]
Suggs 2009	1.6%	-2.90 [-10.92, 5.12]
Tsai 2009	1.6%	4.10 [-4.15, 12.35]
Tsuji 2009	2.9%	5.70 [2.06, 9.34]
**Subtotal (95% CI)**	**53.2%**	**3.78 [1.64, 5.92]**
Heterogeneity: Tau² = 19.12; Chi² = 96.70, df = 21 (P < 0.00001); I² = 78%		
Test for overall effect: Z = 3.46 (P = 0.0005)		
**Total (95% CI)**	**100.0%**	**2.91 [1.56, 4.27]**
Heterogeneity: Tau² = 12.70; Chi² = 149.43, df = 41 (P < 0.00001); I² = 73%		
Test for overall effect: Z = 4.23 (P < 0.0001)		
Test for subgroup differences: Chi² = 2.58, df = 1 (P = 0.11), I² = 61.3%		

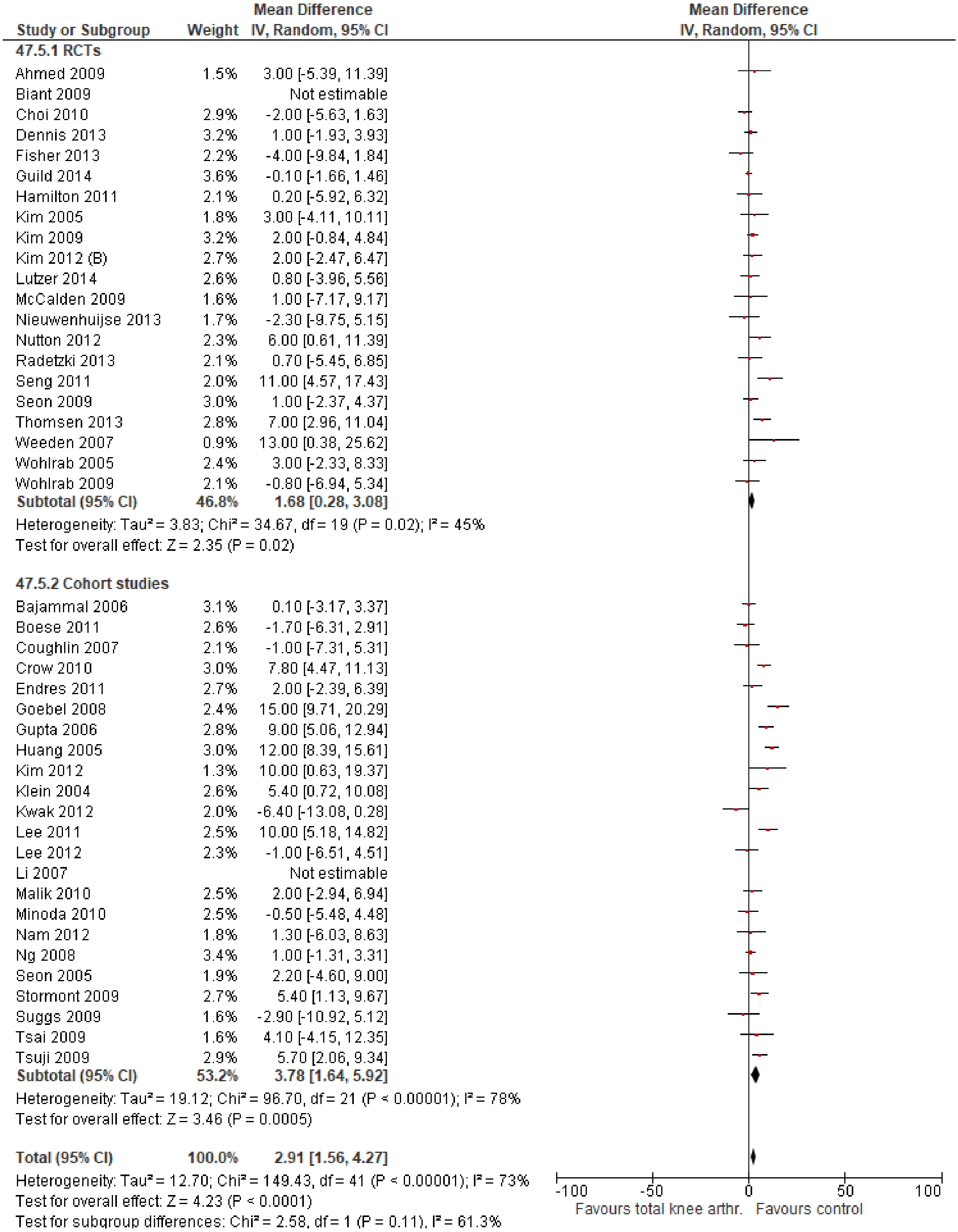

Figure S77: Nieuwenhuijse 2014; Intervention/ Exposure: High-flexion total knee arthroplasty; Outcome: Flexion in degrees

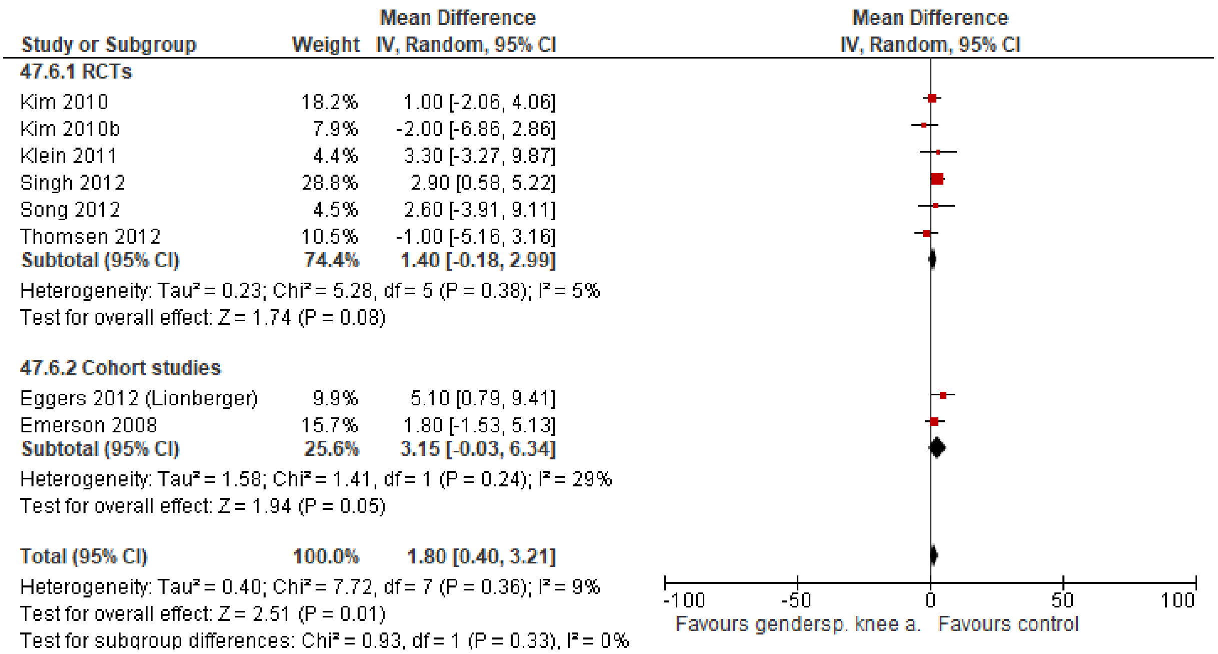

Figure S78: Nieuwenhuijse 2014; Intervention/ Exposure: Gender-specific total knee arthroplasty; Outcome: Flexion-extension range

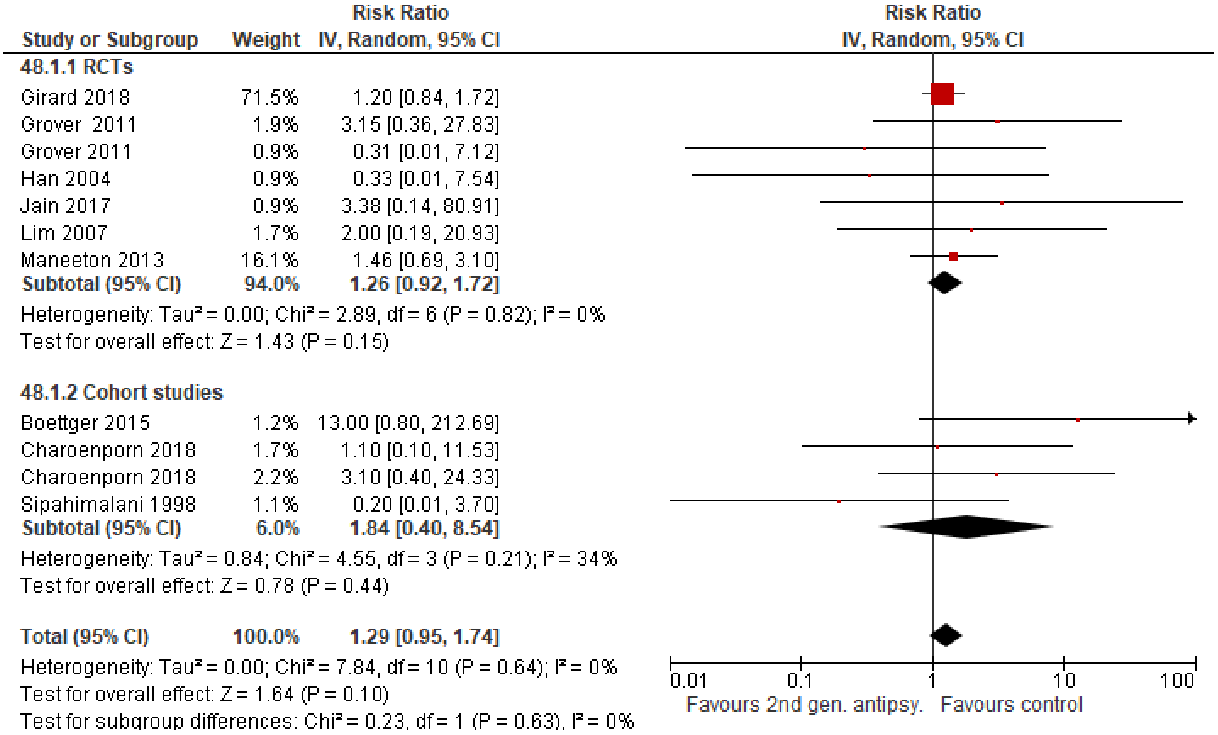

Figure S79: Nikooie 2019; Intervention/ Exposure: Second generation antipsychotics; Outcome: Sedation

Study or Subgroup	Weight	Risk Ratio IV, Random, 95% CI
**48.2.1 RCTs**		
Girard 2010	2.3%	0.58 [0.11, 2.96]
Girard 2018	1.1%	0.51 [0.05, 5.53]
Grover 2011	3.7%	0.49 [0.14, 1.75]
Han 2004	0.6%	0.33 [0.01, 7.45]
Lim 2007	0.7%	0.14 [0.01, 2.65]
Maneeton 2013	0.6%	0.39 [0.02, 9.07]
**Subtotal (95% CI)**	**9.0%**	**0.45 [0.20, 1.01]**
Heterogeneity: Tau² = 0.00; Chi² = 0.76, df = 5 (P = 0.98); I² = 0%		
Test for overall effect: Z = 1.93 (P = 0.05)		
**48.2.2 Cohort studies**		
Boettger 2011	1.1%	0.30 [0.03, 3.00]
Boettger 2015	0.7%	0.20 [0.01, 3.90]
Boettger 2015	1.6%	0.30 [0.04, 2.10]
Boettger 2015	0.7%	0.20 [0.01, 3.90]
Boettger 2015	0.7%	0.10 [0.00, 1.90]
Boettger 2015	0.7%	0.20 [0.01, 3.90]
Boettger 2015	0.7%	0.10 [0.00, 1.90]
Charoenporn 2018	1.4%	1.60 [0.20, 13.19]
Charoenporn 2018	1.3%	1.10 [0.13, 9.20]
Hatta 2014	28.3%	0.70 [0.44, 1.10]
Hatta 2014	5.6%	0.70 [0.25, 2.00]
Hatta 2014	44.8%	1.00 [0.70, 1.45]
Hatta 2014	2.9%	0.50 [0.12, 2.10]
Sipahimalani 1998	0.6%	0.10 [0.00, 2.50]
**Subtotal (95% CI)**	**91.0%**	**0.76 [0.59, 0.99]**
Heterogeneity: Tau² = 0.00; Chi² = 12.42, df = 13 (P = 0.49); I² = 0%		
Test for overall effect: Z = 2.04 (P = 0.04)		
**Total (95% CI)**	**100.0%**	**0.73 [0.57, 0.93]**
Heterogeneity: Tau² = 0.00; Chi² = 14.68, df = 19 (P = 0.74); I² = 0%		
Test for overall effect: Z = 2.53 (P = 0.01)		
Test for subgroup differences: Chi² = 1.50, df = 1 (P = 0.22), I² = 33.2%		

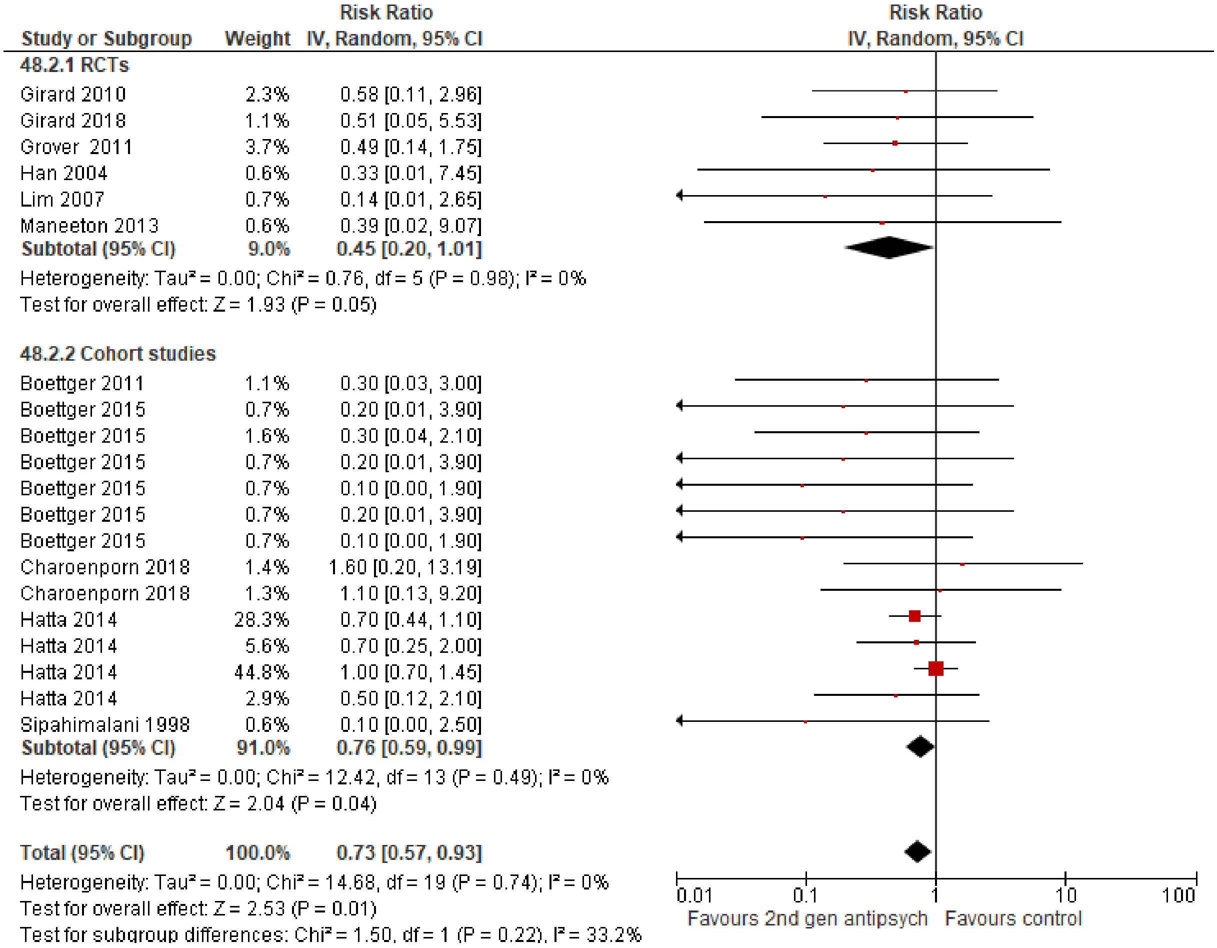

Figure S80: Nikooie 2019; Intervention/ Exposure: Second generation antipsychotics; Outcome: Neurologic outcomes

Study or Subgroup	Weight	Risk Ratio IV, Random, 95% CI
**49.1.1 RCTs**		
Cetti 1993	5.0%	0.37 [0.10, 1.32]
Keating 2011	3.3%	0.53 [0.10, 2.71]
Lantto 2016	2.1%	0.22 [0.03, 1.84]
Metz 2008	4.5%	0.59 [0.15, 2.29]
Möller 2001	2.3%	0.08 [0.01, 0.61]
Nilsson-Helander 2010	3.7%	0.33 [0.07, 1.54]
Nistor 1981	3.5%	0.53 [0.11, 2.61]
Olsson 2013	1.2%	0.09 [0.01, 1.67]
Twaddle 2007	1.8%	2.00 [0.19, 20.67]
Willits 2010	3.0%	0.67 [0.11, 3.87]
**Subtotal (95% CI)**	**30.4%**	**0.40 [0.24, 0.69]**
Heterogeneity: Tau² = 0.00; Chi² = 6.43, df = 9 (P = 0.70); I² = 0%		
Test for overall effect: Z = 3.28 (P = 0.001)		
**49.1.2 Cohort studies**		
Bergkvist 2012	8.2%	0.38 [0.16, 0.94]
Costa 2006	2.5%	1.00 [0.15, 6.81]
Cukelj 2015	1.2%	0.07 [0.00, 1.36]
Ebinesan 2008	1.4%	0.24 [0.02, 3.50]
Fahlström 1998	1.2%	0.09 [0.00, 1.65]
Gubor 2012	5.0%	0.18 [0.05, 0.64]
Gwynne-Jones 2011	4.1%	0.16 [0.04, 0.68]
Jaakkola 2001	1.2%	0.15 [0.01, 2.89]
Jackson 2013	1.7%	0.88 [0.08, 9.28]
Kotnis 2006	1.7%	0.43 [0.04, 4.65]
Lim 2017	3.6%	0.34 [0.07, 1.64]
Miller 2005	4.7%	0.46 [0.12, 1.73]
Nestorson 2000	2.3%	0.18 [0.02, 1.37]
Rajasekar 2005	1.0%	0.23 [0.01, 5.21]
Renninger 2016	1.8%	0.56 [0.05, 5.79]
Van der Linden 2004	6.0%	0.94 [0.30, 2.92]
Wang 2015	20.0%	0.87 [0.69, 1.10]
Weber 2003	2.1%	0.24 [0.03, 1.99]
**Subtotal (95% CI)**	**69.6%**	**0.42 [0.28, 0.65]**
Heterogeneity: Tau² = 0.19; Chi² = 24.39, df = 17 (P = 0.11); I² = 30%		
Test for overall effect: Z = 3.99 (P < 0.0001)		
**Total (95% CI)**	**100.0%**	**0.43 [0.31, 0.60]**
Heterogeneity: Tau² = 0.12; Chi² = 34.22, df = 27 (P = 0.16); I² = 21%		
Test for overall effect: Z = 5.05 (P < 0.00001)		
Test for subgroup differences: Chi² = 0.01, df = 1 (P = 0.90), I² = 0%		

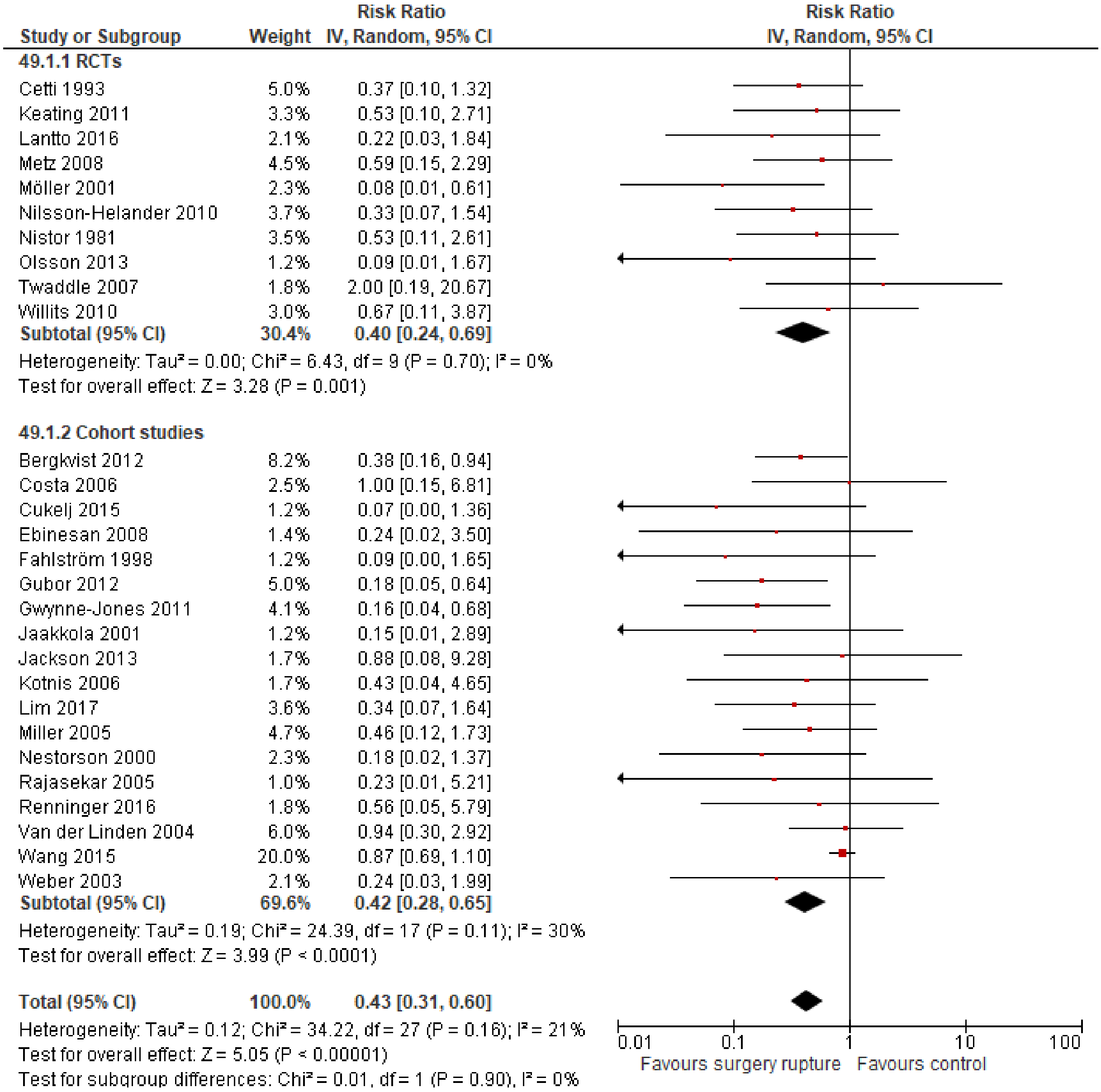

Figure S81: Ochen 2019; Intervention/ Exposure: Surgery for achilles tendon rupture; Outcome: Re-rupture

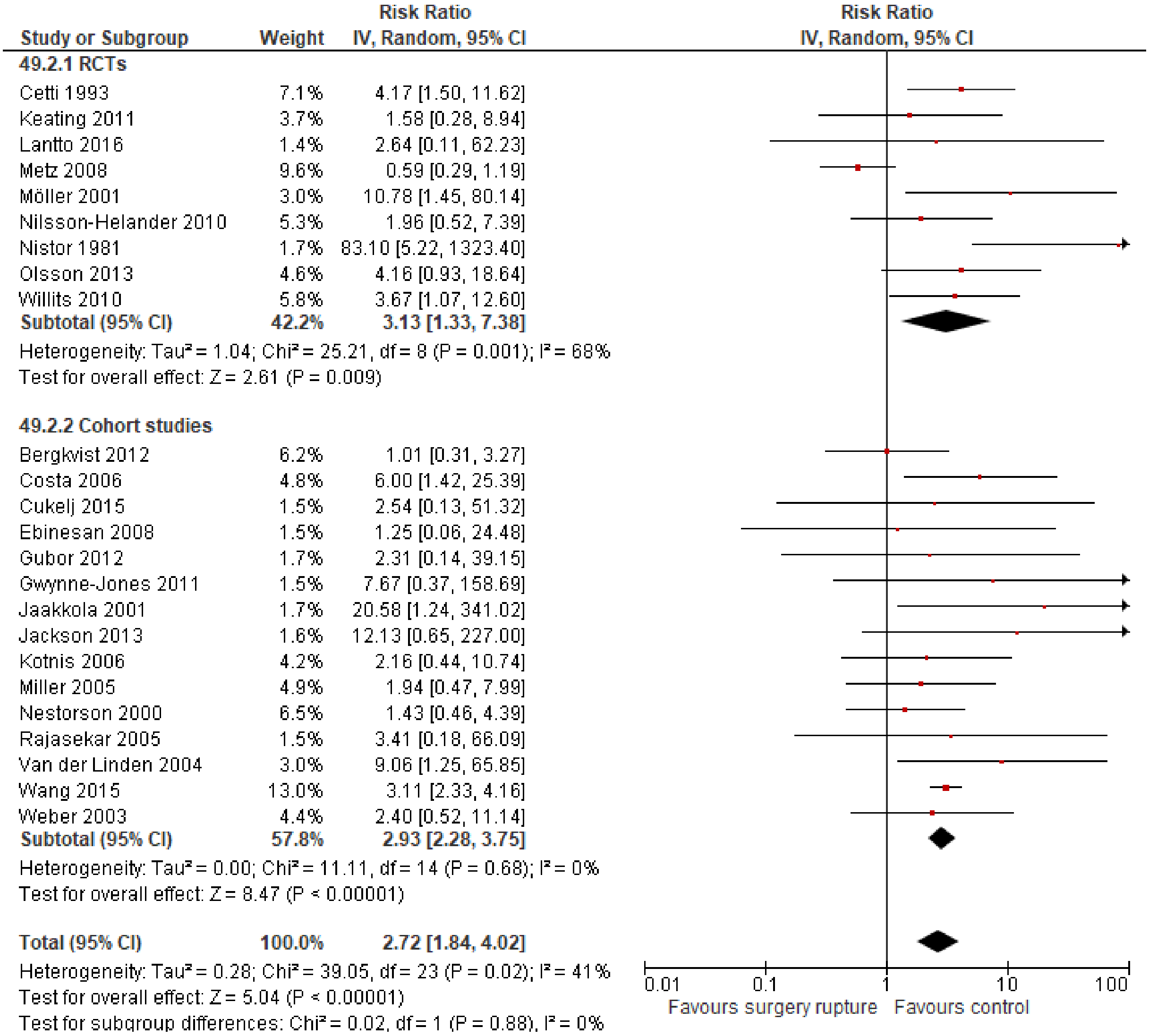

Figure S82: Ochen 2019; Intervention/ Exposure: Surgery for achilles tendon rupture; Outcome: Complications

Study or Subgroup	Weight	Risk Ratio IV, Random, 95% CI
**50.1.1 RCTs**		
Margolis 2008	38.2%	1.01 [0.96, 1.06]
**Subtotal (95% CI)**	**38.2%**	**1.01 [0.96, 1.06]**
Heterogeneity: Not applicable		
Test for overall effect: Z = 0.39 (P = 0.70)		
**50.1.2 Cohort studies**		
Forman 2007	9.9%	0.28 [0.08, 0.98]
Forman 2007	22.4%	0.59 [0.32, 1.09]
Forman 2008	29.5%	0.60 [0.40, 0.90]
**Subtotal (95% CI)**	**61.8%**	**0.57 [0.41, 0.79]**
Heterogeneity: Tau² = 0.00; Chi² = 1.29, df = 2 (P = 0.52); I² = 0%		
Test for overall effect: Z = 3.40 (P = 0.0007)		
**Total (95% CI)**	**100.0%**	**0.68 [0.43, 1.07]**
Heterogeneity: Tau² = 0.14; Chi² = 13.01, df = 3 (P = 0.005); I² = 77%		
Test for overall effect: Z = 1.68 (P = 0.09)		
Test for subgroup differences: Chi² = 11.71, df = 1 (P = 0.0006), I² = 91.5%		

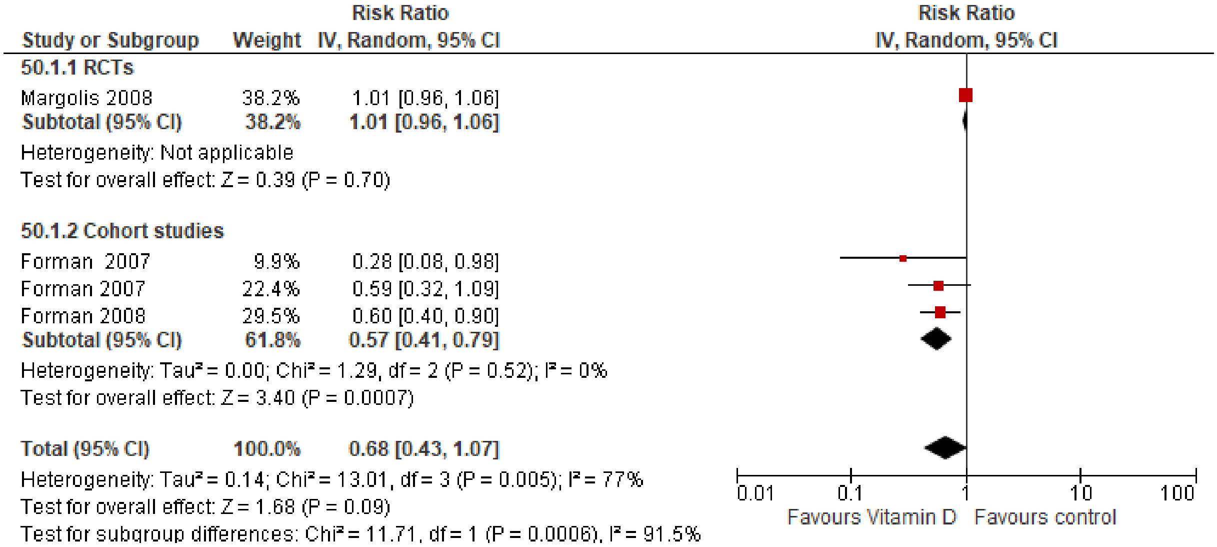

Figure S83: Pittas 2010; Intervention/ Exposure: High vitamin D; Outcome: Hypertension

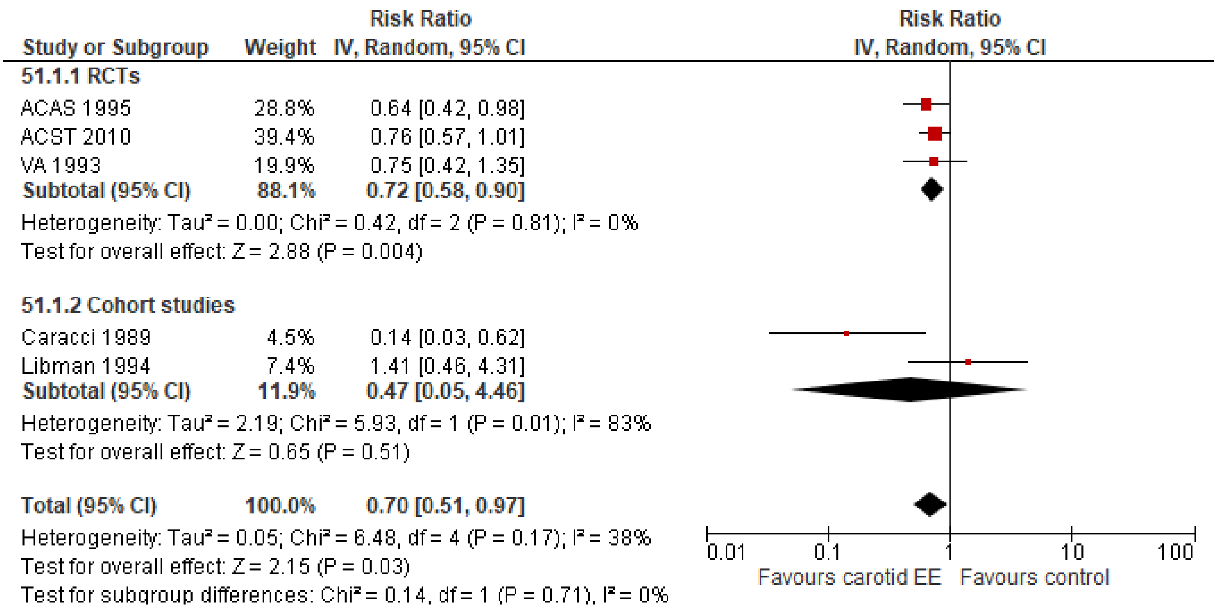

Figure S84: Raman 2013; Intervention/ Exposure: Carotid endarterectomy; Outcome: Ipsilateral stroke

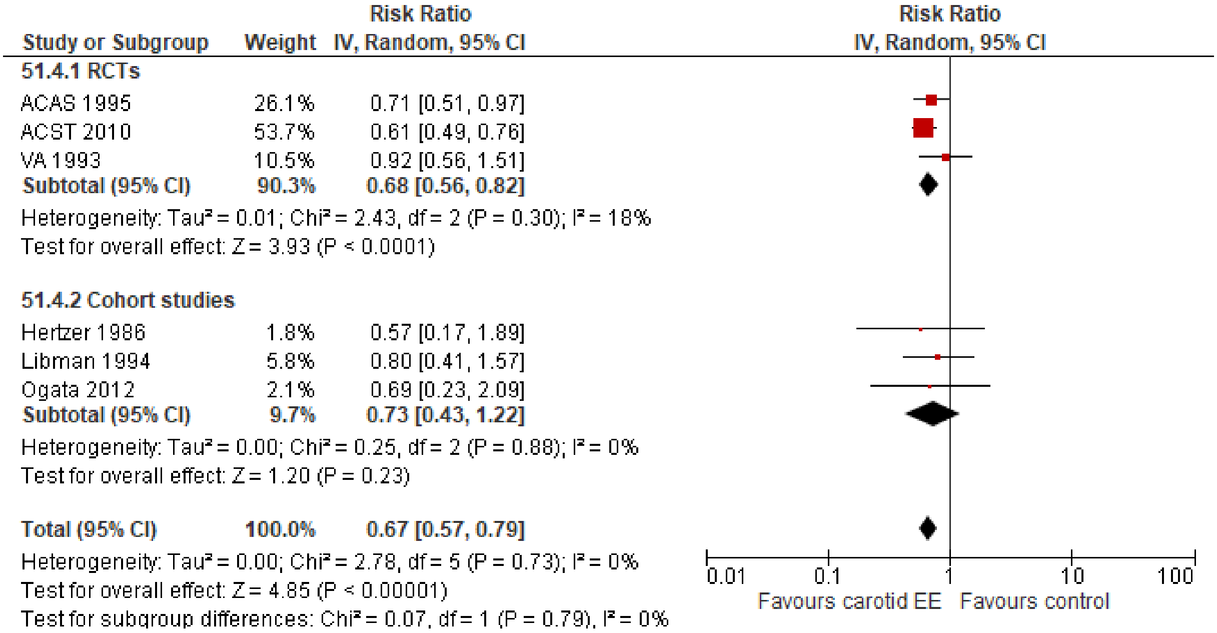

Figure S85: Raman 2013; Intervention/ Exposure: Carotid endarterectomy; Outcome: Stroke

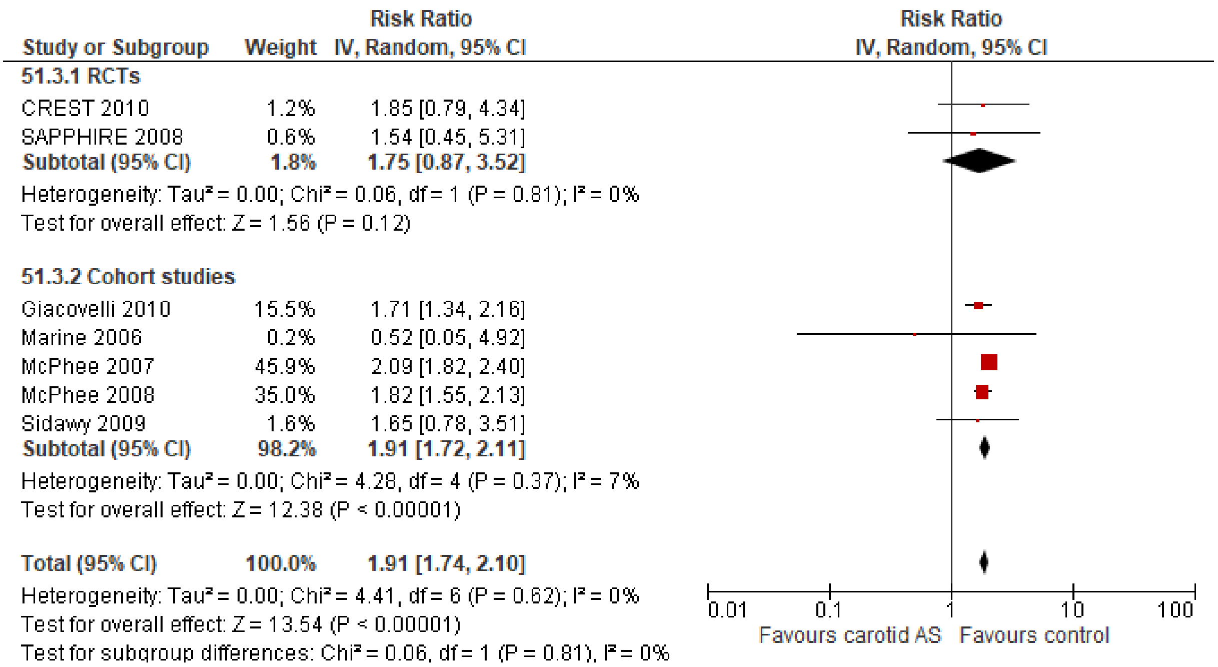

Figure S86: Raman 2013; Intervention/ Exposure: Carotid artery stenting; Outcome: Periprocedural stroke

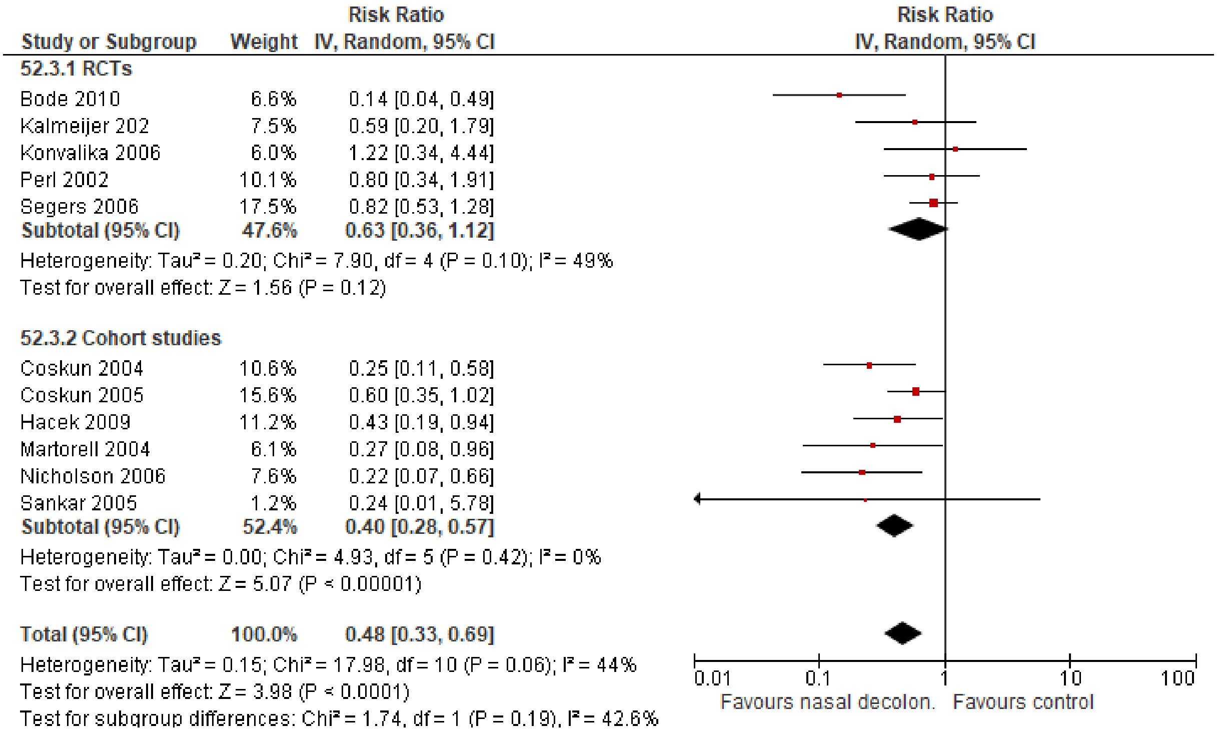

Figure S87: Schweizer 2013; Intervention/ Exposure: Nasal deconolization; Outcome: Surgical site infection

Study or Subgroup	Weight	Risk Ratio IV, Random, 95% CI
**52.4.1 RCTs**		
Dhadwal 2007	6.8%	0.46 [0.15, 1.40]
Finkelstein 2002	13.2%	1.07 [0.69, 1.66]
Niederhauser 1997	1.4%	0.30 [0.01, 7.02]
Periti 1999	6.1%	1.01 [0.30, 3.48]
Saginur 2000	14.2%	1.38 [0.99, 1.90]
Salminen 1999	4.9%	1.26 [0.29, 5.47]
Tyllianakis 2010	5.1%	0.87 [0.21, 3.59]
Vuorisalo 1998	9.7%	0.79 [0.36, 1.73]
**Subtotal (95% CI)**	**61.5%**	**1.13 [0.90, 1.42]**
Heterogeneity: Tau² = 0.00; Chi² = 5.64, df = 7 (P = 0.58); I² = 0%		
Test for overall effect: Z = 1.04 (P = 0.30)		
**52.4.2 Cohort studies**		
Bull 2010	14.1%	1.29 [0.91, 1.82]
Gupta 2006	1.7%	0.92 [0.05, 15.93]
Merrer 2006	2.5%	0.48 [0.05, 4.53]
Pear 1998	4.9%	0.62 [0.14, 2.71]
Sewick 2012	7.4%	0.38 [0.13, 1.07]
Soriano 2006	2.9%	0.07 [0.01, 0.58]
Spelman 2002	5.2%	0.05 [0.01, 0.20]
**Subtotal (95% CI)**	**38.5%**	**0.35 [0.12, 1.03]**
Heterogeneity: Tau² = 1.44; Chi² = 29.28, df = 6 (P < 0.0001); I² = 80%		
Test for overall effect: Z = 1.91 (P = 0.06)		
**Total (95% CI)**	**100.0%**	**0.71 [0.48, 1.05]**
Heterogeneity: Tau² = 0.25; Chi² = 36.39, df = 14 (P = 0.0009); I² = 62%		
Test for overall effect: Z = 1.73 (P = 0.08)		
Test for subgroup differences: Chi² = 4.35, df = 1 (P = 0.04), I² = 77.0%		

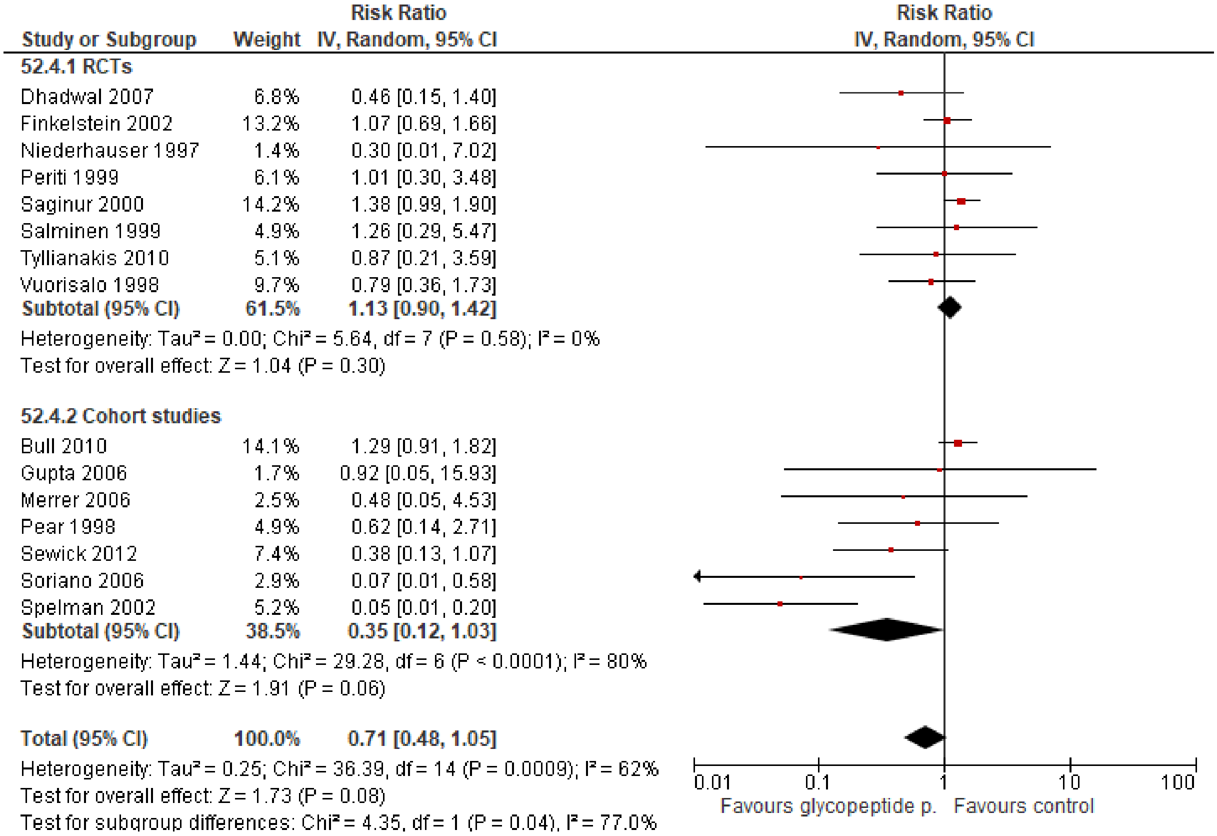

Figure S88: Schweizer 2013; Intervention/ Exposure: Glycopeptide prophylaxis; Outcome: Surgical site infection

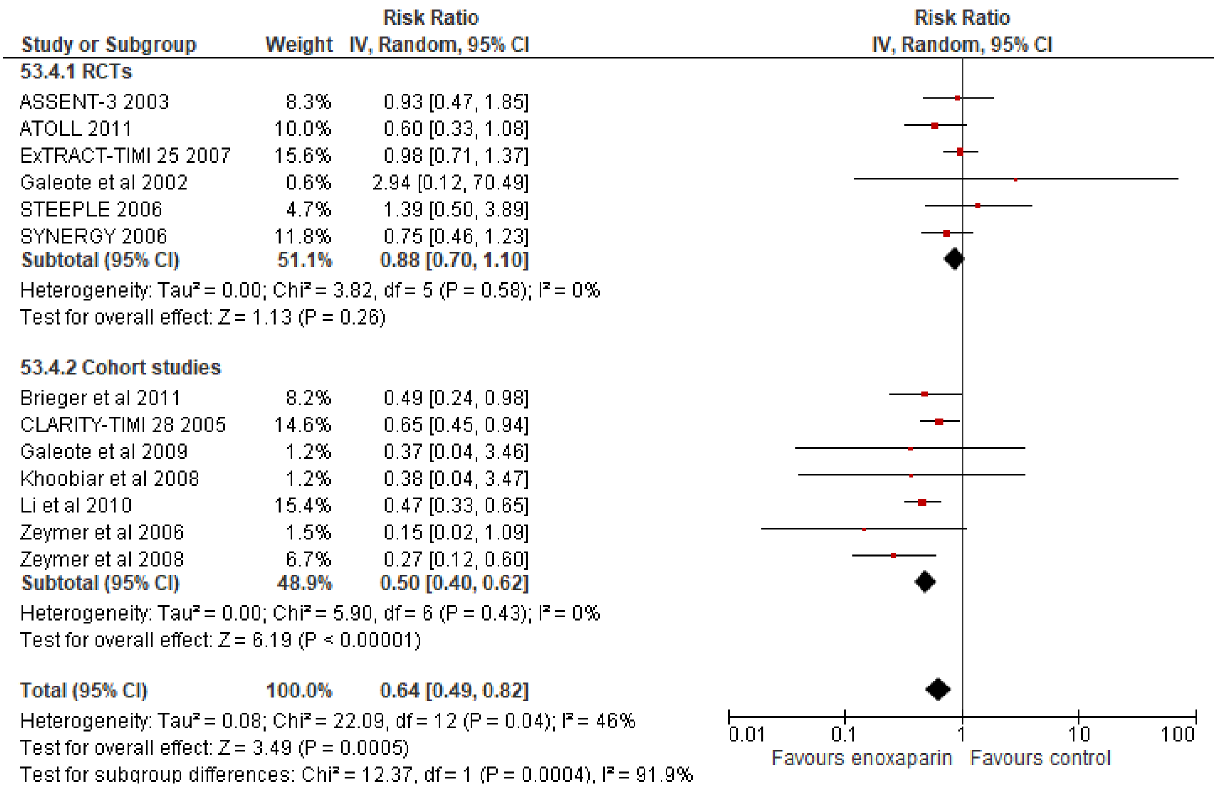

Figure S89: Silvain 2012; Intervention/ Exposure: Enoxaparin; Outcome: All-cause mortality

Study or Subgroup	Weight	Risk Ratio IV, Random, 95% CI
**53.5.1 RCTs**		
ASSENT-3 2003	7.3%	1.52 [0.81, 2.85]
ATOLL 2011	7.9%	0.93 [0.51, 1.68]
CRUISE 2003	1.3%	1.53 [0.26, 9.03]
ExTRACT-TIMI 25 2007	10.6%	0.87 [0.55, 1.38]
Galeote et al 2002	0.9%	0.24 [0.03, 2.12]
Rabah et al 1999	0.6%	1.00 [0.07, 15.26]
STEEPLE 2006	9.8%	0.44 [0.27, 0.72]
SYNERGY 2006	13.6%	1.30 [0.91, 1.85]
ZEUS 2010	5.7%	0.65 [0.31, 1.38]
**Subtotal (95% CI)**	**57.5%**	**0.88 [0.62, 1.24]**
Heterogeneity: Tau² = 0.12; Chi² = 17.12, df = 8 (P = 0.03); I² = 53%		
Test for overall effect: Z = 0.74 (P = 0.46)		
**53.5.2 Cohort studies**		
Brieger et al 2011	8.7%	0.87 [0.50, 1.50]
CLARITY-TIMI 28 2005	9.0%	0.74 [0.44, 1.27]
Diez et al 2009	1.5%	0.41 [0.08, 2.00]
Khoobiar et al 2008	0.5%	0.22 [0.01, 4.55]
Li et al 2010	4.2%	0.87 [0.35, 2.17]
Zeymer et al 2006	8.9%	0.77 [0.45, 1.32]
Zeymer et al 2008	9.7%	0.59 [0.36, 0.98]
**Subtotal (95% CI)**	**42.5%**	**0.72 [0.56, 0.93]**
Heterogeneity: Tau² = 0.00; Chi² = 2.33, df = 6 (P = 0.89); I² = 0%		
Test for overall effect: Z = 2.53 (P = 0.01)		
**Total (95% CI)**	**100.0%**	**0.81 [0.66, 1.00]**
Heterogeneity: Tau² = 0.05; Chi² = 21.57, df = 15 (P = 0.12); I² = 30%		
Test for overall effect: Z = 1.97 (P = 0.05)		
Test for subgroup differences: Chi² = 0.80, df = 1 (P = 0.37), I² = 0%		

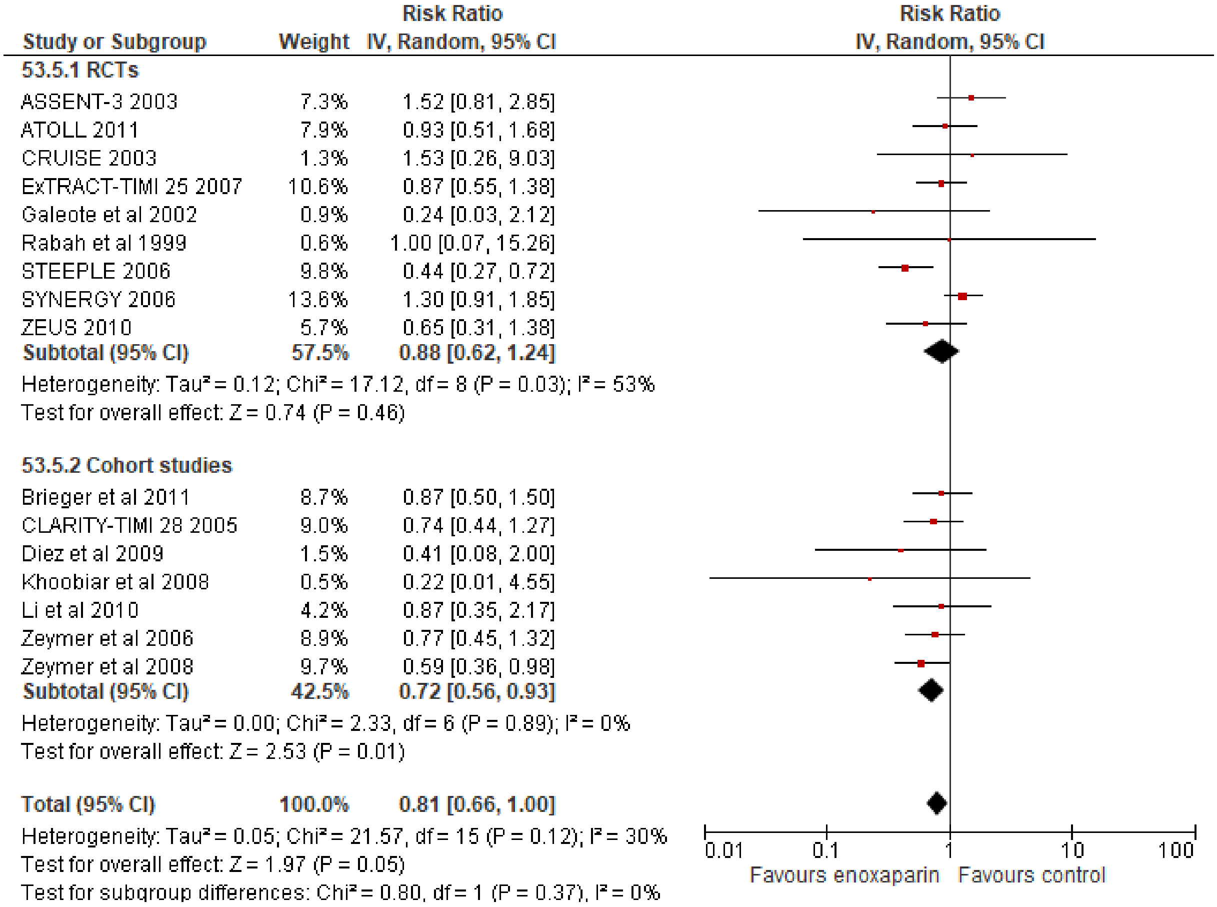

Figure S90: Silvain 2012; Intervention/ Exposure: Enoxaparin; Outcome: Major bleeding

Study or Subgroup	Weight	Risk Ratio IV, Random, 95% CI
**53.6.3 RCTs**		
ACTION 2005	4.0%	0.57 [0.25, 1.30]
ATOLL 2011	7.7%	0.96 [0.59, 1.55]
CRUISE 2003	4.1%	1.13 [0.50, 2.56]
Drozd et al 2001	0.4%	0.20 [0.01, 4.06]
Dudek et al 2000a,b	3.2%	0.39 [0.15, 1.02]
Dudek et al 2000a,b	3.5%	1.25 [0.50, 3.10]
ExTRACT-TIMI 25 2007	13.2%	0.77 [0.66, 0.90]
Galeote et al 2002	1.8%	1.63 [0.41, 6.47]
Her et al 2006	0.6%	0.52 [0.05, 5.63]
Rabah et al 1999	0.4%	0.33 [0.01, 7.87]
STEEPLE 2006	11.1%	1.06 [0.80, 1.39]
SYNERGY 2006	13.2%	0.91 [0.78, 1.06]
ZEUS 2010	2.6%	0.29 [0.10, 0.87]
**Subtotal (95% CI)**	**65.7%**	**0.86 [0.74, 0.99]**
Heterogeneity: $Tau^2 = 0.01$; $Chi^2 = 15.18$, df = 12 (P = 0.23); $I^2 = 21\%$		
Test for overall effect: Z = 2.07 (P = 0.04)		
**53.6.4 Cohort studies**		
Brieger et al 2011	5.8%	0.44 [0.24, 0.83]
Diez et al 2009	5.3%	0.50 [0.25, 0.98]
Galeote et al 2009	0.7%	0.37 [0.04, 3.46]
Khoobiar et al 2008	1.6%	0.85 [0.20, 3.55]
Li et al 2010	10.2%	0.46 [0.33, 0.63]
Zeymer et al 2006	3.9%	0.37 [0.16, 0.87]
Zeymer et al 2008	6.7%	0.36 [0.21, 0.63]
**Subtotal (95% CI)**	**34.3%**	**0.44 [0.35, 0.55]**
Heterogeneity: $Tau^2 = 0.00$; $Chi^2 = 1.62$, df = 6 (P = 0.95); $I^2 = 0\%$		
Test for overall effect: Z = 7.08 (P < 0.00001)		
**Total (95% CI)**	**100.0%**	**0.67 [0.55, 0.81]**
Heterogeneity: $Tau^2 = 0.07$; $Chi^2 = 44.84$, df = 19 (P = 0.0007); $I^2 = 58\%$		
Test for overall effect: Z = 4.04 (P < 0.0001)		
Test for subgroup differences: $Chi^2 = 23.73$, df = 1 (P < 0.00001), $I^2 = 95.8\%$		

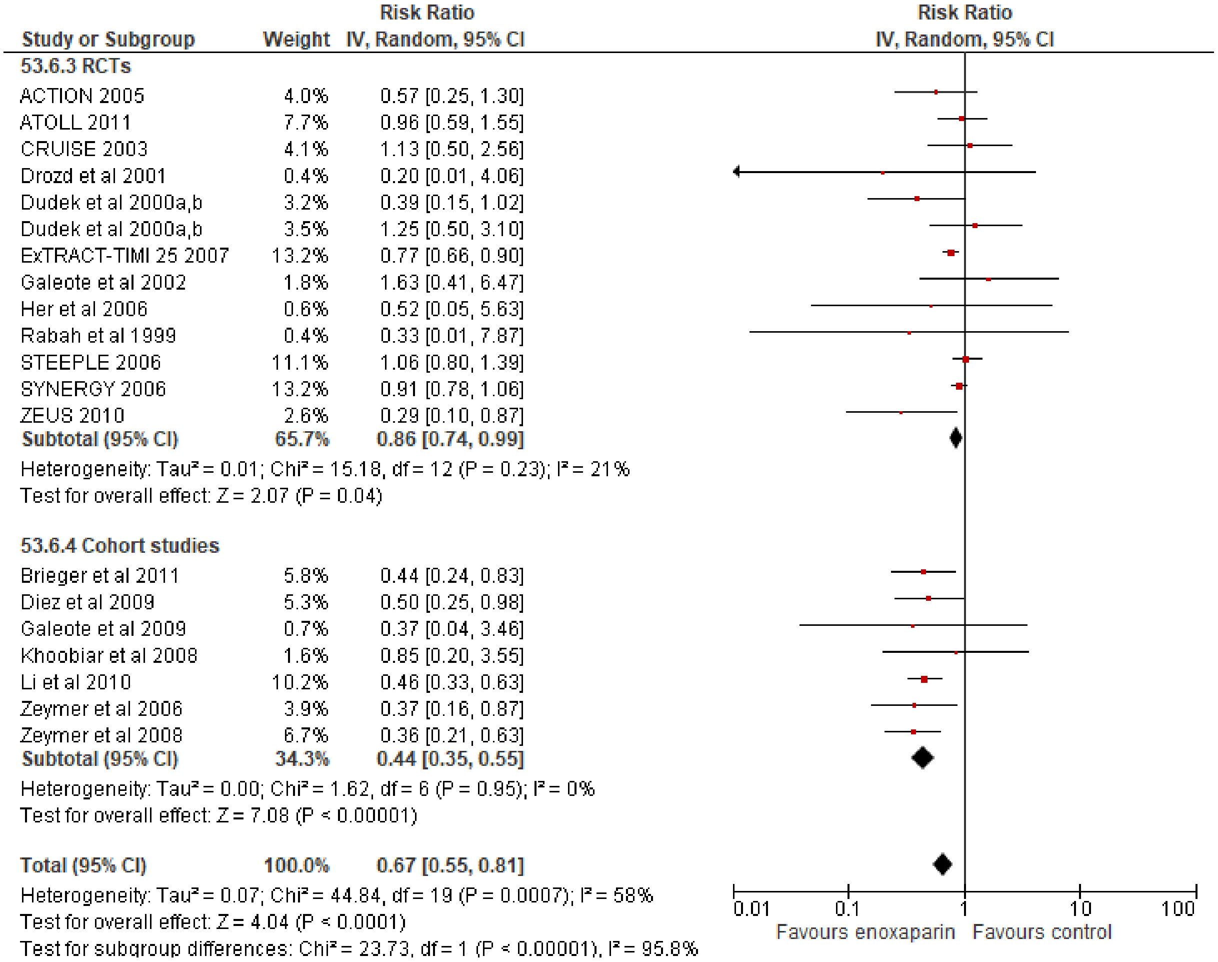

Figure S91: Silvain 2012; Intervention/ Exposure: Enoxaparin; Outcome: All-cause mortality or myocardial infarction

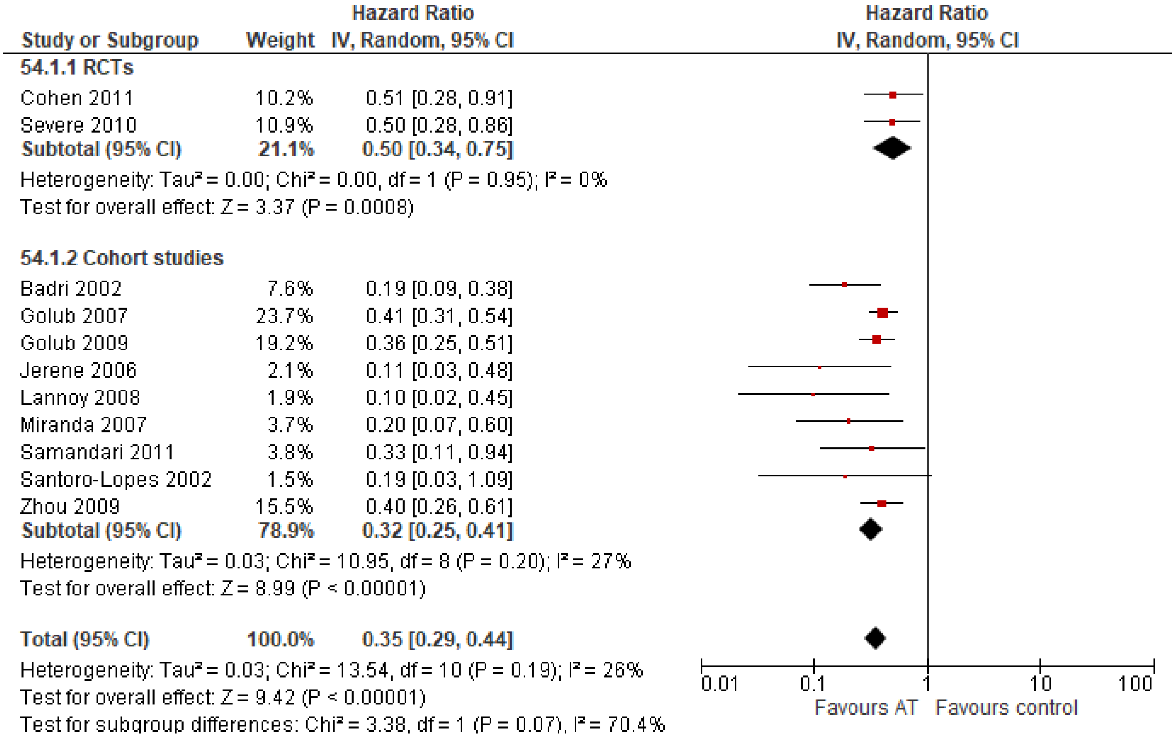

Figure S92: Suthar 2012; Intervention/ Exposure: Antiretroviral therapy; Outcome: Tuberculosis infection

Study or Subgroup	Weight	Mean Difference IV, Random, 95% CI
**55.1.1 RCTs**		
Aeberil 2011	9.8%	-0.17 [-0.42, 0.08]
Reid 2007	2.5%	0.30 [-1.07, 1.67]
Reid 2010	8.2%	0.36 [-0.07, 0.79]
Szanto 1969	8.8%	0.40 [0.03, 0.77]
Brynes 2003	6.7%	0.41 [-0.18, 1.00]
Marckmann 2000	4.8%	0.90 [0.06, 1.74]
Tordoff 1990	8.2%	0.91 [0.48, 1.34]
Werner 1984	5.2%	1.40 [0.62, 2.18]
Raben 2002	3.4%	2.60 [1.48, 3.72]
Popitt 2002	0.5%	3.97 [0.54, 7.40]
**Subtotal (95% CI)**	**58.0%**	**0.74 [0.30, 1.19]**
Heterogeneity: Tau² = 0.34; Chi² = 50.98, df = 9 (P < 0.00001); I² = 82%		
Test for overall effect: Z = 3.31 (P = 0.0009)		
**55.1.2 Cohort studies**		
Shulze 2004	9.8%	0.00 [-0.26, 0.26]
Bes-Restrollo 2010	10.9%	0.06 [0.00, 0.12]
Odegaard 2010	10.2%	0.53 [0.33, 0.73]
Palmer 2008	11.0%	0.61 [0.60, 0.62]
**Subtotal (95% CI)**	**42.0%**	**0.31 [-0.07, 0.68]**
Heterogeneity: Tau² = 0.14; Chi² = 334.81, df = 3 (P < 0.00001); I² = 99%		
Test for overall effect: Z = 1.59 (P = 0.11)		
**Total (95% CI)**	**100.0%**	**0.51 [0.26, 0.75]**
Heterogeneity: Tau² = 0.14; Chi² = 394.78, df = 13 (P < 0.00001); I² = 97%		
Test for overall effect: Z = 4.06 (P < 0.0001)		
Test for subgroup differences: Chi² = 2.21, df = 1 (P = 0.14), I² = 54.8%		

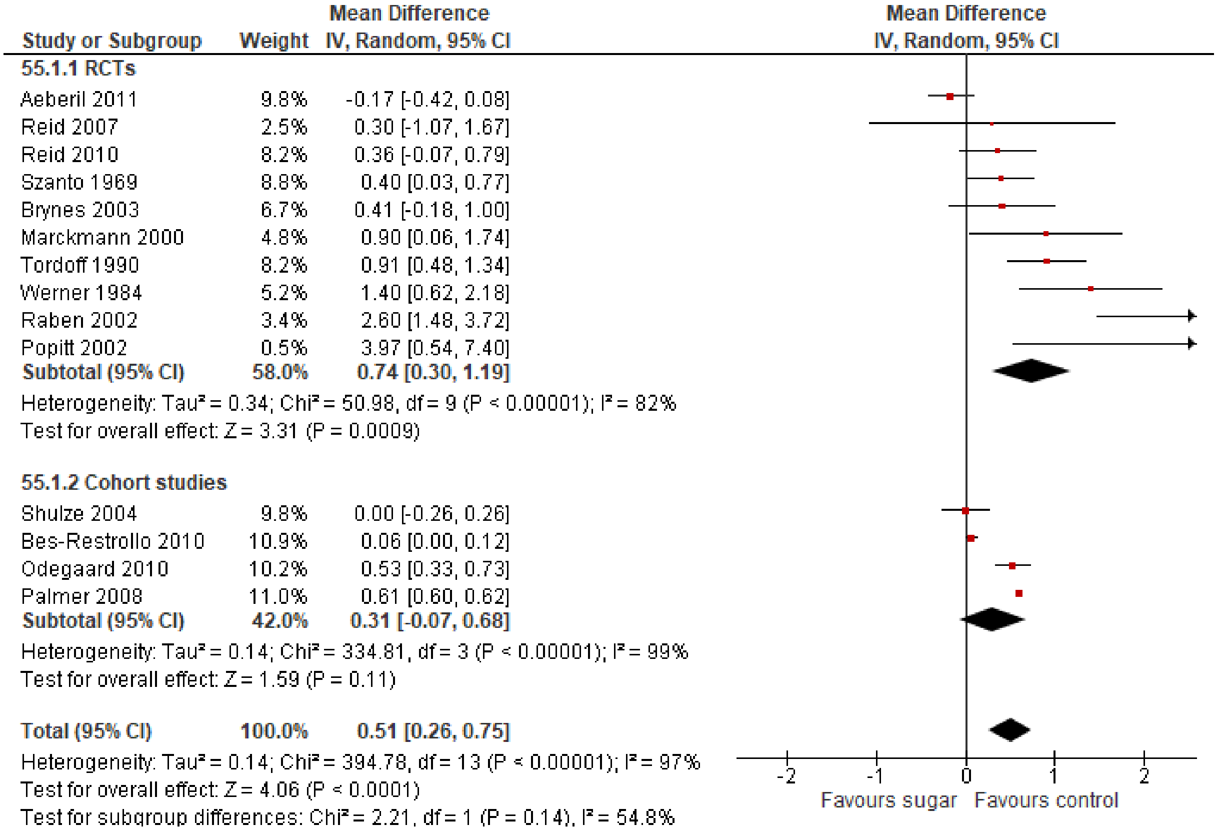

Figure S93: Te Morenga 2013; Intervention/ Exposure: High sugar intake; Outcome: Weight gain

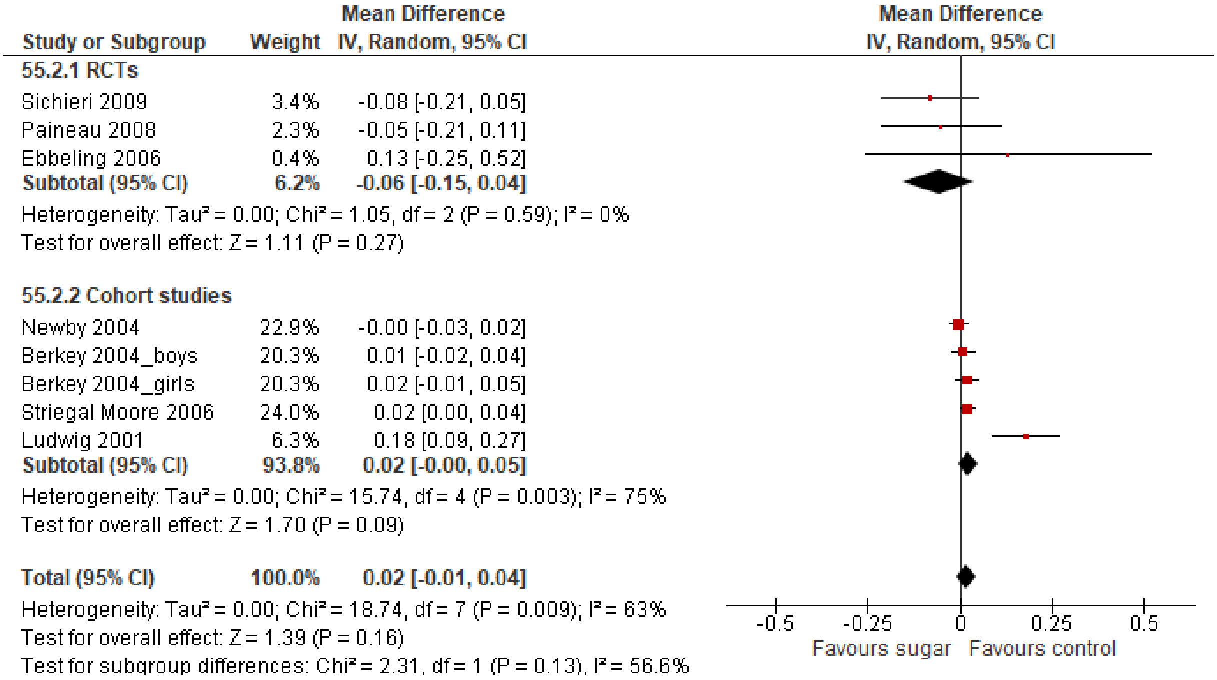

Figure S94: Te Morenga 2013; Intervention/ Exposure: High sugar intake; Outcome: Body Mass Index

Study or Subgroup	Weight	Risk Ratio IV, Random, 95% CI
**56.1.1 RCTs**		
Potter 1997	8.5%	0.14 [0.03, 0.60]
Lemaitre 2009	23.8%	0.69 [0.55, 0.87]
Hayward 2006	24.1%	0.74 [0.61, 0.90]
Potter 1997	19.1%	0.87 [0.49, 1.55]
**Subtotal (95% CI)**	**75.5%**	**0.71 [0.55, 0.90]**
Heterogeneity: Tau² = 0.02; Chi² = 5.49, df = 3 (P = 0.14); I² = 45%		
Test for overall effect: Z = 2.83 (P = 0.005)		
**56.1.2 Cohort studies**		
Oshitani 2000	24.5%	0.31 [0.26, 0.36]
**Subtotal (95% CI)**	**24.5%**	**0.31 [0.26, 0.36]**
Heterogeneity: Not applicable		
Test for overall effect: Z = 15.32 (P < 0.00001)		
**Total (95% CI)**	**100.0%**	**0.53 [0.31, 0.89]**
Heterogeneity: Tau² = 0.28; Chi² = 68.49, df = 4 (P < 0.00001); I² = 94%		
Test for overall effect: Z = 2.42 (P = 0.02)		
Test for subgroup differences: Chi² = 32.83, df = 1 (P < 0.00001), I² = 97.0%		

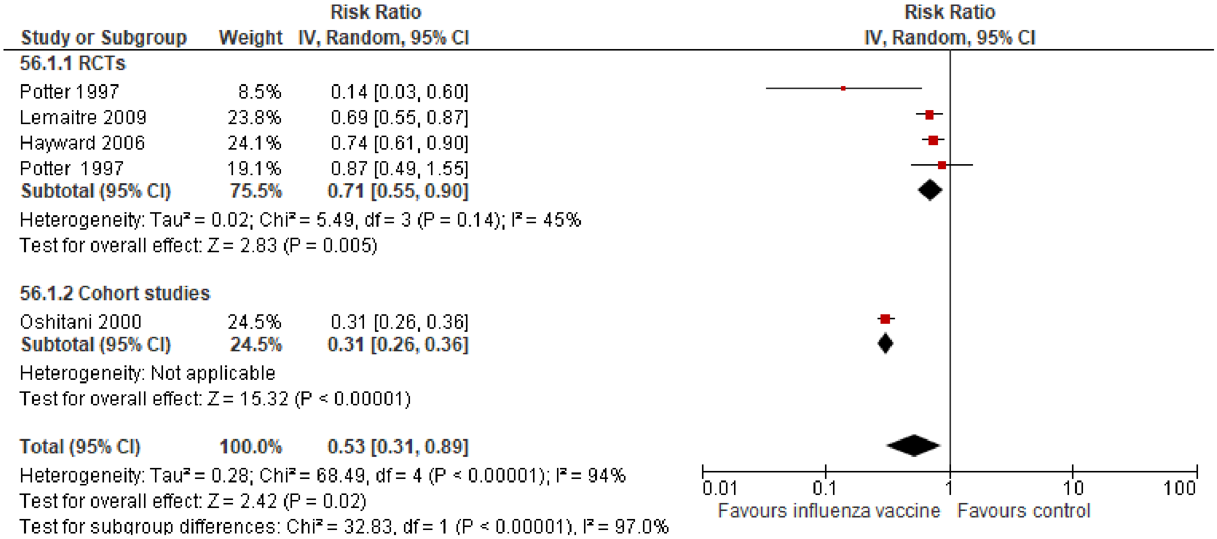

Figure S95: Thomas 2010; Intervention/ Exposure: Influenza vaccines; Outcome: Influenza-like illness

Study or Subgroup	Weight	Risk Ratio IV, Random, 95% CI
**57.3.1 RCTs**		
Overbosch 2001	9.8%	4.08 [1.68, 9.90]
Schlagenhau 2003	4.6%	2.13 [0.54, 8.36]
van Riemsdijk 1997	6.4%	1.95 [0.63, 6.04]
**Subtotal (95% CI)**	**20.8%**	**2.86 [1.53, 5.31]**
Heterogeneity: Tau² = 0.00; Chi² = 1.24, df = 2 (P = 0.54); I² = 0%		
Test for overall effect: Z = 3.31 (P = 0.0009)		
**57.3.2 Cohort studies**		
Andersson 2008	7.9%	2.48 [0.90, 6.80]
Kato 2013	5.2%	5.85 [1.64, 20.85]
Korhonen 2007	4.5%	8.26 [2.10, 32.50]
Kuhner 2005	5.8%	1.01 [0.31, 3.35]
Napoletano 2007	25.4%	3.55 [2.25, 5.59]
Sharafeldin 2010	10.7%	1.24 [0.54, 2.87]
Stoney 2016	1.2%	1.18 [0.07, 19.02]
Tan 2017	14.8%	2.81 [1.42, 5.57]
Tuck 2016	3.7%	3.63 [0.78, 16.88]
**Subtotal (95% CI)**	**79.2%**	**2.73 [1.84, 4.06]**
Heterogeneity: Tau² = 0.11; Chi² = 11.65, df = 8 (P = 0.17); I² = 31%		
Test for overall effect: Z = 4.97 (P < 0.00001)		
**Total (95% CI)**	**100.0%**	**2.78 [2.05, 3.77]**
Heterogeneity: Tau² = 0.04; Chi² = 12.89, df = 11 (P = 0.30); I² = 15%		
Test for overall effect: Z = 6.59 (P < 0.00001)		
Test for subgroup differences: Chi² = 0.01, df = 1 (P = 0.91), I² = 0%		

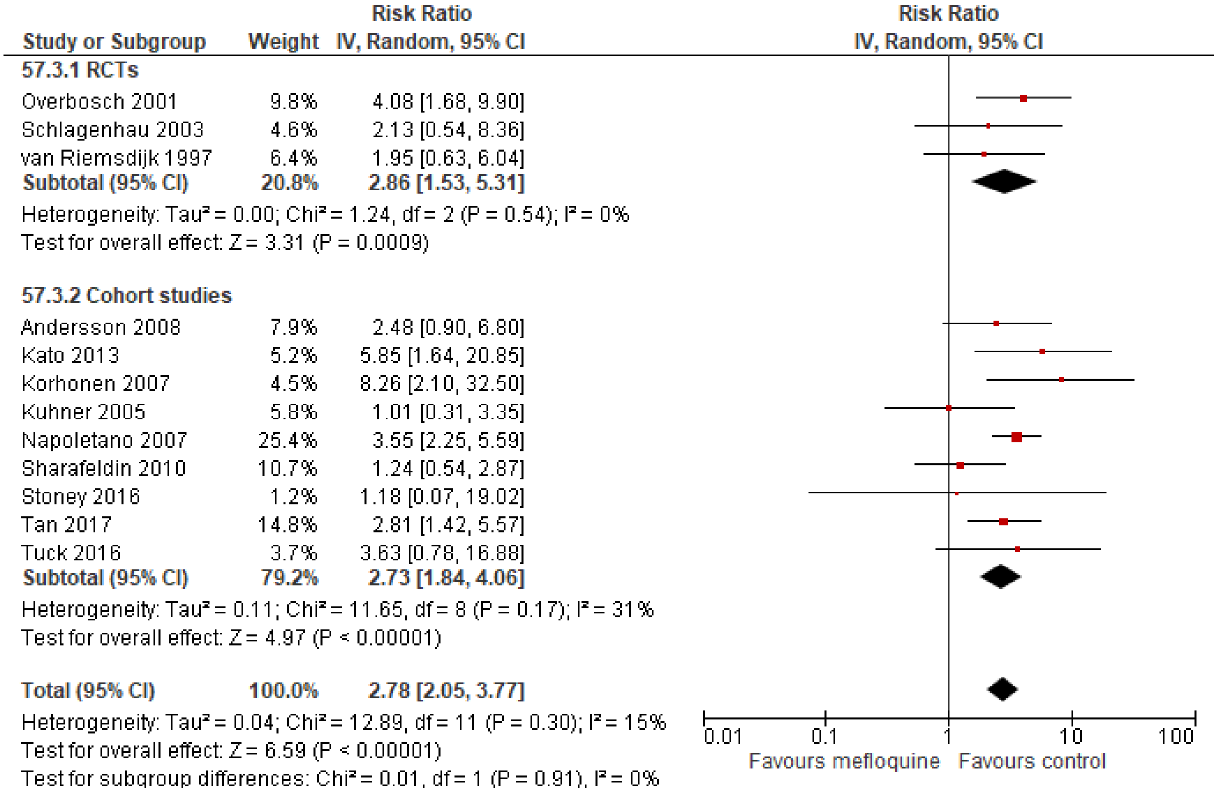

Figure S96: Tickell-Painter 2017; Intervention/ Exposure: Mefloquine; Outcome: Discontinuation due to adverse effects

Study or Subgroup	Weight	Risk Ratio IV, Random, 95% CI
**57.2.1 RCTs**		
Bunnag 1992	18.8%	0.35 [0.01, 8.45]
Nosten 1994	18.7%	2.87 [0.12, 69.89]
Sossohouto 1995	18.8%	0.31 [0.01, 7.54]
**Subtotal (95% CI)**	**56.3%**	**0.68 [0.11, 4.27]**
Heterogeneity: Tau² = 0.00; Chi² = 1.18, df = 2 (P = 0.55); I² = 0%		
Test for overall effect: Z = 0.42 (P = 0.68)		
**57.2.2 Cohort studies**		
Hoebe 1997	20.9%	4.48 [0.22, 92.06]
Petersen 2000	22.9%	2.20 [0.12, 39.59]
**Subtotal (95% CI)**	**43.7%**	**3.09 [0.38, 24.95]**
Heterogeneity: Tau² = 0.00; Chi² = 0.11, df = 1 (P = 0.74); I² = 0%		
Test for overall effect: Z = 1.06 (P = 0.29)		
**Total (95% CI)**	**100.0%**	**1.31 [0.33, 5.23]**
Heterogeneity: Tau² = 0.00; Chi² = 2.44, df = 4 (P = 0.66); I² = 0%		
Test for overall effect: Z = 0.39 (P = 0.70)		
Test for subgroup differences: Chi² = 1.14, df = 1 (P = 0.29), I² = 12.5%		

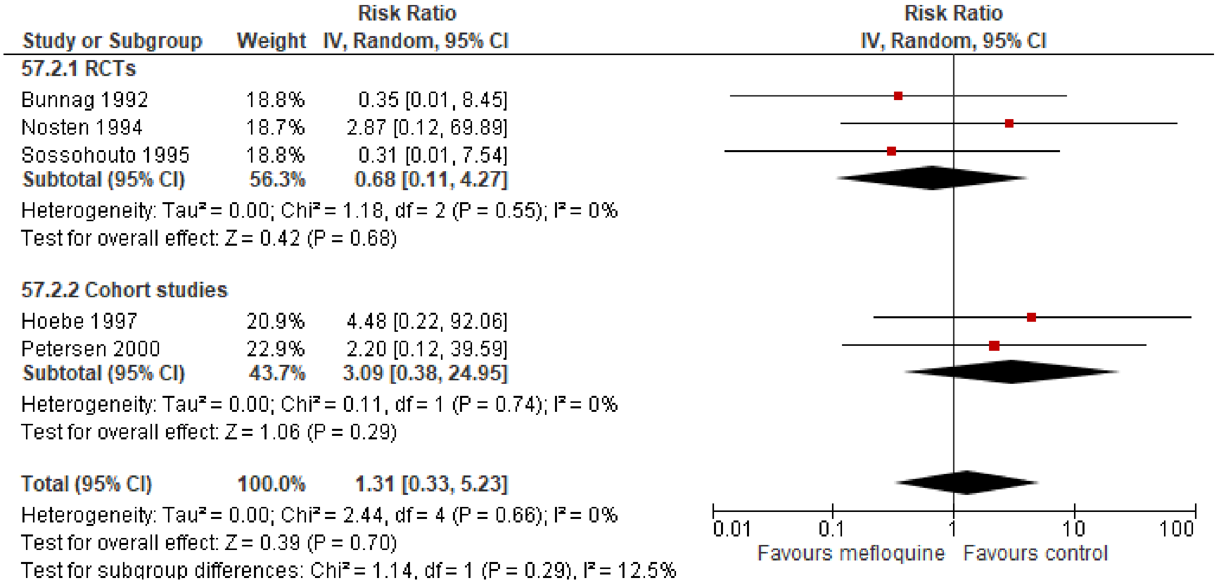

Figure S97: Tickell-Painter 2017; Intervention/ Exposure: Mefloquine; Outcome: Serious adverse events or effects

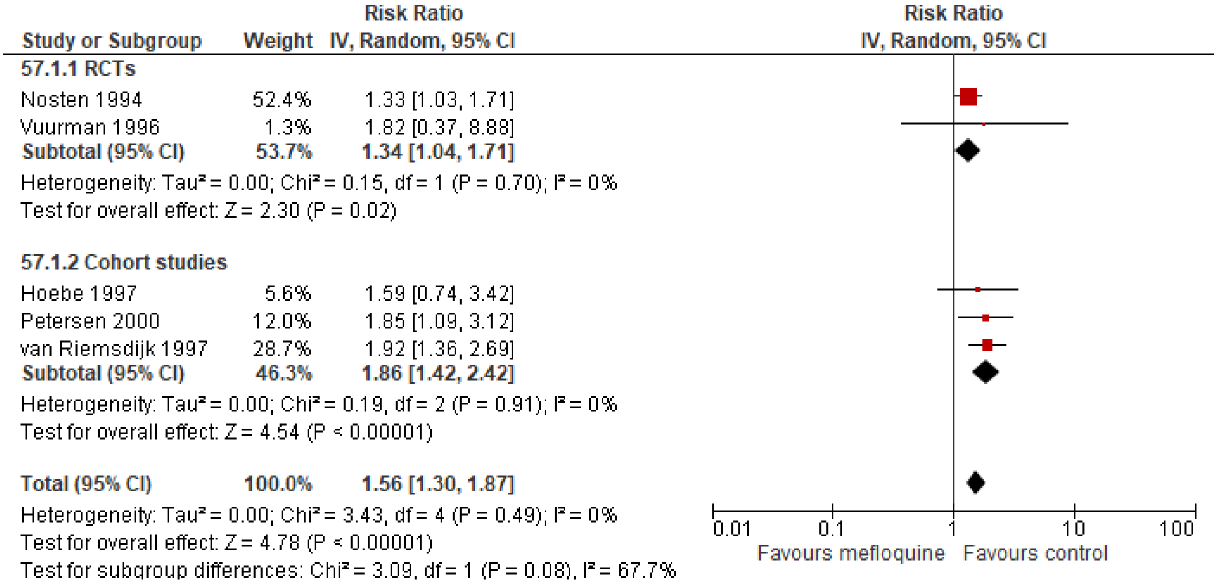

Figure S98: Tickell-Painter 2017; Intervention/ Exposure: Mefloquine; Outcome: Nausea

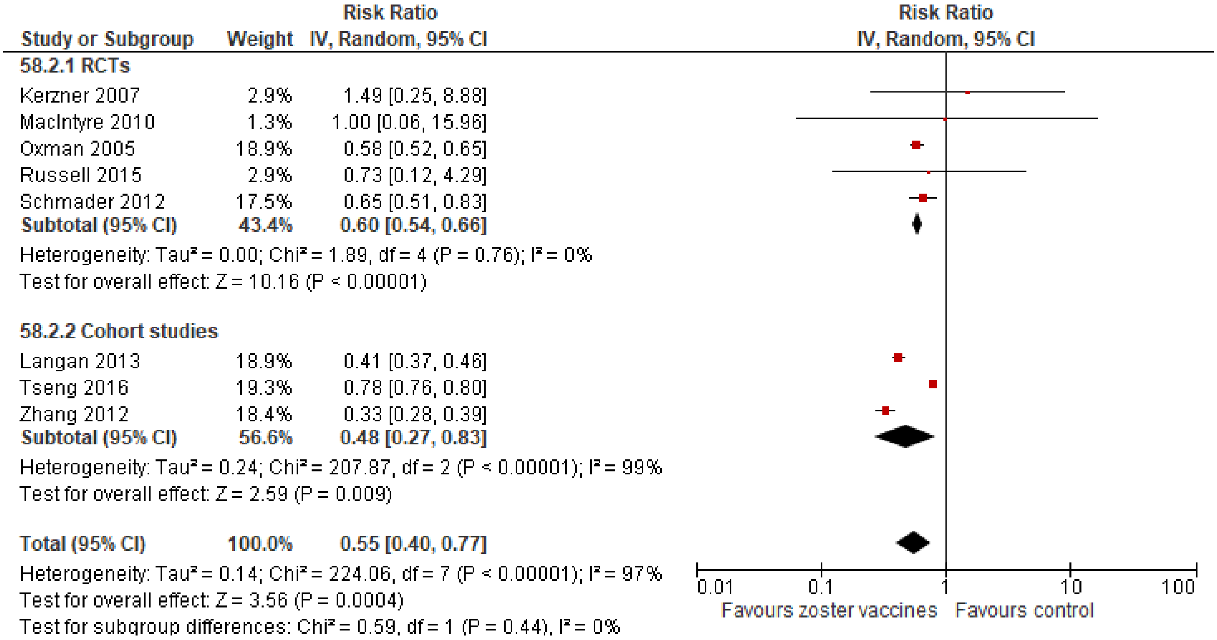

Figure S99: Tricco 2018; Intervention/ Exposure: Live-attenuated zoster vaccines; Outcome: Suspected Herpes Zoster

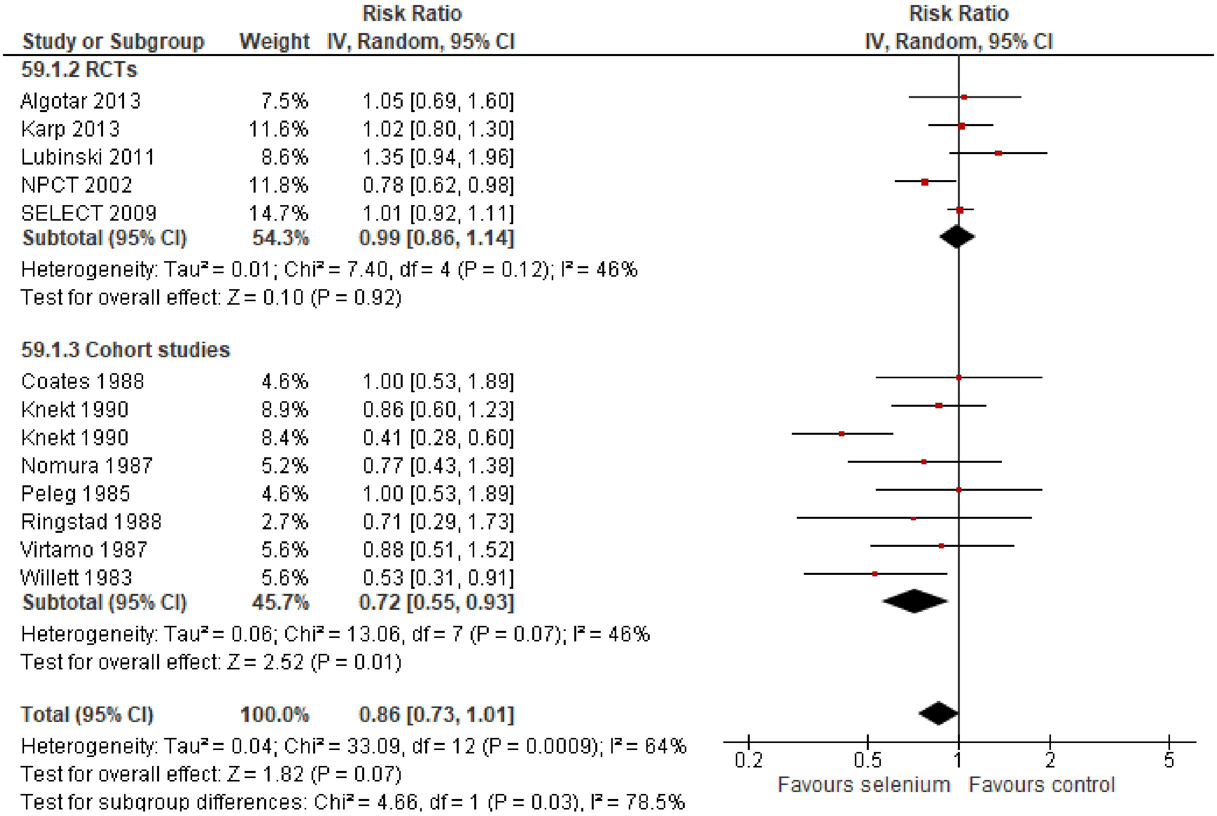

Figure S100: Vinceti 2018; Intervention/ Exposure: High selenium; Outcome: Cancer

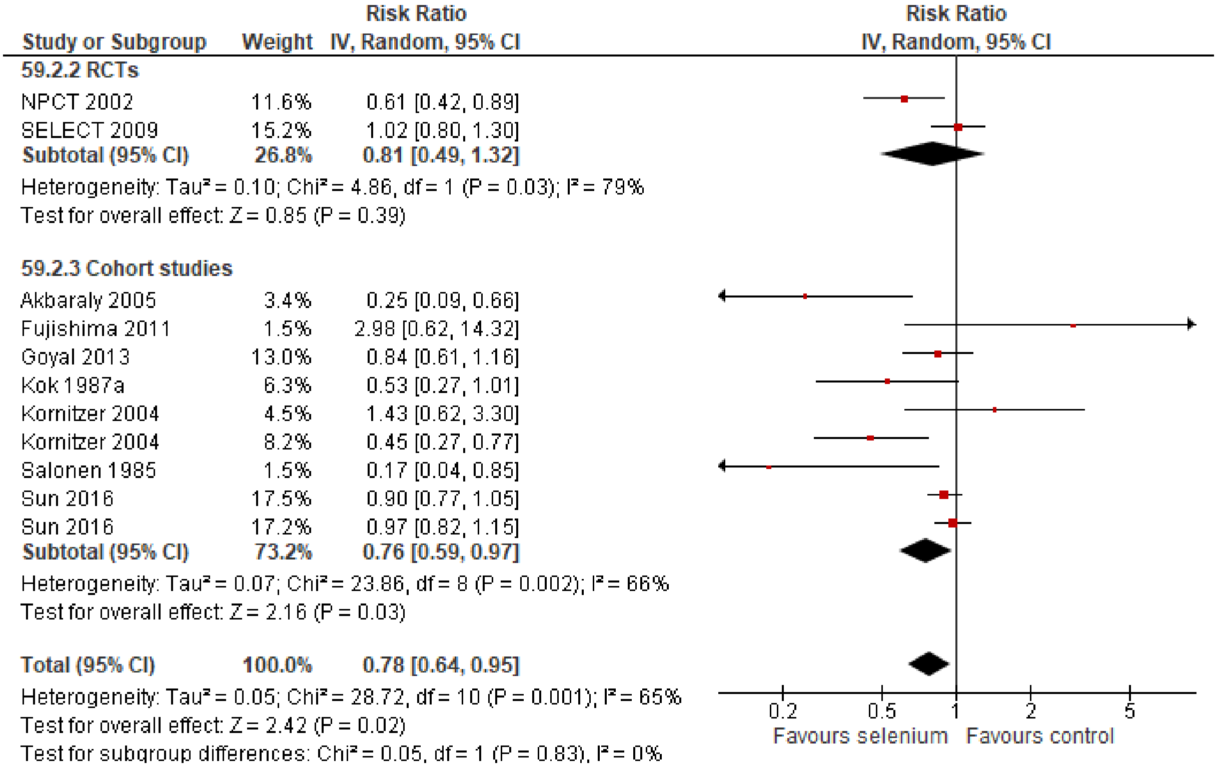

Figure S101: Vinceti 2018; Intervention/ Exposure: High selenium; Outcome: Cancer mortality

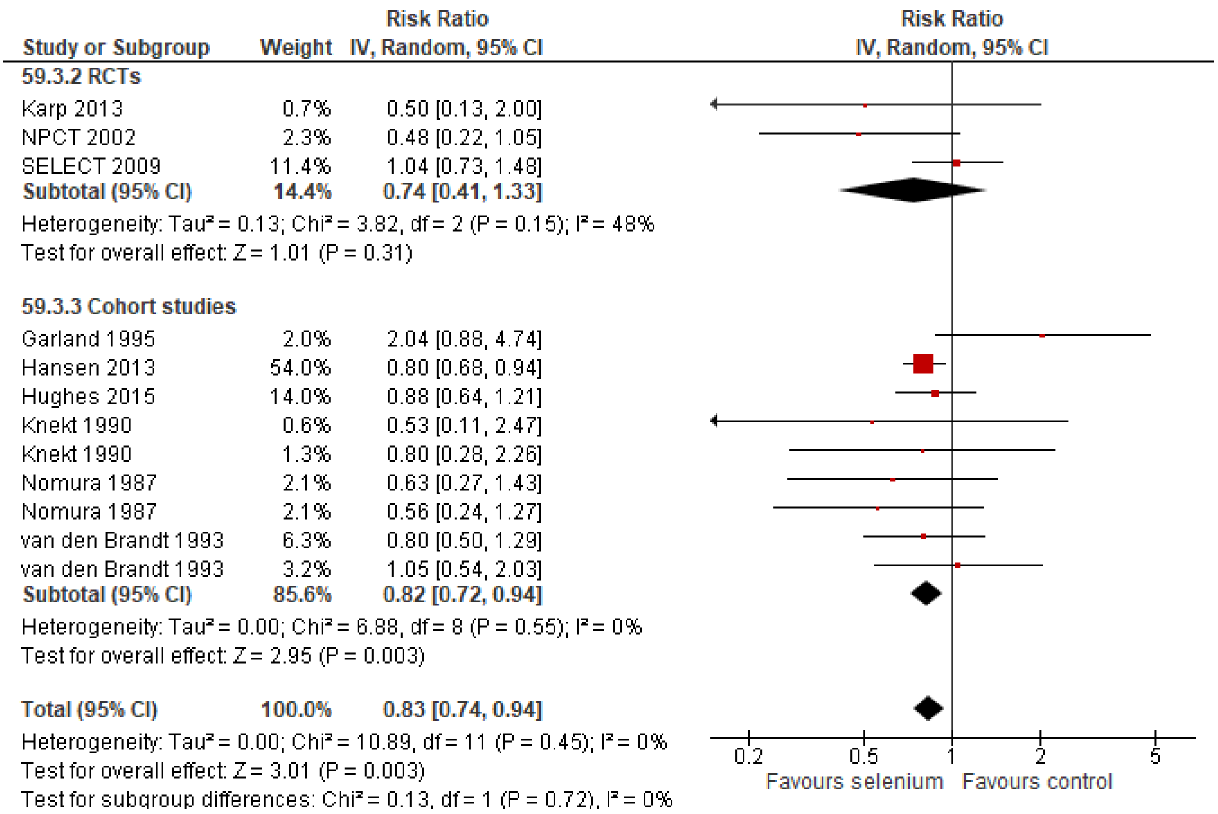

Figure S102: Vinceti 2018; Intervention/ Exposure: High selenium; Outcome: Colorectal cancer

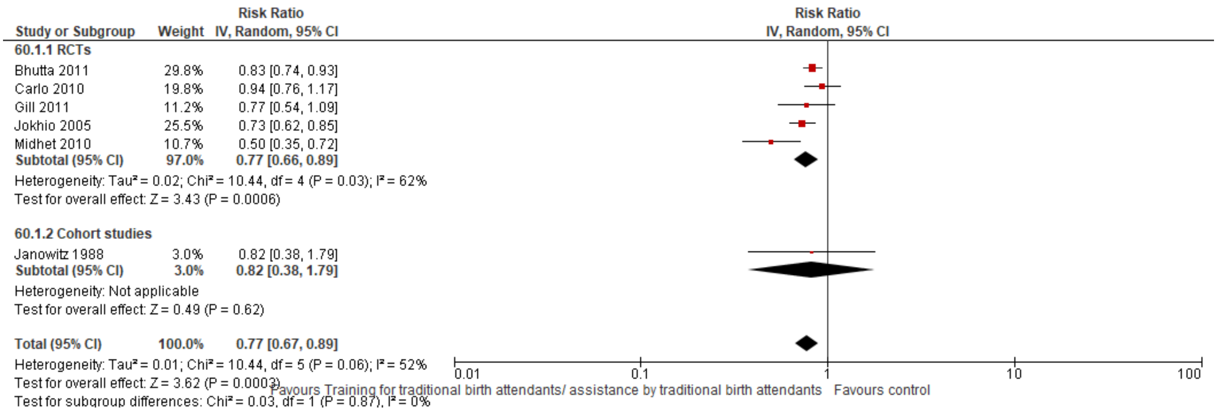

Figure S103: Wilson 2011; Intervention/ Exposure: Training for traditional birth attendants/ assistance by traditional birth attendants; Outcome: Perinatal mortality

Study or Subgroup	Weight	Risk Ratio IV, Random, 95% CI
**60.2.1 RCTs**		
Azad	12.9%	0.95 [0.75, 1.21]
Bhutta 2011	37.0%	0.85 [0.76, 0.96]
Carlo 2010	9.3%	0.87 [0.65, 1.16]
Gill 2011	3.4%	0.55 [0.33, 0.91]
Jokhio 2005	29.8%	0.72 [0.63, 0.83]
Midhet 2010	4.5%	0.70 [0.45, 1.08]
**Subtotal (95% CI)**	**97.0%**	**0.80 [0.71, 0.90]**
Heterogeneity: Tau² = 0.01; Chi² = 7.99, df = 5 (P = 0.16); I² = 37%		
Test for overall effect: Z = 3.78 (P = 0.0002)		
**60.2.2 Cohort studies**		
Gloyd 2001	2.2%	0.86 [0.46, 1.61]
Janowitz 1988	0.8%	0.65 [0.23, 1.88]
**Subtotal (95% CI)**	**3.0%**	**0.80 [0.47, 1.37]**
Heterogeneity: Tau² = 0.00; Chi² = 0.19, df = 1 (P = 0.66); I² = 0%		
Test for overall effect: Z = 0.80 (P = 0.42)		
**Total (95% CI)**	**100.0%**	**0.80 [0.73, 0.88]**
Heterogeneity: Tau² = 0.00; Chi² = 8.18, df = 7 (P = 0.32); I² = 14%		
Test for overall effect: Z = 4.59 (P < 0.00001)		
Test for subgroup differences: Chi² = 0.00, df = 1 (P = 0.99), I² = 0%		

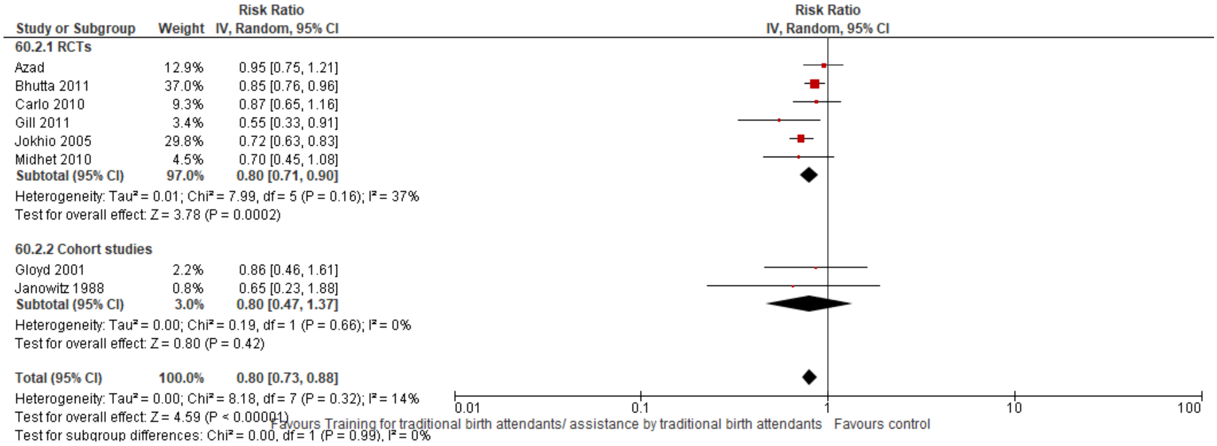

Figure S104: Wilson 2011; Intervention/ Exposure: Training for traditional birth attendants/ assistance by traditional birth attendants; Outcome: Neonatal mortality

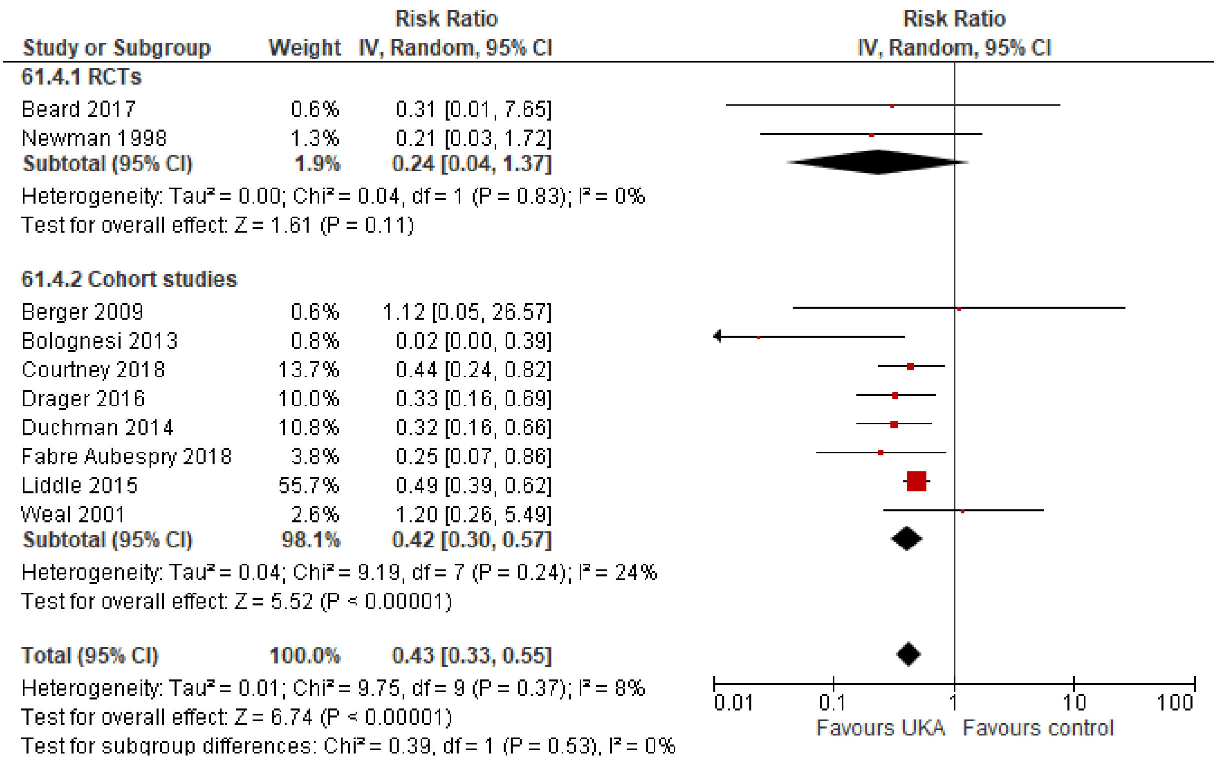

Figure S105: Wilson 2019; Intervention/ Exposure: Unicompartimental knee arthroplasty; Outcome: Venous thromboembolism

Study or Subgroup	Weight	Mean Difference IV, Random, 95% CI
**61.5.1 RCTs**		
Newman 1998	8.9%	-6.70 [-7.73, -5.67]
Sun 2011	7.4%	2.00 [-0.67, 4.67]
Weal 2001	5.9%	-9.30 [-13.43, -5.17]
**Subtotal (95% CI)**	**22.2%**	**-4.58 [-10.75, 1.59]**
Heterogeneity: Tau² = 27.67; Chi² = 38.37, df = 2 (P < 0.00001); I² = 95%		
Test for overall effect: Z = 1.45 (P = 0.15)		
**61.5.2 Cohort studies**		
Ackroyd 2002	8.1%	-10.00 [-12.05, -7.95]
Amin 2006	8.4%	-4.00 [-5.71, -2.29]
Artz 2015	6.6%	-11.20 [-14.66, -7.74]
Artz 2015 2	6.9%	-14.90 [-18.07, -11.73]
Fisher 2010	4.1%	-9.00 [-15.06, -2.94]
Horikawa 2015		Not estimable
Howell 2015	9.1%	-9.00 [-9.41, -8.59]
Lombardi 2009 1	7.8%	-5.00 [-7.28, -2.72]
Lombardi 2009 2	7.6%	-5.00 [-7.52, -2.48]
Lum 2016	8.0%	-7.10 [-9.28, -4.92]
Lum 2018		Not estimable
Mathews 2013	3.7%	-15.30 [-21.97, -8.63]
Siman 2017	7.4%	-8.00 [-10.67, -5.33]
**Subtotal (95% CI)**	**77.8%**	**-8.43 [-10.15, -6.71]**
Heterogeneity: Tau² = 6.20; Chi² = 73.85, df = 10 (P < 0.00001); I² = 86%		
Test for overall effect: Z = 9.61 (P < 0.00001)		
**Total (95% CI)**	**100.0%**	**-7.60 [-9.27, -5.93]**
Heterogeneity: Tau² = 7.85; Chi² = 144.23, df = 13 (P < 0.00001); I² = 91%		
Test for overall effect: Z = 8.94 (P < 0.00001)		
Test for subgroup differences: Chi² = 1.39, df = 1 (P = 0.24), I² = 27.9%		

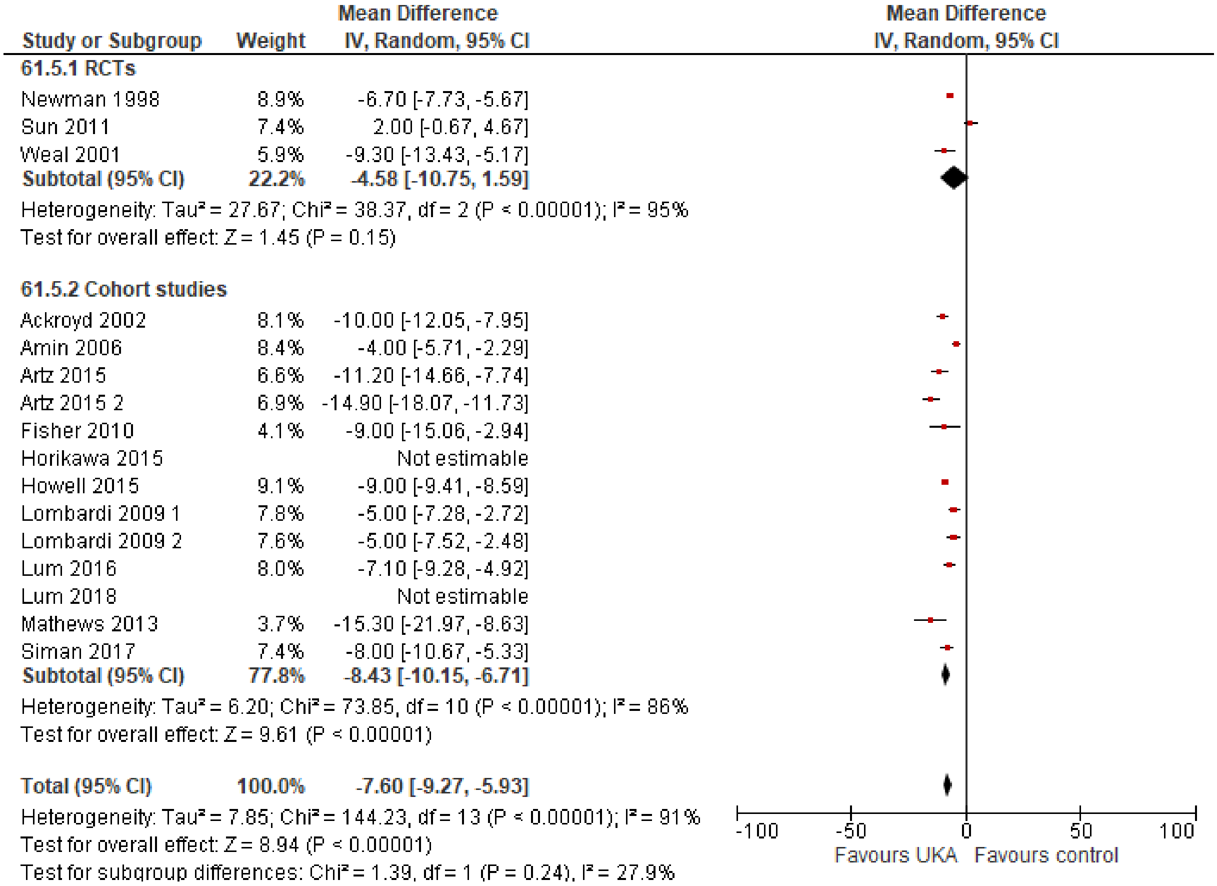

Figure S106: Wilson 2019; Intervention/ Exposure: Unicompartimental knee arthroplasty; Outcome: Flexion-extension range

Study or Subgroup	Weight	Mean Difference IV, Random, 95% CI
**61.6.1 RCTs**		
Beard 2017	10.2%	-1.00 [-4.43, 2.43]
Kulshrestha 2017	9.9%	9.00 [1.49, 16.51]
Sun 2011	10.0%	-12.70 [-19.01, -6.39]
**Subtotal (95% CI)**	**30.2%**	**-1.72 [-11.89, 8.45]**
Heterogeneity: Tau² = 71.69; Chi² = 19.61, df = 2 (P < 0.0001); I² = 90%		
Test for overall effect: Z = 0.33 (P = 0.74)		
**61.6.3 Cohort studies**		
Courtney 2018	10.1%	-6.20 [-12.33, -0.07]
Duchman 2014	10.3%	-2.36 [-4.81, 0.09]
Lombardi 2009	10.1%	-5.00 [-10.06, 0.06]
Lum 2018		Not estimable
Manzotti 2007	10.1%	-56.50 [-62.05, -50.95]
Schwab 2015	10.1%	-21.00 [-25.99, -16.01]
Shankar 2016	9.0%	-30.80 [-45.01, -16.59]
Siman 2017	10.1%	-45.60 [-51.21, -39.99]
**Subtotal (95% CI)**	**69.8%**	**-23.80 [-40.43, -7.17]**
Heterogeneity: Tau² = 491.19; Chi² = 464.14, df = 6 (P < 0.00001); I² = 99%		
Test for overall effect: Z = 2.80 (P = 0.005)		
**Total (95% CI)**	**100.0%**	**-17.07 [-29.11, -5.04]**
Heterogeneity: Tau² = 365.45; Chi² = 542.59, df = 9 (P < 0.00001); I² = 98%		
Test for overall effect: Z = 2.78 (P = 0.005)		
Test for subgroup differences: Chi² = 4.93, df = 1 (P = 0.03), I² = 79.7%		

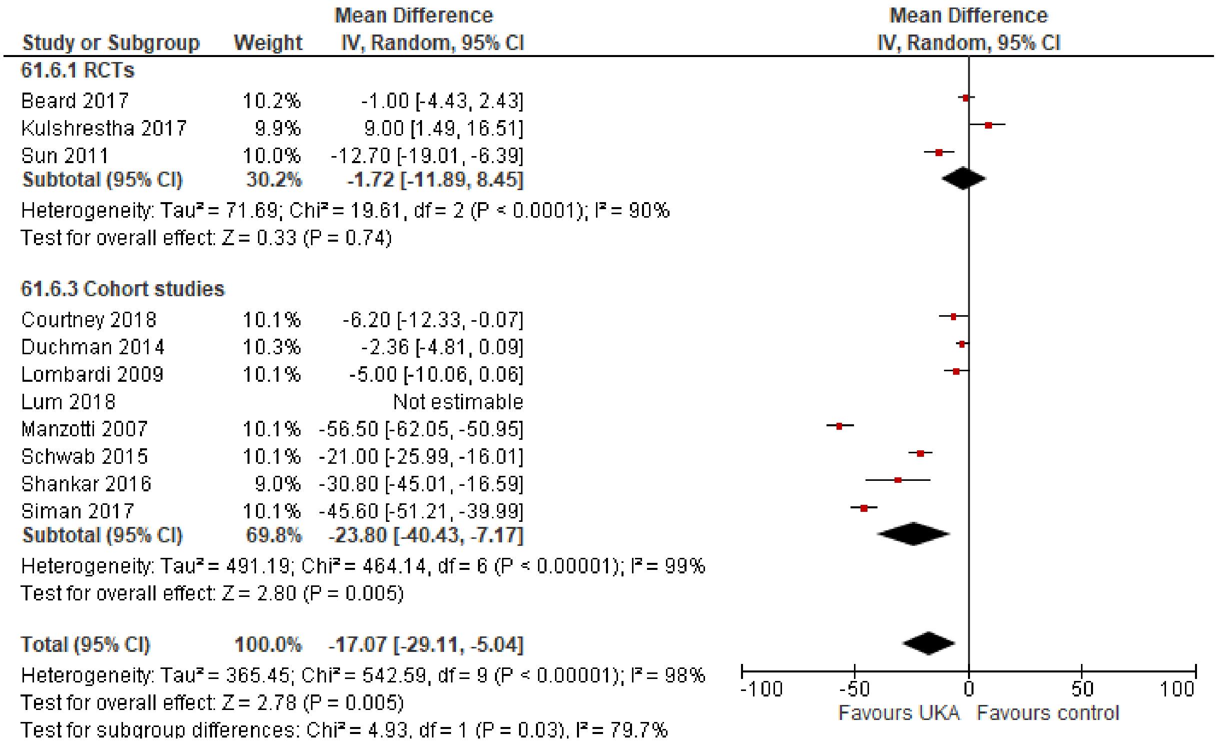

Figure S107: Wilson 2019; Intervention/ Exposure: Unicompartimental knee arthroplasty; Outcome: Operation duration

Study or Subgroup	Weight	Risk Ratio IV, Random, 95% CI
**62.5.1 RCTs**		
Diprose 2005	4.6%	0.37 [0.02, 8.01]
Gill 2009	34.9%	1.67 [0.55, 5.13]
**Subtotal (95% CI)**	**39.4%**	**1.40 [0.49, 4.02]**
Heterogeneity: Tau² = 0.00; Chi² = 0.82, df = 1 (P = 0.36); I² = 0%		
Test for overall effect: Z = 0.63 (P = 0.53)		
**62.5.2 Cohort studies**		
Gelsomino 2008	14.5%	0.67 [0.12, 3.78]
Karkouti 2005	46.1%	1.00 [0.38, 2.65]
**Subtotal (95% CI)**	**60.6%**	**0.91 [0.39, 2.12]**
Heterogeneity: Tau² = 0.00; Chi² = 0.16, df = 1 (P = 0.69); I² = 0%		
Test for overall effect: Z = 0.22 (P = 0.82)		
**Total (95% CI)**	**100.0%**	**1.08 [0.56, 2.09]**
Heterogeneity: Tau² = 0.00; Chi² = 1.38, df = 3 (P = 0.71); I² = 0%		
Test for overall effect: Z = 0.22 (P = 0.82)		
Test for subgroup differences: Chi² = 0.40, df = 1 (P = 0.53), I² = 0%		

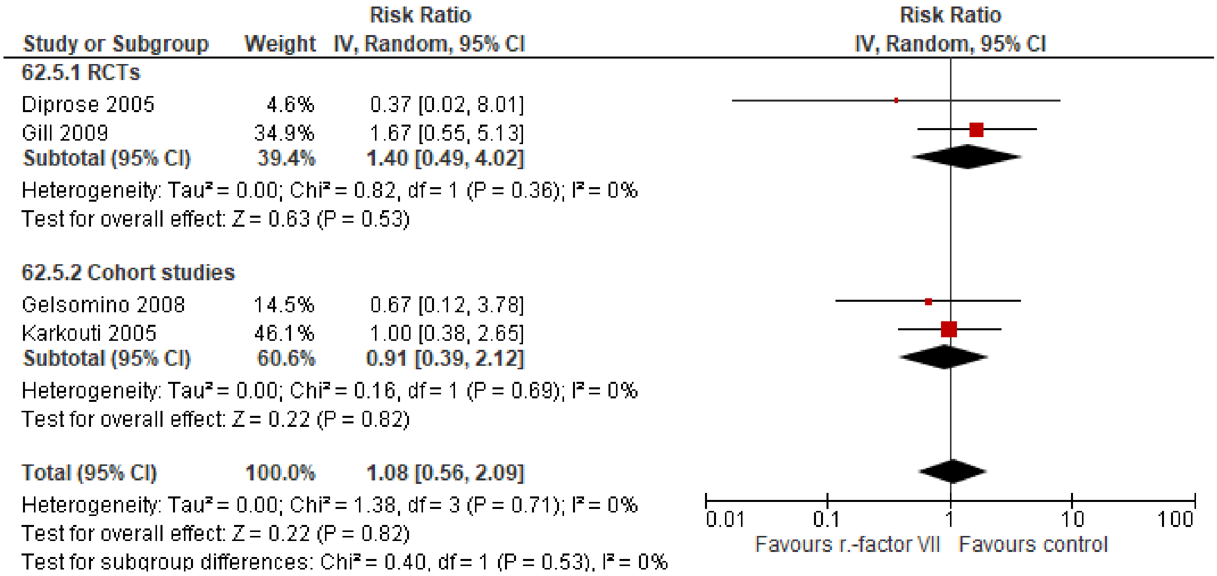

Figure S108: Yank 2011; Intervention/ Exposure: Recombinant factor VII; Outcome: All-cause mortality

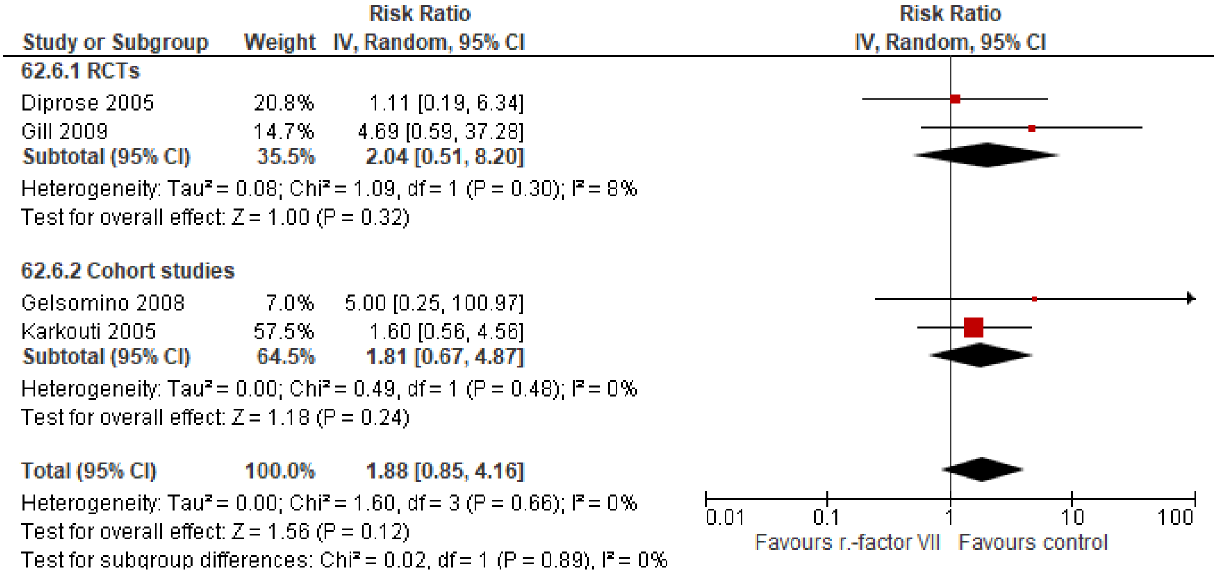

Figure S109: Yank 2011; Intervention/ Exposure: Recombinant factor VII; Outcome: Thromboembolism

Study or Subgroup	Weight	Odds Ratio IV, Random, 95% CI
**63.4.1 RCTs**		
ABSORB China, 2015	3.0%	3.01 [0.12, 75.44]
ABSORB II, 2015	2.5%	5.54 [0.16, 194.25]
ABSORB III, 2015	32.0%	2.09 [0.78, 5.60]
ABSORB Japan, 2015	10.6%	1.00 [0.18, 5.55]
TROFI II, 2016	3.0%	3.04 [0.12, 75.14]
**Subtotal (95% CI)**	**51.1%**	**1.97 [0.90, 4.29]**
Heterogeneity: Tau² = 0.00; Chi² = 1.07, df = 4 (P = 0.90); I² = 0%		
Test for overall effect: Z = 1.70 (P = 0.09)		
**63.4.2 Cohort studies**		
Abizaid, 2015	12.9%	4.03 [0.85, 19.03]
Brugaletta, 2015	20.2%	1.77 [0.51, 6.11]
Cortese, 2015	15.8%	1.83 [0.45, 7.42]
**Subtotal (95% CI)**	**48.9%**	**2.22 [1.00, 4.93]**
Heterogeneity: Tau² = 0.00; Chi² = 0.77, df = 2 (P = 0.68); I² = 0%		
Test for overall effect: Z = 1.96 (P = 0.05)		
**Total (95% CI)**	**100.0%**	**2.09 [1.20, 3.64]**
Heterogeneity: Tau² = 0.00; Chi² = 1.89, df = 7 (P = 0.97); I² = 0%		
Test for overall effect: Z = 2.59 (P = 0.010)		
Test for subgroup differences: Chi² = 0.05, df = 1 (P = 0.83), I² = 0%		

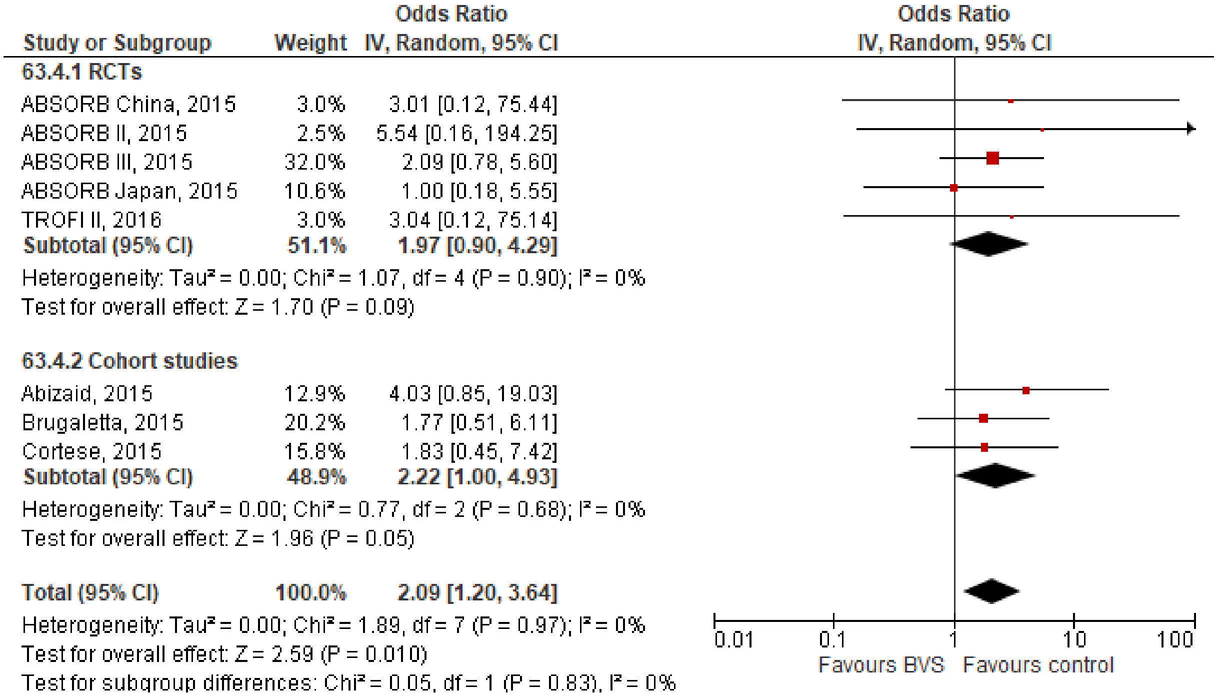

Figure S110: Zhang 2016; Intervention/ Exposure: Everolimus-eluting bioresorbable vascular scaffold; Outcome: Stent thrombosis

Study or Subgroup	Weight	Odds Ratio IV, Random, 95% CI
**63.2.1 RCTs**		
ABSORB China, 2015	6.3%	0.09 [0.00, 1.61]
ABSORB II, 2015	5.2%	0.16 [0.01, 4.06]
ABSORB III, 2015	24.8%	2.60 [0.75, 9.00]
ABSORB Japan, 2015	5.7%	2.55 [0.12, 53.53]
EVERBIO II, 2015	9.6%	0.33 [0.03, 3.28]
**Subtotal (95% CI)**	**51.6%**	**0.66 [0.16, 2.81]**
Heterogeneity: Tau² = 1.20; Chi² = 7.41, df = 4 (P = 0.12); I² = 46%		
Test for overall effect: Z = 0.56 (P = 0.58)		
**63.2.2 Cohort studies**		
Cortese, 2015	11.3%	0.40 [0.05, 3.16]
Costopoulos, 2015	5.7%	0.20 [0.01, 4.13]
Muramatsu, 2014	5.7%	0.33 [0.02, 7.00]
Onuma, 2014	25.7%	0.99 [0.30, 3.35]
**Subtotal (95% CI)**	**48.4%**	**0.63 [0.25, 1.63]**
Heterogeneity: Tau² = 0.00; Chi² = 1.47, df = 3 (P = 0.69); I² = 0%		
Test for overall effect: Z = 0.95 (P = 0.34)		
**Total (95% CI)**	**100.0%**	**0.72 [0.34, 1.55]**
Heterogeneity: Tau² = 0.20; Chi² = 9.41, df = 8 (P = 0.31); I² = 15%		
Test for overall effect: Z = 0.83 (P = 0.40)		
Test for subgroup differences: Chi² = 0.00, df = 1 (P = 0.96), I² = 0%		

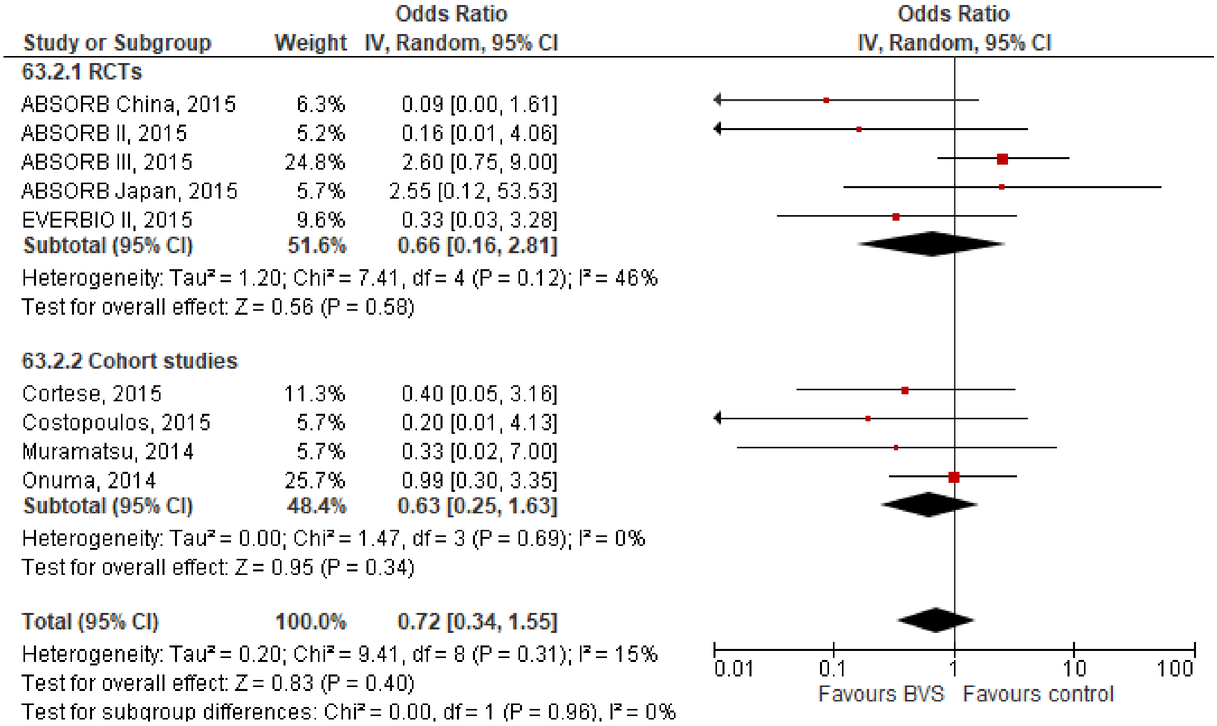

Figure S111: Zhang 2016; Intervention/ Exposure: Everolimus-eluting bioresorbable vascular scaffold; Outcome: All-cause mortality

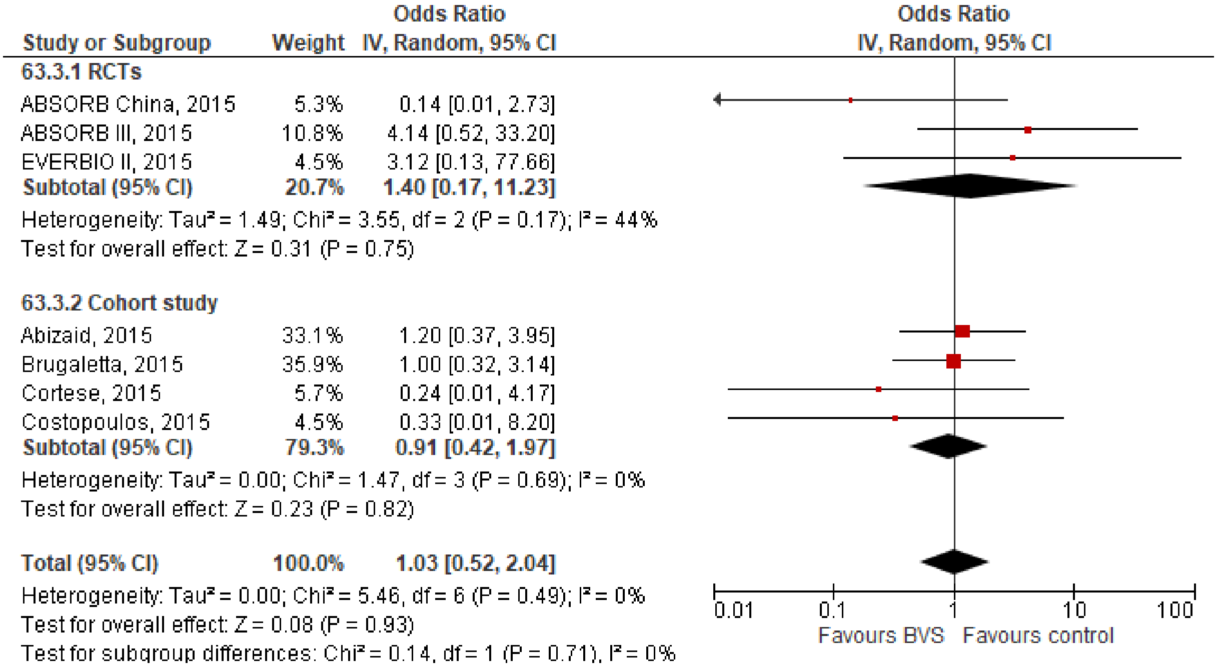

Figure S112: Zhang 2016; Intervention/ Exposure: Everolimus-eluting bioresorbable vascular scaffold; Outcome: Coronary heart disease mortality

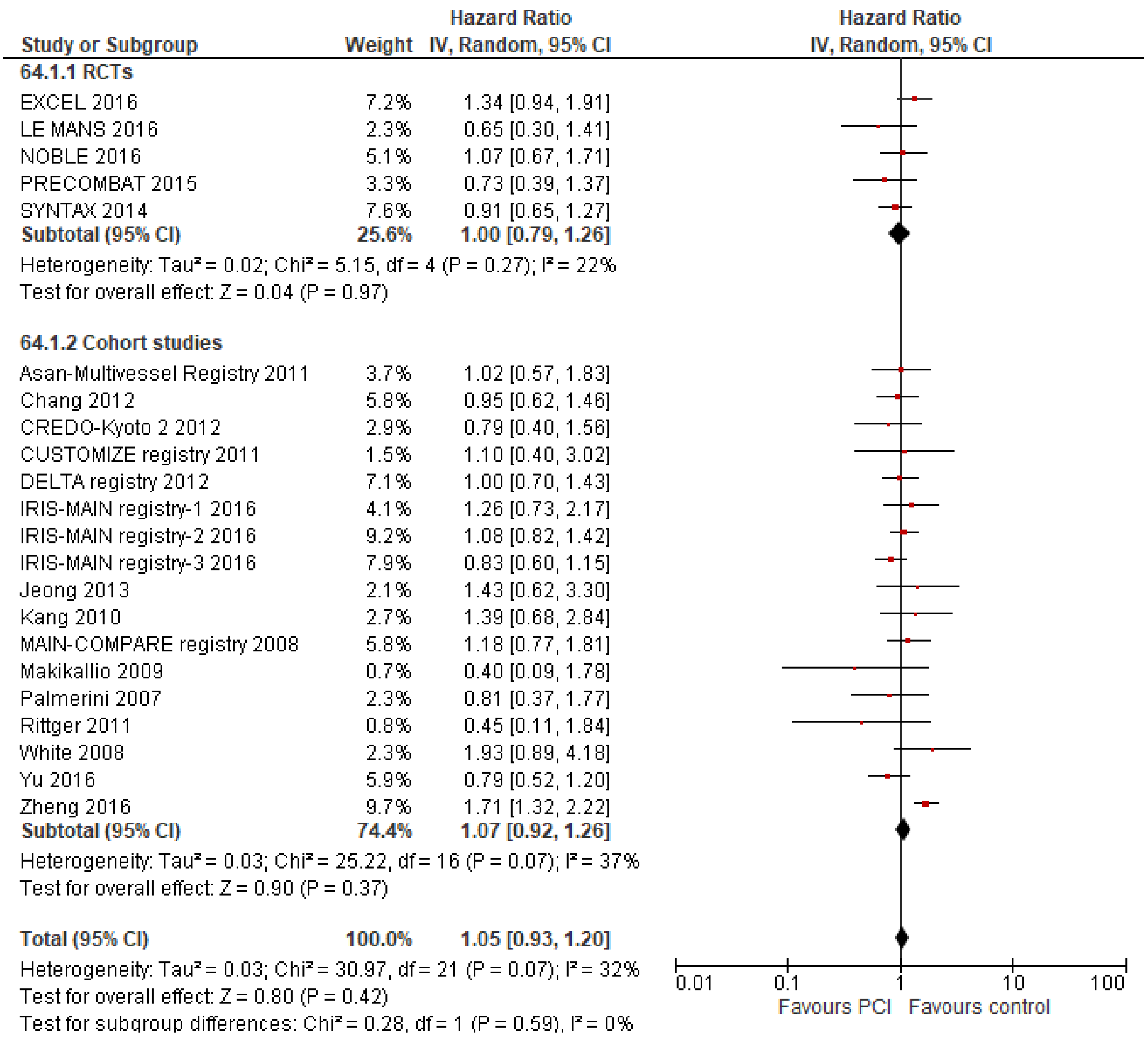

Figure S113: Zhang 2017; Intervention/ Exposure: Percutaneous coronary intervention; Outcome: All-cause mortality

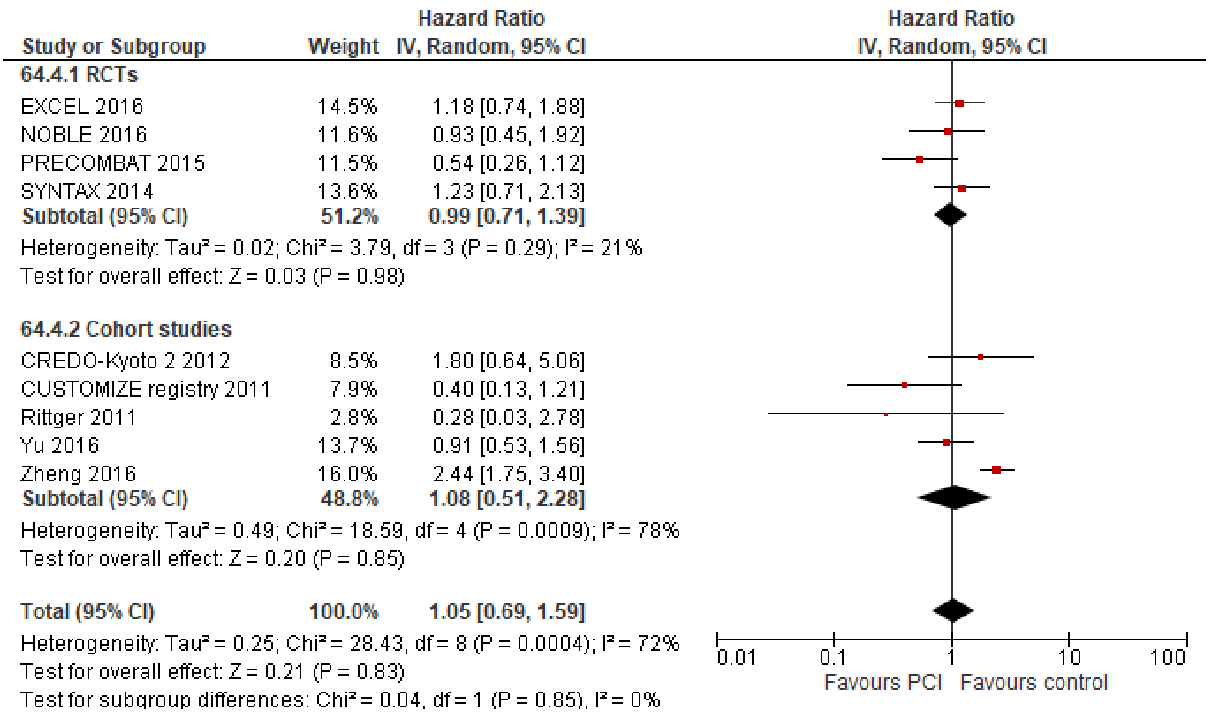

Figure S114: Zhang 2017; Intervention/ Exposure: Percutaneous coronary intervention; Outcome: Cardiovascular mortality

Study or Subgroup	Weight	Hazard Ratio IV, Random, 95% CI
**64.3.1 RCTs**		
EXCEL 2016	19.1%	0.93 [0.67, 1.29]
LE MANS 2016	4.9%	0.88 [0.24, 3.23]
NOBLE 2016	10.9%	2.88 [1.40, 5.92]
PRECOMBAT 2015	5.8%	1.20 [0.37, 3.89]
SYNTAX 2014	13.0%	1.67 [0.91, 3.06]
**Subtotal (95% CI)**	**53.7%**	**1.39 [0.86, 2.26]**
Heterogeneity: Tau² = 0.16; Chi² = 9.34, df = 4 (P = 0.05); I² = 57%		
Test for overall effect: Z = 1.33 (P = 0.18)		
**64.3.2 Cohort studies**		
CREDO-Kyoto 2 2012	6.2%	2.47 [0.81, 7.53]
CUSTOMIZE registry 2011	1.3%	4.80 [0.30, 76.80]
Jeong 2013	3.6%	4.73 [0.99, 22.60]
Yu 2016	13.7%	1.66 [0.94, 2.93]
Zheng 2016	21.5%	2.00 [1.61, 2.48]
**Subtotal (95% CI)**	**46.3%**	**2.00 [1.65, 2.44]**
Heterogeneity: Tau² = 0.00; Chi² = 2.10, df = 4 (P = 0.72); I² = 0%		
Test for overall effect: Z = 6.91 (P < 0.00001)		
**Total (95% CI)**	**100.0%**	**1.69 [1.22, 2.33]**
Heterogeneity: Tau² = 0.12; Chi² = 21.05, df = 9 (P = 0.01); I² = 57%		
Test for overall effect: Z = 3.17 (P = 0.002)		
Test for subgroup differences: Chi² = 1.88, df = 1 (P = 0.17), I² = 46.8%		

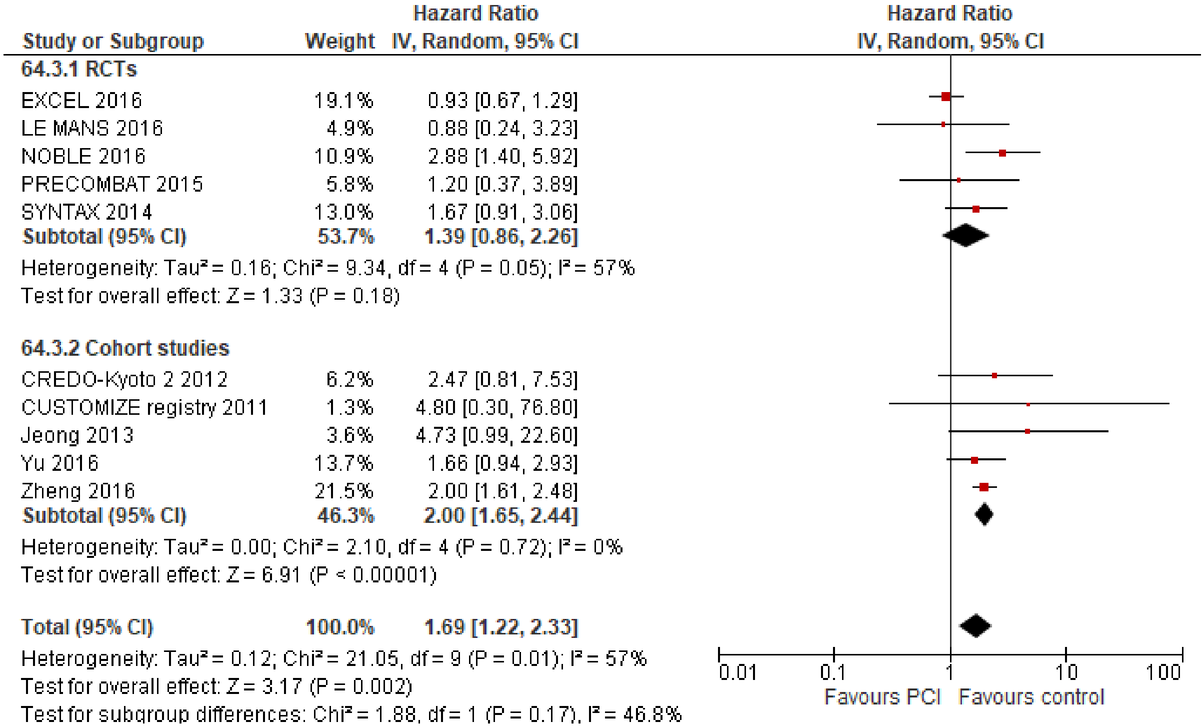

Figure S115: Zhang 2017; Intervention/ Exposure: Percutaneous coronary intervention; Outcome: Myocardial infarction

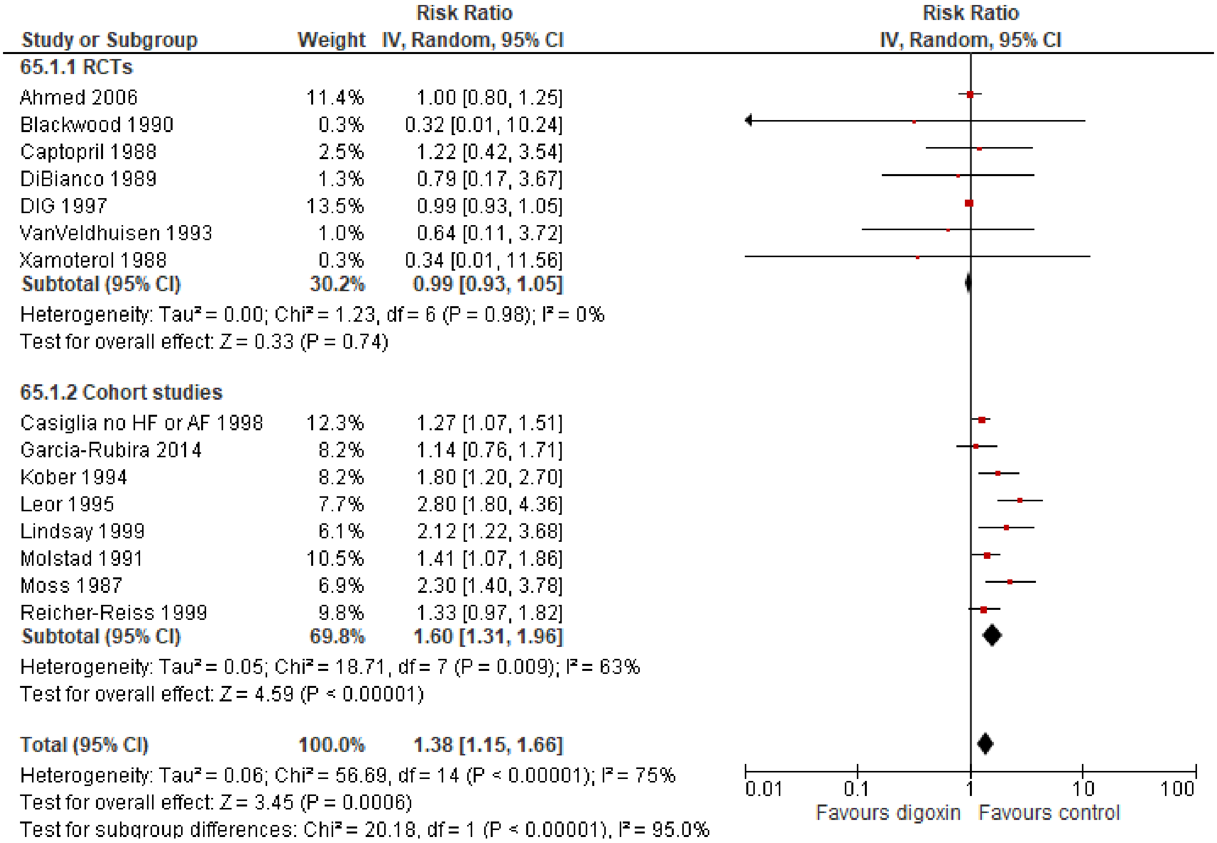

Figure S116: Ziff 2015; Intervention/ Exposure: Digoxin; Outcome: All-cause mortality

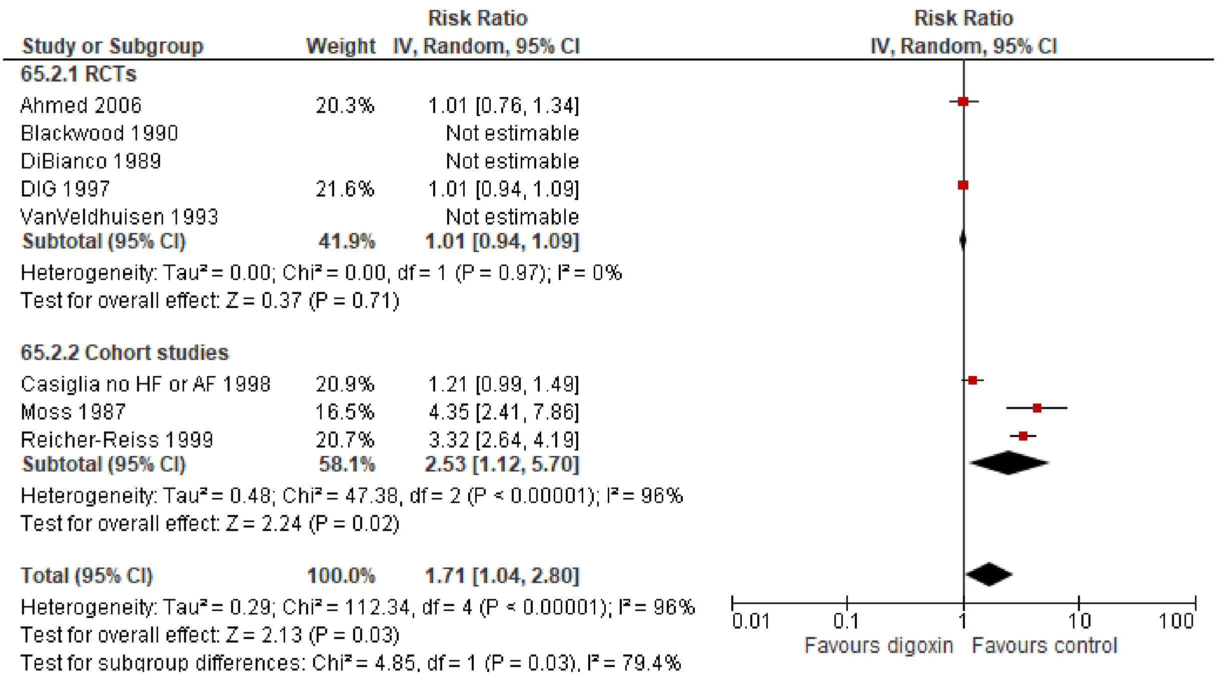

Figure S117: Ziff 2015; Intervention/ Exposure: Digoxin; Outcome: Cardiovascular mortality

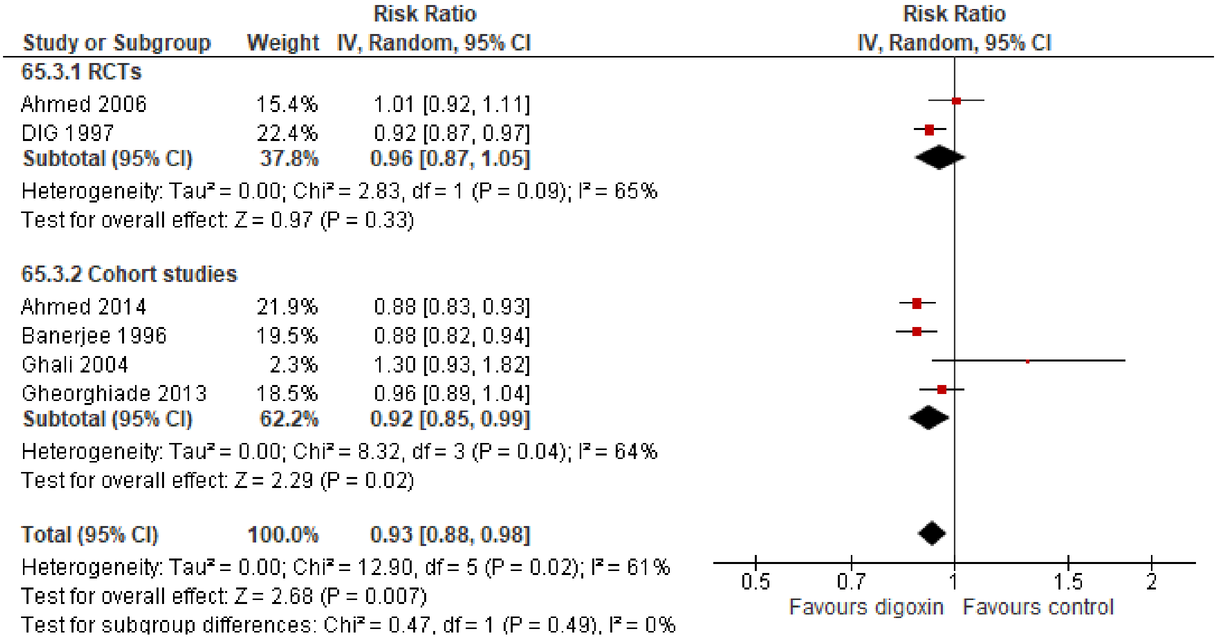

Figure S118: Ziff 2015; Intervention/ Exposure: Digoxin; Outcome: Hospital admission

Supplementary References

1. Abou-Setta AM, Beaupre LA, Rashiq S, Dryden DM, Hamm MP, Sadowski CA, et al. Comparative effectiveness of pain management interventions for hip fracture: a systematic review. Ann Intern Med. 2011;155(4):234-45.

2. Aburto NJ, Ziolkovska A, Hooper L, Elliott P, Cappuccio FP, Meerpohl JJ. Effect of lower sodium intake on health: systematic review and meta-analyses. Bmj. 2013;346:f1326.

3. Ahmad Y, Sen S, Shun-Shin MJ, Ouyang J, Finegold JA, Al-Lamee RK, et al. Intra-aortic Balloon Pump Therapy for Acute Myocardial Infarction: A Meta-analysis. JAMA Intern Med. 2015;175(6):931-9.

4. Alexander DD, Miller PE, Van Elswyk ME, Kuratko CN, Bylsma LC. A Meta-Analysis of Randomized Controlled Trials and Prospective Cohort Studies of Eicosapentaenoic and Docosahexaenoic Long-Chain Omega-3 Fatty Acids and Coronary Heart Disease Risk. Mayo Clin Proc. 2017;92(1):15-29.

5. Alipanah N, Jarlsberg L, Miller C, Linh NN, Falzon D, Jaramillo E, et al. Adherence interventions and outcomes of tuberculosis treatment: A systematic review and meta-analysis of trials and observational studies. PLoS Med. 2018;15(7):e1002595.

6. Anglemyer A, Rutherford GW, Horvath T, Baggaley RC, Egger M, Siegfried N. Antiretroviral therapy for prevention of HIV transmission in HIV-discordant couples. Cochrane Database Syst Rev. 2013(4):Cd009153.

7. Azad MB, Abou-Setta AM, Chauhan BF, Rabbani R, Lys J, Copstein L, et al. Nonnutritive sweeteners and cardiometabolic health: a systematic review and meta-analysis of randomized controlled trials and prospective cohort studies. Canadian Medical Association Journal. 2017;189(28):E929.

8. Barnard S, Kim C, Park MH, Ngo TD. Doctors or mid-level providers for abortion. Cochrane Database Syst Rev. 2015(7):Cd011242.

9. Bellemain-Appaix A, O'Connor SA, Silvain J, Cucherat M, Beygui F, Barthelemy O, et al. Association of clopidogrel pretreatment with mortality, cardiovascular events, and major bleeding among patients undergoing percutaneous coronary intervention: a systematic review and meta-analysis. JAMA. 2012;308(23):2507-16.

10. Bellemain-Appaix A, Kerneis M, O'Connor SA, Silvain J, Cucherat M, Beygui F, et al. Reappraisal of thienopyridine pretreatment in patients with non-ST elevation acute coronary syndrome: a systematic review and meta-analysis. Bmj. 2014;349:g6269.

11. Bloomfield HE, Koeller E, Greer N, MacDonald R, Kane R, Wilt TJ. Effects on Health Outcomes of a Mediterranean Diet With No Restriction on Fat Intake: A Systematic Review and Meta-analysis. Ann Intern Med. 2016;165(7):491-500.

12. Bolland MJ, Leung W, Tai V, Bastin S, Gamble GD, Grey A, et al. Calcium intake and risk of fracture: systematic review. Bmj. 2015;351:h4580.

13. Brenner H, Stock C, Hoffmeister M. Effect of screening sigmoidoscopy and screening colonoscopy on colorectal cancer incidence and mortality: systematic review and meta-analysis of randomised controlled trials and observational studies. Bmj. 2014;348:g2467.

14. Chowdhury R, Stevens S, Gorman D, Pan A, Warnakula S, Chowdhury S, et al. Association between fish consumption, long chain omega 3 fatty acids, and risk of cerebrovascular disease: systematic review and meta-analysis. Bmj. 2012;345:e6698.

15. Chowdhury R, Warnakula S, Kunutsor S, Crowe F, Ward HA, Johnson L, et al. Association of dietary, circulating, and supplement fatty acids with coronary risk: a systematic review and meta-analysis. Ann Intern Med. 2014;160(6):398-406.

16. Chowdhury R, Kunutsor S, Vitezova A, Oliver-Williams C, Chowdhury S, Kiefte-de-Jong JC, et al. Vitamin D and risk of cause specific death: systematic review and meta-analysis of observational cohort and randomised intervention studies. Bmj. 2014;348:g1903.

17. Chung M, Lee J, Terasawa T, Lau J, Trikalinos TA. Vitamin D with or without calcium supplementation for prevention of cancer and fractures: an updated meta-analysis for the U.S. Preventive Services Task Force. Ann Intern Med. 2011;155(12):827-38.

18. Chung M, Tang AM, Fu Z, Wang DD, Newberry SJ. Calcium Intake and Cardiovascular Disease Risk: An Updated Systematic Review and Meta-analysis. Ann Intern Med. 2016;165(12):856-66.

19. Ding M, Huang T, Bergholdt HK, Nordestgaard BG, Ellervik C, Qi L. Dairy consumption, systolic blood pressure, and risk of hypertension: Mendelian randomization study. Bmj. 2017;356:j1000.

20. Fenton JJ, Weyrich MS, Durbin S, Liu Y, Bang H, Melnikow J. Prostate-Specific Antigen-Based Screening for Prostate Cancer: Evidence Report and Systematic Review for the US Preventive Services Task Force. JAMA. 2018;319(18):1914-31.

21. Filippini G, Del Giovane C, Clerico M, Beiki O, Mattoscio M, Piazza F, et al. Treatment with disease-modifying drugs for people with a first clinical attack suggestive of multiple sclerosis. Cochrane Database Syst Rev. 2017;4:Cd012200.

22. Fluri F, Engelter S, Lyrer P. Extracranial-intracranial arterial bypass surgery for occlusive carotid artery disease. Cochrane Database Syst Rev. 2010(2):Cd005953.

23. Gargiulo G, Sannino A, Capodanno D, Barbanti M, Buccheri S, Perrino C, et al. Transcatheter Aortic Valve Implantation Versus Surgical Aortic Valve Replacement: A Systematic Review and Meta-analysis. Ann Intern Med. 2016;165(5):334-44.

24. Hartling L, Dryden DM, Guthrie A, Muise M, Vandermeer B, Donovan L. Benefits and harms of treating gestational diabetes mellitus: a systematic review and meta-analysis for the U.S. Preventive Services Task Force and the National Institutes of Health Office of Medical Applications of Research. Ann Intern Med. 2013;159(2):123-9.

25. Henderson JT, Webber EM, Bean SI. Screening for Asymptomatic Bacteriuria in Adults: Updated Evidence Report and Systematic Review for the US Preventive Services Task Force. JAMA. 2019;322(12):1195-205.

26. Higgins JP, Soares-Weiser K, Lopez-Lopez JA, Kakourou A, Chaplin K, Christensen H, et al. Association of BCG, DTP, and measles containing vaccines with childhood mortality: systematic review. Bmj. 2016;355:i5170.

27. Hopley C, Stengel D, Ekkernkamp A, Wich M. Primary total hip arthroplasty versus hemiarthroplasty for displaced intracapsular hip fractures in older patients: systematic review. Bmj. 2010;340:c2332.

28. Hüpfl M, Selig HF, Nagele P. Chest-compression-only versus standard cardiopulmonary resuscitation: a meta-analysis. Lancet. 2010;376(9752):1552-7.

29. Jamal SA, Vandermeer B, Raggi P, Mendelssohn DC, Chatterley T, Dorgan M, et al. Effect of calcium-based versus non-calcium-based phosphate binders on mortality in patients with chronic kidney disease: an updated systematic review and meta-analysis. Lancet. 2013;382(9900):1268-77.

30. Jefferson T, Di Pietrantonj C, Al-Ansary LA, Ferroni E, Thorning S, Thomas RE. Vaccines for preventing influenza in the elderly. Cochrane Database Syst Rev. 2010(2):Cd004876.

31. Jefferson T, Rivetti A, Di Pietrantonj C, Demicheli V, Ferroni E. Vaccines for preventing influenza in healthy children. Cochrane Database Syst Rev. 2012(8):Cd004879.

32. Jin H, Leng Q, Li C. Dietary flavonoid for preventing colorectal neoplasms. Cochrane Database Syst Rev. 2012(8):Cd009350.

33. Johnston BC, Zeraatkar D, Han MA, Vernooij RWM, Valli C, El Dib R, et al. Unprocessed Red Meat and Processed Meat Consumption: Dietary Guideline Recommendations From the Nutritional Recommendations (NutriRECS) Consortium. Ann Intern Med. 2019;171(10):756-64.

34. Kansagara D, Dyer E, Englander H, Fu R, Freeman M, Kagen D. Treatment of anemia in patients with heart disease: a systematic review. Ann Intern Med. 2013;159(11):746-57.

35. Keag OE, Norman JE, Stock SJ. Long-term risks and benefits associated with cesarean delivery for mother, baby, and subsequent pregnancies: Systematic review and meta-analysis. PLoS Med. 2018;15(1):e1002494.

36. Kredo T, Adeniyi FB, Bateganya M, Pienaar ED. Task shifting from doctors to non-doctors for initiation and maintenance of antiretroviral therapy. Cochrane Database Syst Rev. 2014(7):Cd007331.

37. Li L, Shen J, Bala MM, Busse JW, Ebrahim S, Vandvik PO, et al. Incretin treatment and risk of pancreatitis in patients with type 2 diabetes mellitus: systematic review and meta-analysis of randomised and non-randomised studies. Bmj. 2014;348:g2366.

38. Li L, Li S, Deng K, Liu J, Vandvik PO, Zhao P, et al. Dipeptidyl peptidase-4 inhibitors and risk of heart failure in type 2 diabetes: systematic review and meta-analysis of randomised and observational studies. Bmj. 2016;352:i610.

39. Matthews A, Stanway S, Farmer RE, Strongman H, Thomas S, Lyon AR, et al. Long term adjuvant endocrine therapy and risk of cardiovascular disease in female breast cancer survivors: systematic review. Bmj. 2018;363:k3845.

40. Menne J, Dumann E, Haller H, Schmidt BMW. Acute kidney injury and adverse renal events in patients receiving SGLT2-inhibitors: A systematic review and meta-analysis. PLoS Med. 2019;16(12):e1002983.

41. Mesgarpour B, Heidinger BH, Roth D, Schmitz S, Walsh CD, Herkner H. Harms of off-label erythropoiesis-stimulating agents for critically ill people. Cochrane Database Syst Rev. 2017;8:Cd010969.

42. Moberley S, Holden J, Tatham DP, Andrews RM. Vaccines for preventing pneumococcal infection in adults. Cochrane Database Syst Rev. 2013(1):Cd000422.

43. Molnar AO, Fergusson D, Tsampalieros AK, Bennett A, Fergusson N, Ramsay T, et al. Generic immunosuppression in solid organ transplantation: systematic review and meta-analysis. Bmj. 2015;350:h3163.

44. Navarese EP, Gurbel PA, Andreotti F, Tantry U, Jeong YH, Kozinski M, et al. Optimal timing of coronary invasive strategy in non-ST-segment elevation acute coronary syndromes: a systematic review and meta-analysis. Ann Intern Med. 2013;158(4):261-70.

45. Nelson RL, Furner SE, Westercamp M, Farquhar C. Cesarean delivery for the prevention of anal incontinence. Cochrane Database Syst Rev. 2010(2):Cd006756.

46. Nieuwenhuijse MJ, Nelissen RG, Schoones JW, Sedrakyan A. Appraisal of evidence base for introduction of new implants in hip and knee replacement: a systematic review of five widely used device technologies. Bmj. 2014;349:g5133.

47. Nikooie R, Neufeld KJ, Oh ES, Wilson LM, Zhang A, Robinson KA, et al. Antipsychotics for Treating Delirium in Hospitalized Adults: A Systematic Review. Ann Intern Med. 2019.

48. Ochen Y, Beks RB, van Heijl M, Hietbrink F, Leenen LPH, van der Velde D, et al. Operative treatment versus nonoperative treatment of Achilles tendon ruptures: systematic review and meta-analysis. Bmj. 2019;364:k5120.

49. Pittas AG, Chung M, Trikalinos T, Mitri J, Brendel M, Patel K, et al. Systematic review: Vitamin D and cardiometabolic outcomes. Ann Intern Med. 2010;152(5):307-14.

50. Raman G, Moorthy D, Hadar N, Dahabreh IJ, O'Donnell TF, Thaler DE, et al. Management strategies for asymptomatic carotid stenosis: a systematic review and meta-analysis. Ann Intern Med. 2013;158(9):676-85.

51. Schweizer M, Perencevich E, McDanel J, Carson J, Formanek M, Hafner J, et al. Effectiveness of a bundled intervention of decolonization and prophylaxis to decrease Gram positive surgical site infections after cardiac or orthopedic surgery: systematic review and meta-analysis. Bmj. 2013;346:f2743.

52. Silvain J, Beygui F, Barthelemy O, Pollack C, Cohen M, Zeymer U, et al. Efficacy and safety of enoxaparin versus unfractionated heparin during percutaneous coronary intervention: systematic review and meta-analysis. Bmj. 2012;344:e553.

53. Suthar AB, Lawn SD, del Amo J, Getahun H, Dye C, Sculier D, et al. Antiretroviral therapy for prevention of tuberculosis in adults with HIV: a systematic review and meta-analysis. PLoS Med. 2012;9(7):e1001270.

54. Te Morenga L, Mallard S, Mann J. Dietary sugars and body weight: systematic review and meta-analyses of randomised controlled trials and cohort studies. Bmj. 2013;346:e7492.

55. Thomas RE, Jefferson T, Lasserson TJ. Influenza vaccination for healthcare workers who work with the elderly. Cochrane Database Syst Rev. 2010(2):Cd005187.

56. Tickell-Painter M, Maayan N, Saunders R, Pace C, Sinclair D. Mefloquine for preventing malaria during travel to endemic areas. Cochrane Database Syst Rev. 2017;10:Cd006491.

57. Tricco AC, Zarin W, Cardoso R, Veroniki AA, Khan PA, Nincic V, et al. Efficacy, effectiveness, and safety of herpes zoster vaccines in adults aged 50 and older: systematic review and network meta-analysis. Bmj. 2018;363:k4029.

58. Vinceti M, Filippini T, Del Giovane C, Dennert G, Zwahlen M, Brinkman M, et al. Selenium for preventing cancer. Cochrane Database Syst Rev. 2018;1:Cd005195.

59. Wilson A, Gallos ID, Plana N, Lissauer D, Khan KS, Zamora J, et al. Effectiveness of strategies incorporating training and support of traditional birth attendants on perinatal and maternal mortality: meta-analysis. Bmj. 2011;343:d7102.

60. Wilson HA, Middleton R, Abram SGF, Smith S, Alvand A, Jackson WF, et al. Patient relevant outcomes of unicompartmental versus total knee replacement: systematic review and meta-analysis. Bmj. 2019;364:l352.

61. Yank V, Tuohy CV, Logan AC, Bravata DM, Staudenmayer K, Eisenhut R, et al. Systematic review: benefits and harms of in-hospital use of recombinant factor VIIa for off-label indications. Ann Intern Med. 2011;154(8):529-40.

62. Zhang XL, Zhu L, Wei ZH, Zhu QQ, Qiao JZ, Dai Q, et al. Comparative Efficacy and Safety of Everolimus-Eluting Bioresorbable Scaffold Versus Everolimus-Eluting Metallic Stents: A Systematic Review and Meta-analysis. Ann Intern Med. 2016;164(11):752-63.

63. Zhang XL, Zhu QQ, Yang JJ, Chen YH, Li Y, Zhu SH, et al. Percutaneous intervention versus coronary artery bypass graft surgery in left main coronary artery stenosis: a systematic review and meta-analysis. BMC Med. 2017;15(1):84.

64. Ziff OJ, Lane DA, Samra M, Griffith M, Kirchhof P, Lip GY, et al. Safety and efficacy of digoxin: systematic review and meta-analysis of observational and controlled trial data. Bmj. 2015;351:h4451.

65. Bröckelmann N, Balduzzi S, Harms L, Beyerbach J, Petropoulou M, Kubiak C, et al. Evaluating agreement between bodies of evidence from randomized controlled trials and cohort studies in medical research: a meta-epidemiological study. BMC Med. 2022;20(1):174.
